# Machine Learning Predicts Decompression Levels for Lumbar Spinal Stenosis Using Canal Radiomic Features from Computed Tomography Myelography

**DOI:** 10.3390/diagnostics14010053

**Published:** 2023-12-26

**Authors:** Guoxin Fan, Dongdong Wang, Yufeng Li, Zhipeng Xu, Hong Wang, Huaqing Liu, Xiang Liao

**Affiliations:** 1 Department of Pain Medicine, Huazhong University of Science and Technology Union Shenzhen Hospital, Shenzhen 518056, China; fan06309@163.com (G.F.); xzp1702@163.com (Z.X.); wanghong1234560509@163.com (H.W.); 2 Department of Spine Surgery, Third Affiliated Hospital, Sun Yat-sen University, Guangzhou 510630, China; 3 Department of Orthopaedics, Putuo People’s Hospital, Tongji University, Shanghai 200060, China; dongdongwang1994@126.com; 4 Department of Sports Medicine, Eighth Affiliated Hospital, Sun Yat-sen University, Shenzhen 518033, China; liyf357@mail.sysu.edu.cn; 5 Artificial Intelligence Innovation Center, Research Institute of Tsinghua, Guangzhou 510700, China

**Keywords:** machine learning, lumbar spinal stenosis, computed tomography myelography, decompression level, predictive analysis

## Abstract

Background: The accurate preoperative identification of decompression levels is crucial for the success of surgery in patients with multi-level lumbar spinal stenosis (LSS). The objective of this study was to develop machine learning (ML) classifiers that can predict decompression levels using computed tomography myelography (CTM) data from LSS patients. Methods: A total of 1095 lumbar levels from 219 patients were included in this study. The bony spinal canal in CTM images was manually delineated, and radiomic features were extracted. The extracted data were randomly divided into training and testing datasets (8:2). Six feature selection methods combined with 12 ML algorithms were employed, resulting in a total of 72 ML classifiers. The main evaluation indicator for all classifiers was the area under the curve of the receiver operating characteristic (ROC-AUC), with the precision–recall AUC (PR-AUC) serving as the secondary indicator. The prediction outcome of ML classifiers was decompression level or not. Results: The embedding linear support vector (embeddingLSVC) was the optimal feature selection method. The feature importance analysis revealed the top 5 important features of the 15 radiomic predictors, which included 2 texture features, 2 first-order intensity features, and 1 shape feature. Except for shape features, these features might be eye-discernible but hardly quantified. The top two ML classifiers were embeddingLSVC combined with support vector machine (EmbeddingLSVC_SVM) and embeddingLSVC combined with gradient boosting (EmbeddingLSVC_GradientBoost). These classifiers achieved ROC-AUCs over 0.90 and PR-AUCs over 0.80 in independent testing among the 72 classifiers. Further comparisons indicated that EmbeddingLSVC_SVM appeared to be the optimal classifier, demonstrating superior discrimination ability, slight advantages in the Brier scores on the calibration curve, and Net benefits on the Decision Curve Analysis. Conclusions: ML successfully extracted valuable and interpretable radiomic features from the spinal canal using CTM images, and accurately predicted decompression levels for LSS patients. The EmbeddingLSVC_SVM classifier has the potential to assist surgical decision making in clinical practice, as it showed high discrimination, advantageous calibration, and competitive utility in selecting decompression levels in LSS patients using canal radiomic features from CTM.

## 1. Introduction

Lumbar spinal stenosis (LSS) is a prevalent and disabling disease in the elderly, affecting an estimated 103 million people worldwide [1]. As a common degenerative disease, LSS comprises the narrowing of the spinal canal with subsequent neural compression and often results in reduced quality of life in geriatric patients [2]. In recent decades, severe degenerative LSS has become the major indication of spine surgery among individuals over the age of 65 years [3,4]. When conservative treatments fail, surgical decompression is typically recommended. Recently, spinal endoscopic decompression has become the cutting edge of minimally invasive spine surgery for managing LSS [5], and it has been favored over open surgery techniques by spine surgeons [6]. A recent network meta-analysis indicated that compared to other surgical interventions for LSS, endoscopic decompression was less invasive and associated with lower complication rates and shorter hospitalization times [7]. However, the effectiveness of spinal endoscopic decompression heavily relies on the accurate diagnosis and localization of the decompression levels, which remains challenging, particularly in multi-level LSS patients.

Accurately predicting decompression levels before surgery would undoubtedly benefit surgical decision making for LSS patients. In recent years, machine learning (ML) and deep learning (DL) have demonstrated their advantages in many medical fields, especially in predictive analysis [8]. In studies associated with LSS, researchers have applied ML algorithms for quantitative and qualitative analysis [9,10,11,12]. For example, Gaonkar et al. used an ML technique to establish a normative range of spinal canal areas in the lumbar spine from MR images [9]. Huber et al. applied ML to detect and grade lumbar stenosis, in which a decision tree classifier with texture analysis showed a higher reproducibility in LSS detection [10]. Researchers, such as Hallinan et al. [11], Bharadwaj et al. [13], Won et al. [12], Han et al. [14], and Altun et al. [15], have introduced DL models for the automatic diagnosis, detection, and classification of LSS using lumbar MR images, and achieved comparable performances with radiologists. In outcome predictions for LSS, researchers have also reported several ML studies such as patient-reported outcome measures [16,17], clinical outcome predictions [18,19,20,21], patient-specific outcomes (such as patient resource utilization [22], non-home discharge placement prediction [23], prolonged opioid prescriptions [24], and prolonged length of hospital stay [25]). Other ML studies, such as surgical candidacy prediction [26] and prior authorization approval prediction [27], have been also reported.

Radiomics is one of the most popular techniques for extracting high-throughput image features in quantitative analysis studies [28]. Radiomics is a process that converts digital medical images (MR, CT, etc.) to mineable high-throughput data and analyzes these data, which might result in decision support for diagnosis, prognosis, and prediction approaches for personalizing management and treatment [29]. Combined with ML techniques, the accurate, quantitative, and interpretative evaluation of regions of interest (ROIs) of the radiomic data could be achieved, which has been expected to assist in the diagnosis, classification, and decision making of certain diseases [30].

However, to the best of our knowledge, few ML studies are available that aid in predicting decompression levels in LSS patients. Jujjavarapu et al. [31] developed a DL model to predict decompression surgery for lumbar disc herniation and LSS patients using patients’ demographics, diagnosis and procedure codes, drug names, and diagnostic imaging reports. However, the AUCs of the DL model were 0.725 and 0.655 in early and late surgery prediction, respectively. An ML study introduced by Wilson et al. [26] used the percentage reduction in the canal area, at each disc level, as quantitative MRI predictors, and they achieved a high discrimination (an AUC of 0.88) in predicting surgical candidacy for LSS patients. However, their studies did not predict decompression levels. Roller et al. [32] applied SpineNet to predict decompression levels in LSS patients using quantified features of sagittal MRI, but it was not available using radiomic features or CT myelography (CTM).

Therefore, the aim of this study was to develop ML classifiers utilizing radiomic features based on CTM images to predict decompression levels in LSS patients. We present the following article in accordance with the TRIPOD reporting checklist.

## 2. Materials and Methods

### 2.1. Data Collection

Prior to data extraction, institutional review board (IRB) approval was obtained (KY-2022-031-01). A waiver of consent was granted due to the retrospective nature of the study and minimal risk involved. The medical records of LSS patients who underwent CTM examination in our radiology department between January 2015 and December 2021 were retrospectively reviewed. The inclusion criteria were as follows: (1) single-level or multi-level LSS patients with or without radicular symptoms; (2) patients who underwent spinal decompressive surgeries and owned preoperative CTM scans; (3) age > 18 years. The exclusion criteria were as follows: (1) those who experienced complications with intraspinal tumors and congenital spinal malformations (tethered cord, diplomyelia, etc.); (2) low-quality CTM images due to metal implants in the patient’s body; (3) unsuccessful contrast injection (e.g., epidural distribution of the contrast). The scan settings of the CTM scans were as follows: slice thickness of 1–5 mm (median 3 mm), 120–135 kV (median 120 kV), 60–200 mA (median 200 mA). Baseline characteristics of these patients including age, gender, and decompression levels were recorded.

By tracking and reviewing the medical records in our institution, these patients were identified whether they underwent elective spinal decompression surgery. Given that the decompression surgeries of the patient cohort are established facts, the decompression or non-decompression levels could be regarded as the ground truth. Thus, the lumbar disc levels of decompression were recorded and regarded as the decompression levels, while the rest of the lumbar disc levels were regarded as the non-decompression levels.

### 2.2. Radiomic Features

The CTM scan of the lumbar spine consists of five levels, indicating that each case may have at least one decompression level. Therefore, using case samples to develop ML models would be inappropriate. Conversely, utilizing the disc levels as samples to develop the ML model can predict the probability of each level being a decompression level, which is undoubtedly more valuable.

In this study, the osseous spinal canals of each disc level with the most stenotic slices on the CTM scans were delineated on 3D Slicer 4.11 [33,34]. The manual canal delineations were initially conducted by a human expert, who was experienced in lumbar CTM image reading and 3D Slicer operation, and then reviewed by another two experts with similar experience. Any disagreements of the delineated areas of the canal segmentation were discussed and reviewed by these three experts. 

**Figure 1:**
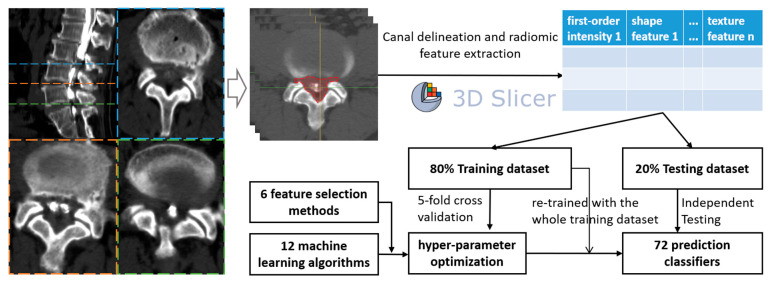
Flow chart of the study design.

As the ROIs were delineated, the PyRadiomic module of Slicer was used to extract the radiomic features (texture features, first-order intensity, shape features, etc.) from all levels of each patient, generating csv files of each level sample. Finally, all samples were merged using Python 3.8.13 (Python Software Foundation) and used to develop ML classifiers (Figure 1).

### 2.3. Model Development

Six common feature selection methods were utilized in this study, including embedding tree, embedding random forest (RF), embedding logistic regression (embeddingLR), embedding linear support vector classifier (embeddingLSVC), maximal information coefficient (MIC), and recursive feature elimination (RFE). The feature importance of each feature selection method was used to rate the most valuable radiomic features to predict decompression levels in LSS patients. Additionally, twelve ML algorithms were utilized, namely, multilayer perceptron (MLP), gradient boosting, adaptive boosting (AdaBoost), logistic regression (LR), bagging, linear discriminant analysis (LDA), RF, extra trees, support vector machine (SVM), decision tree, k-nearest neighbor (KNN), and Gaussian naïve Bayes (NB). Therefore, a total of 72 initial classifiers from 6 × 12 combinations were developed.

The included patient data were divided into two sub-datasets (training:testing = 8:2). Firstly, six common feature selection methods were utilized for the training dataset, so six feature-selected training datasets were created. With the six feature-selected training datasets, we endeavored to optimize the hyper-parameters for each of the 12 ML algorithms. The optimization process of the hyper-parameters was achieved using 5-fold cross-validation with the GridSearchCV or RandomSearchCV function in the scikit-learn package, and the scoring metric was the area under the curve of the receiver operating characteristic (ROC-AUC).

We used the SelectFromModel function from the Python package scikit-learn to conduct the embedding classifier feature selection with the training dataset. SelectFromModel is a meta-transformer that can be used alongside any estimator that assigns importance to each feature through a specific attribute (such as coef_, feature_importances_) or via an importance_getter callable after fitting. The features are considered unimportant and removed if the corresponding importance of the feature values are below the provided threshold (or mean importance when no threshold is provided). Thus, the feature importance could be quantified using the SelectFromModel function of the scikit-learn package, and the initial number of radiomic features was 15. Additionally, the weaker learners (base estimator) of the meta-algorithms, like bagging, gradient boosting, and Adaboost, used in this study were logistic regression, LDA, SVM, KNN, GaussianNB, and decision tree. The above-mentioned six base estimators were all defined via their own optimized hyper-parameters.

With the optimized hyper-parameters (Table S1) for each of the 12 ML algorithms, we retrained them with the six feature-selected training datasets, without any cross-validation. As a result, a total of 72 ML classifiers were obtained and assessed. The ROC-AUCs in the independent testing were the primary indicators to assess the prediction performance for all ML classifiers, and the precision–recall AUC (PR-AUC) was the secondary indicator. Additionally, the calibration curves and the Decision Curve Analysis (DCA) were also obtained to assess the predictive consistency and the clinical utility of ML classifiers, respectively. Other indicators of discrimination ability included sensitivity, specificity, accuracy, positive predictive value (PPV), and negative predictive value (NPV).

Top ML classifiers were selected based on the overall evaluation of the discrimination ability, the predictive consistency, and the clinical utility of all 72 ML classifiers in the independent testing datasets. Firstly, top classifiers with ROC-AUCs over 0.90 were initially selected, and differences in ROC-AUCs were statistically detected. Then, we also compared the top classifiers with PR-AUCs over 0.80, and differences in PR-AUCs were statistically detected. Additionally, other indicators of discrimination ability were also compared for the top-n selected classifiers if necessary. Finally, the Brier score was used to assess predictive consistency, and the Net benefit was used to assess the clinical utility of the top-n selected classifiers.

### 2.4. Statistical Analysis

The predictions for all classifiers were defined as category outcomes: decompression level or not. The differences in baseline characteristics between decompression level and non-decompression level cohorts were compared using a simple *t*-test and chi-squared test. The ROCs were compared using the Delong test, and the PR-AUCs were compared using the Wilcoxon rank sum test. A *p* < 0.05 was considered a statistically significant difference.

## 3. Results

A total of 219 patients and 1095 disc levels were identified and enrolled in this study. Among them, the non-decompression level samples had 711 lumbar disc levels, and the decompression level samples had 384 lumbar disc levels. The baseline characteristics of the training dataset and testing dataset are shown in Table 1. No significant differences were observed in baseline characteristics.

**Figure 2:**
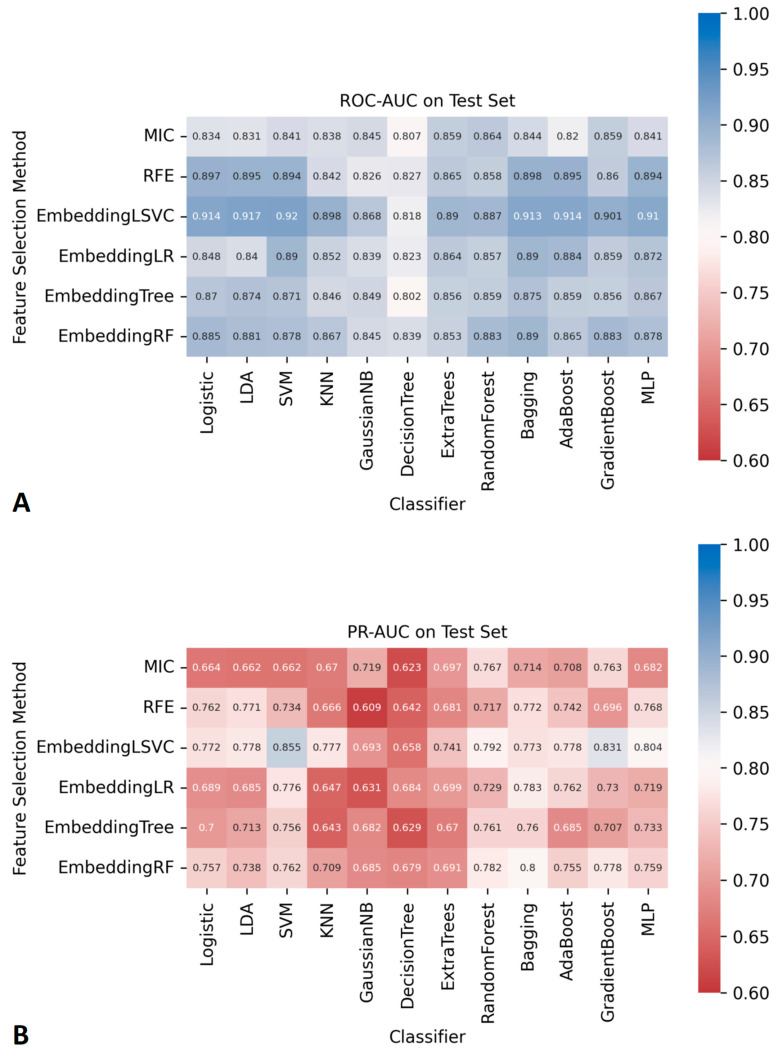
The AUCs of 72 prediction models constructed using different feature selection methods and ML algorithms. (**A**) ROC-AUCs. (**B**) PR-AUCs.

The AUCs of all ML classifiers during the cross-validation and the training process can be found in Figure S1. The AUCs of the 72 ML classifiers in the independent testing datasets are shown in Figure 2. Among them, seven ML classifiers, based on the embedding LSVC feature selection method, achieved satisfactory ROC-AUCs (higher than 0.90). No other ML classifiers with ROC-AUCs over 0.90 were found using ML with other feature selection methods. Similarly, two classifiers with embeddingLSVC achieved satisfactory PR-AUCs (higher than 0.80) in the independent testing datasets. No other ML classifiers with PR-AUCs over 0.80 were found using ML with other feature selection methods. The 95% CI of the ROC-AUCs and PR-AUCs of all classifiers are shown in Tables S2 and S3. To sum up, the embeddingLSVC seems to be the best feature selection method to extract the most valuable radiomic features of CTM.

As shown in Figure 2, there were seven classifiers with ROC-AUCs over 0.90 in the independent testing datasets, and the top classifier was EmbeddingLSVC_SVM (ROC-AUC, 0.920). However, the Delong tests indicated no significant differences in the ROC-AUCs among these top seven classifiers (Table 2). Similarly, there were two classifiers with PR-AUCs over 0.80 in the independent testing datasets (Table 3). EmbeddingLSVC_SVM was the top PR-AUC classifier (PR-AUC, 0.855), and EmbeddingLSVC_GradientBoost was the second best PR-AUC classifier (PR-AUC, 0.831). The Wilcoxon rank sum test revealed a significant difference in the PR-AUCs among these top two classifiers. In brief, the discrimination ability of the EmbeddingLSVC_SVM classifier seemed to be slightly superior to that of the EmbeddingLSVC_GradientBoost classifier. The results of the Wilcoxon rank sum test of the top 10 PR-AUC classifiers are shown in Table S4.

**Figure 3:**
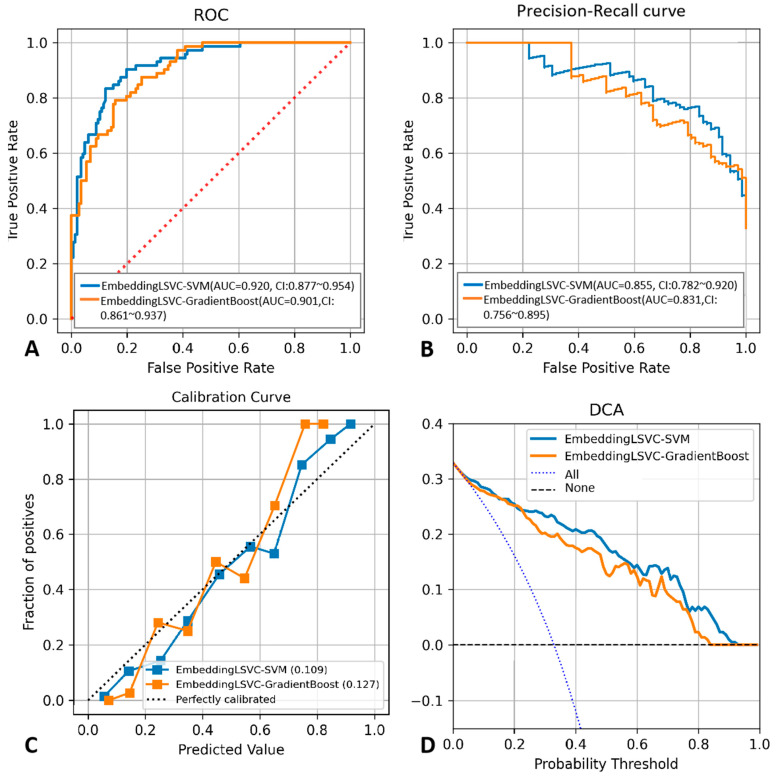
Performance comparison between the EmbeddingLSVC_SVM classifier and the EmbeddingLSVC_GradientBoost classifier. (**A**) ROC curve. (**B**) Precision–recall curve. (**C**) Calibration curve. (**D**) Decision Curve Analysis.

To improve the reproducibility of the study, we disclosed the hyper-parameter settings of the top two classifiers as follows: The kernel of EmbeddingLSVC-SVM was “rbf”, while the max depth of EmbeddingLSVC_GradientBoost was 2, and the n_estimators of EmbeddingLSVC_GradientBoost was 40. Other indicators reflecting the discrimination ability of the top two ML classifiers are shown in Table 4, while the probability threshold was set to 0.5. The EmbeddingLSVC_SVM classifier was superior across all discrimination indicators. To further compare the top two ML classifiers, the calibration curves and the DCA curves of the top two ML classifiers were also obtained (Figure 3). When considering predictive consistency, the Brier scores of the top two ML classifiers were similar, but the EmbeddingLSVC_SVM classifier seemed to be slightly higher. For clinical utility, however, the Net benefit of the Brier score of the EmbeddingLSVC_SVM classifier showed slight superiority over the EmbeddingLSVC_GradiantBoost classifier on the DCA curves. In summary, the EmbeddingLSVC_SVM classifier seems to be the optimal classifier.

**Figure 4:**
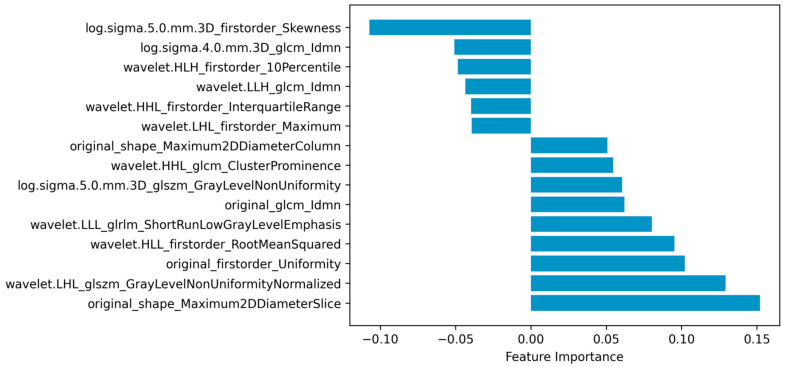
Radiomic features selected via embedding linear support vector classifier (EmbeddingLSVC).

As embeddingLSVC was the optimal feature selection method and the EmbeddingLSVC_SVM classifier was the optimal classifier, the interpretation of the selected radiomic features was vital for introducing the optimal classifier into clinical practice. Permutation importance showed that the top important radiomic feature was “original shape Maximum2DDiameterSlice”, which indicates that the space of the bony spinal canal was the most important predictor to identify the decompression levels. The second important radiomic feature was “wavelet.LHL.glszm.GrayLevelNonUniformityNormalized”, which indicated that the non-uniformity of the gray level of CTM was a hardly quantified but valuable predictor. The third and fourth important features were “original.first.order.Uniformity” and “wavelet.HLL.firstorder.RootMeanSquared”, respectively. These two radiomic features belonged to the first-order intensity features, which might be subjectively perceivable but also hard to quantify. The fifth importantradiomic feature was “wavelet.LLL.glrlm.ShortRunLowGrayLevelEmphasis”, which emphasized the importance of a low gray level of the ROI. In summary, the top five important radiomic predictors included two texture features, two first-order intensity features, and one shape feature, all of which were eye-discernible but hard to objectively quantify, except for the shape feature (Figure 4). The complete results are available in the compressed Supplementary Files.

## 4. Discussion

ML predictions based on CTM radiomics were expected to assist in the decision-making process for the surgical management of LSS. In this study, we found that embeddingLSVC was the optimal feature selection method for extracting the most valuable radiomic predictors. Additionally, the EmbeddingLSVC_SVM classifier appeared to be the optimal ML classifier to facilitate the identification of decompression levels for LSS patients. Finally, this study also revealed that the space of the bony spinal canal was an old-fashioned but the most important predictor. To the best of our knowledge, this is the first study to utilize ML algorithms and radiomic data to predict decompression levels in LSS patients using only CTM images.

Surgical decompression using a variety of techniques (such as single-level or multi-level decompression, micro-decompression, open decompression, and decompression with or without spinal fusion) has been proven to be beneficial to long-term outcomes for LSS patients [35,36,37,38]. However, it remains challenging for surgeons to determine decompression levels, especially for multi-level stenosis. A randomized controlled trial indicated that micro-decompression was effective in the treatment of multi-level LSS, with superior results regarding less back pain postoperatively and less blood loss and soft tissue dissection compared to open surgery [39]. However, the accurate identification and localization of decompression levels are important prerequisites for micro-decompression; otherwise, extensive decompression or open spinal surgery is required for multiple suspected levels. Although the clinical outcomes are similar [38,39], multi-level decompression and open spinal surgery have disadvantages, such as more surgical trauma, more back pain postoperatively, a larger volume of estimated blood loss, longer hospital stays, and so on [38,39,40,41]. Selective decompression, when considering decompressive surgery for suspected multi-level LSS patients, could help avoid the risk and invasiveness of extensive procedures [42]. Therefore, improving the ability of spine surgeons to identify decompression levels is essential, and the presented prediction classifier could serve as helpful assistance in the surgical decision-making process.

In clinical practice, the identification of decompression levels largely depends on radiologic examinations and surgeon experience. Multiple imaging modalities, such as MRI, CT, and CTM, have been widely used in different situations [43]. Among them, MRI is regarded as the standard imaging modality for spinal stenosis assessment, but it might underestimate the severity of stenosis [44,45,46]. In some cases (e.g., when MRI findings were inconclusive or ambiguous), CTM was an adjunct imaging modality in clinical practice [47], as it showed advantages in detecting multi-level LSS [48]. Moreover, with the increasing use of spinal instrumentation, CTM still remains an alternative imaging method in the investigation of LSS [49]. However, young physicians are unfamiliar with interpreting this old-fashioned imaging modality, and even experienced physicians may fail to objectively and quantitatively assess LSS. In recent studies, quantitative texture analysis has been proven to be a valuable tool in detecting distinct quantifiable differences in tissues that cannot be depicted via qualitative visual assessments [18,50,51]. Therefore, it is necessary to utilize radiomic techniques and ML algorithms to mine valuable CTM features.

Radiomics is one of the most popular techniques for extracting high-throughput image features of ROIs from multi-dimensional data [28]. When combined with ML techniques, radiomic data can be used to achieve the accurate, quantitative, and interpretative evaluation of ROIs, which is expected to assist in the diagnosis, classification, and decision making of diseases [30]. In this study, we found that the shape feature of the spinal canal on CTM images was the most important predictor for identifying decompression levels in LSS patients. Evidently, the shape features reflect the diameter of the osseous spinal canal, which correlates spinal degeneration with the narrowing of space around the nerves. The second important predictor reflects the non-uniformity of the gray level of CTM, which should be eye-discernible, although hard to quantify but valuable, as it might reflect the heterogeneous content of the spinal canal (e.g., the spinal nerves, ligamentum flavum, and myelogram contrast). The two first-order intensity features might reflect the level of the myelogram contrast in the spinal canal, which is consistent with clinical practice, as the non-distribution of myelogram contrast at certain levels is usually regarded as the imaging sign of the culprit level. The fifth important predictor also reflects the texture of the spinal canal like the second best predictor, but it mainly emphasizes the contribution of the low gray level to the selection of decompression levels. Interestingly, all the above five predictors should be subjectively perceptible to human eyes, but they were hard to objectively quantify, except for the shape feature, and their importance ranking has never been disclosed.

While identifying radiomic predictors undoubtedly increased the interpretability of decompression levels on CTM, integrating these predictors into personalized prediction would increase applicability. Jujjavarapu et al. [31] developed a DL model to use patients’ clinical data to predict decompression surgery for lumbar disc herniation and LSS patients. The DL models achieved a mean AUC of 0.725 for early surgery and 0.655 for late surgery. However, the radiomic data were not used in this study. An ML study by Wilson et al. [26] used the percentage reduction in the canal area at each disc level as quantitative MRI predictors, and they achieved a high discrimination in predicting surgical candidacy for LSS patients (AUCs of L1–L5 were 0.71 to 0.89, and the overall AUC was 0.88). André et al. [52] assessed the feasibility of training a DL model on synthetic patients generated from EHR data to predict the positive and negative outcomes of decompression surgery, with an AUC of 0.78. However, their study did not provide the indicators of precision and sensitivity. The current study found that the optimal classifier could achieve superior AUCs (an ROC-AUC of 0.920 and a PR-AUC of 0.855) compared to almost all classifiers mentioned above, with balanced specificity (0.833) and sensitivity (0.864). Additionally, a slightly higher sensitivity might reduce the risks of missing decompression levels, in which patients might undertake the surgery without symptom relief. The improved discrimination of our optimal classifier can be explained by the high-throughput radiomic features we adopted, which included shape, first-order intensity, texture features, etc. Moreover, we also adopted six feature selection methods to identify the valuable predictors and deployed 12 ML algorithms to train the predictive classifiers. We found that embeddingLSVC was the optimal feature selection method and that the EmbeddingLSVC_SVM classifier was the optimal ML classifier via comprehensive comparisons. It should be mentioned that the EmbeddingLSVC_SVM classifier not only demonstrated a high discrimination ability but also favorable predictive consistency and superior clinical utility. Clinical utility is vital when considering a classifier to assist in decision making, and notably, the EmbeddingLSVC_SVM classifier happened to exhibit a higher Net benefit in the DCA curve.

Several limitations should be noted in this study. First, the prediction performance of the optimal ML classifier was not compared with clinical physicians of varying expertise levels. This is because the main aim of this study was to explore the assistance potential of ML algorithms in predicting decompression levels in LSS patients. However, we could not simply draw the conclusion that the optimal ML classifier is good enough for decision making in clinical practice. This is because surgeons need not only useful information from preoperative radiology images to predict decompression levels in LSS patients but also clinical information like the localization of radiating pain if the patient presents with this. Therefore, further study should investigate the add-on value of the optimal ML classifier in human identification of decompression levels. Second, the current study lacked external validation. We can overcome these obstacles by including more data from other institutions to further validate the ML classifiers. Last but not least, we selected decompression levels instead of culprit levels as the prediction outcome, as the ground truth of the latter is hard to objectively confirm due to the retrospective nature of this study.

## 5. Conclusions

ML successfully extracted valuable and interpretable radiomic features from the spinal canal using CTM images and accurately predicted decompression levels in LSS patients. The EmbeddingLSVC_SVM classifier has the potential to assist in surgical decision-making processes in clinical practice, as it showed high discrimination, favorable calibration, and advantageous utility in selecting decompression levels in LSS patients using canal radiomic features from CTM. Future studies with improved algorithms, multi-center data, and human comparisons are needed to further confirm the application potential of the optimal prediction classifier.

## Figures and Tables

**Table 1:** Basic characteristics of 219 patients.

Subjects	Training Dataset (*n* = 176)	Testing Dataset (*n* = 43)	*p*-Value
Age (years)	61.59 ± 13.43	57.47 ± 14.95	0.126
Gender			0.978
Male	83	21	
Female	93	22	
Decompression level			0.233
L1/L2	4	0	
L2/L3	18	5	
L3/L4	47	14	
L4/L5	116	41	
L5/S1	90	48	

Data are presented as mean ± SD.

**Table 2:** Delong tests of comparisons among top embeddingLSVC_ML classifiers with ROC-AUC over 0.90 in the independent testing datasets.

Ranking	ROC-AUC	*p*-Values	SVM	LDA	AdaBoost	LR	Bagging	MLP	GradientBoost
1	0.920	SVM	1	-	-	-	-	-	-
2	0.917	LDA	0.820	1	-	-	-	-	-
3	0.914	AdaBoost	0.632	0.316	1	-	-	-	-
4	0.914	LR	0.602	0.268	0.900	1	-	-	-
5	0.913	Bagging	0.665	0.521	0.867	0.698	1	-	-
6	0.910	MLP	0.328	0.177	0.452	0.504	0.439	1	-
7	0.901	GradientBoost	0.139	0.230	0.332	0.365	0.339	0.510	1

ML—machine learning; ROC—receiver operating characteristic; AUC—the area under the curve; MLP—multilayer perceptron; AdaBoost—adaptive boosting; LDA—linear discriminant analysis; SVM—support vector machine; LR—logistic regression; GradientBoost—gradient boosting; LSVC—linear support vector classifier.

**Table 3:** Wilcoxon rank sum test of comparisons among top embeddingLSVC_ML classifiers with PR-AUCs over 0.80 in the independent testing datasets.

Ranking	PR-AUC	*p*-Values	SVM	GradientBoost
1	0.855	SVM	1	<0.001
2	0.831	GradientBoost	<0.001	1

ML—machine learning; PR-AUC—the area under the curve of the precision–recall curve; SVM—support vector machine; GradientBoost—gradient boosting; LSVC—linear support vector classifier.

**Table 4:** Other indicators of discrimination ability for the top two selected classifiers.

EmbeddingLSVC_ML	Sensitivity	Specificity	Accuracy	PPV	NPV
SVM	0.833	0.864	0.854	0.750	0.864
GradientBoost	0.694	0.850	0.799	0.694	0.850

ML—machine learning; PPV—positive predictive value; NPV—negative predictive value; SVM—support vector machine; GradientBoost—gradient boosting; LSVC—linear support vector classifier.

## Data Availability

The complete results presented in the study are included in the article/Supplementary Materials, and the pertinent codes in the study are shared in the following link (https://github.com/Huatsing-Lau/CTMLSS_DL_Classify (accessed on 2 November 2023)). Further inquiries can be directed to the corresponding authors.

## Supplementary Materials

The following supporting information can be downloaded at: https://www.mdpi.com/article/10.3390/diagnostics14010053/s1. Table S1: The hyper-parameters used for model training. Table S2: The 95%CI of ROC-AUCs in the independent testing datasets. Table S3: The 95%CI of PR-AUCs in the independent testing datasets. Table S4: The *p*-values of comparisons among the top 10 PR-AUC classifiers in the independent testing datasets. Figure S1: Segmental sample data distribution and results of model (A) based on CTM data; (B) results of ROC-AUC on the CV validation dataset; (C) results of ROC-AUC on the training dataset; (D) results of PR-AUC on the training datasets.
