# Supplementary figures and images for: Machine Learning Predicts Decompression Levels for Lumbar Spinal Stenosis Using Canal Radiomic Features from Computed Tomography Myelography (part 2 of 2)
Source: Diagnostics (Basel). 2023 Dec 26;14(1):53. doi: 10.3390/diagnostics14010053 (PMC10795799; doi:10.3390/diagnostics14010053)

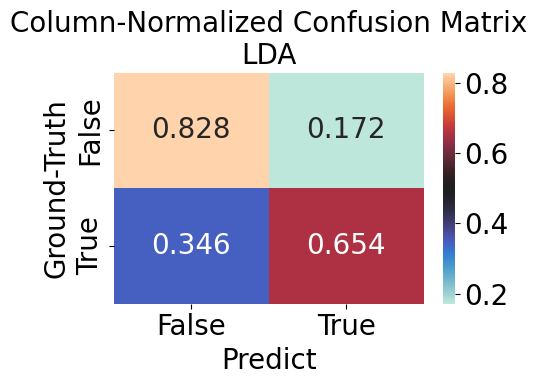

Supplement: Supplementary file 1 [file diagnostics-14-00053-s001.zip › Results of all classifiers/EmbeddingLR/LDA/Train Set/Column-Normalized Confusion Matrix LDA.png]

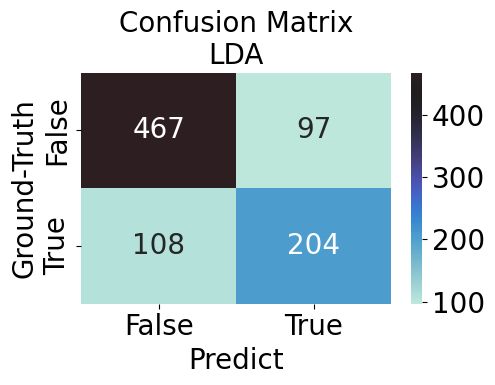

Supplement: Supplementary file 1 [file diagnostics-14-00053-s001.zip › Results of all classifiers/EmbeddingLR/LDA/Train Set/Confusion Matrix LDA.png]

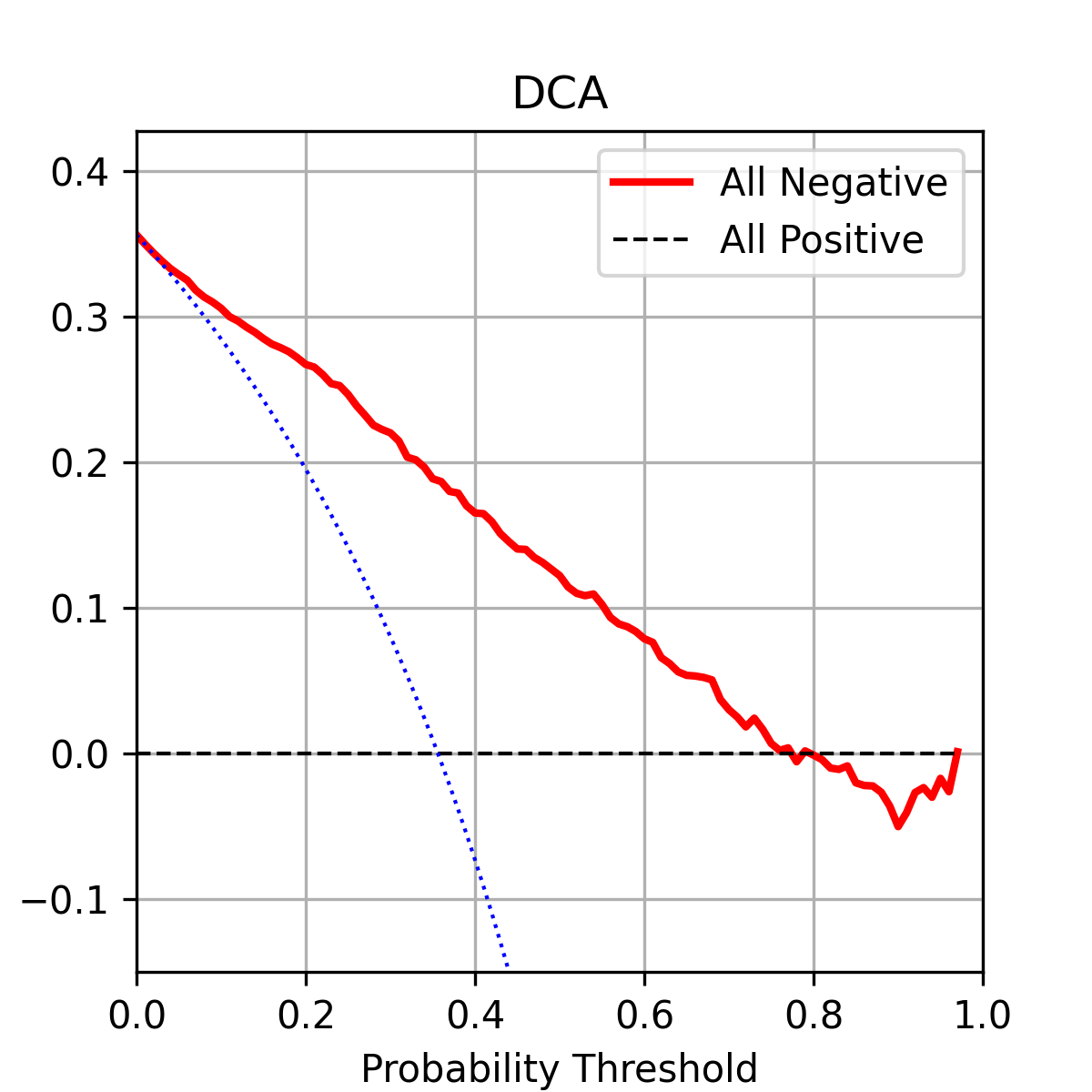

Supplement: Supplementary file 1 [file diagnostics-14-00053-s001.zip › Results of all classifiers/EmbeddingLR/LDA/Train Set/DCA.png]

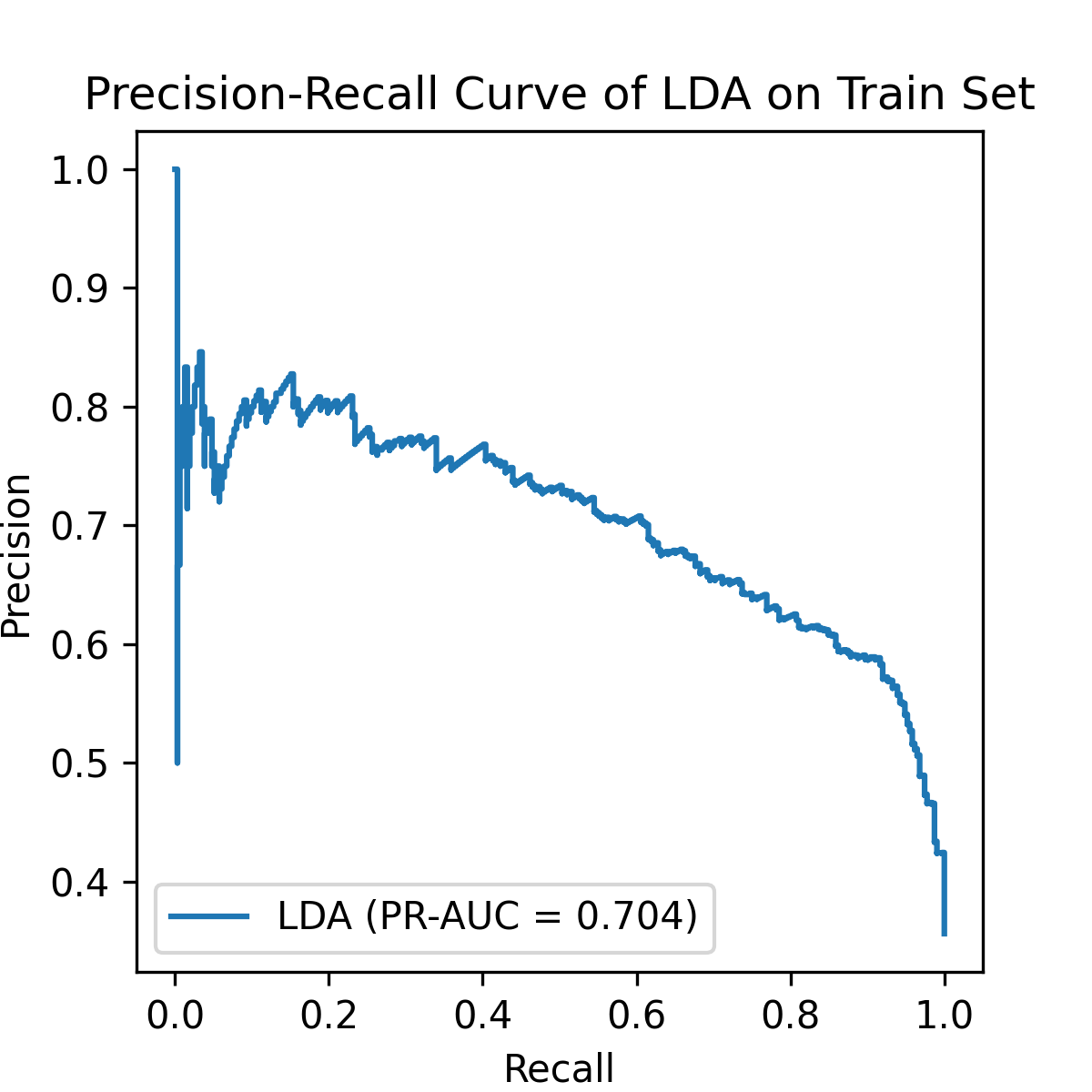

Supplement: Supplementary file 1 [file diagnostics-14-00053-s001.zip › Results of all classifiers/EmbeddingLR/LDA/Train Set/Precision-Recall Curve of LDA on Train Set.png]

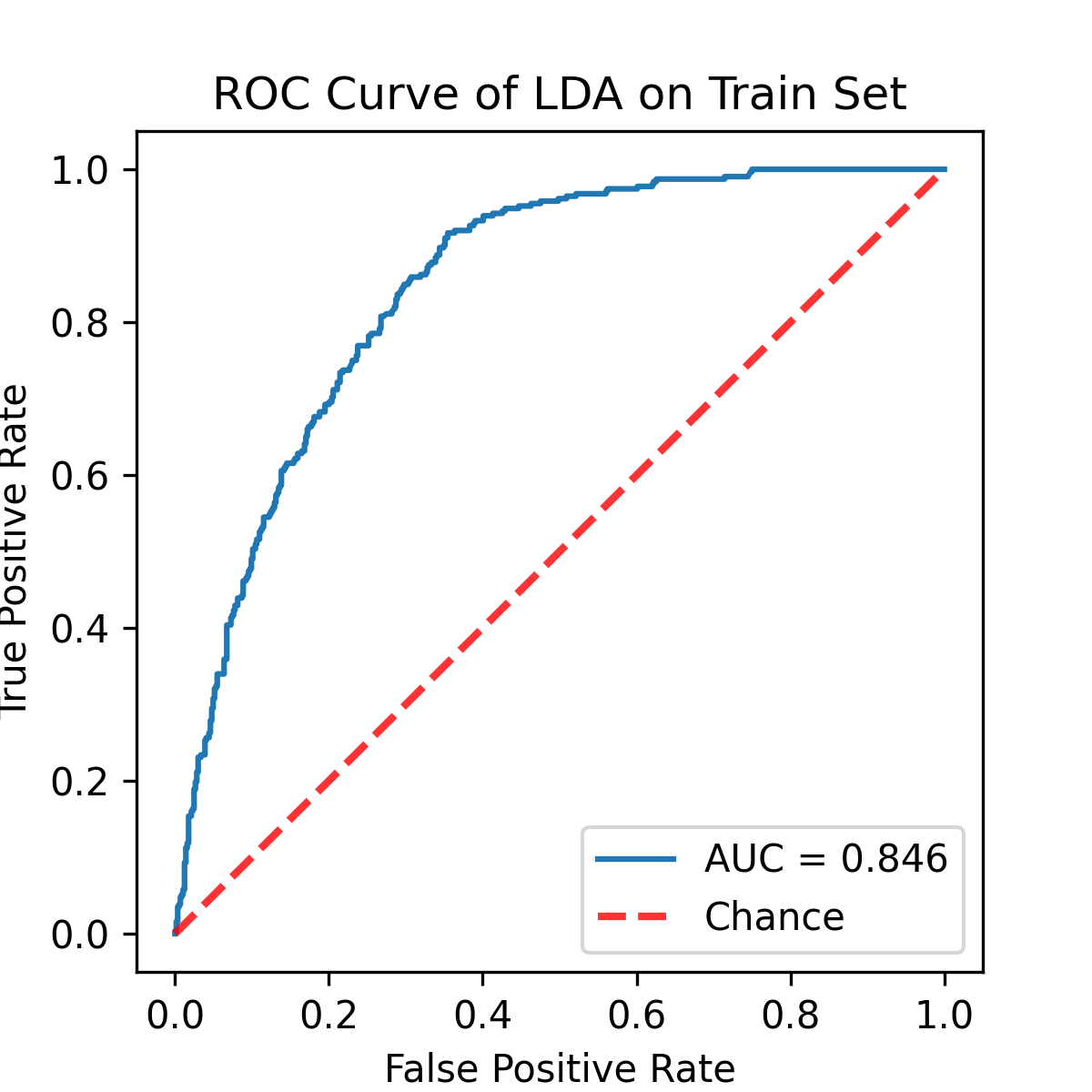

Supplement: Supplementary file 1 [file diagnostics-14-00053-s001.zip › Results of all classifiers/EmbeddingLR/LDA/Train Set/ROC Curve of LDA on Train Set.png]

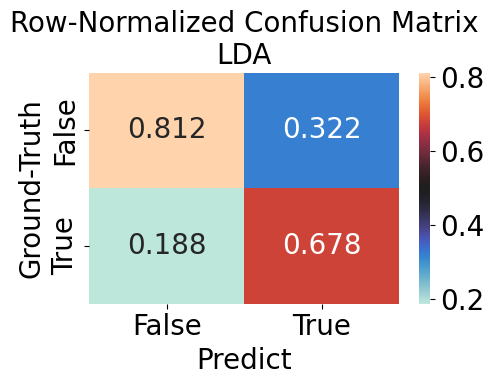

Supplement: Supplementary file 1 [file diagnostics-14-00053-s001.zip › Results of all classifiers/EmbeddingLR/LDA/Train Set/Row-Normalized Confusion Matrix LDA.png]

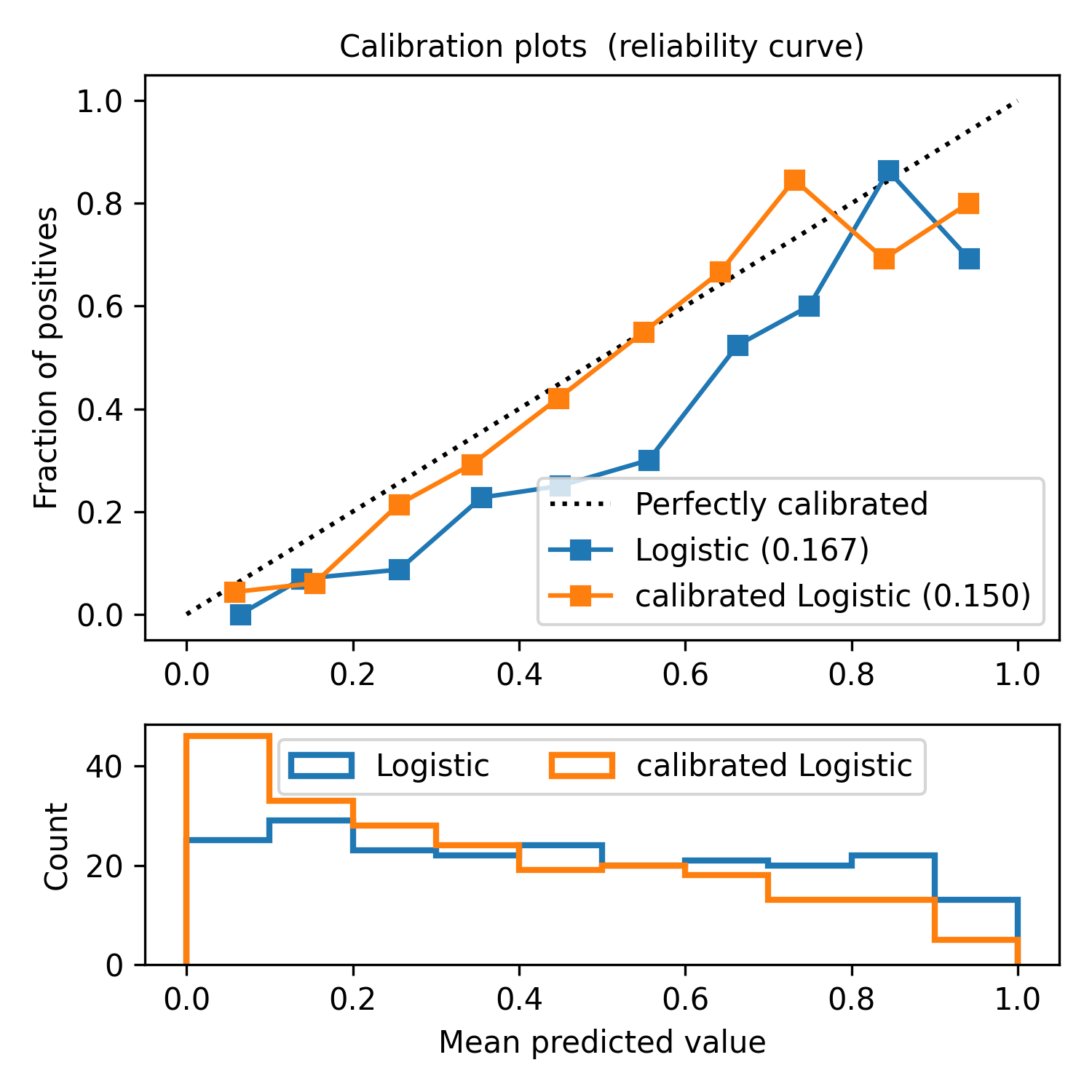

Supplement: Supplementary file 1 [file diagnostics-14-00053-s001.zip › Results of all classifiers/EmbeddingLR/Logistic/Test Set/Calibration plots.png]

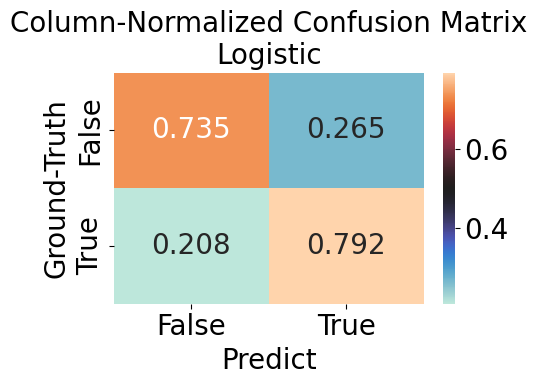

Supplement: Supplementary file 1 [file diagnostics-14-00053-s001.zip › Results of all classifiers/EmbeddingLR/Logistic/Test Set/Column-Normalized Confusion Matrix Logistic.png]

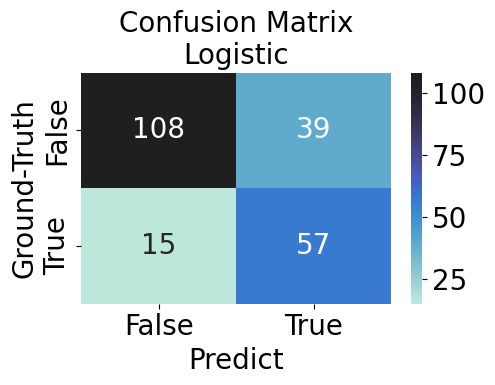

Supplement: Supplementary file 1 [file diagnostics-14-00053-s001.zip › Results of all classifiers/EmbeddingLR/Logistic/Test Set/Confusion Matrix Logistic.png]

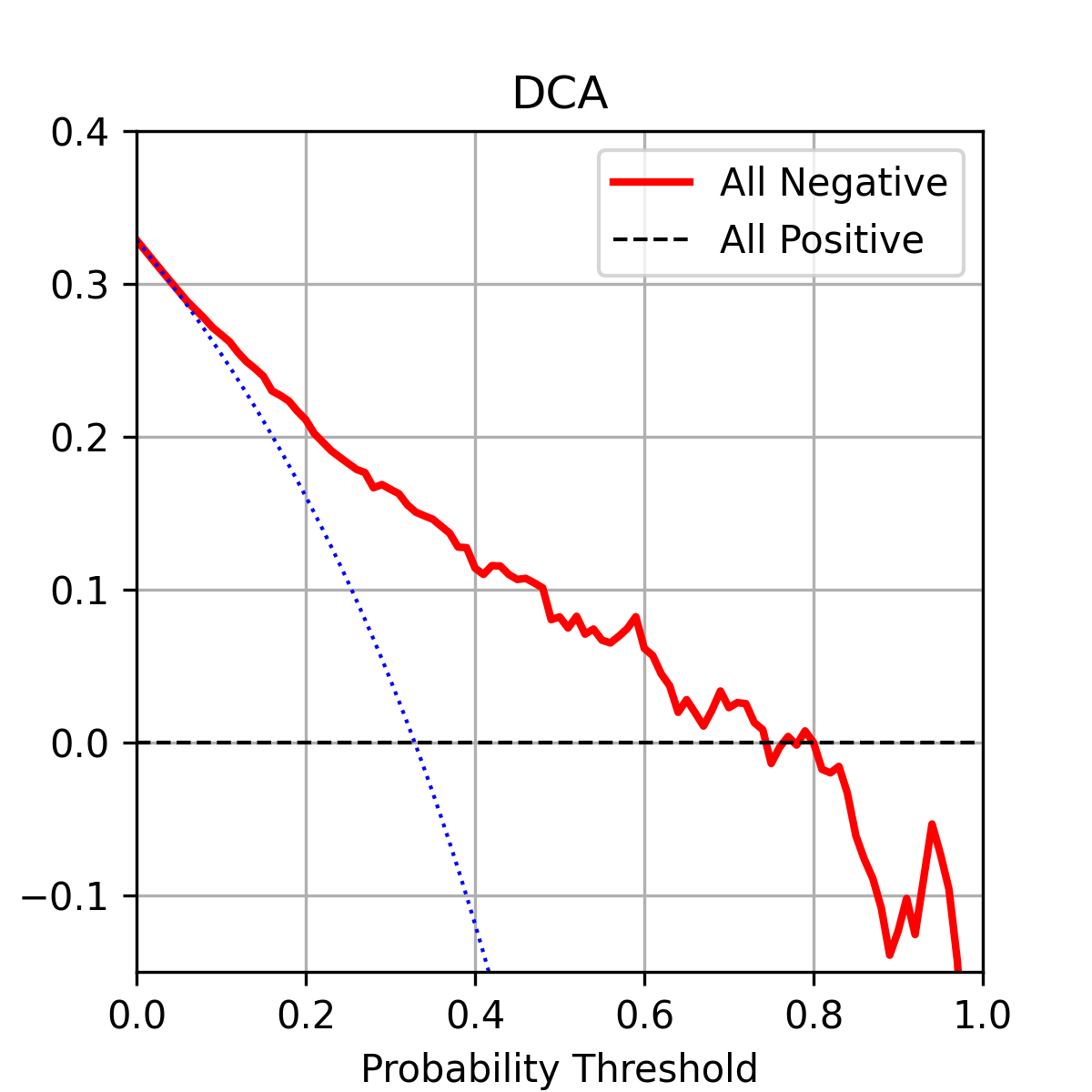

Supplement: Supplementary file 1 [file diagnostics-14-00053-s001.zip › Results of all classifiers/EmbeddingLR/Logistic/Test Set/DCA.png]

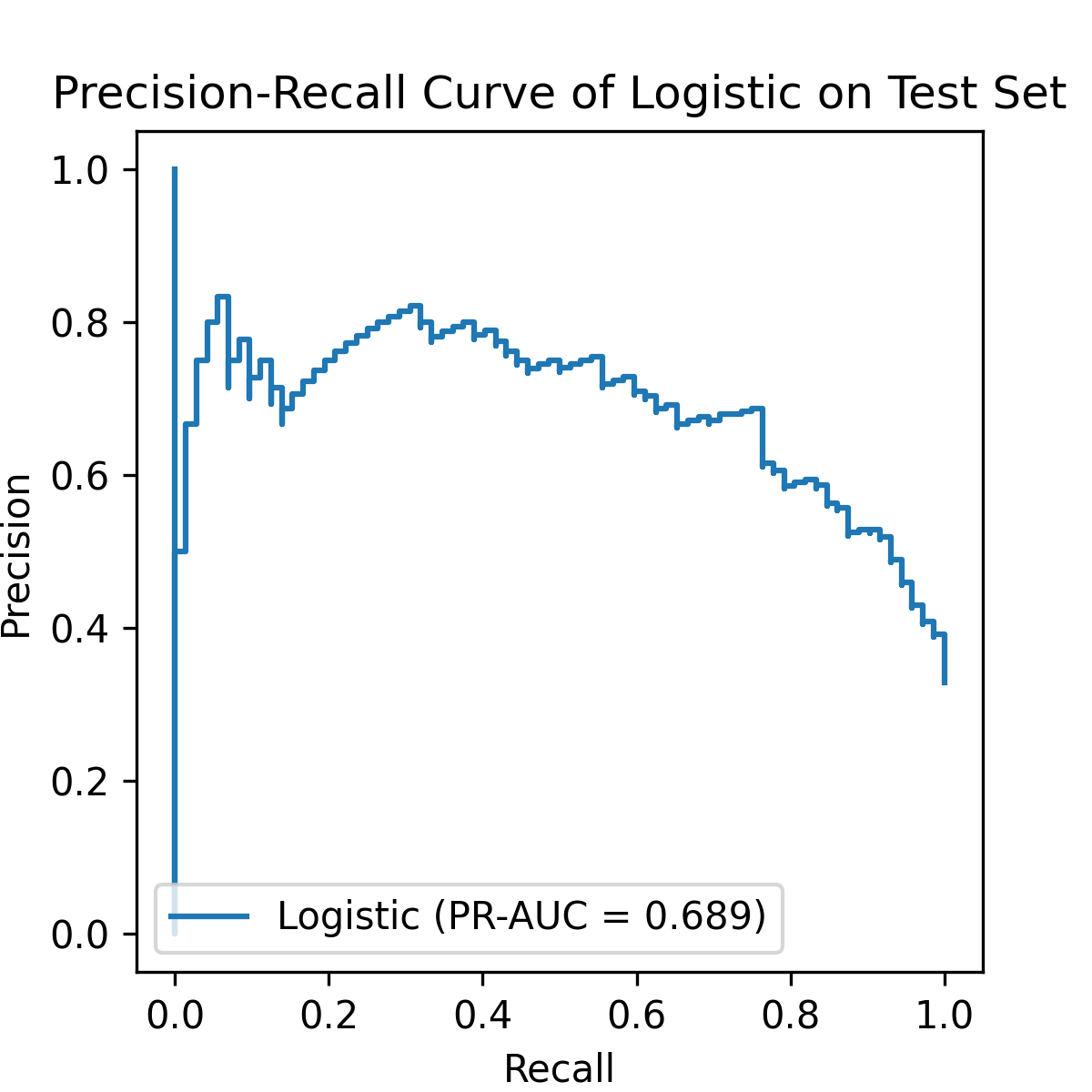

Supplement: Supplementary file 1 [file diagnostics-14-00053-s001.zip › Results of all classifiers/EmbeddingLR/Logistic/Test Set/Precision-Recall Curve of Logistic on Test Set.png]

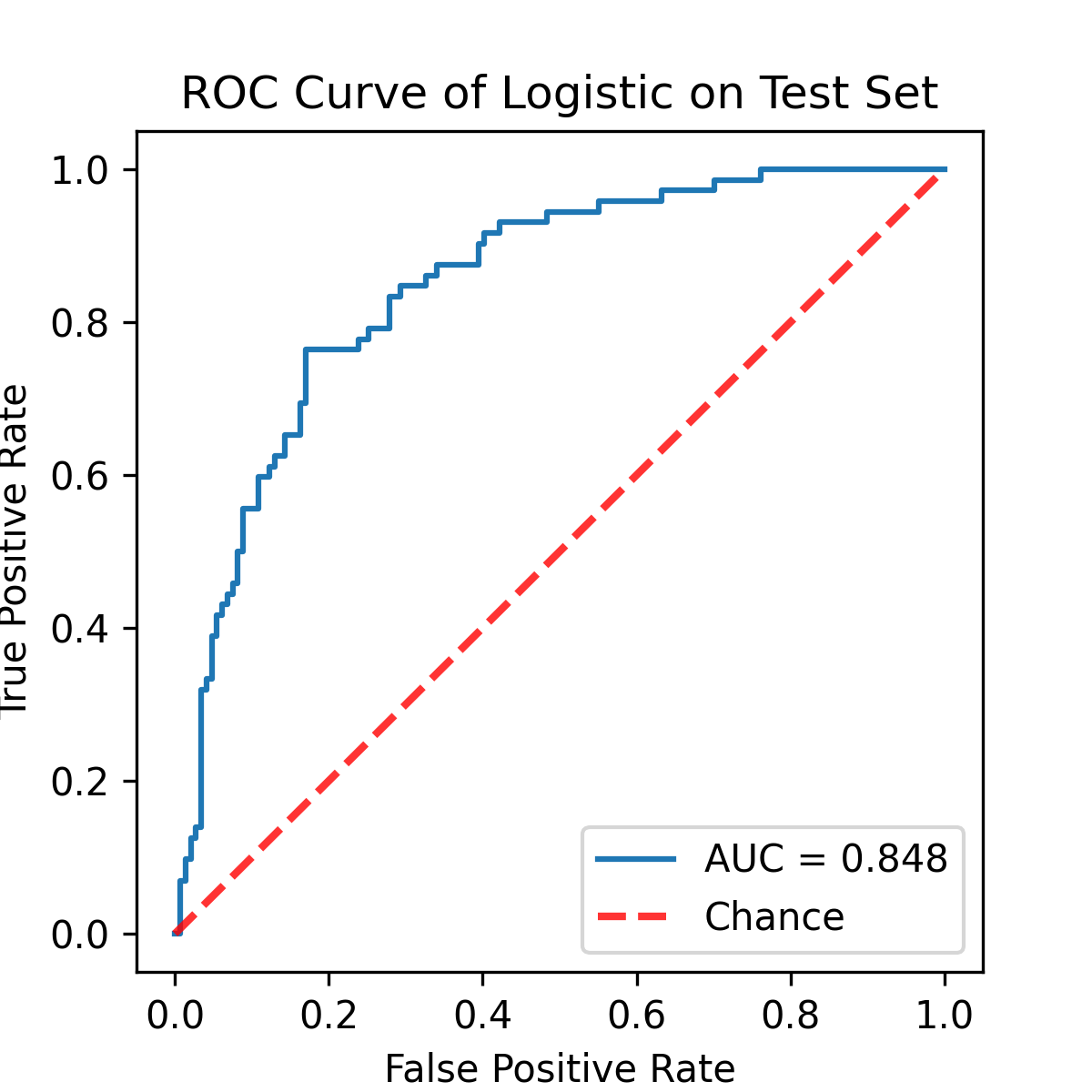

Supplement: Supplementary file 1 [file diagnostics-14-00053-s001.zip › Results of all classifiers/EmbeddingLR/Logistic/Test Set/ROC Curve of Logistic on Test Set.png]

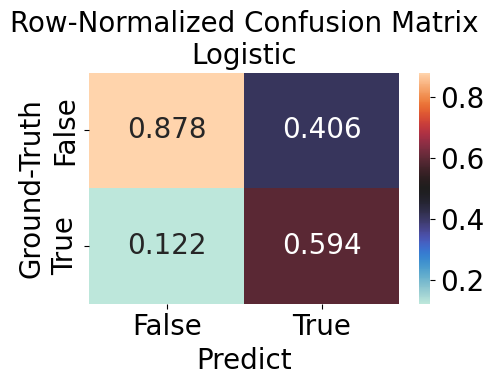

Supplement: Supplementary file 1 [file diagnostics-14-00053-s001.zip › Results of all classifiers/EmbeddingLR/Logistic/Test Set/Row-Normalized Confusion Matrix Logistic.png]

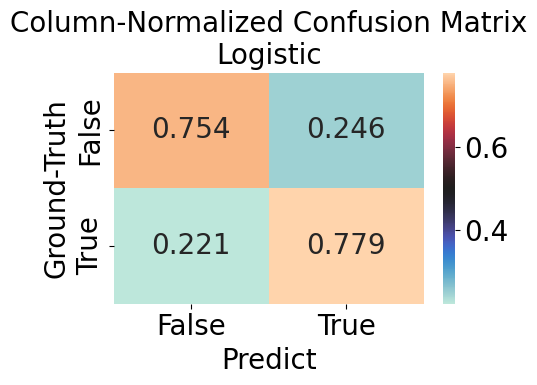

Supplement: Supplementary file 1 [file diagnostics-14-00053-s001.zip › Results of all classifiers/EmbeddingLR/Logistic/Train Set/Column-Normalized Confusion Matrix Logistic.png]

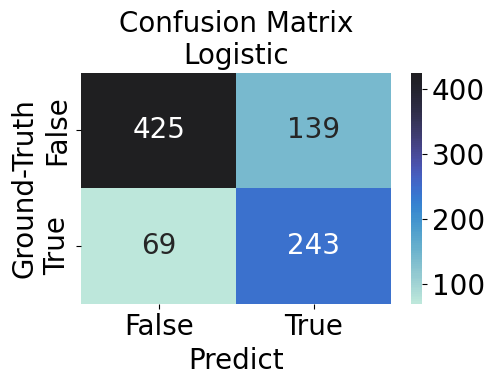

Supplement: Supplementary file 1 [file diagnostics-14-00053-s001.zip › Results of all classifiers/EmbeddingLR/Logistic/Train Set/Confusion Matrix Logistic.png]

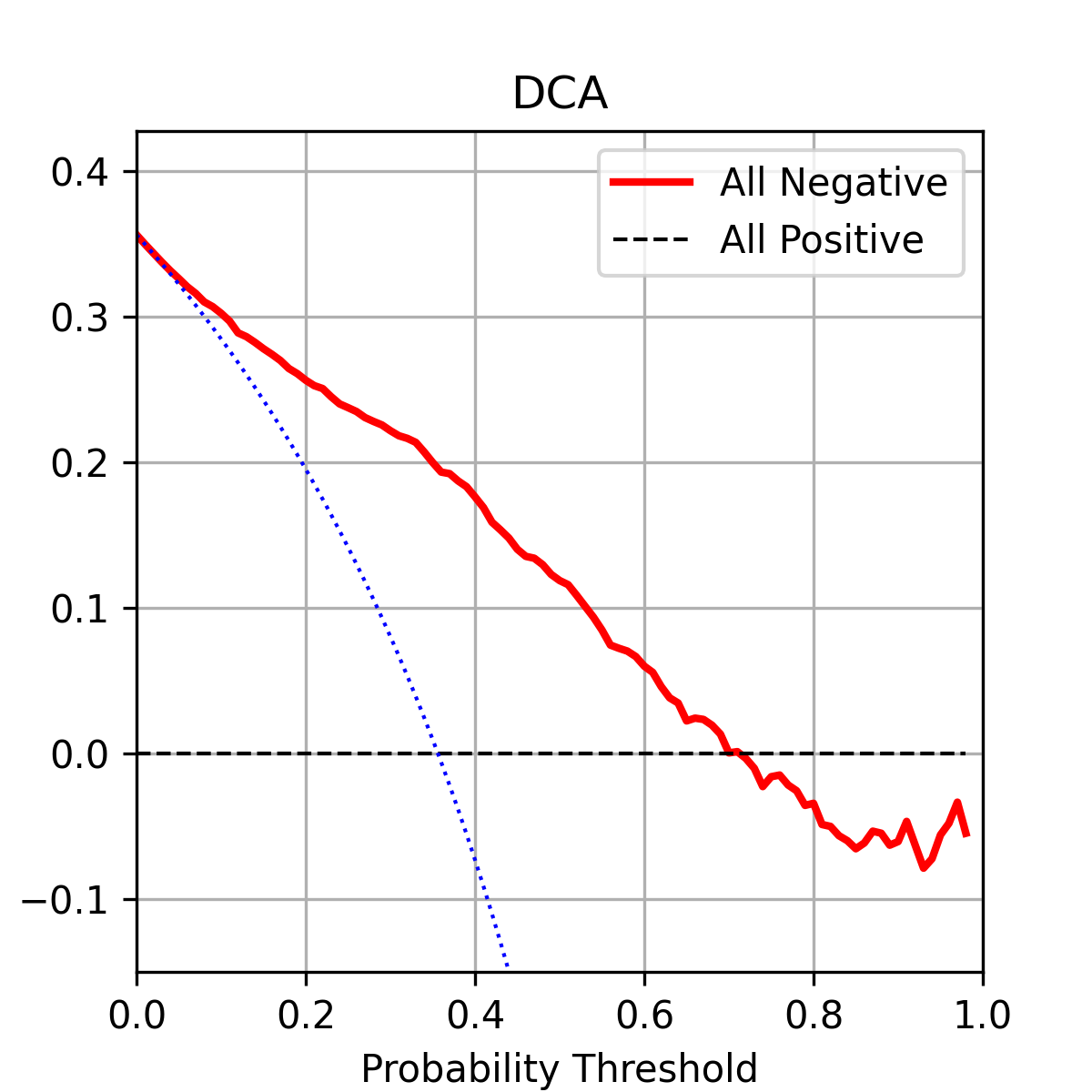

Supplement: Supplementary file 1 [file diagnostics-14-00053-s001.zip › Results of all classifiers/EmbeddingLR/Logistic/Train Set/DCA.png]

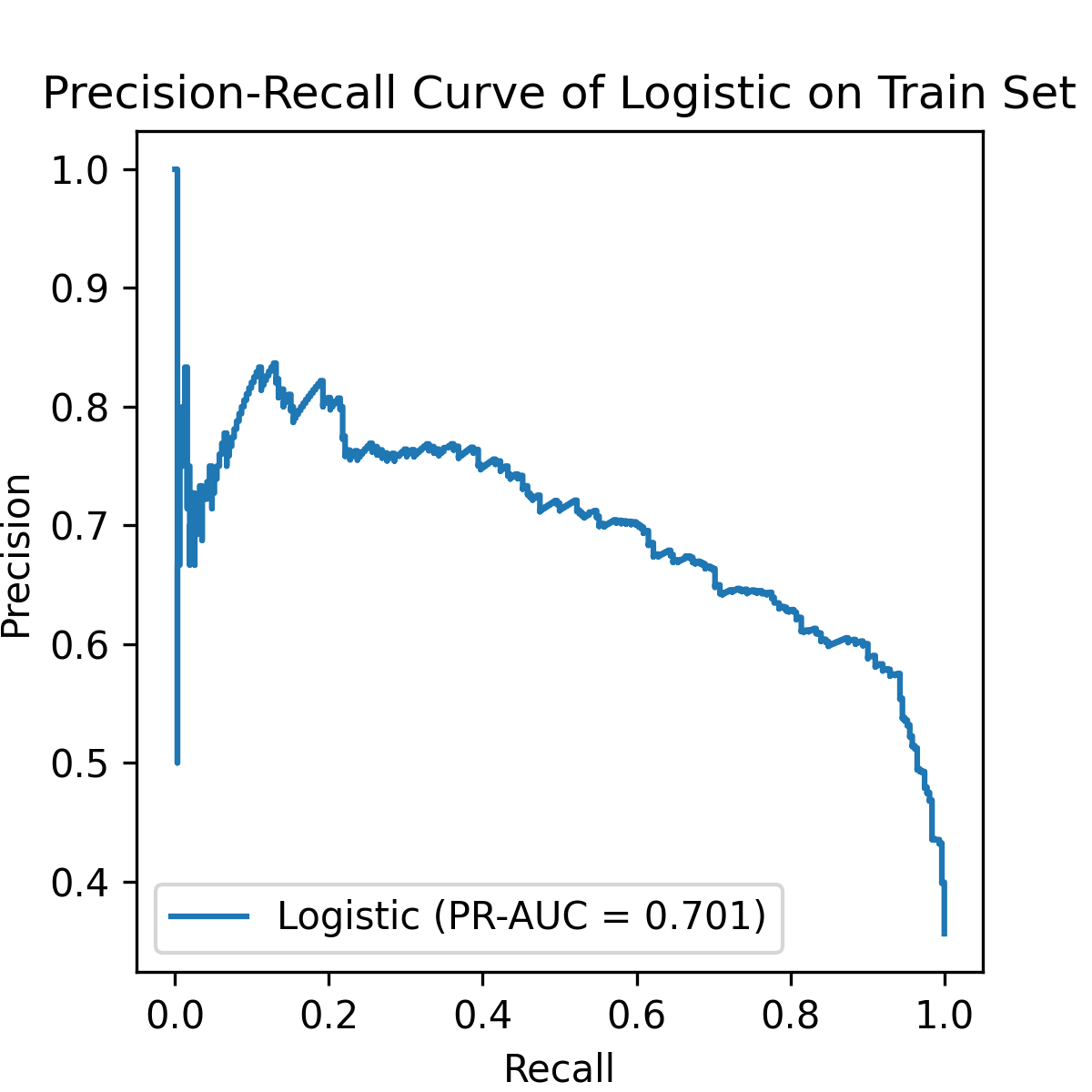

Supplement: Supplementary file 1 [file diagnostics-14-00053-s001.zip › Results of all classifiers/EmbeddingLR/Logistic/Train Set/Precision-Recall Curve of Logistic on Train Set.png]

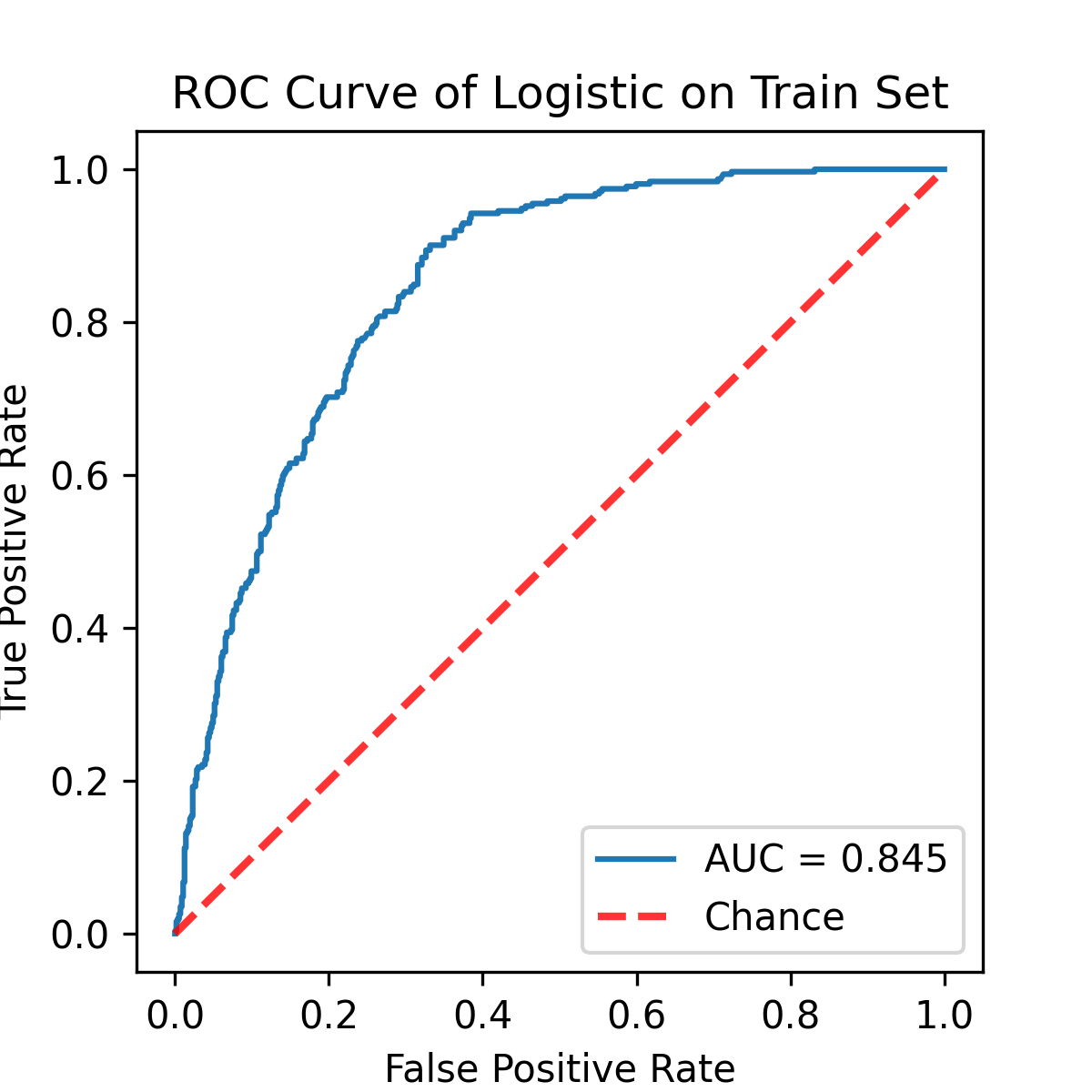

Supplement: Supplementary file 1 [file diagnostics-14-00053-s001.zip › Results of all classifiers/EmbeddingLR/Logistic/Train Set/ROC Curve of Logistic on Train Set.png]

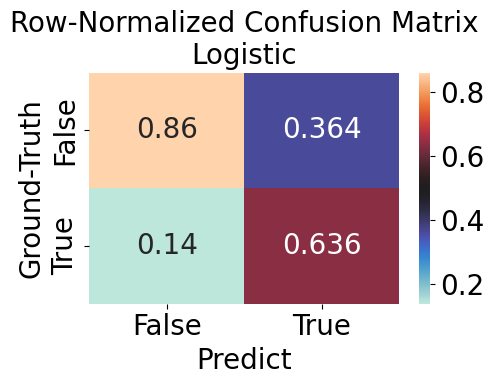

Supplement: Supplementary file 1 [file diagnostics-14-00053-s001.zip › Results of all classifiers/EmbeddingLR/Logistic/Train Set/Row-Normalized Confusion Matrix Logistic.png]

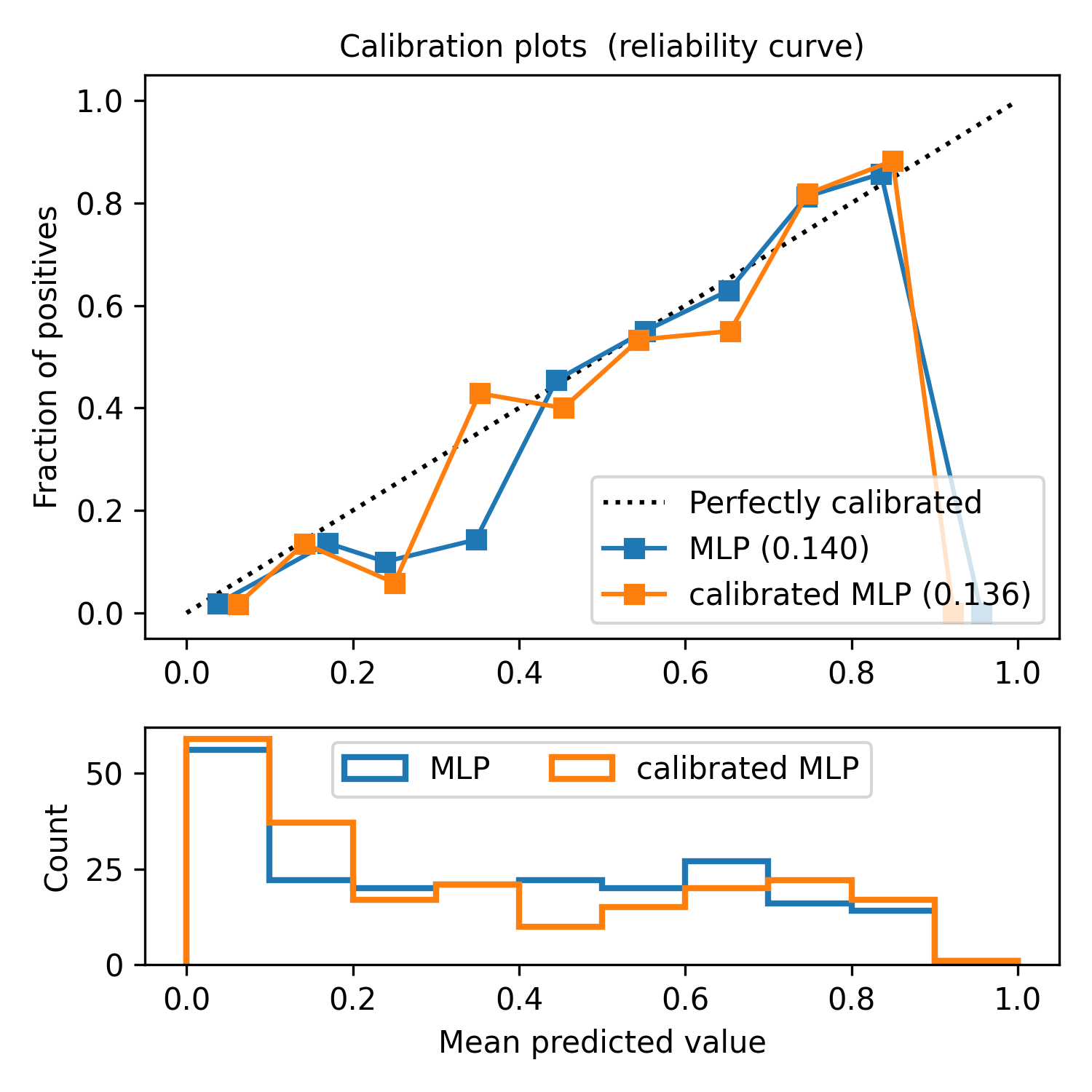

Supplement: Supplementary file 1 [file diagnostics-14-00053-s001.zip › Results of all classifiers/EmbeddingLR/MLP/Test Set/Calibration plots.png]

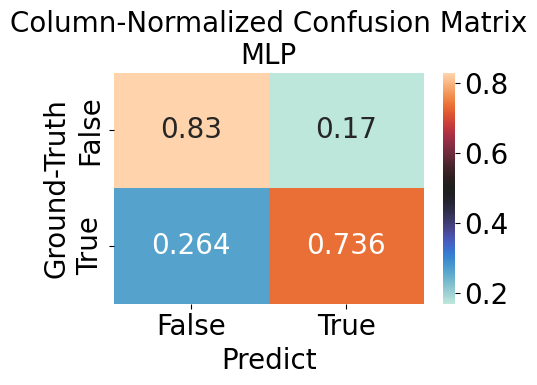

Supplement: Supplementary file 1 [file diagnostics-14-00053-s001.zip › Results of all classifiers/EmbeddingLR/MLP/Test Set/Column-Normalized Confusion Matrix MLP.png]

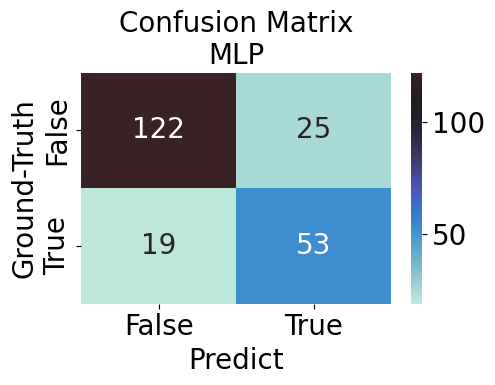

Supplement: Supplementary file 1 [file diagnostics-14-00053-s001.zip › Results of all classifiers/EmbeddingLR/MLP/Test Set/Confusion Matrix MLP.png]

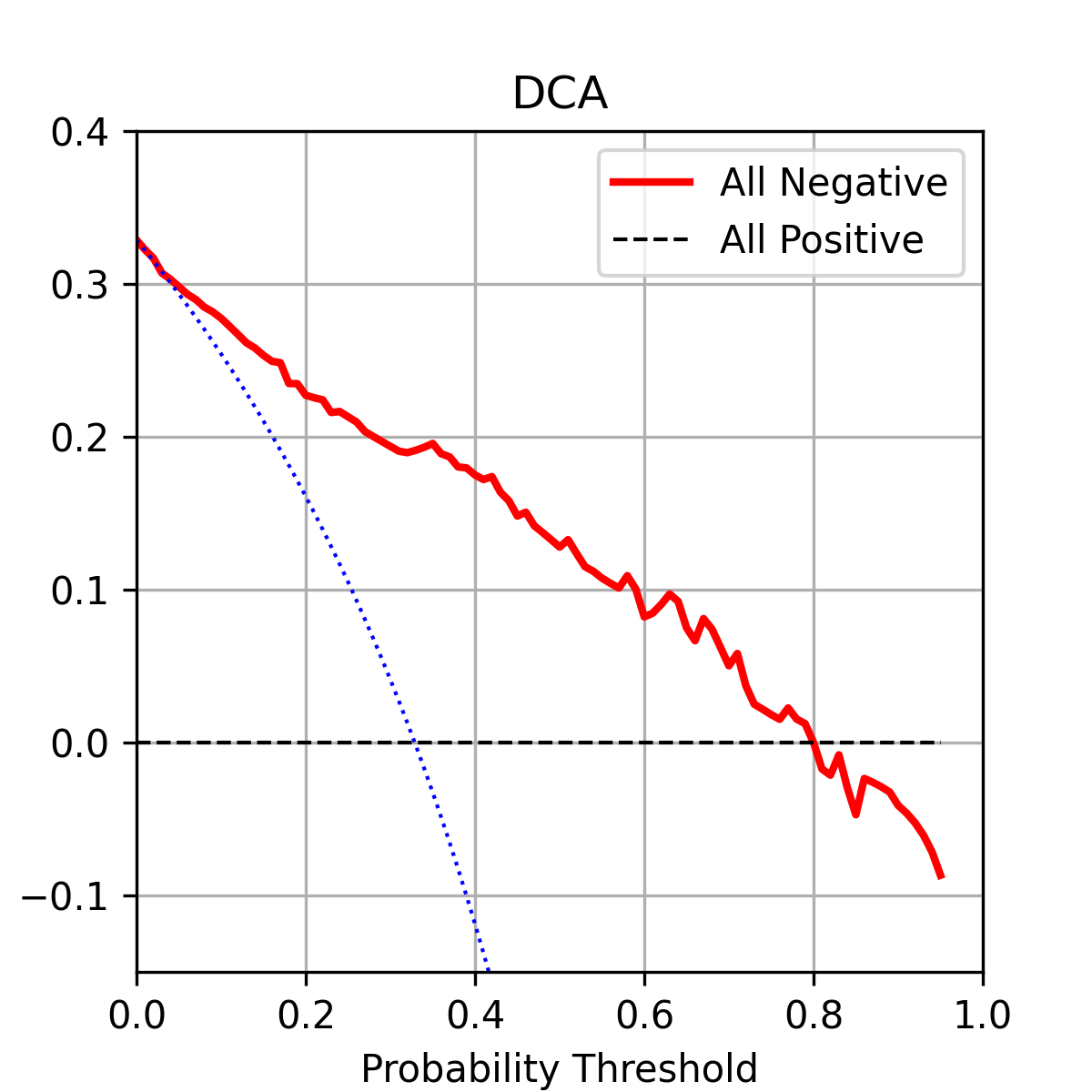

Supplement: Supplementary file 1 [file diagnostics-14-00053-s001.zip › Results of all classifiers/EmbeddingLR/MLP/Test Set/DCA.png]

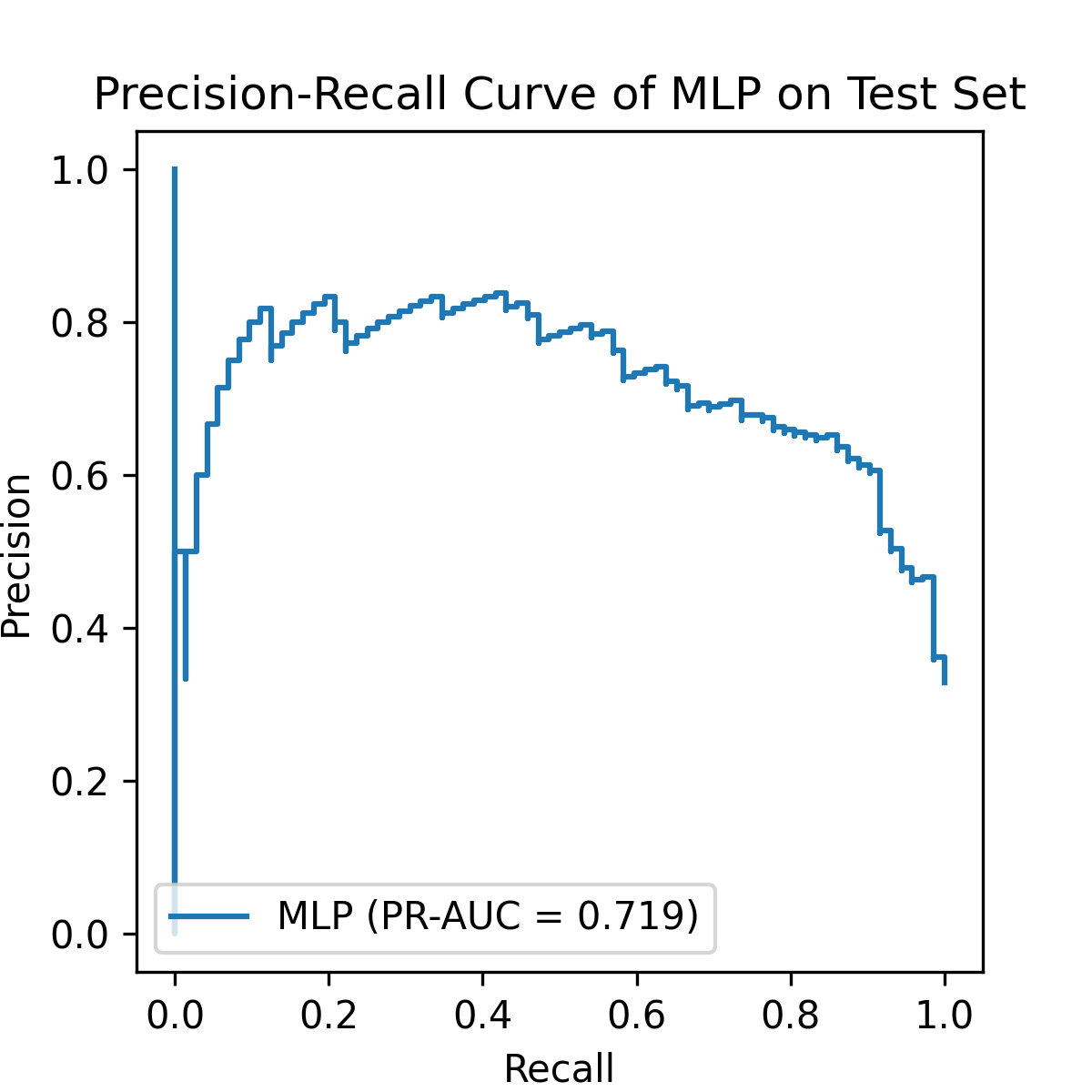

Supplement: Supplementary file 1 [file diagnostics-14-00053-s001.zip › Results of all classifiers/EmbeddingLR/MLP/Test Set/Precision-Recall Curve of MLP on Test Set.png]

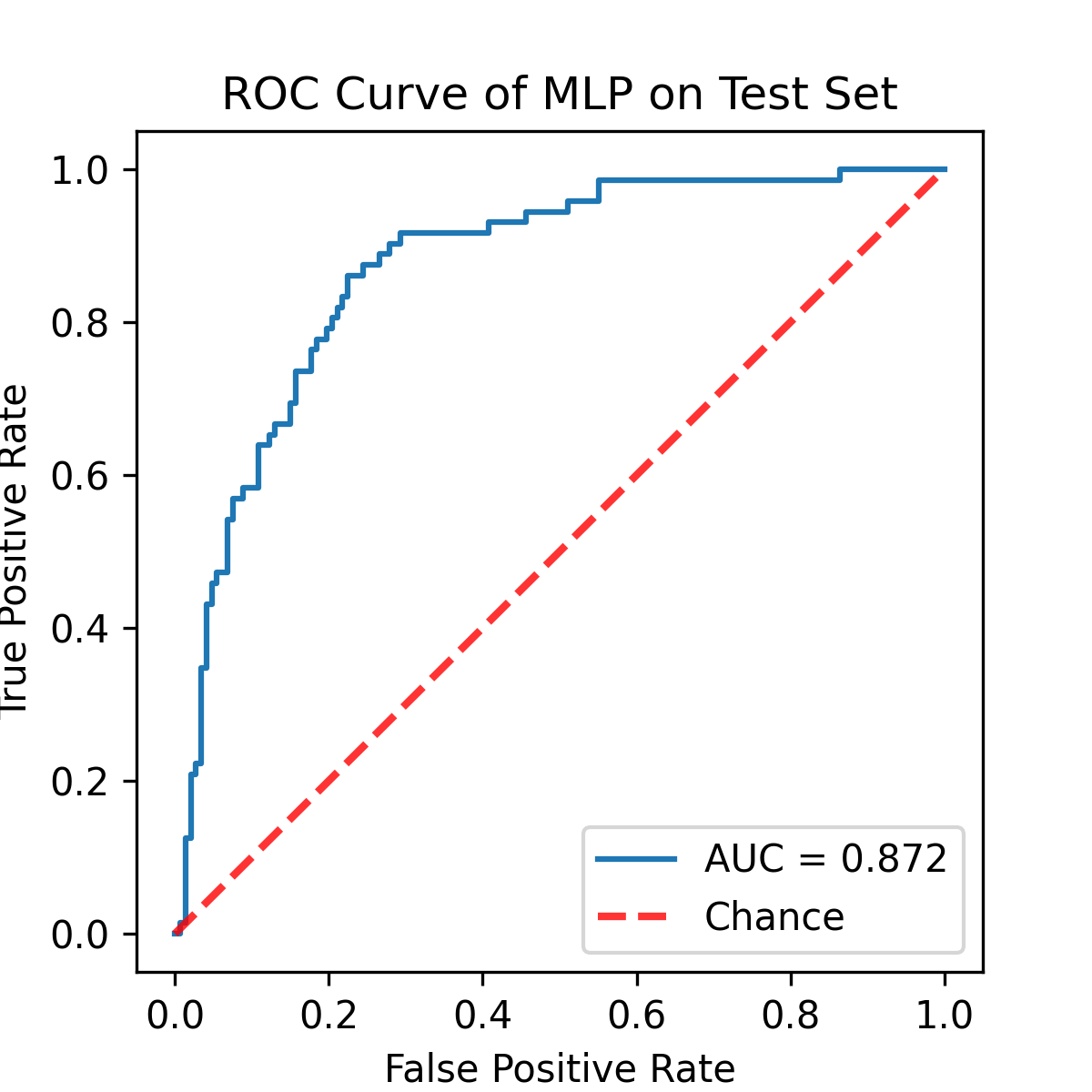

Supplement: Supplementary file 1 [file diagnostics-14-00053-s001.zip › Results of all classifiers/EmbeddingLR/MLP/Test Set/ROC Curve of MLP on Test Set.png]

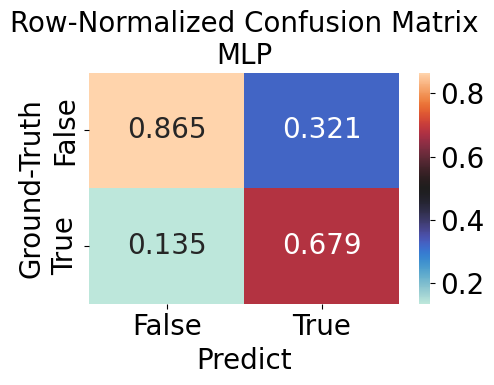

Supplement: Supplementary file 1 [file diagnostics-14-00053-s001.zip › Results of all classifiers/EmbeddingLR/MLP/Test Set/Row-Normalized Confusion Matrix MLP.png]

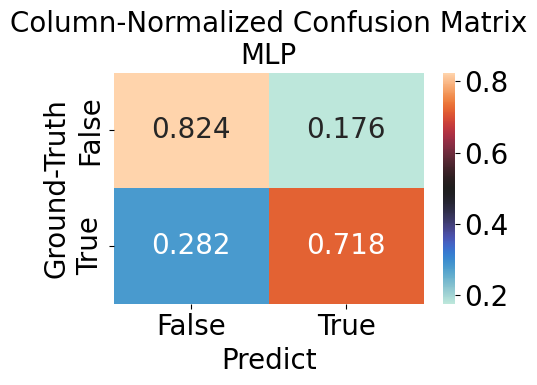

Supplement: Supplementary file 1 [file diagnostics-14-00053-s001.zip › Results of all classifiers/EmbeddingLR/MLP/Train Set/Column-Normalized Confusion Matrix MLP.png]

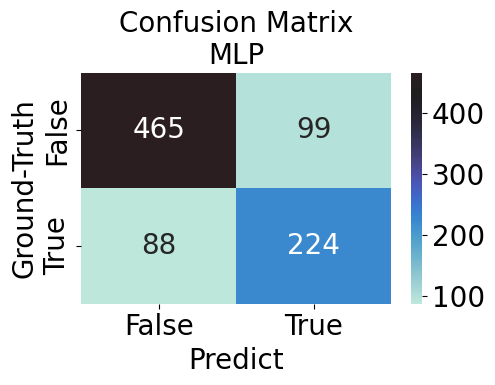

Supplement: Supplementary file 1 [file diagnostics-14-00053-s001.zip › Results of all classifiers/EmbeddingLR/MLP/Train Set/Confusion Matrix MLP.png]

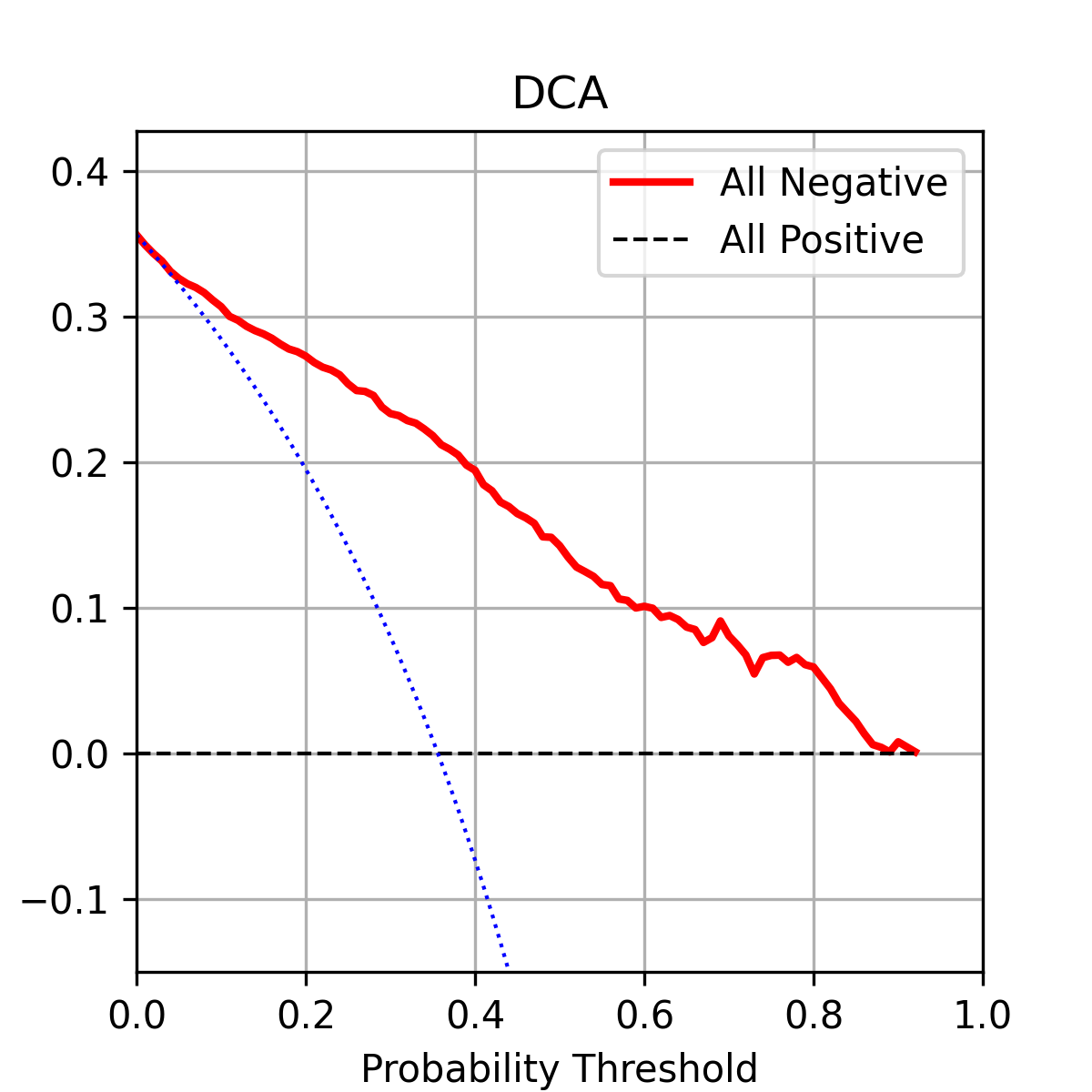

Supplement: Supplementary file 1 [file diagnostics-14-00053-s001.zip › Results of all classifiers/EmbeddingLR/MLP/Train Set/DCA.png]

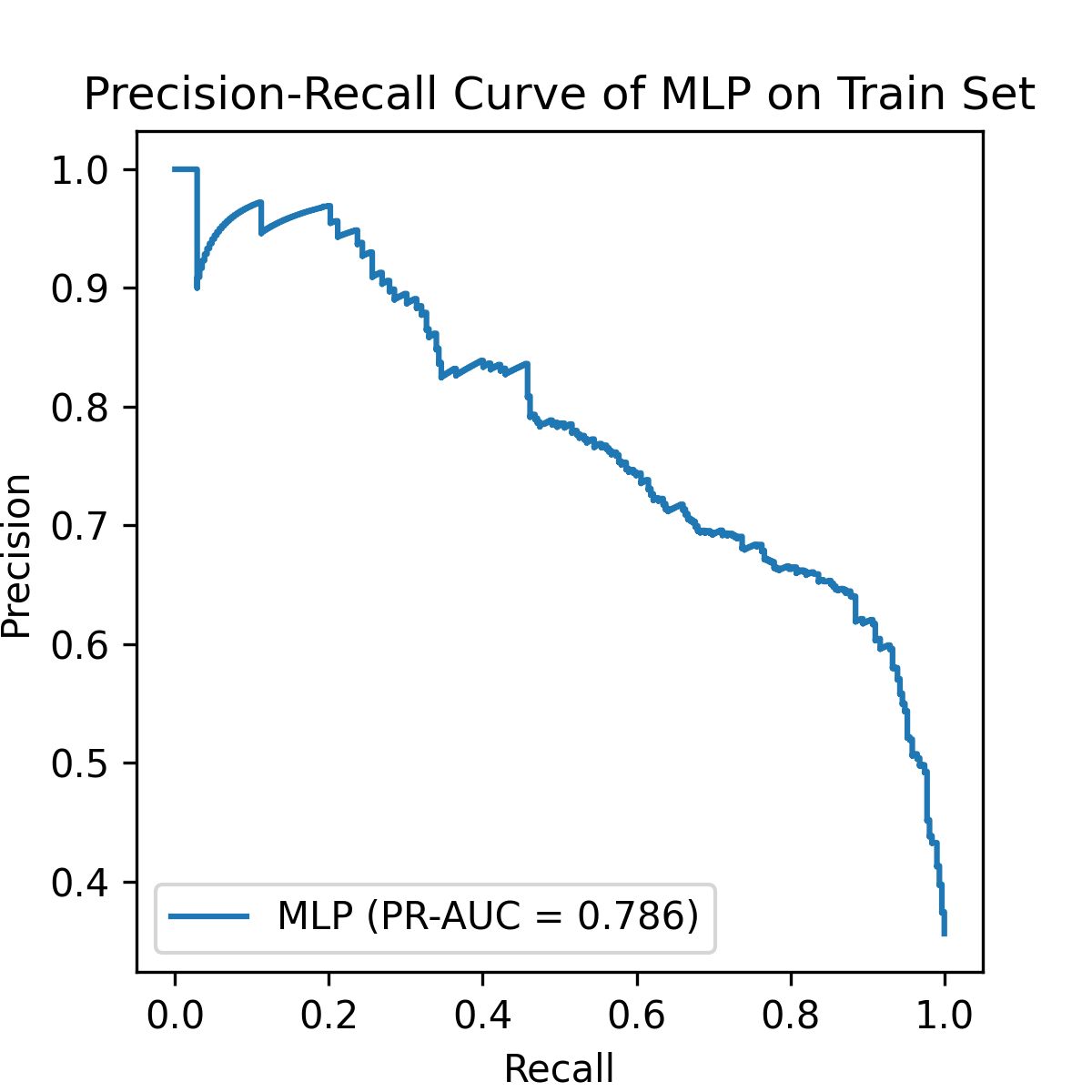

Supplement: Supplementary file 1 [file diagnostics-14-00053-s001.zip › Results of all classifiers/EmbeddingLR/MLP/Train Set/Precision-Recall Curve of MLP on Train Set.png]

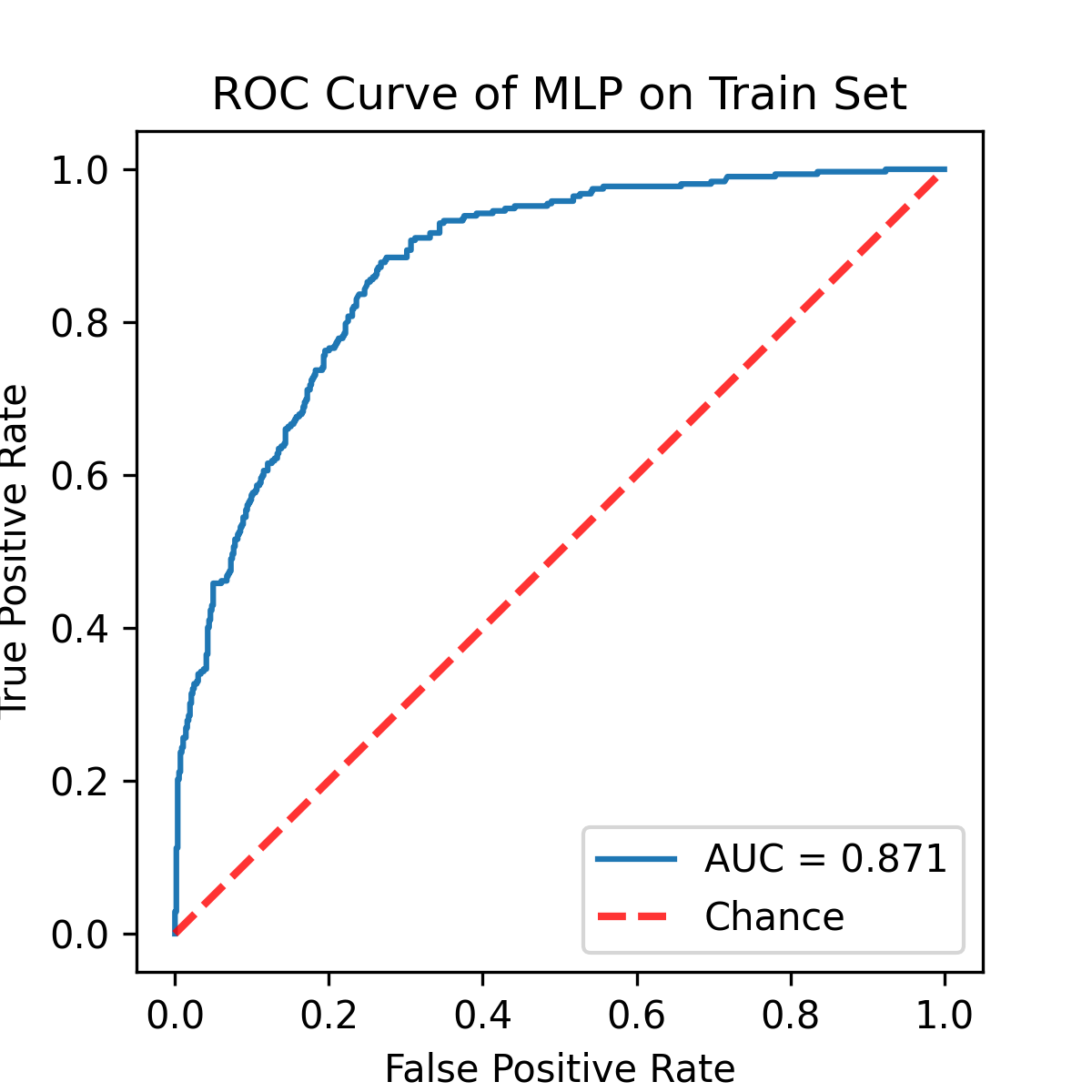

Supplement: Supplementary file 1 [file diagnostics-14-00053-s001.zip › Results of all classifiers/EmbeddingLR/MLP/Train Set/ROC Curve of MLP on Train Set.png]

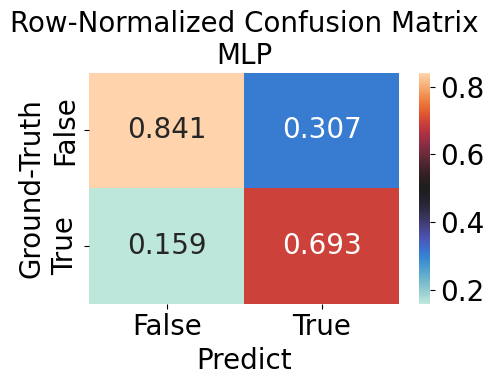

Supplement: Supplementary file 1 [file diagnostics-14-00053-s001.zip › Results of all classifiers/EmbeddingLR/MLP/Train Set/Row-Normalized Confusion Matrix MLP.png]

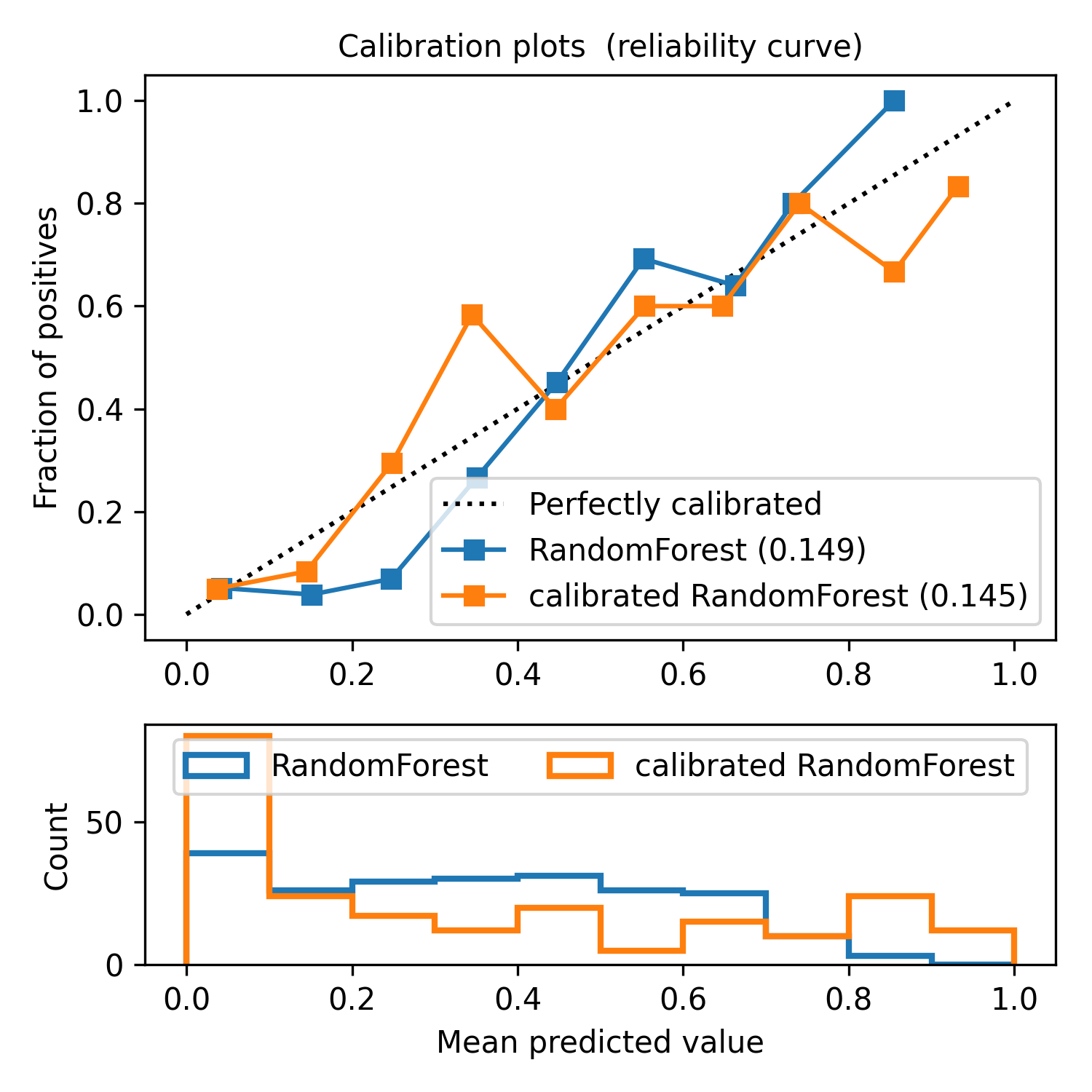

Supplement: Supplementary file 1 [file diagnostics-14-00053-s001.zip › Results of all classifiers/EmbeddingLR/RandomForest/Test Set/Calibration plots.png]

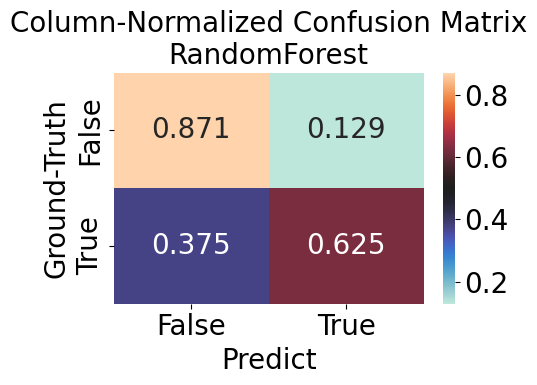

Supplement: Supplementary file 1 [file diagnostics-14-00053-s001.zip › Results of all classifiers/EmbeddingLR/RandomForest/Test Set/Column-Normalized Confusion Matrix RandomForest.png]

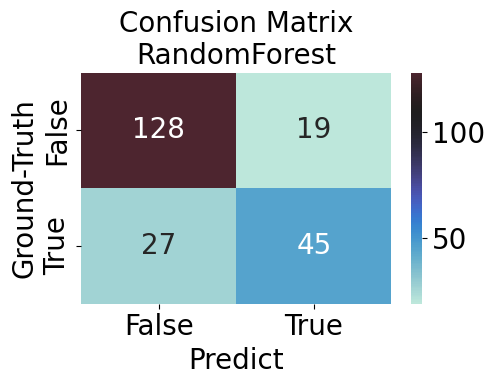

Supplement: Supplementary file 1 [file diagnostics-14-00053-s001.zip › Results of all classifiers/EmbeddingLR/RandomForest/Test Set/Confusion Matrix RandomForest.png]

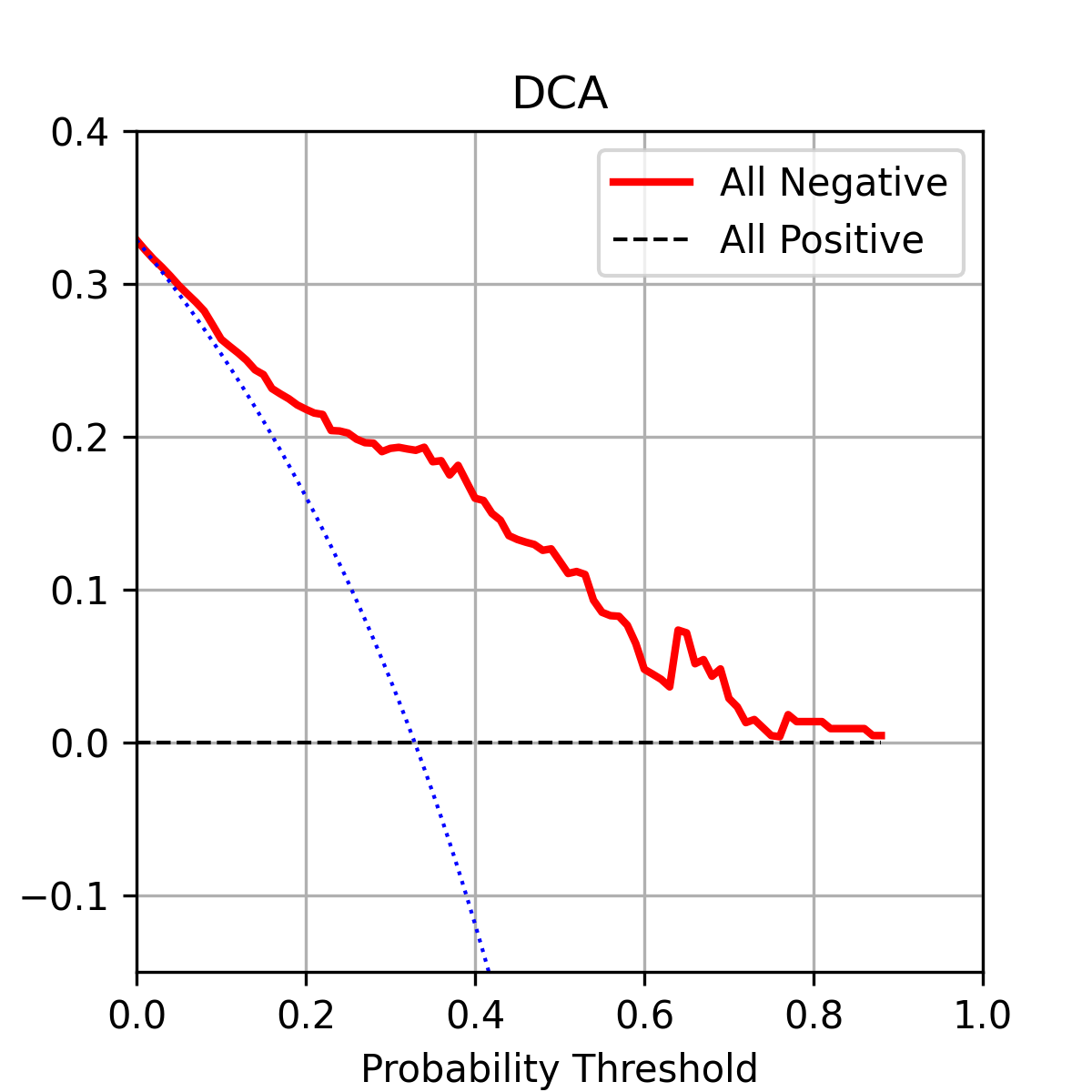

Supplement: Supplementary file 1 [file diagnostics-14-00053-s001.zip › Results of all classifiers/EmbeddingLR/RandomForest/Test Set/DCA.png]

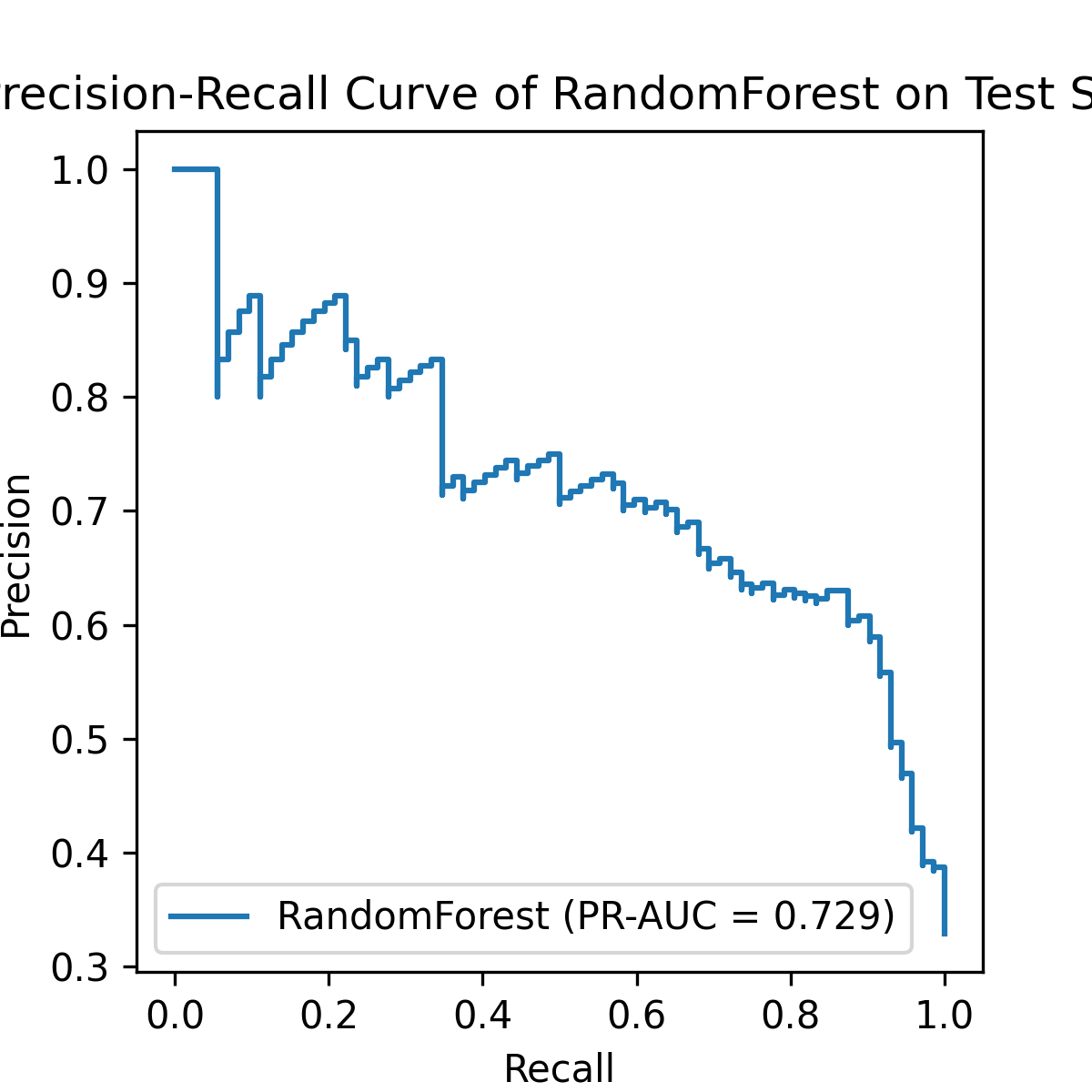

Supplement: Supplementary file 1 [file diagnostics-14-00053-s001.zip › Results of all classifiers/EmbeddingLR/RandomForest/Test Set/Precision-Recall Curve of RandomForest on Test Set.png]

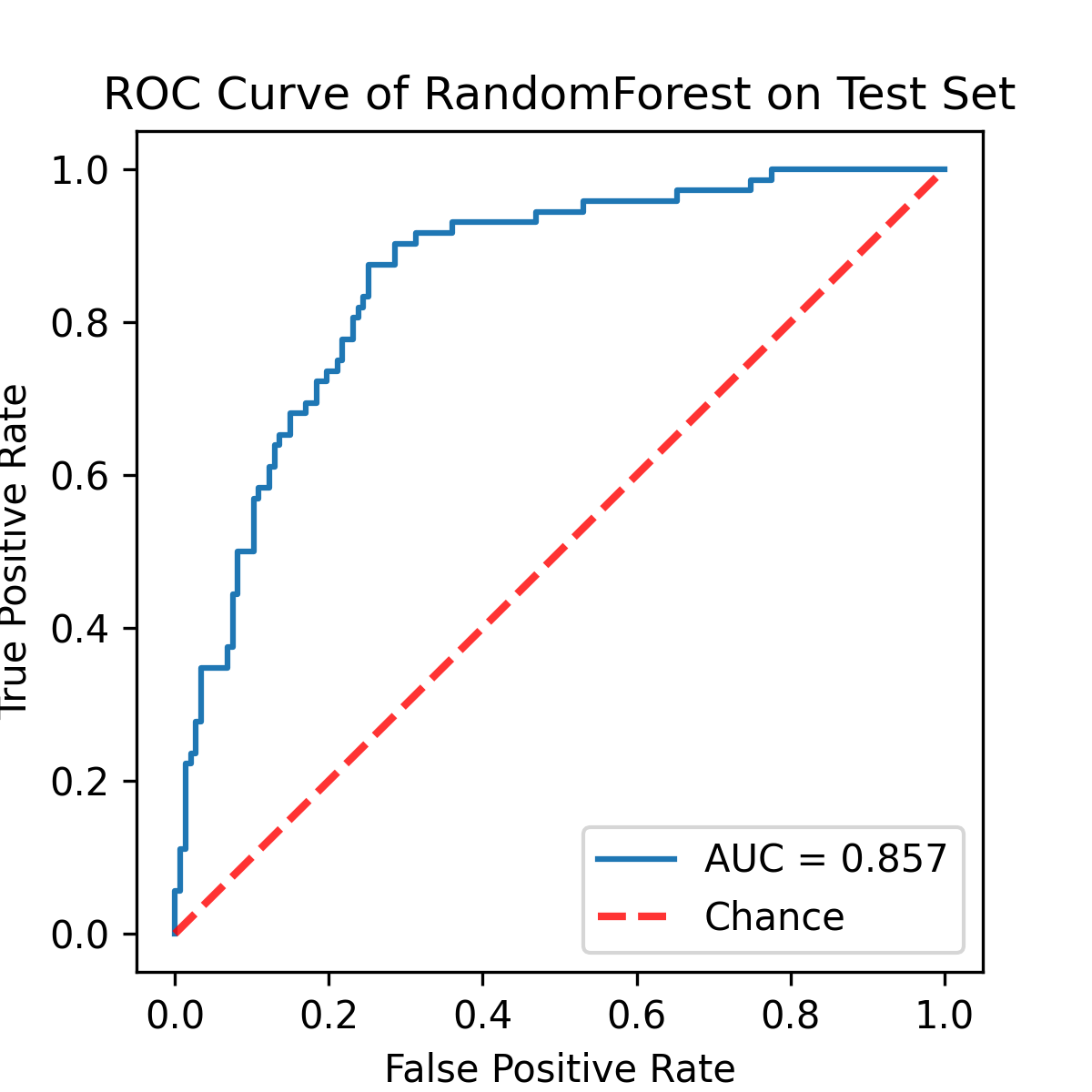

Supplement: Supplementary file 1 [file diagnostics-14-00053-s001.zip › Results of all classifiers/EmbeddingLR/RandomForest/Test Set/ROC Curve of RandomForest on Test Set.png]

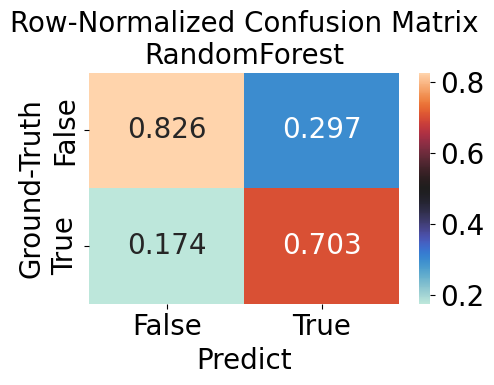

Supplement: Supplementary file 1 [file diagnostics-14-00053-s001.zip › Results of all classifiers/EmbeddingLR/RandomForest/Test Set/Row-Normalized Confusion Matrix RandomForest.png]

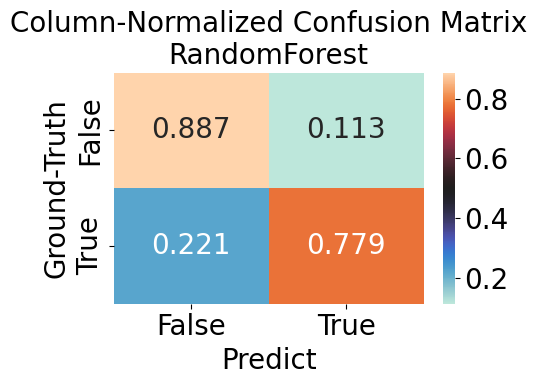

Supplement: Supplementary file 1 [file diagnostics-14-00053-s001.zip › Results of all classifiers/EmbeddingLR/RandomForest/Train Set/Column-Normalized Confusion Matrix RandomForest.png]

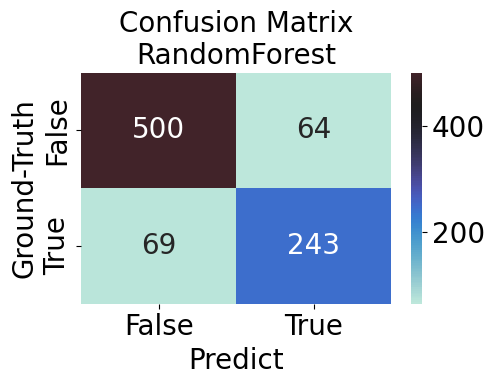

Supplement: Supplementary file 1 [file diagnostics-14-00053-s001.zip › Results of all classifiers/EmbeddingLR/RandomForest/Train Set/Confusion Matrix RandomForest.png]

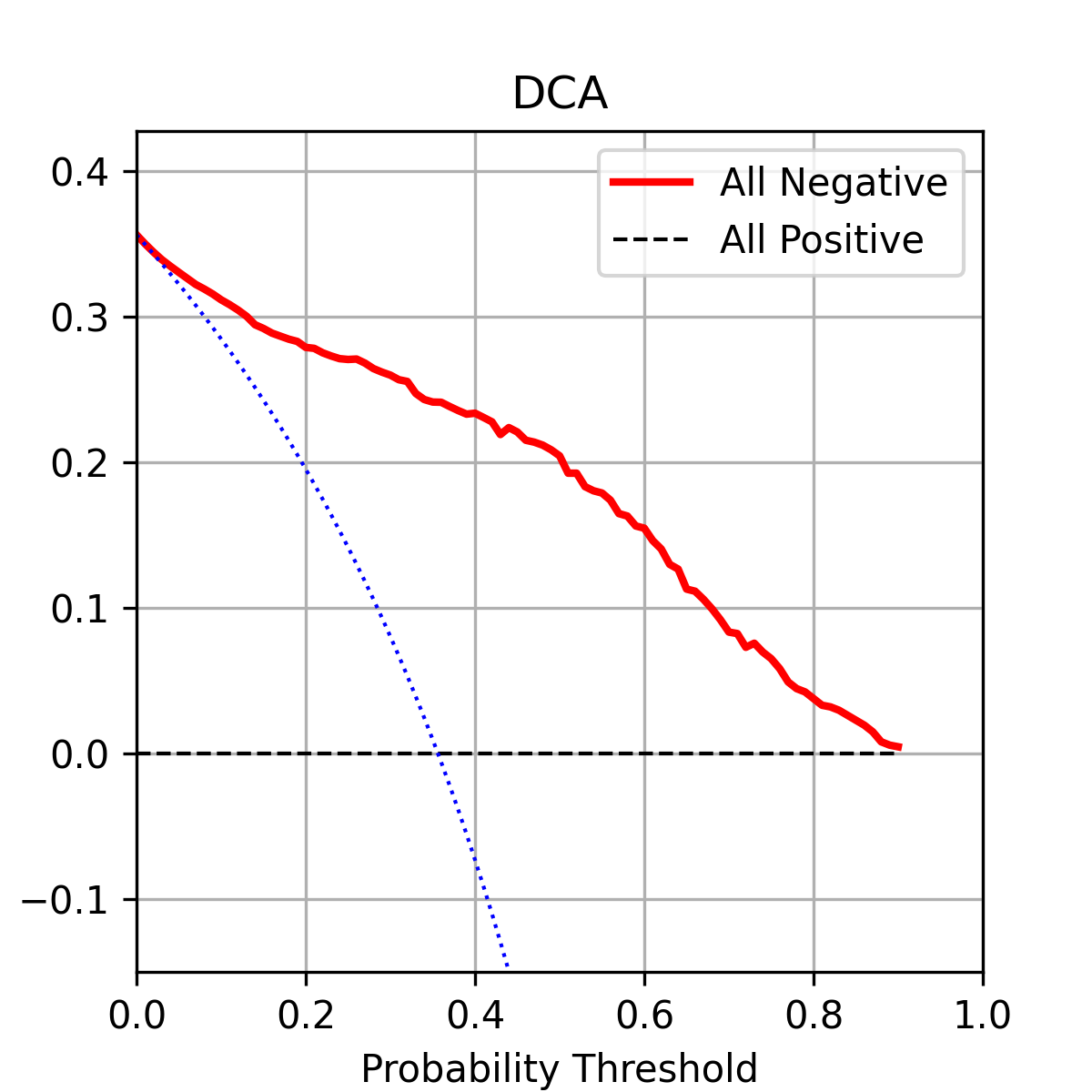

Supplement: Supplementary file 1 [file diagnostics-14-00053-s001.zip › Results of all classifiers/EmbeddingLR/RandomForest/Train Set/DCA.png]

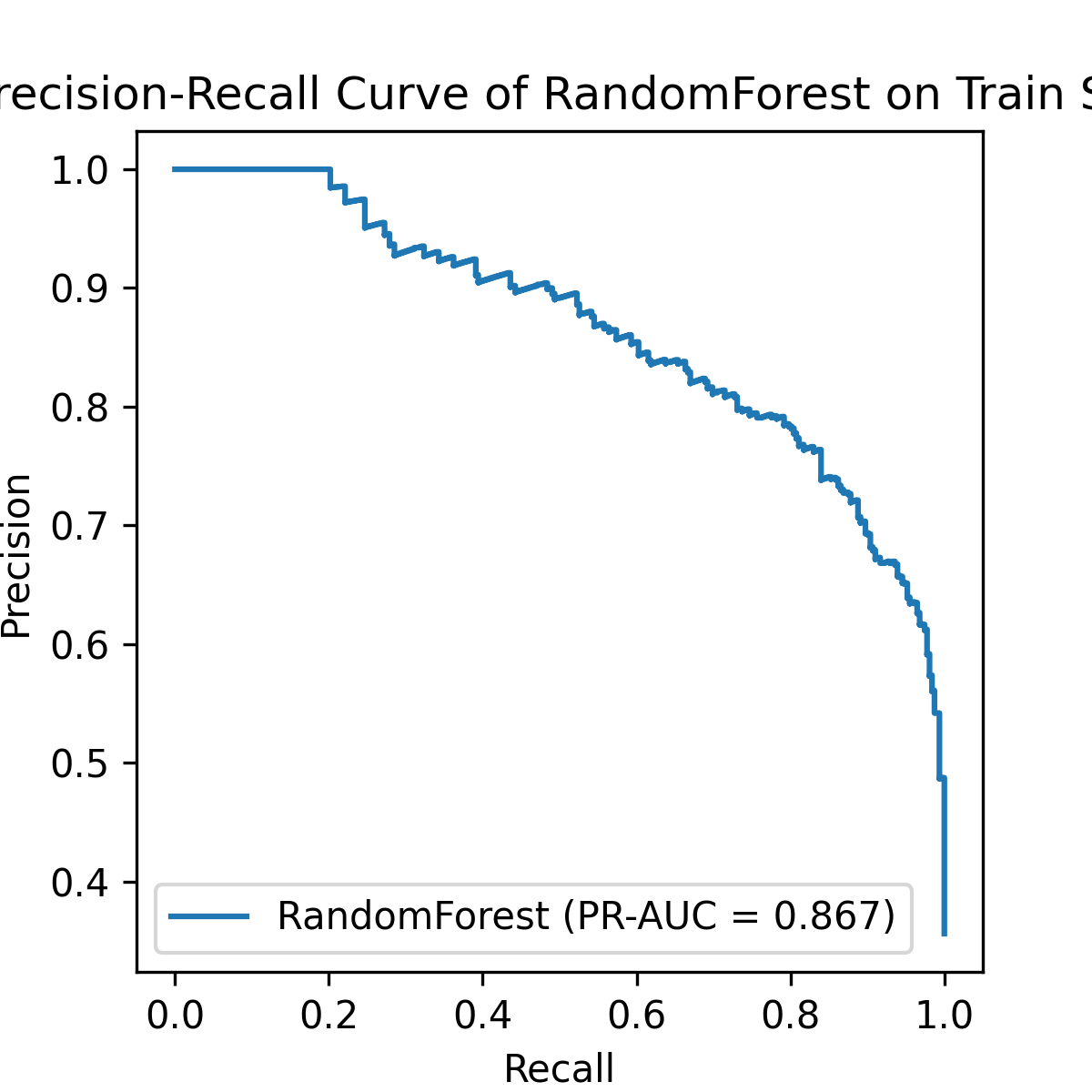

Supplement: Supplementary file 1 [file diagnostics-14-00053-s001.zip › Results of all classifiers/EmbeddingLR/RandomForest/Train Set/Precision-Recall Curve of RandomForest on Train Set.png]

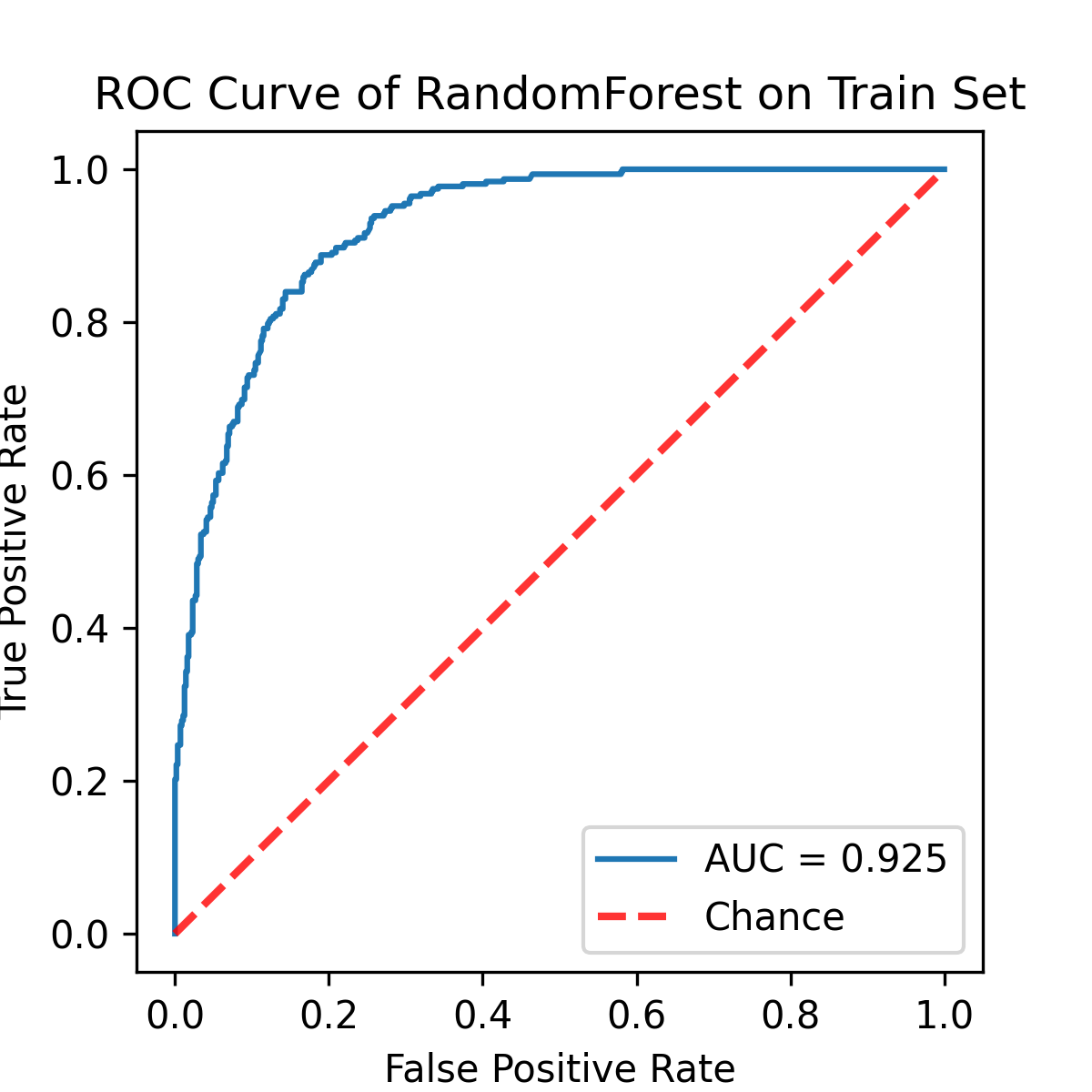

Supplement: Supplementary file 1 [file diagnostics-14-00053-s001.zip › Results of all classifiers/EmbeddingLR/RandomForest/Train Set/ROC Curve of RandomForest on Train Set.png]

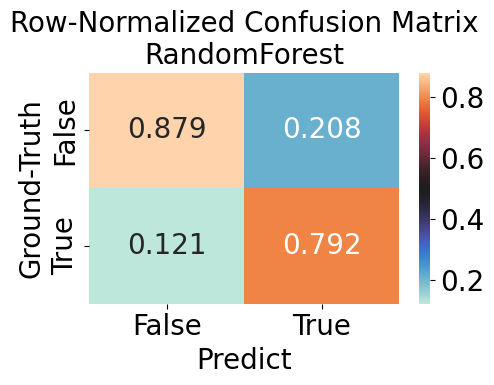

Supplement: Supplementary file 1 [file diagnostics-14-00053-s001.zip › Results of all classifiers/EmbeddingLR/RandomForest/Train Set/Row-Normalized Confusion Matrix RandomForest.png]

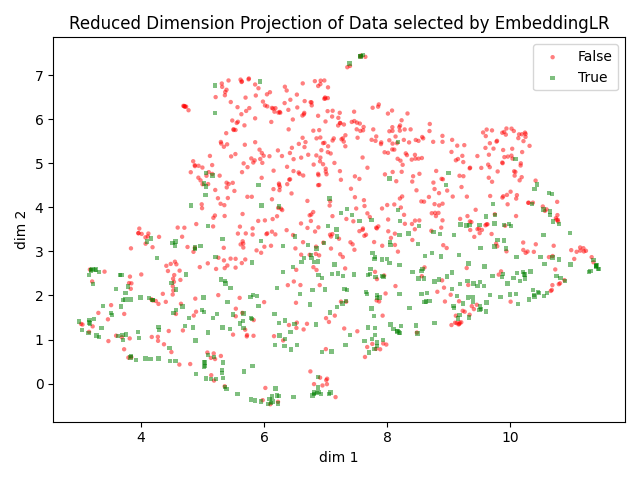

Supplement: Supplementary file 1 [file diagnostics-14-00053-s001.zip › Results of all classifiers/EmbeddingLR/Reduced Dimension Projection of Data selected by EmbeddingLR.png]

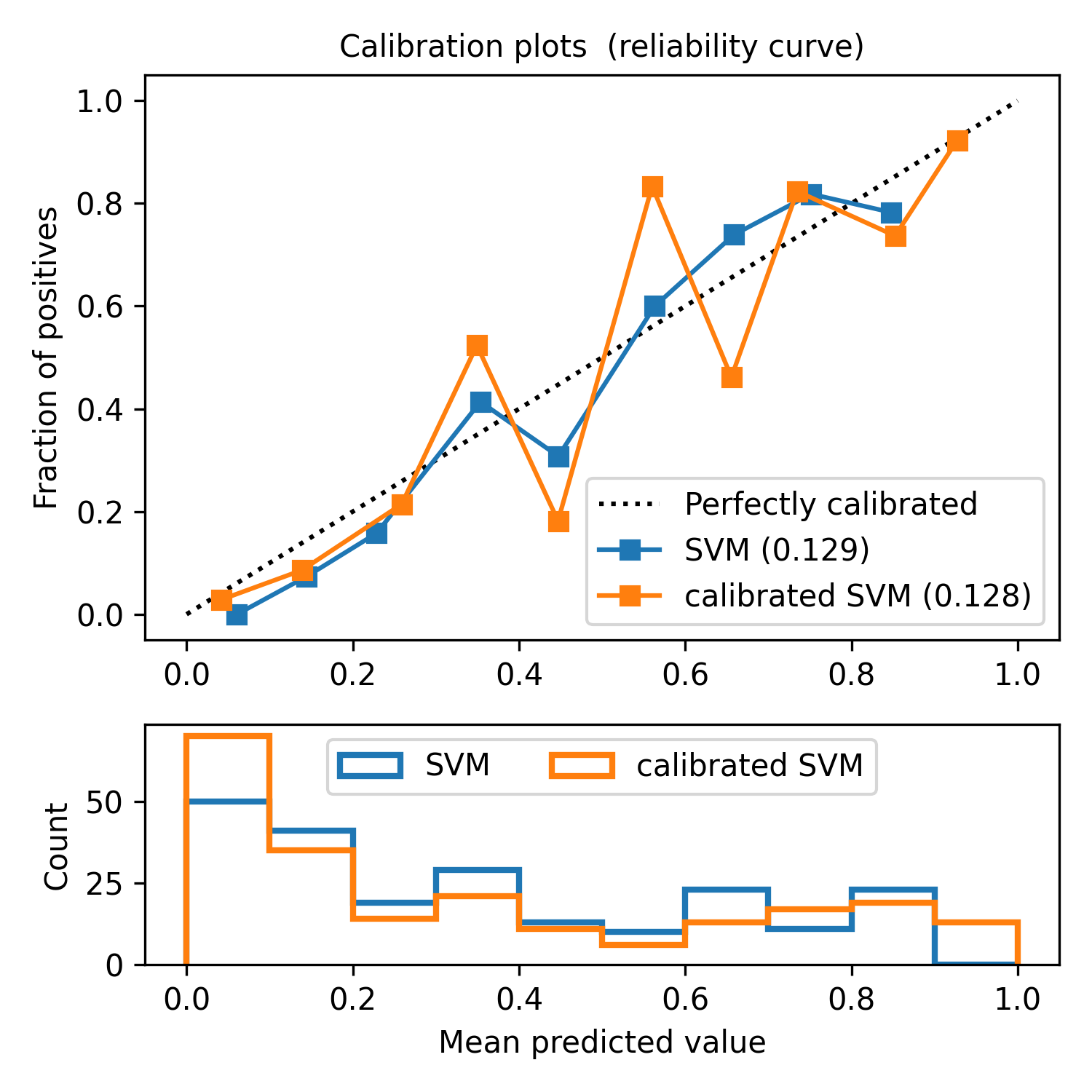

Supplement: Supplementary file 1 [file diagnostics-14-00053-s001.zip › Results of all classifiers/EmbeddingLR/SVM/Test Set/Calibration plots.png]

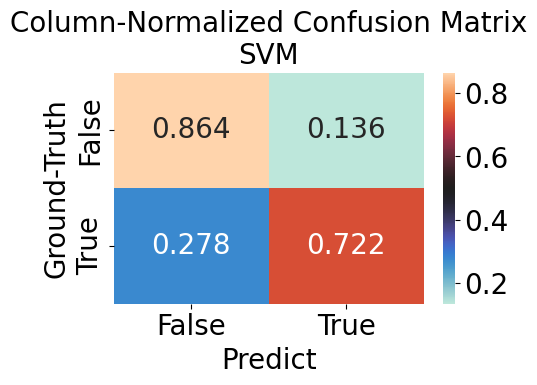

Supplement: Supplementary file 1 [file diagnostics-14-00053-s001.zip › Results of all classifiers/EmbeddingLR/SVM/Test Set/Column-Normalized Confusion Matrix SVM.png]

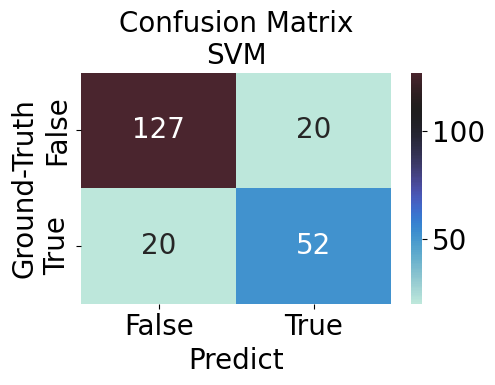

Supplement: Supplementary file 1 [file diagnostics-14-00053-s001.zip › Results of all classifiers/EmbeddingLR/SVM/Test Set/Confusion Matrix SVM.png]

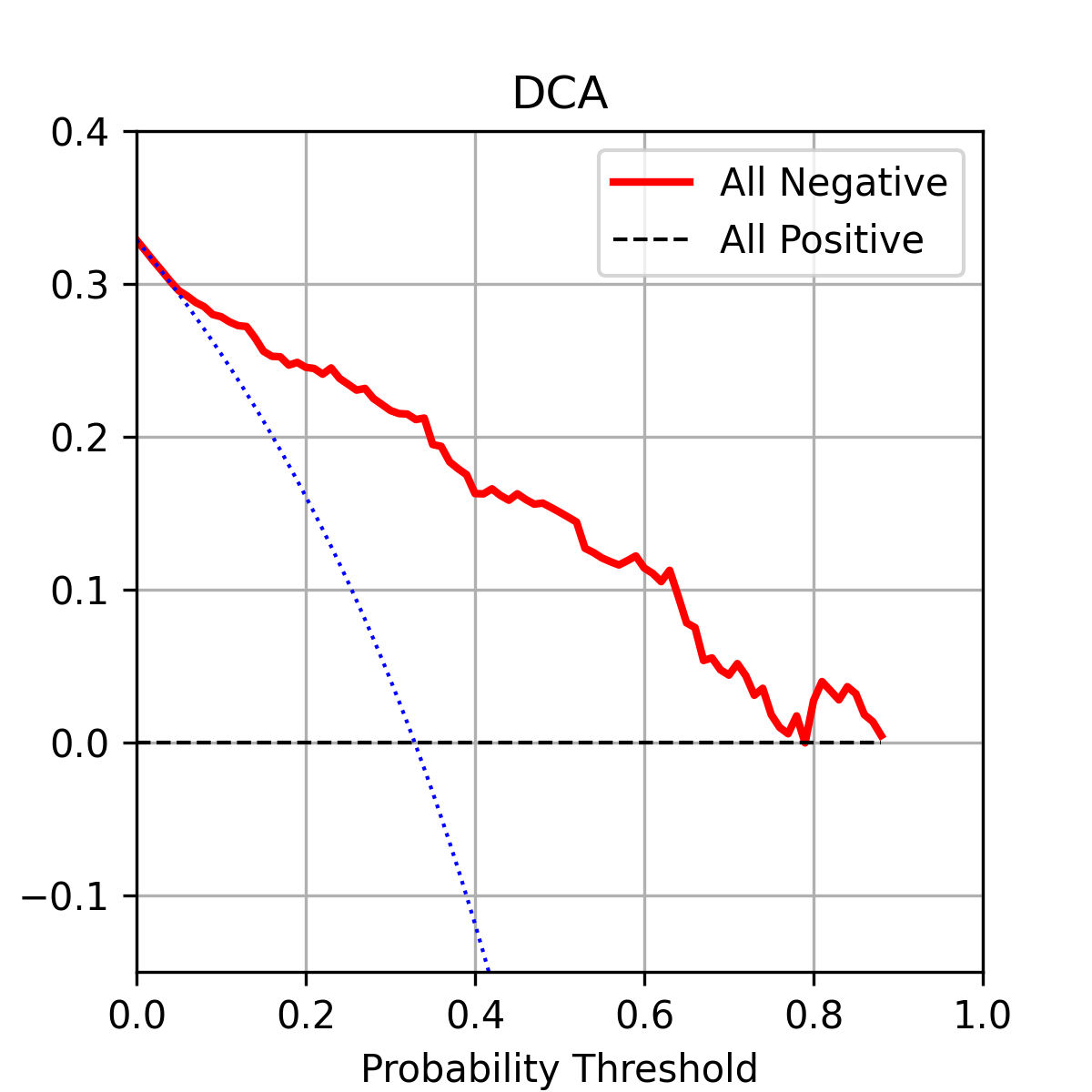

Supplement: Supplementary file 1 [file diagnostics-14-00053-s001.zip › Results of all classifiers/EmbeddingLR/SVM/Test Set/DCA.png]

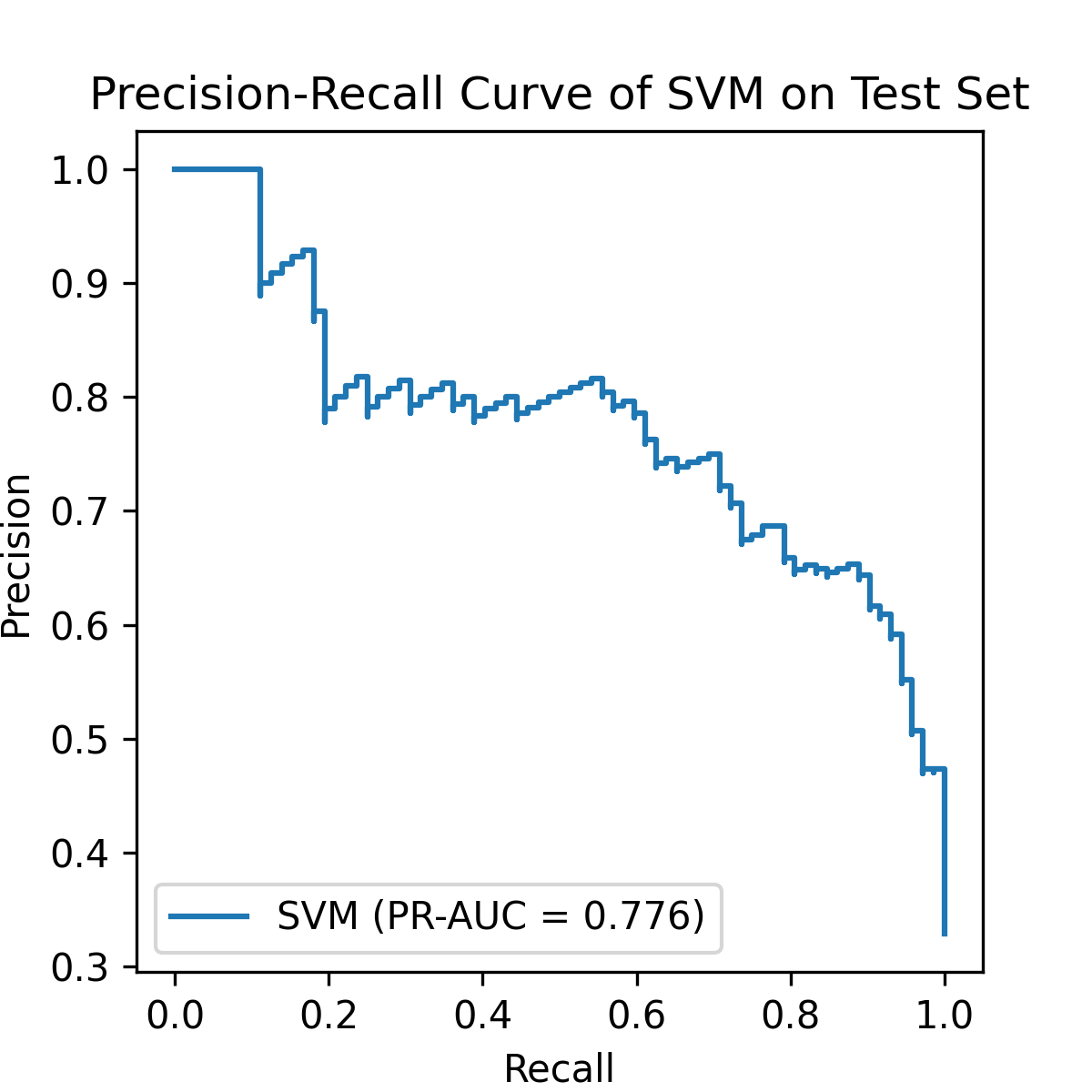

Supplement: Supplementary file 1 [file diagnostics-14-00053-s001.zip › Results of all classifiers/EmbeddingLR/SVM/Test Set/Precision-Recall Curve of SVM on Test Set.png]

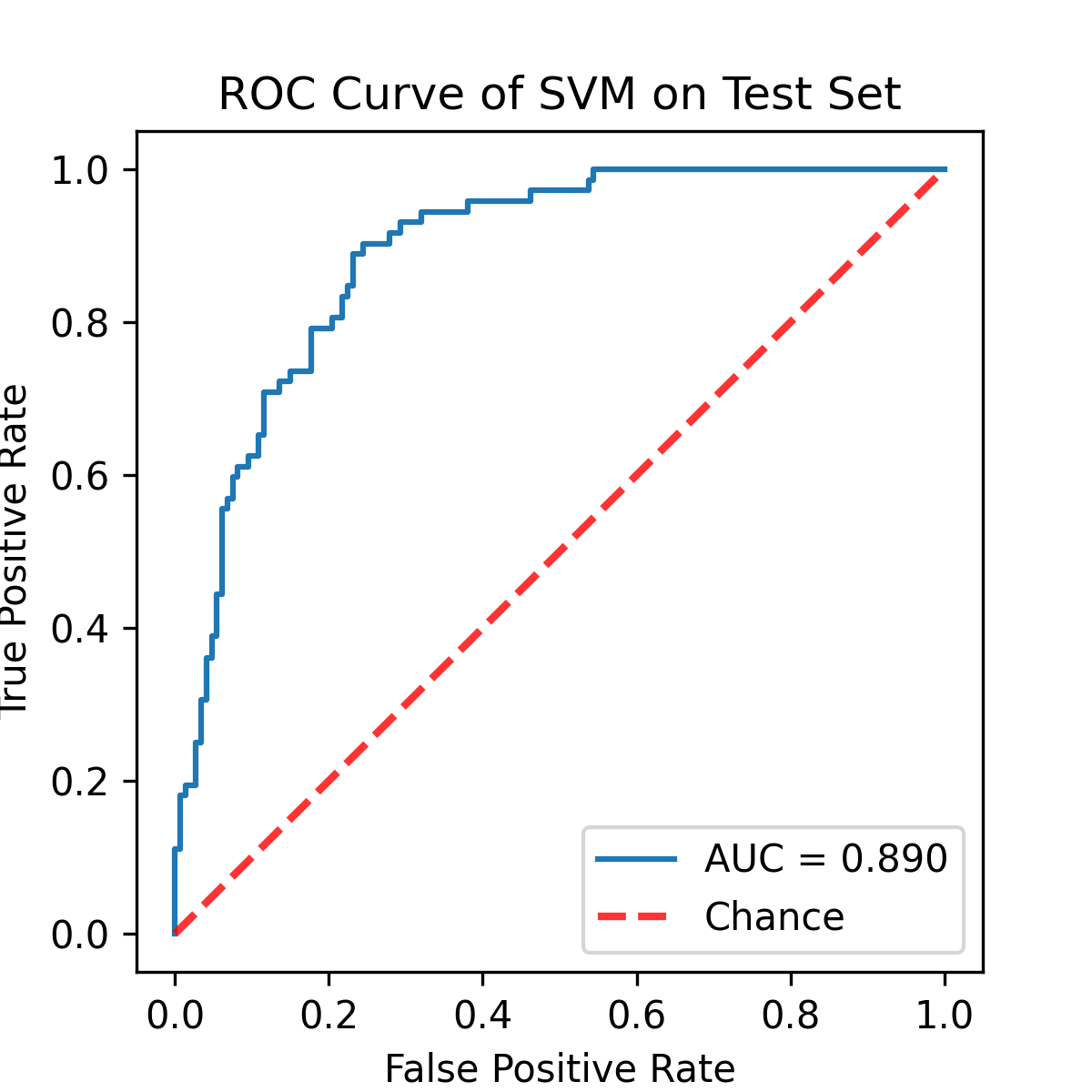

Supplement: Supplementary file 1 [file diagnostics-14-00053-s001.zip › Results of all classifiers/EmbeddingLR/SVM/Test Set/ROC Curve of SVM on Test Set.png]

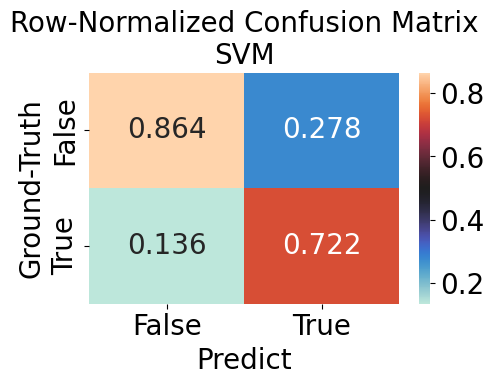

Supplement: Supplementary file 1 [file diagnostics-14-00053-s001.zip › Results of all classifiers/EmbeddingLR/SVM/Test Set/Row-Normalized Confusion Matrix SVM.png]

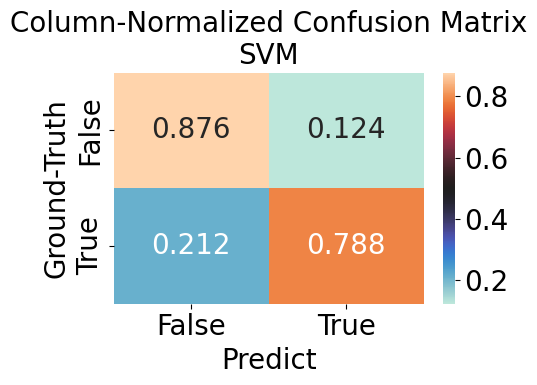

Supplement: Supplementary file 1 [file diagnostics-14-00053-s001.zip › Results of all classifiers/EmbeddingLR/SVM/Train Set/Column-Normalized Confusion Matrix SVM.png]

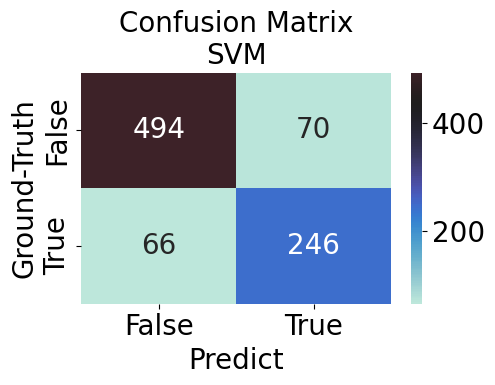

Supplement: Supplementary file 1 [file diagnostics-14-00053-s001.zip › Results of all classifiers/EmbeddingLR/SVM/Train Set/Confusion Matrix SVM.png]

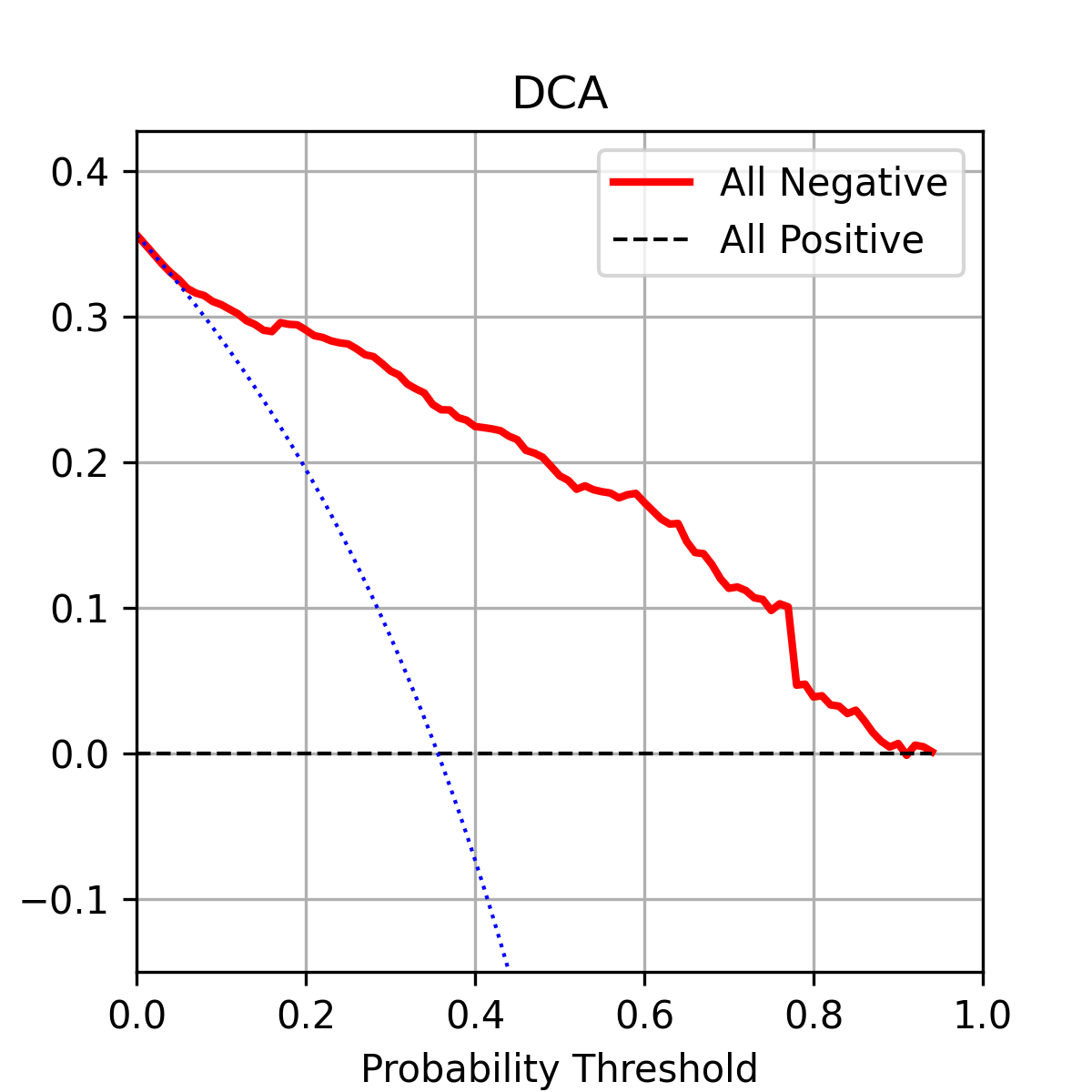

Supplement: Supplementary file 1 [file diagnostics-14-00053-s001.zip › Results of all classifiers/EmbeddingLR/SVM/Train Set/DCA.png]

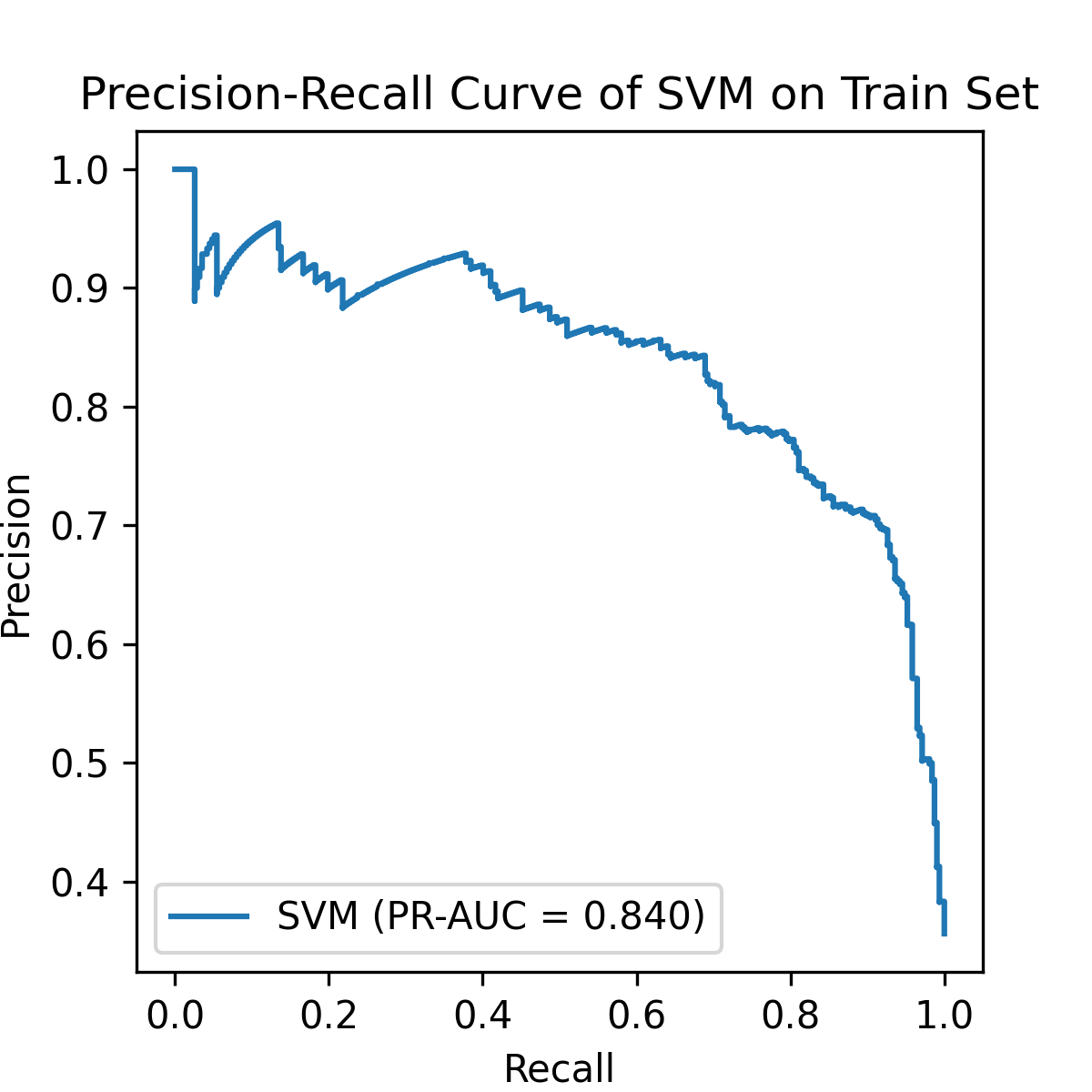

Supplement: Supplementary file 1 [file diagnostics-14-00053-s001.zip › Results of all classifiers/EmbeddingLR/SVM/Train Set/Precision-Recall Curve of SVM on Train Set.png]

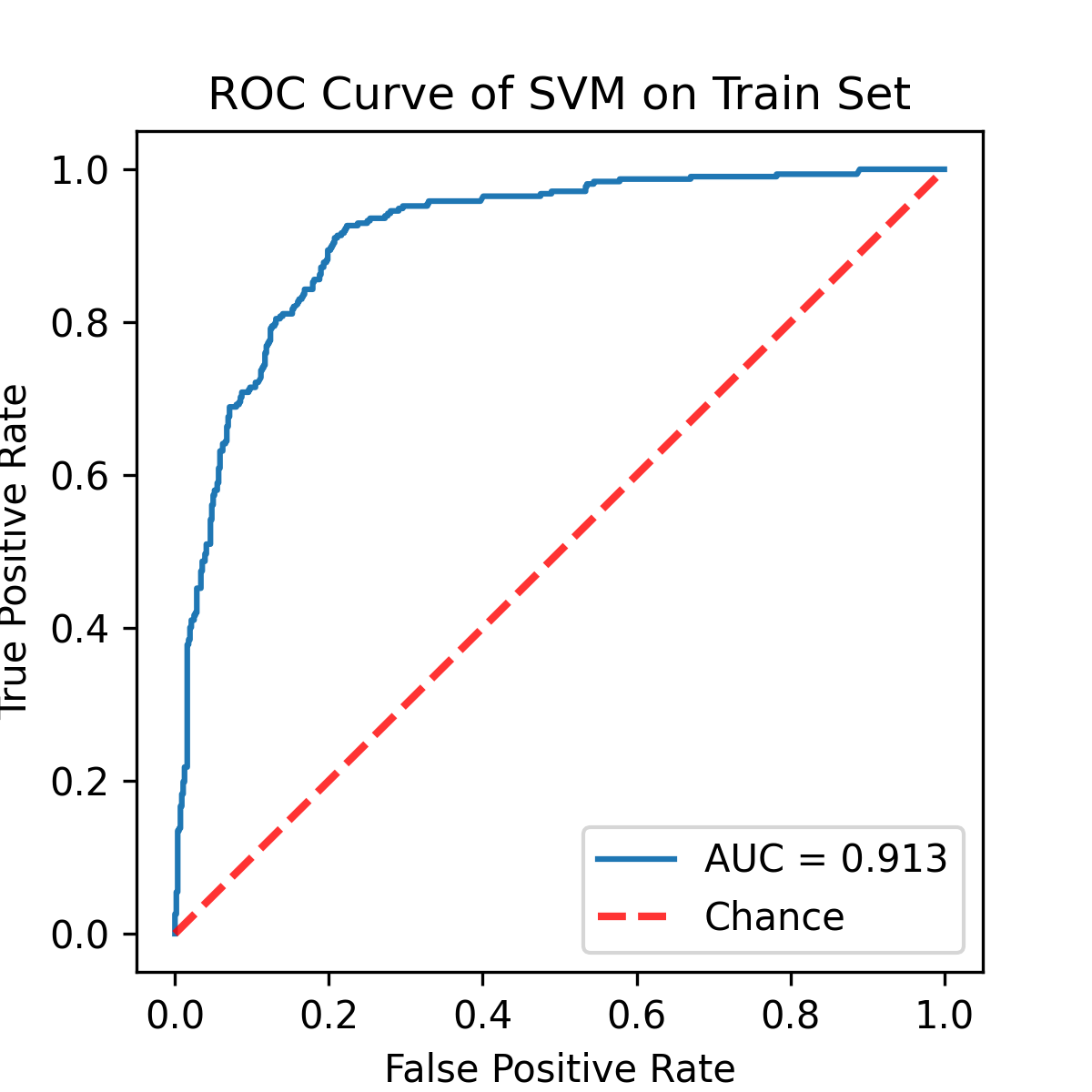

Supplement: Supplementary file 1 [file diagnostics-14-00053-s001.zip › Results of all classifiers/EmbeddingLR/SVM/Train Set/ROC Curve of SVM on Train Set.png]

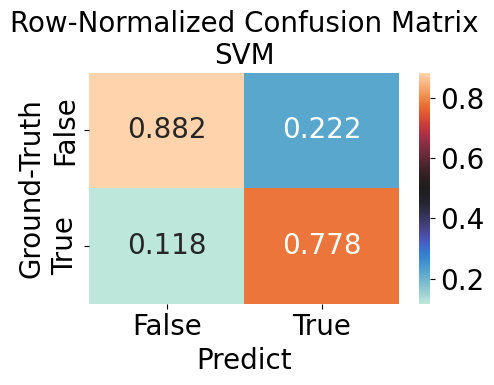

Supplement: Supplementary file 1 [file diagnostics-14-00053-s001.zip › Results of all classifiers/EmbeddingLR/SVM/Train Set/Row-Normalized Confusion Matrix SVM.png]

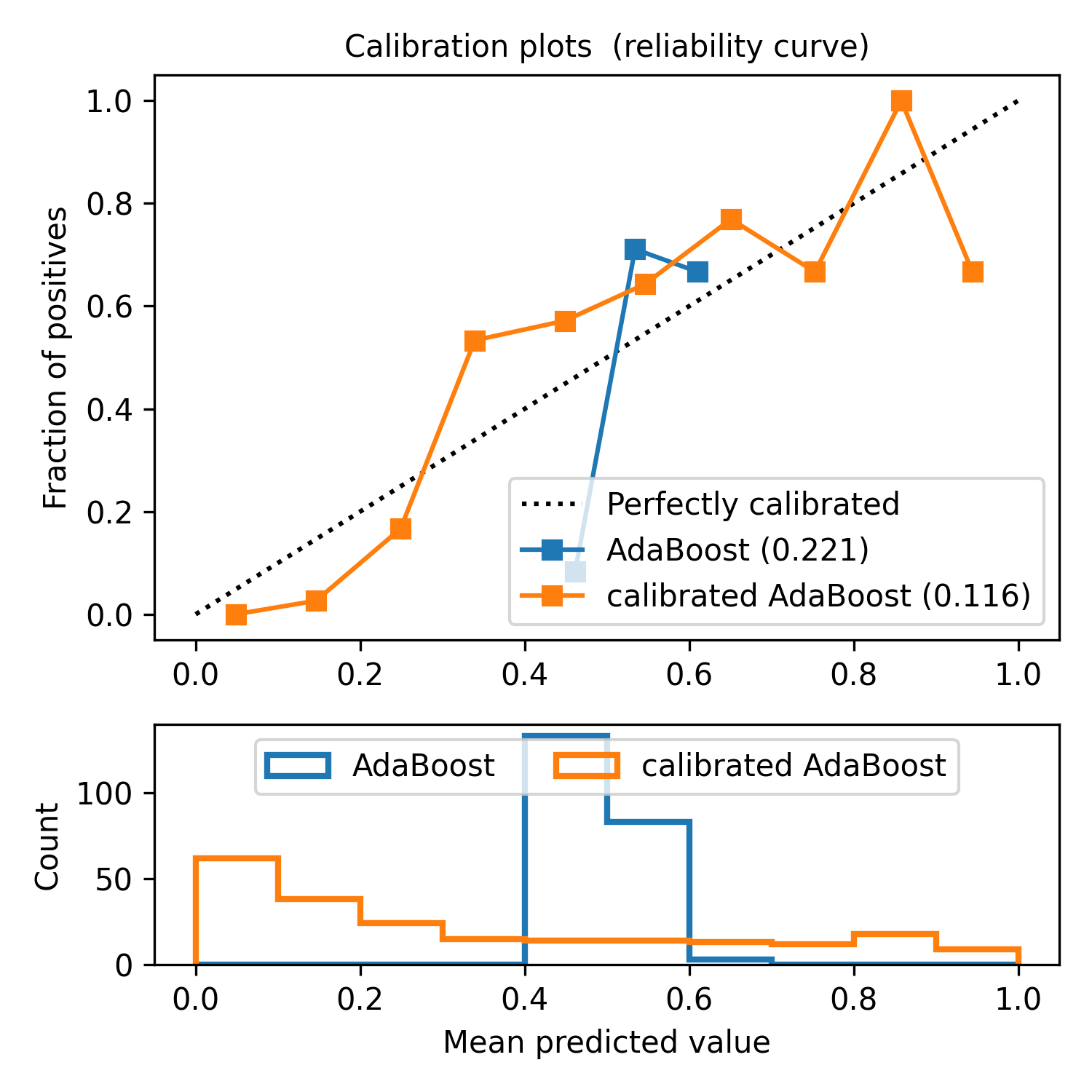

Supplement: Supplementary file 1 [file diagnostics-14-00053-s001.zip › Results of all classifiers/EmbeddingLSVC/AdaBoost/Test Set/Calibration plots.png]

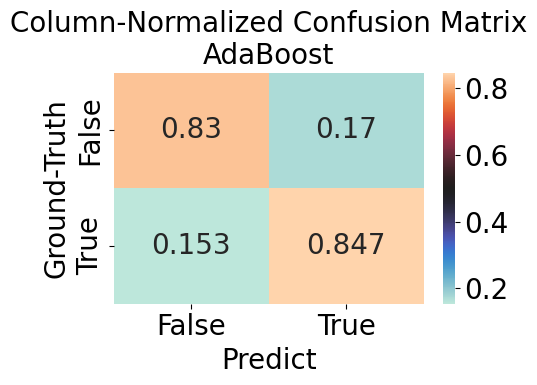

Supplement: Supplementary file 1 [file diagnostics-14-00053-s001.zip › Results of all classifiers/EmbeddingLSVC/AdaBoost/Test Set/Column-Normalized Confusion Matrix AdaBoost.png]

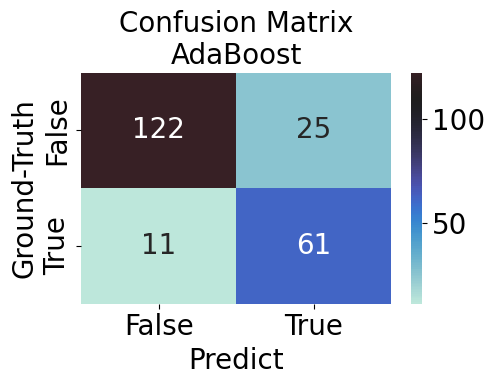

Supplement: Supplementary file 1 [file diagnostics-14-00053-s001.zip › Results of all classifiers/EmbeddingLSVC/AdaBoost/Test Set/Confusion Matrix AdaBoost.png]

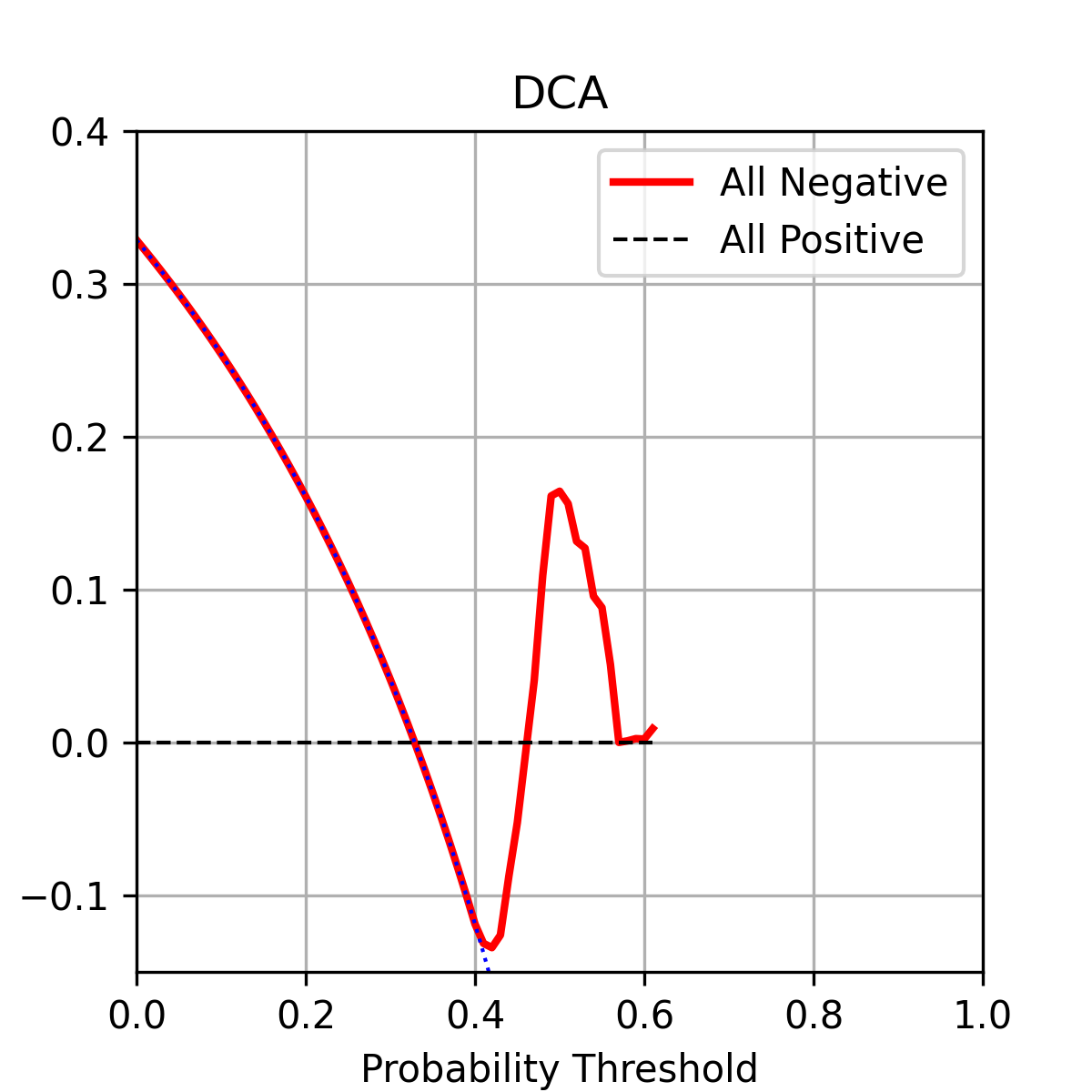

Supplement: Supplementary file 1 [file diagnostics-14-00053-s001.zip › Results of all classifiers/EmbeddingLSVC/AdaBoost/Test Set/DCA.png]

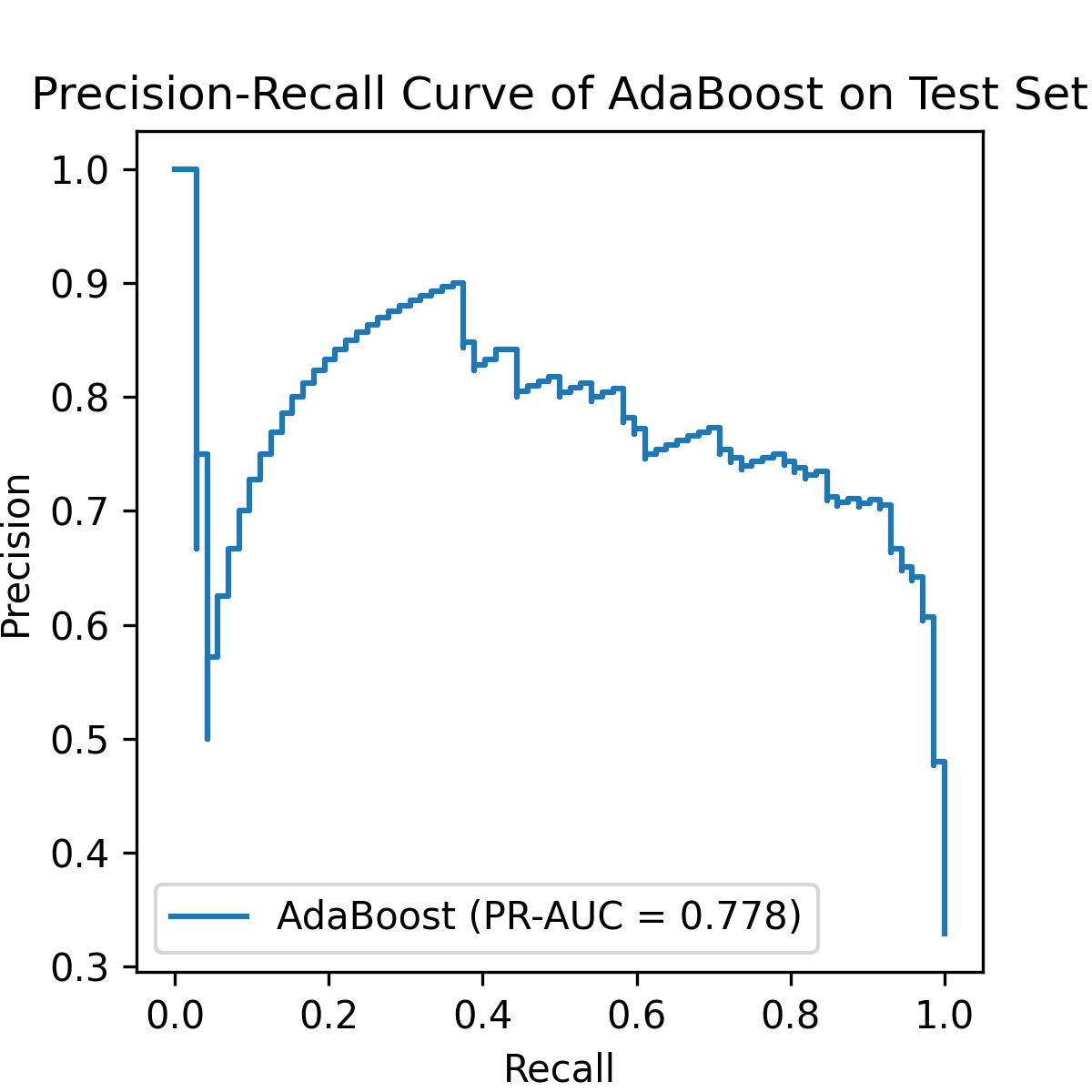

Supplement: Supplementary file 1 [file diagnostics-14-00053-s001.zip › Results of all classifiers/EmbeddingLSVC/AdaBoost/Test Set/Precision-Recall Curve of AdaBoost on Test Set.png]

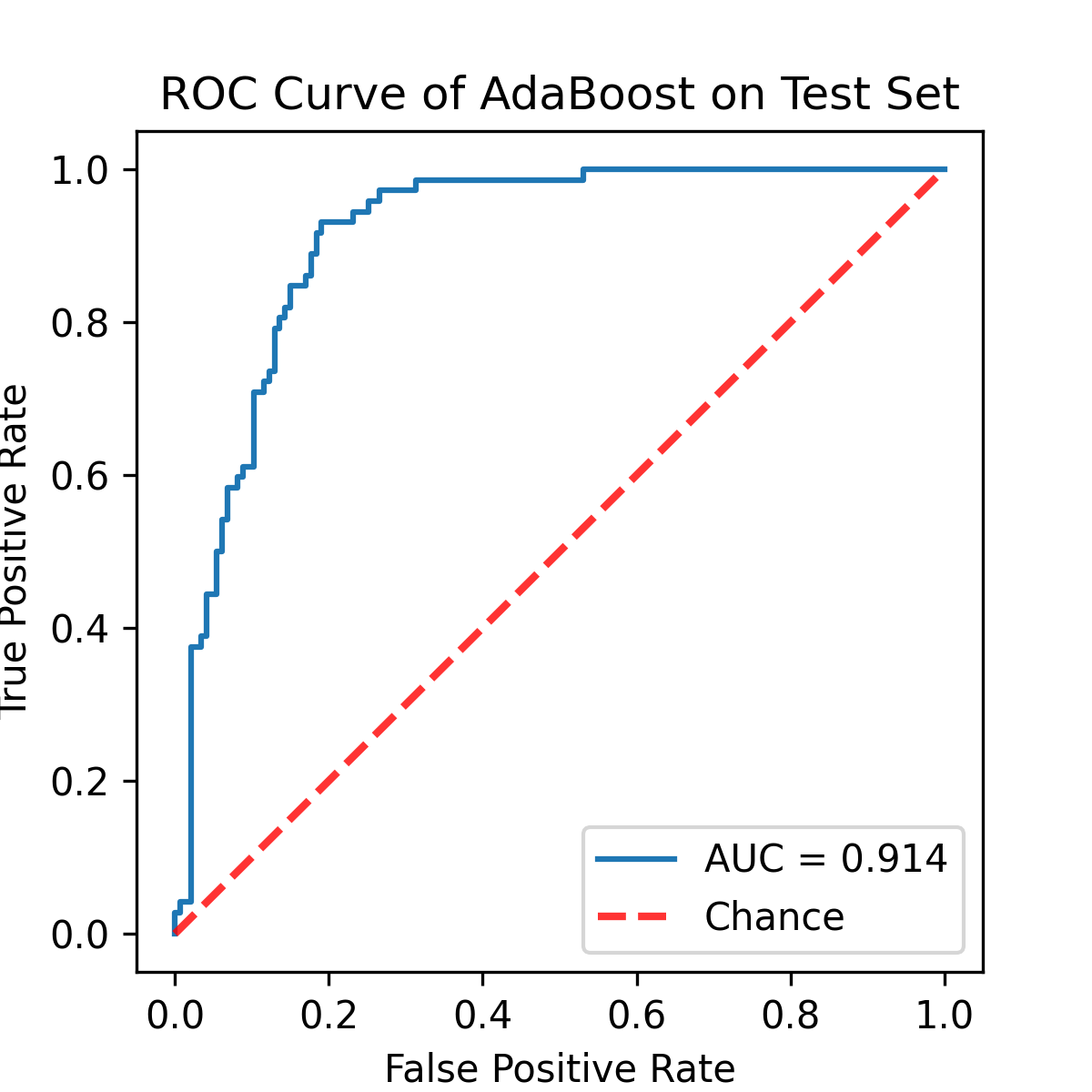

Supplement: Supplementary file 1 [file diagnostics-14-00053-s001.zip › Results of all classifiers/EmbeddingLSVC/AdaBoost/Test Set/ROC Curve of AdaBoost on Test Set.png]

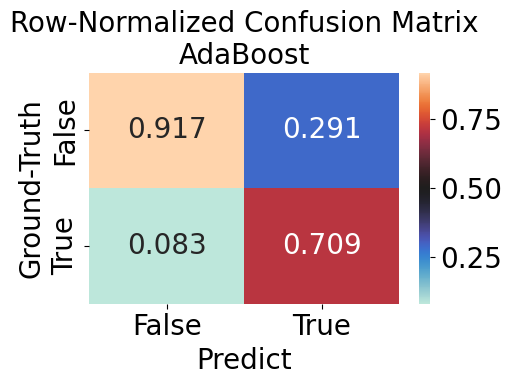

Supplement: Supplementary file 1 [file diagnostics-14-00053-s001.zip › Results of all classifiers/EmbeddingLSVC/AdaBoost/Test Set/Row-Normalized Confusion Matrix AdaBoost.png]

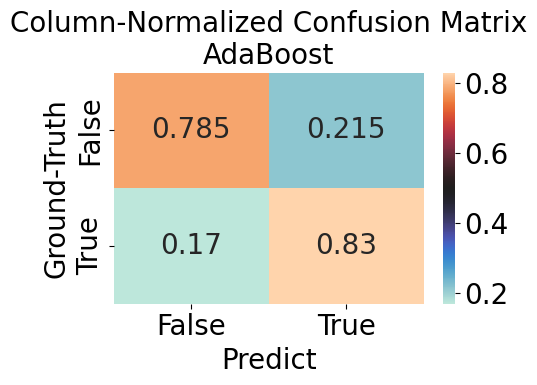

Supplement: Supplementary file 1 [file diagnostics-14-00053-s001.zip › Results of all classifiers/EmbeddingLSVC/AdaBoost/Train Set/Column-Normalized Confusion Matrix AdaBoost.png]

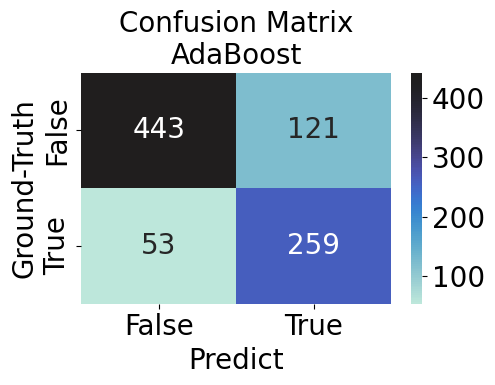

Supplement: Supplementary file 1 [file diagnostics-14-00053-s001.zip › Results of all classifiers/EmbeddingLSVC/AdaBoost/Train Set/Confusion Matrix AdaBoost.png]

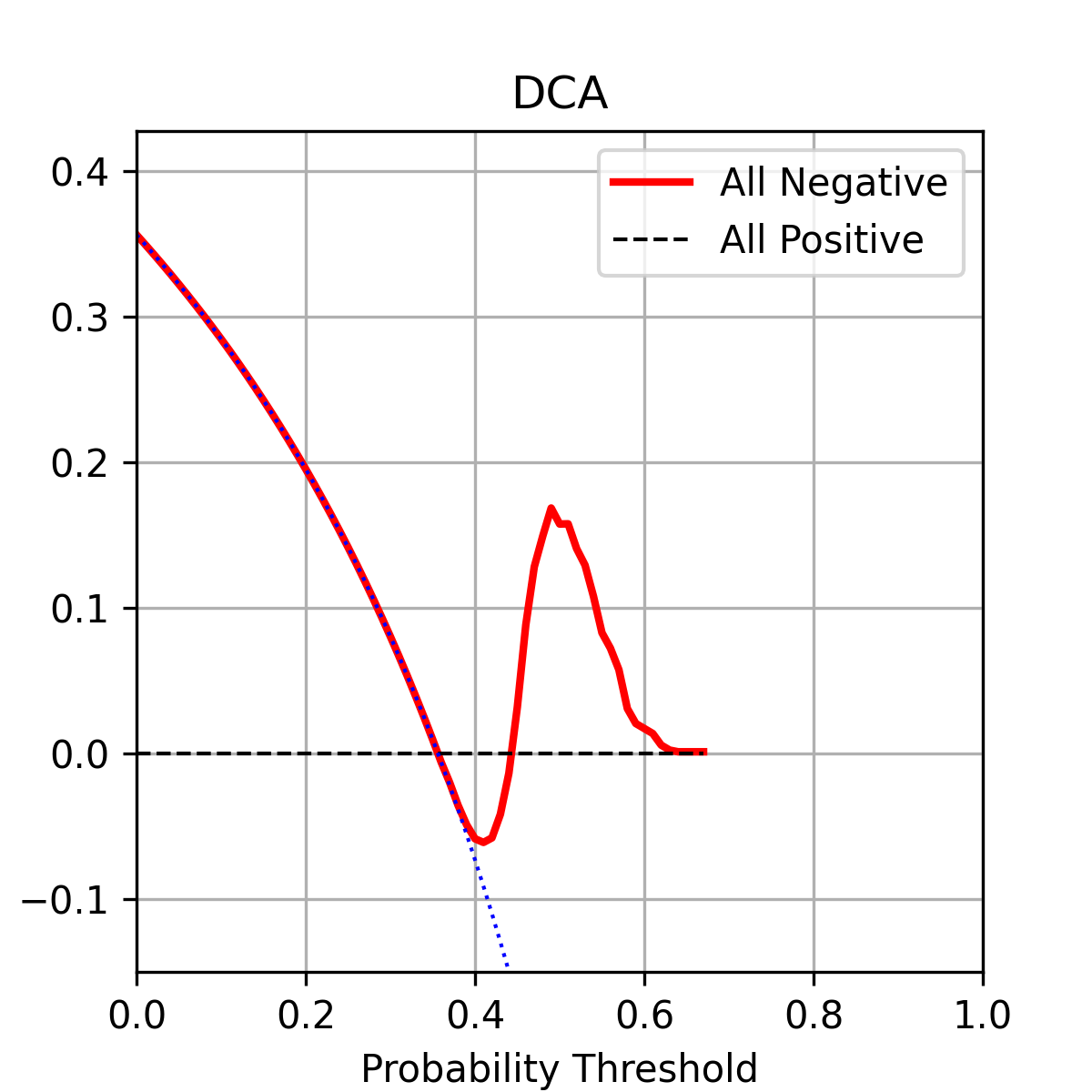

Supplement: Supplementary file 1 [file diagnostics-14-00053-s001.zip › Results of all classifiers/EmbeddingLSVC/AdaBoost/Train Set/DCA.png]

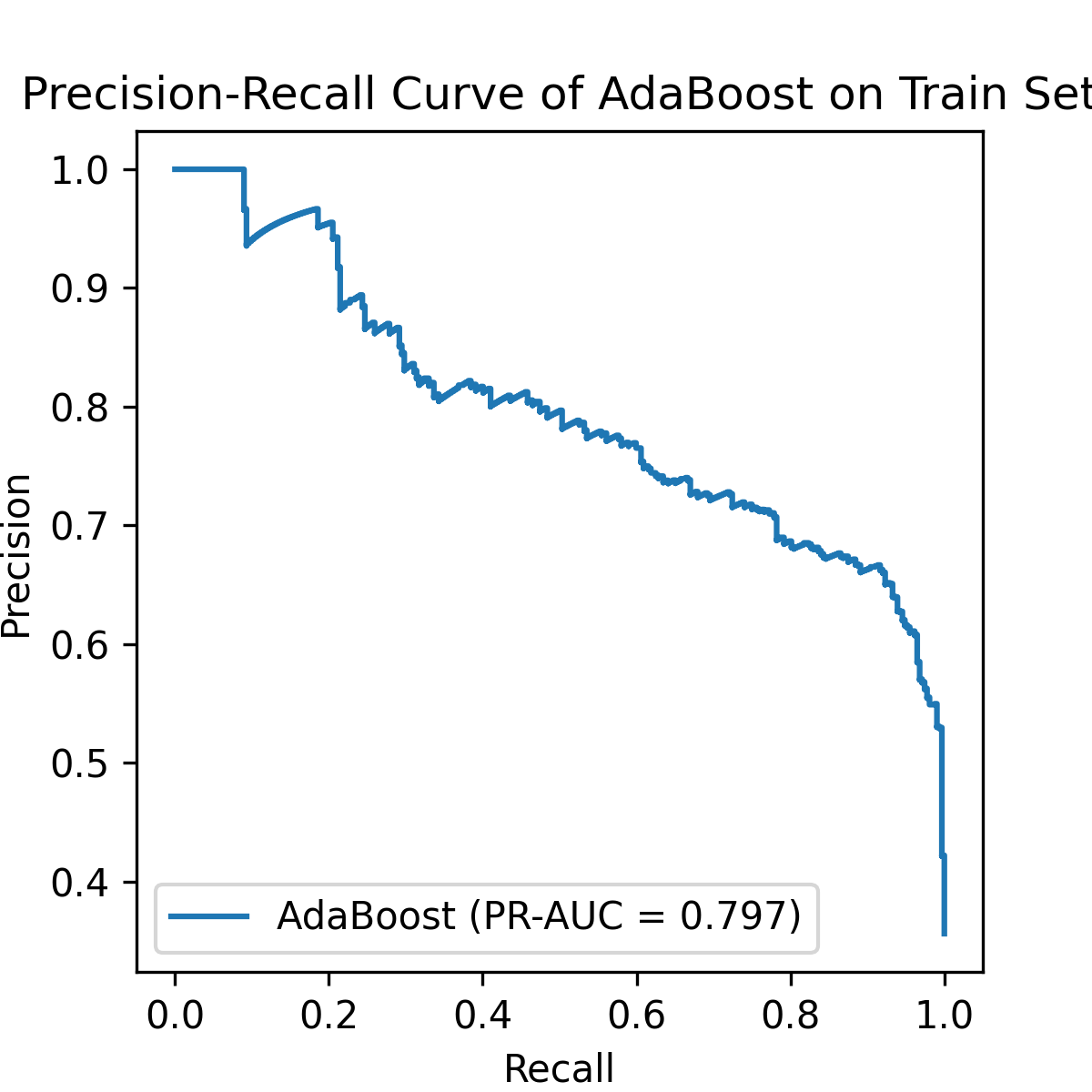

Supplement: Supplementary file 1 [file diagnostics-14-00053-s001.zip › Results of all classifiers/EmbeddingLSVC/AdaBoost/Train Set/Precision-Recall Curve of AdaBoost on Train Set.png]

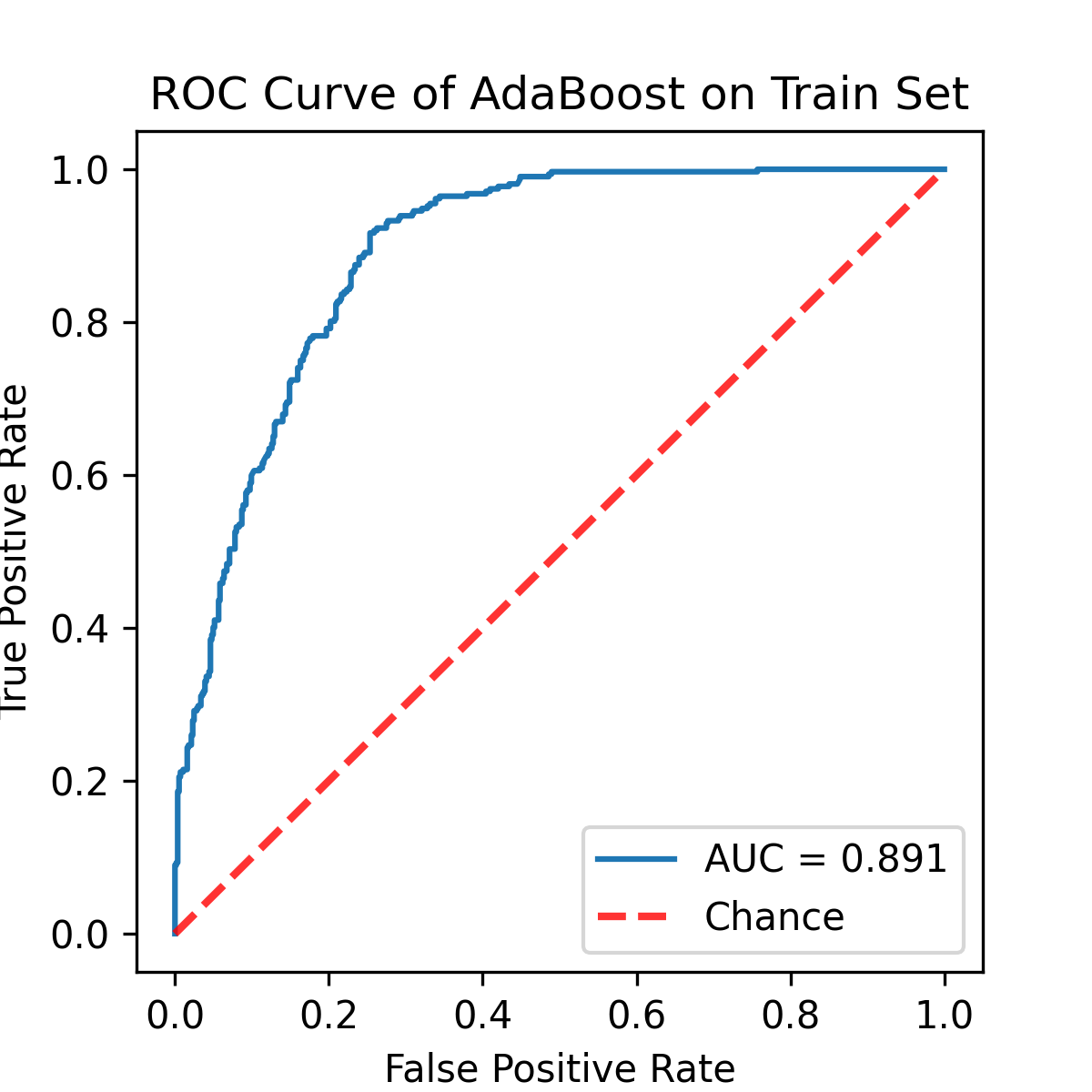

Supplement: Supplementary file 1 [file diagnostics-14-00053-s001.zip › Results of all classifiers/EmbeddingLSVC/AdaBoost/Train Set/ROC Curve of AdaBoost on Train Set.png]

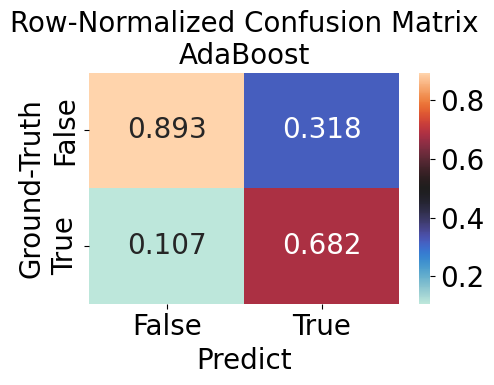

Supplement: Supplementary file 1 [file diagnostics-14-00053-s001.zip › Results of all classifiers/EmbeddingLSVC/AdaBoost/Train Set/Row-Normalized Confusion Matrix AdaBoost.png]

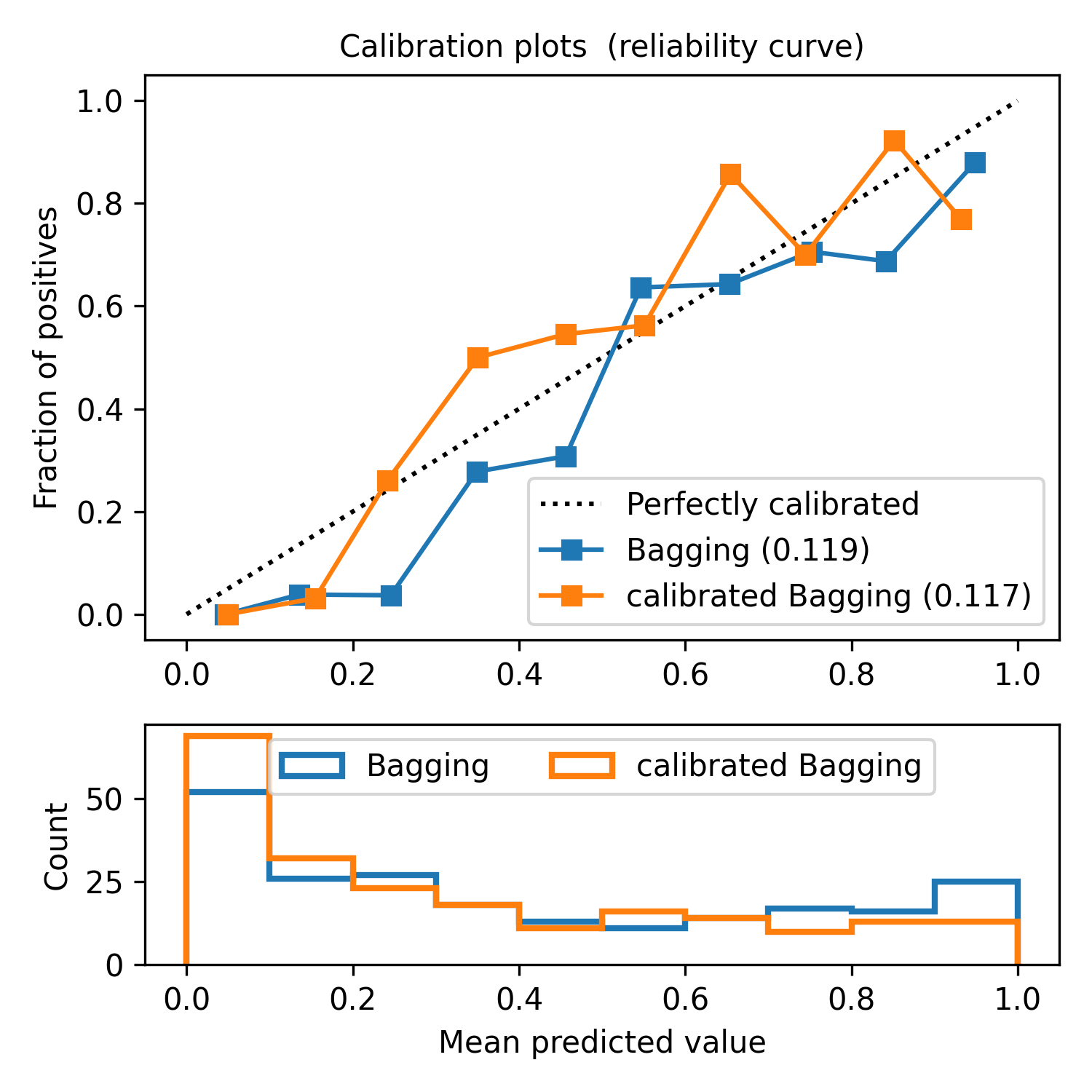

Supplement: Supplementary file 1 [file diagnostics-14-00053-s001.zip › Results of all classifiers/EmbeddingLSVC/Bagging/Test Set/Calibration plots.png]
