# Supplementary figures and images for: Machine Learning Predicts Decompression Levels for Lumbar Spinal Stenosis Using Canal Radiomic Features from Computed Tomography Myelography (part 1 of 2)
Source: Diagnostics (Basel). 2023 Dec 26;14(1):53. doi: 10.3390/diagnostics14010053 (PMC10795799; doi:10.3390/diagnostics14010053)

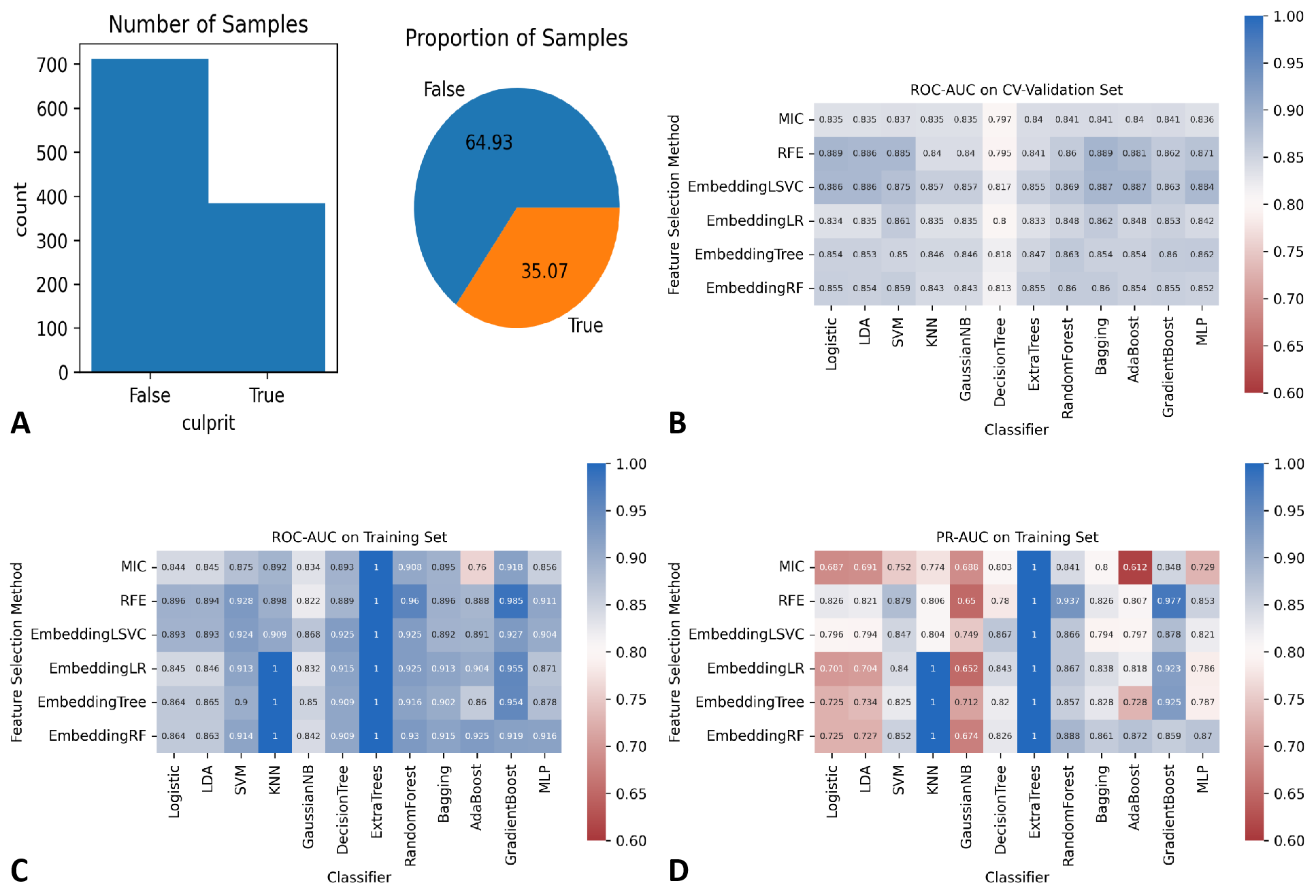

Supplement: Supplementary file 1 [file diagnostics-14-00053-s001.zip › Figure S1.tif]

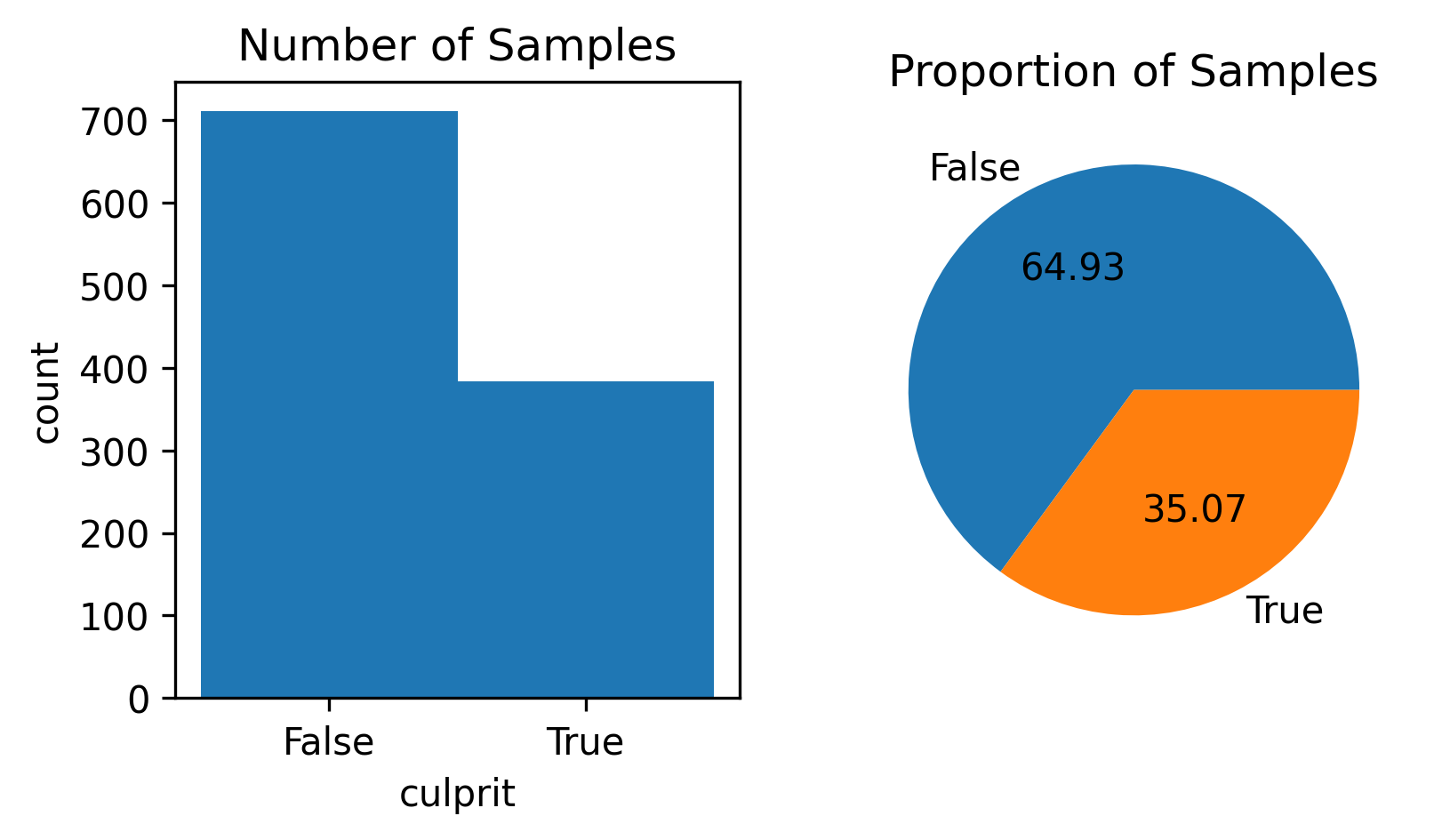

Supplement: Supplementary file 1 [file diagnostics-14-00053-s001.zip › Results of all classifiers/Distribution of Samples for Different Categories in the Dataset.png]

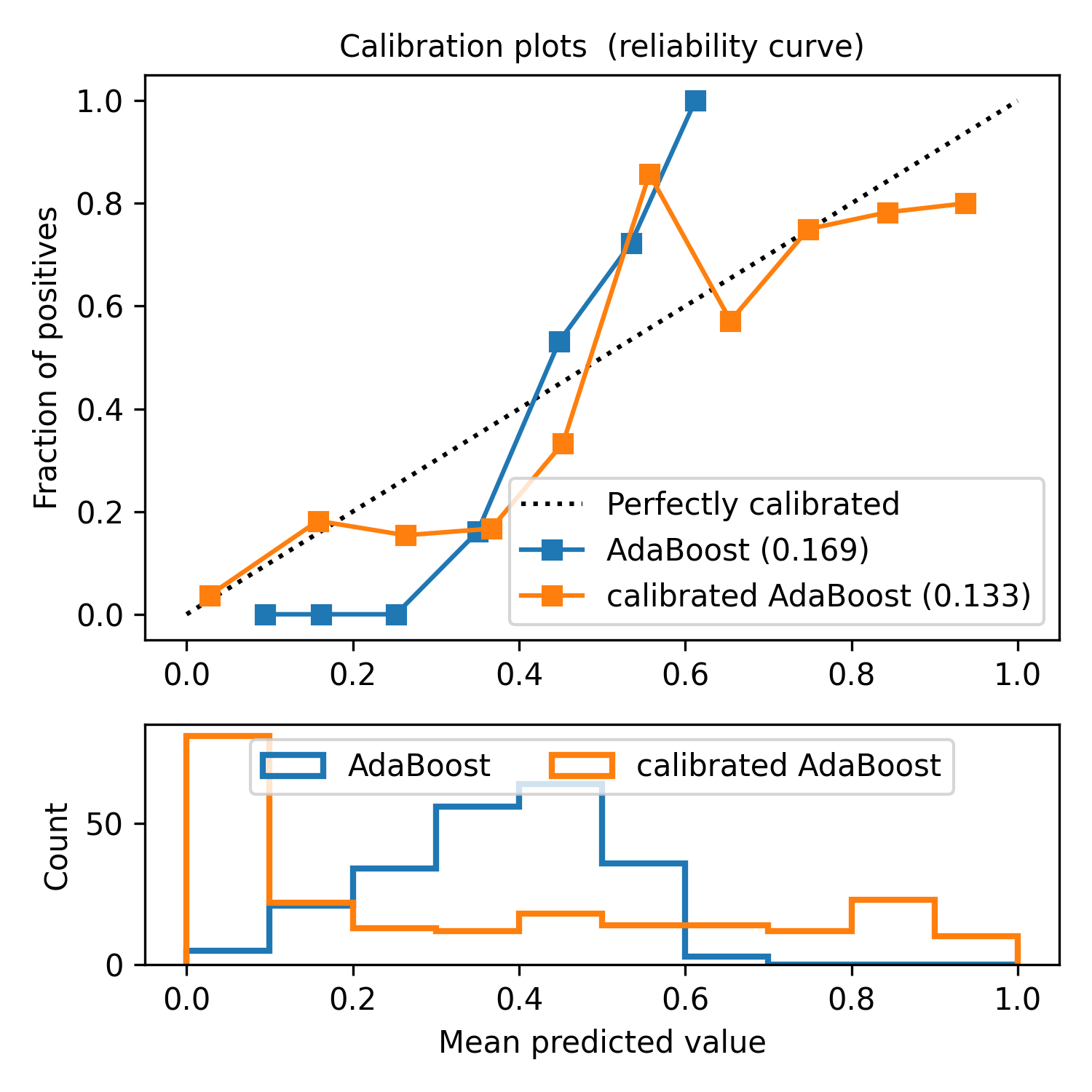

Supplement: Supplementary file 1 [file diagnostics-14-00053-s001.zip › Results of all classifiers/EmbeddingLR/AdaBoost/Test Set/Calibration plots.png]

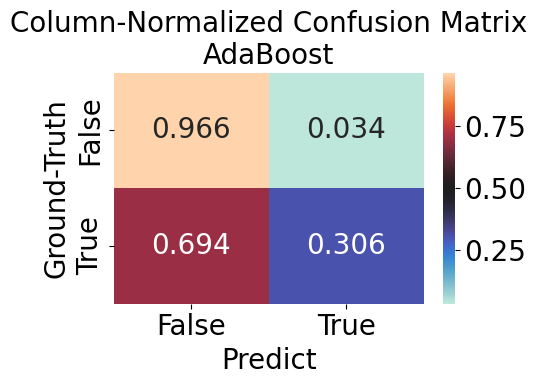

Supplement: Supplementary file 1 [file diagnostics-14-00053-s001.zip › Results of all classifiers/EmbeddingLR/AdaBoost/Test Set/Column-Normalized Confusion Matrix AdaBoost.png]

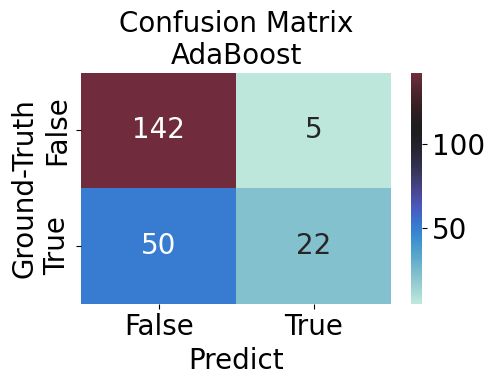

Supplement: Supplementary file 1 [file diagnostics-14-00053-s001.zip › Results of all classifiers/EmbeddingLR/AdaBoost/Test Set/Confusion Matrix AdaBoost.png]

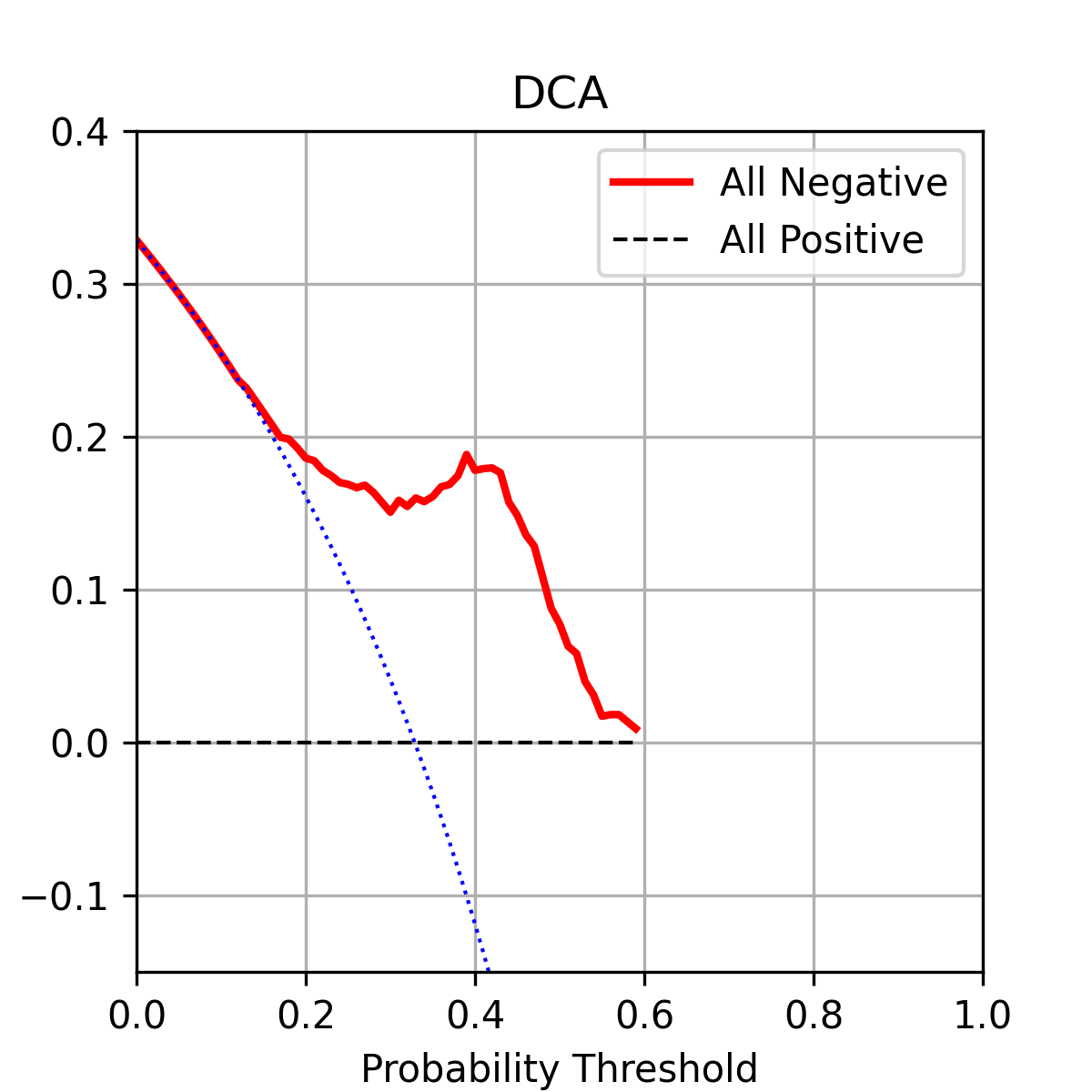

Supplement: Supplementary file 1 [file diagnostics-14-00053-s001.zip › Results of all classifiers/EmbeddingLR/AdaBoost/Test Set/DCA.png]

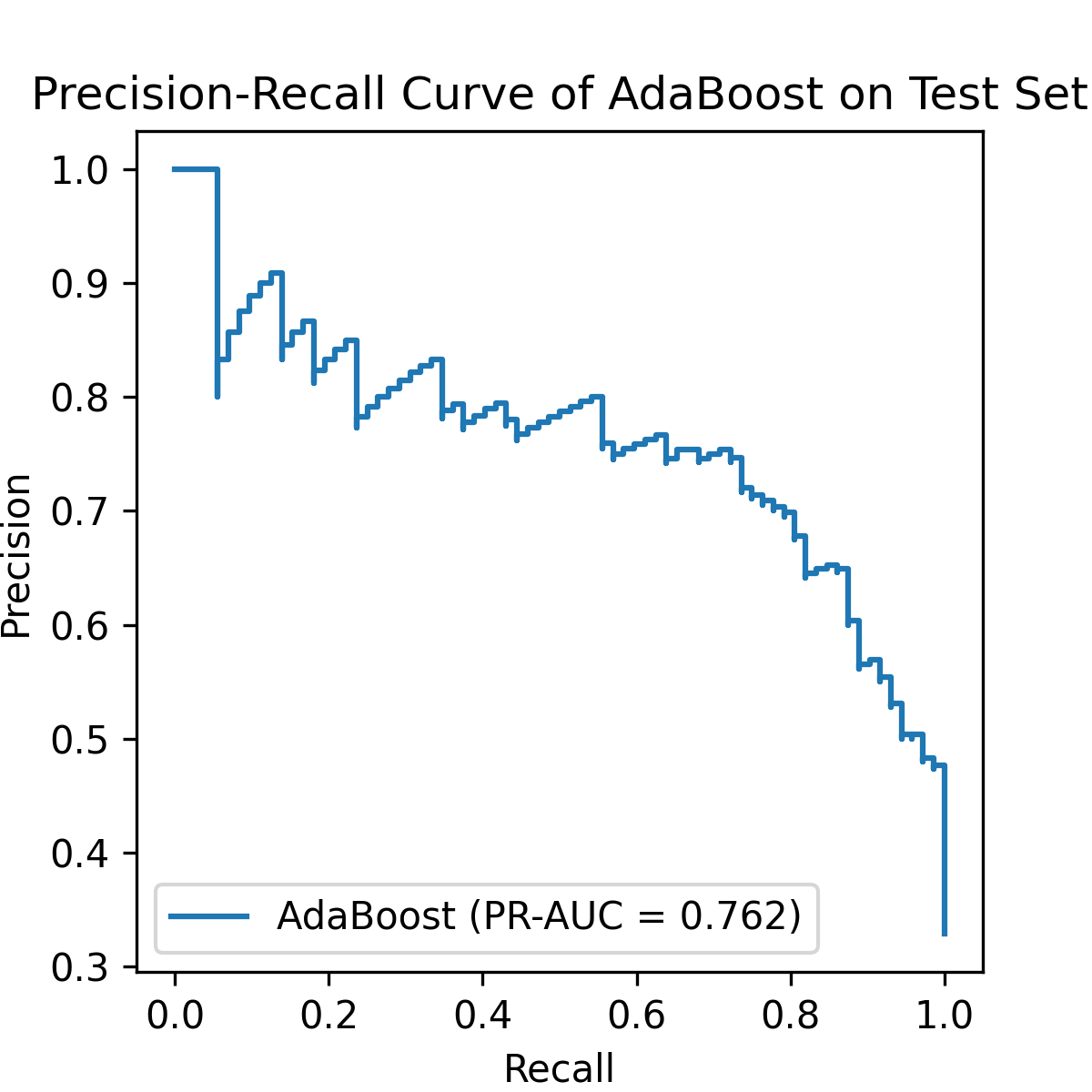

Supplement: Supplementary file 1 [file diagnostics-14-00053-s001.zip › Results of all classifiers/EmbeddingLR/AdaBoost/Test Set/Precision-Recall Curve of AdaBoost on Test Set.png]

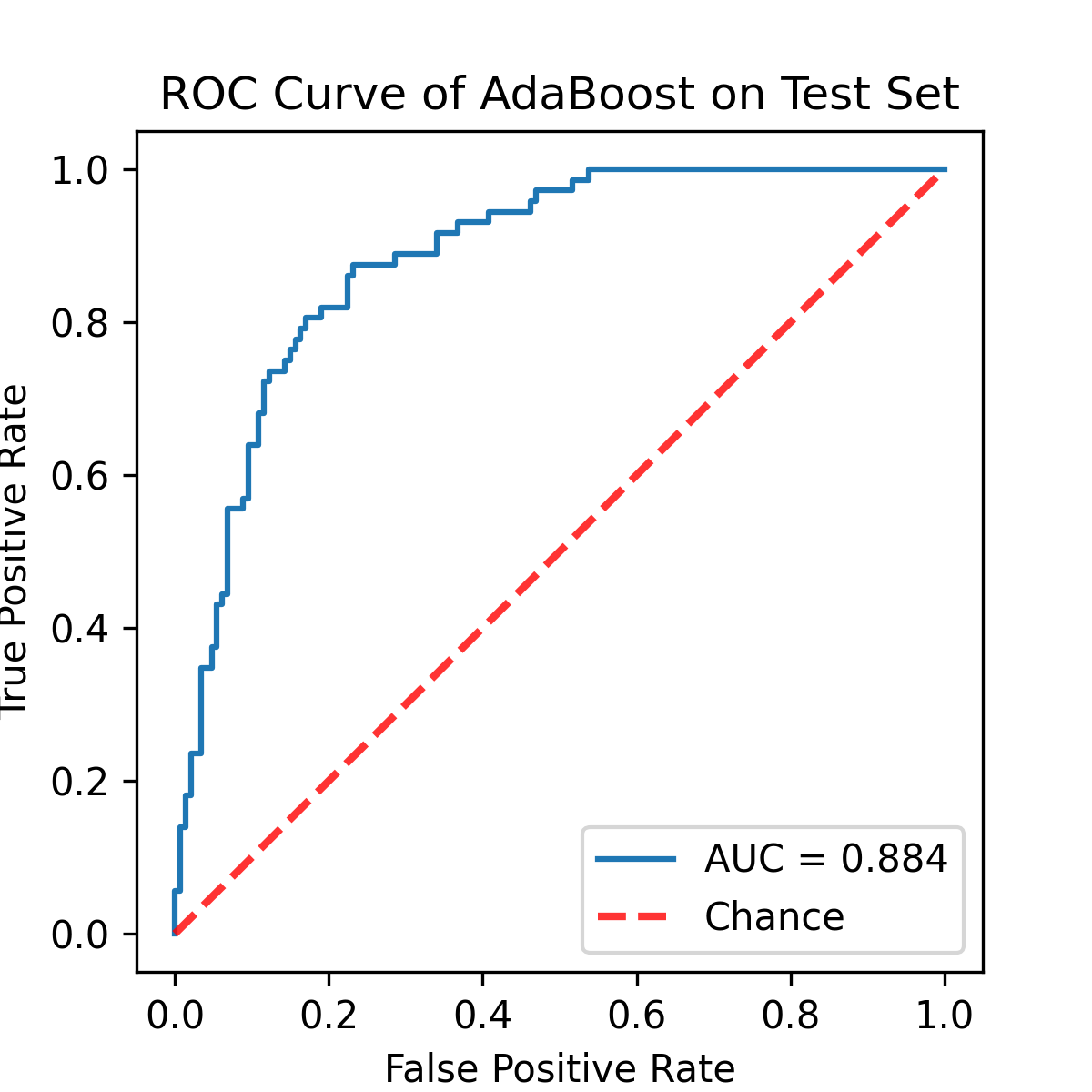

Supplement: Supplementary file 1 [file diagnostics-14-00053-s001.zip › Results of all classifiers/EmbeddingLR/AdaBoost/Test Set/ROC Curve of AdaBoost on Test Set.png]

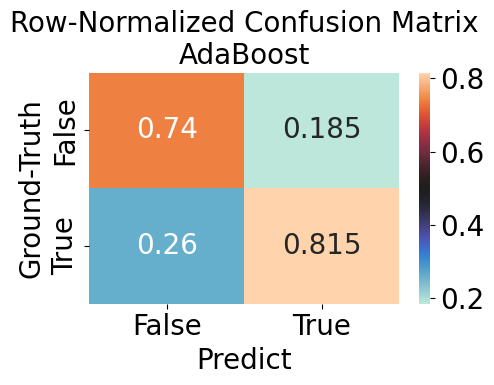

Supplement: Supplementary file 1 [file diagnostics-14-00053-s001.zip › Results of all classifiers/EmbeddingLR/AdaBoost/Test Set/Row-Normalized Confusion Matrix AdaBoost.png]

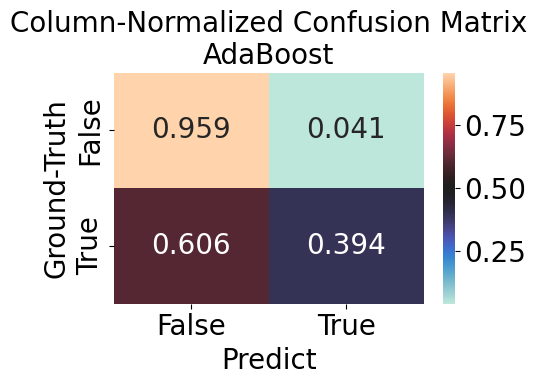

Supplement: Supplementary file 1 [file diagnostics-14-00053-s001.zip › Results of all classifiers/EmbeddingLR/AdaBoost/Train Set/Column-Normalized Confusion Matrix AdaBoost.png]

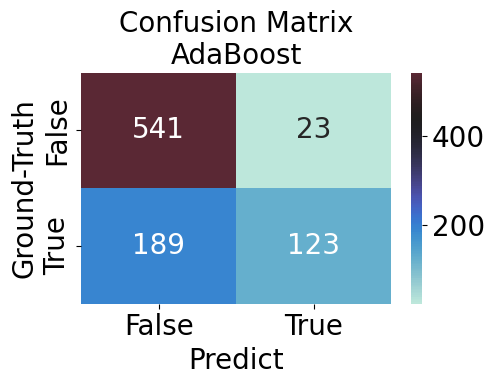

Supplement: Supplementary file 1 [file diagnostics-14-00053-s001.zip › Results of all classifiers/EmbeddingLR/AdaBoost/Train Set/Confusion Matrix AdaBoost.png]

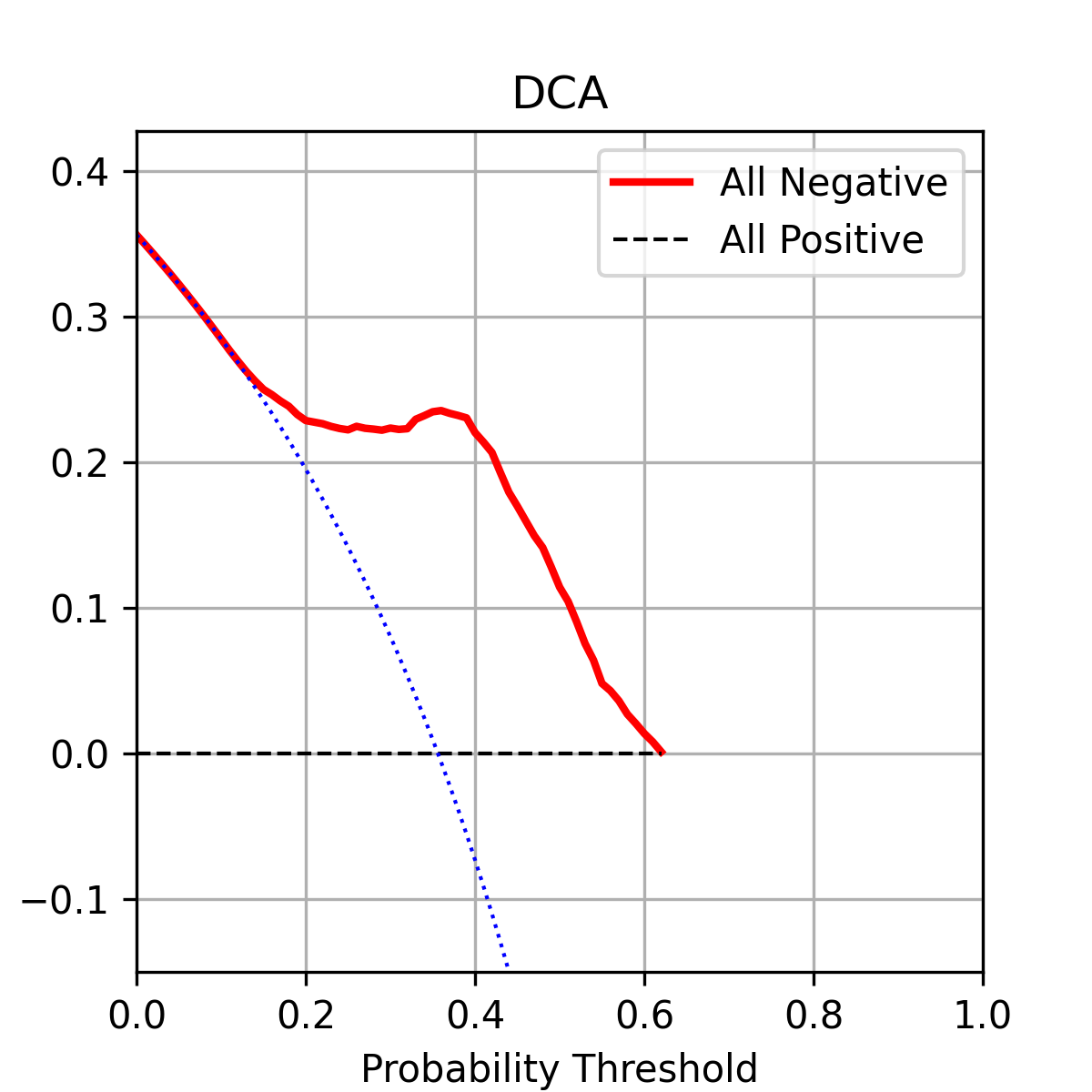

Supplement: Supplementary file 1 [file diagnostics-14-00053-s001.zip › Results of all classifiers/EmbeddingLR/AdaBoost/Train Set/DCA.png]

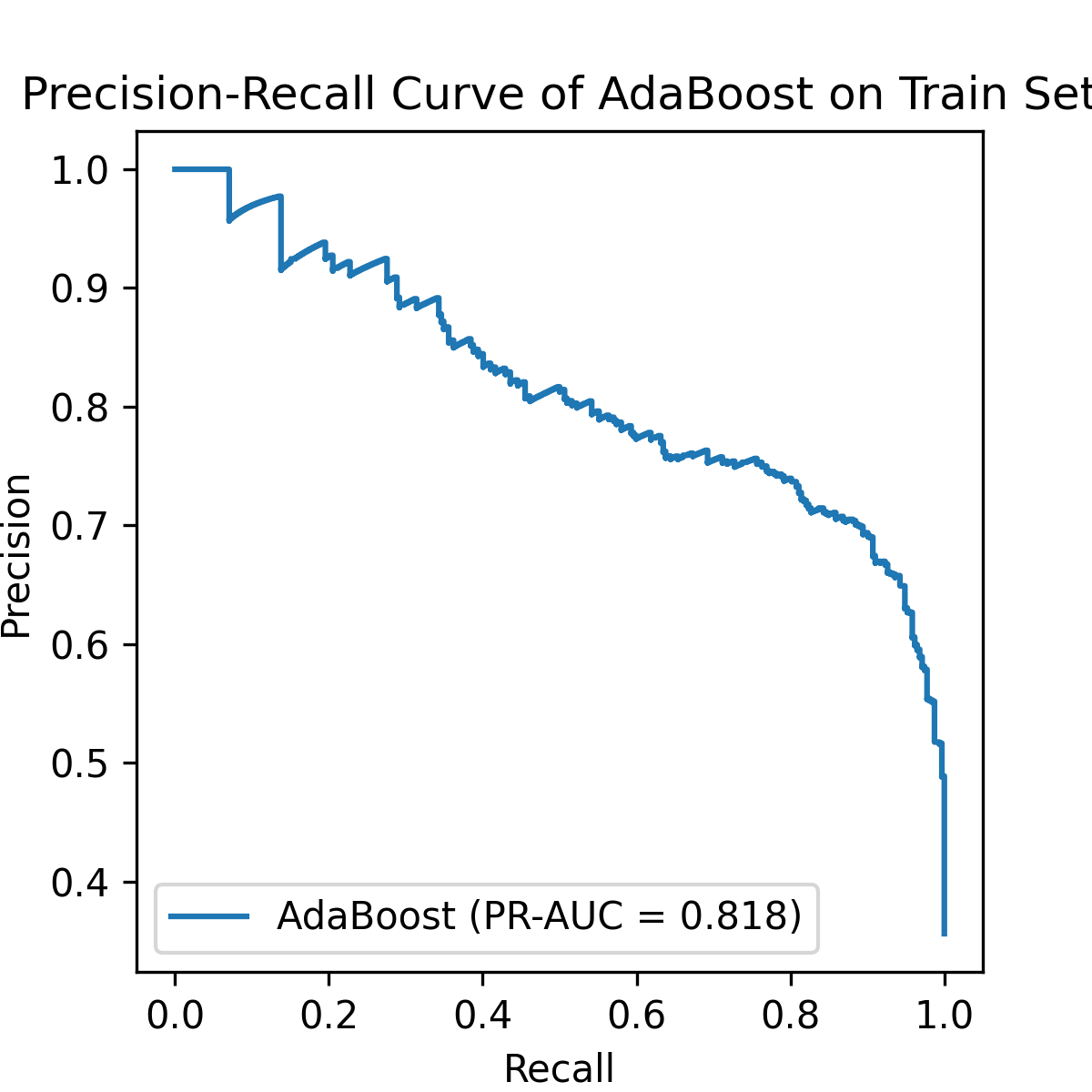

Supplement: Supplementary file 1 [file diagnostics-14-00053-s001.zip › Results of all classifiers/EmbeddingLR/AdaBoost/Train Set/Precision-Recall Curve of AdaBoost on Train Set.png]

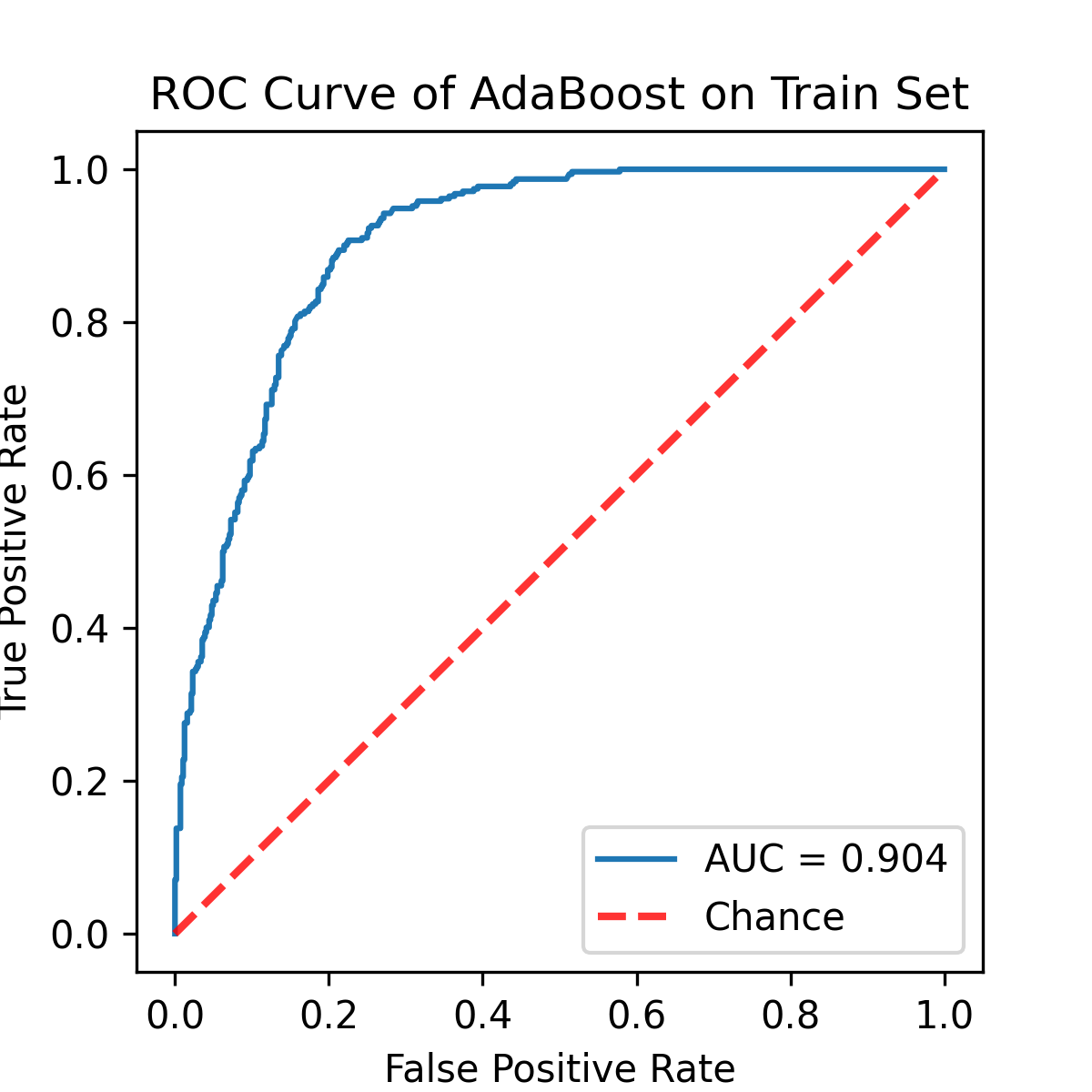

Supplement: Supplementary file 1 [file diagnostics-14-00053-s001.zip › Results of all classifiers/EmbeddingLR/AdaBoost/Train Set/ROC Curve of AdaBoost on Train Set.png]

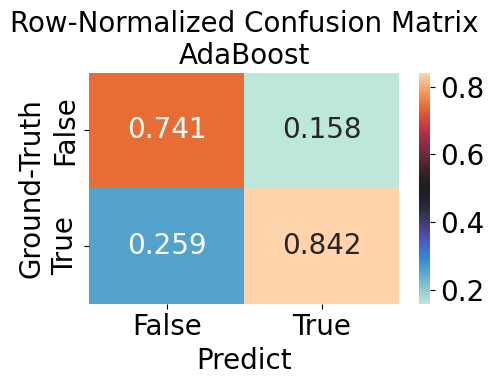

Supplement: Supplementary file 1 [file diagnostics-14-00053-s001.zip › Results of all classifiers/EmbeddingLR/AdaBoost/Train Set/Row-Normalized Confusion Matrix AdaBoost.png]

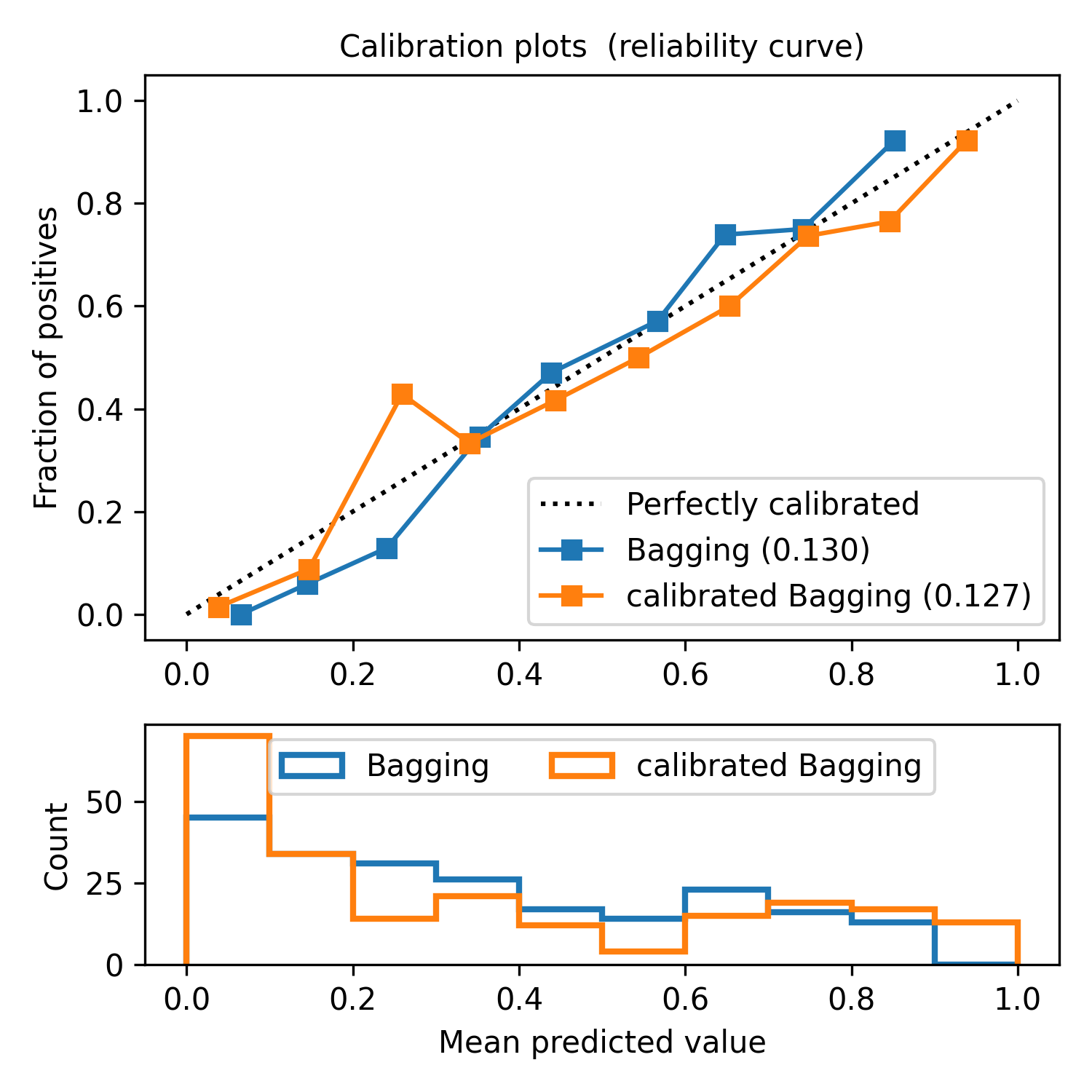

Supplement: Supplementary file 1 [file diagnostics-14-00053-s001.zip › Results of all classifiers/EmbeddingLR/Bagging/Test Set/Calibration plots.png]

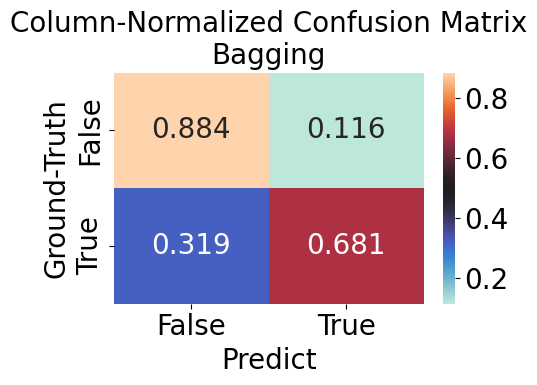

Supplement: Supplementary file 1 [file diagnostics-14-00053-s001.zip › Results of all classifiers/EmbeddingLR/Bagging/Test Set/Column-Normalized Confusion Matrix Bagging.png]

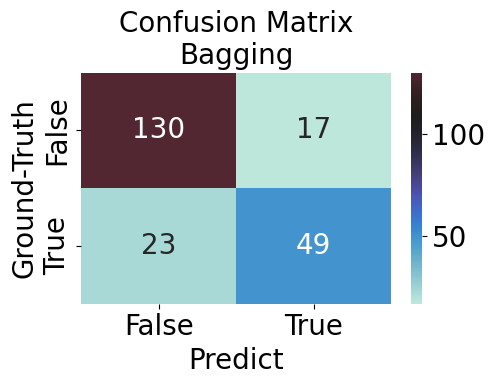

Supplement: Supplementary file 1 [file diagnostics-14-00053-s001.zip › Results of all classifiers/EmbeddingLR/Bagging/Test Set/Confusion Matrix Bagging.png]

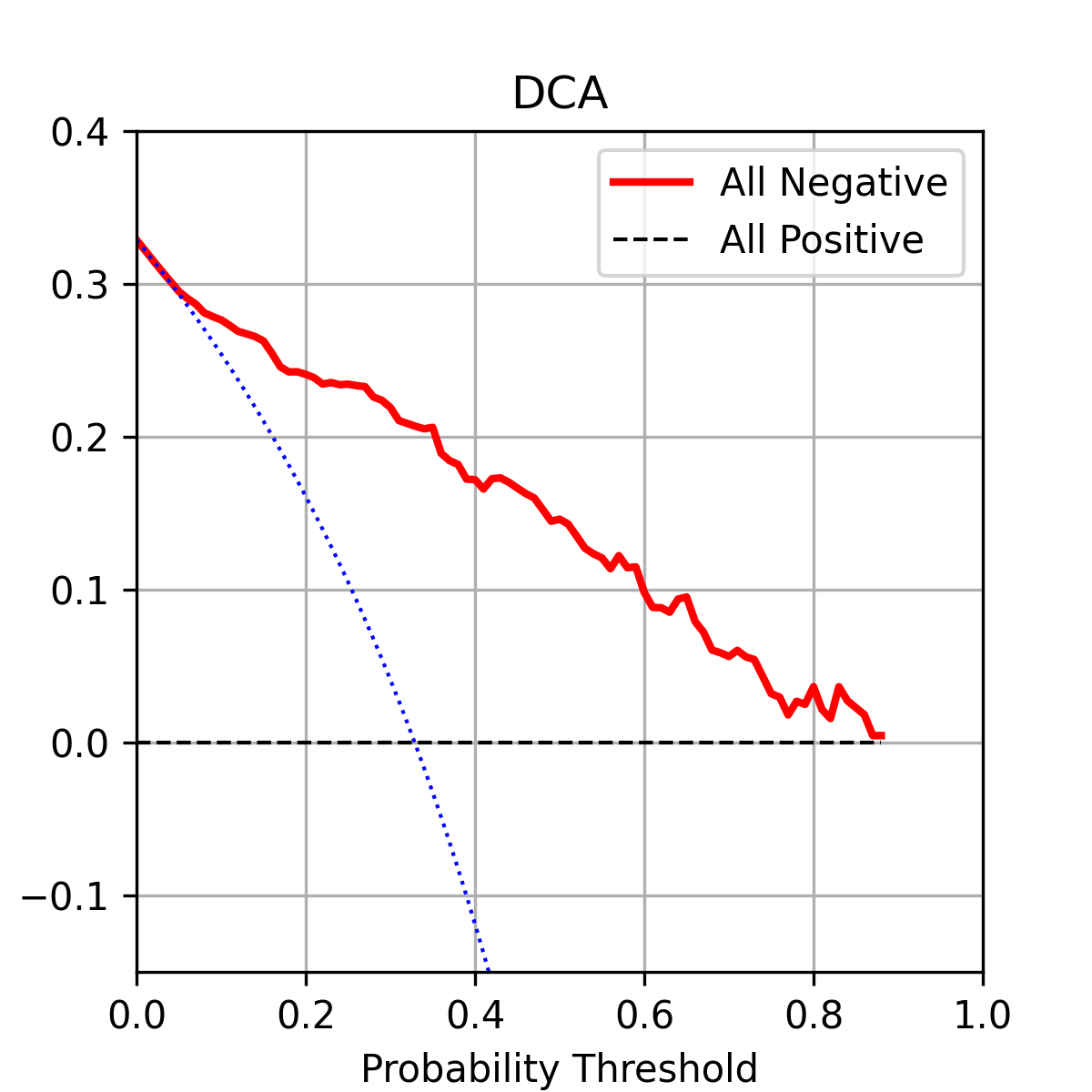

Supplement: Supplementary file 1 [file diagnostics-14-00053-s001.zip › Results of all classifiers/EmbeddingLR/Bagging/Test Set/DCA.png]

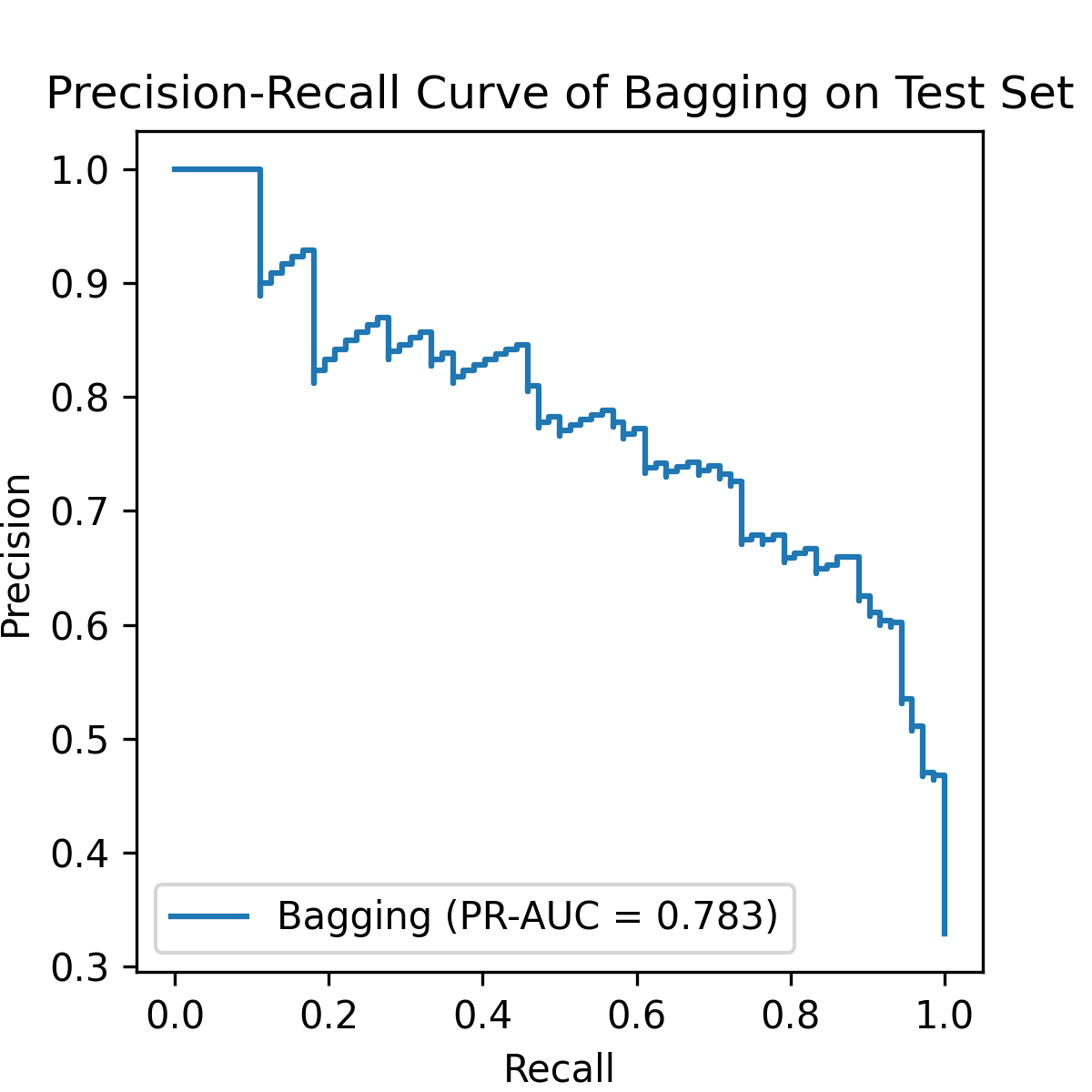

Supplement: Supplementary file 1 [file diagnostics-14-00053-s001.zip › Results of all classifiers/EmbeddingLR/Bagging/Test Set/Precision-Recall Curve of Bagging on Test Set.png]

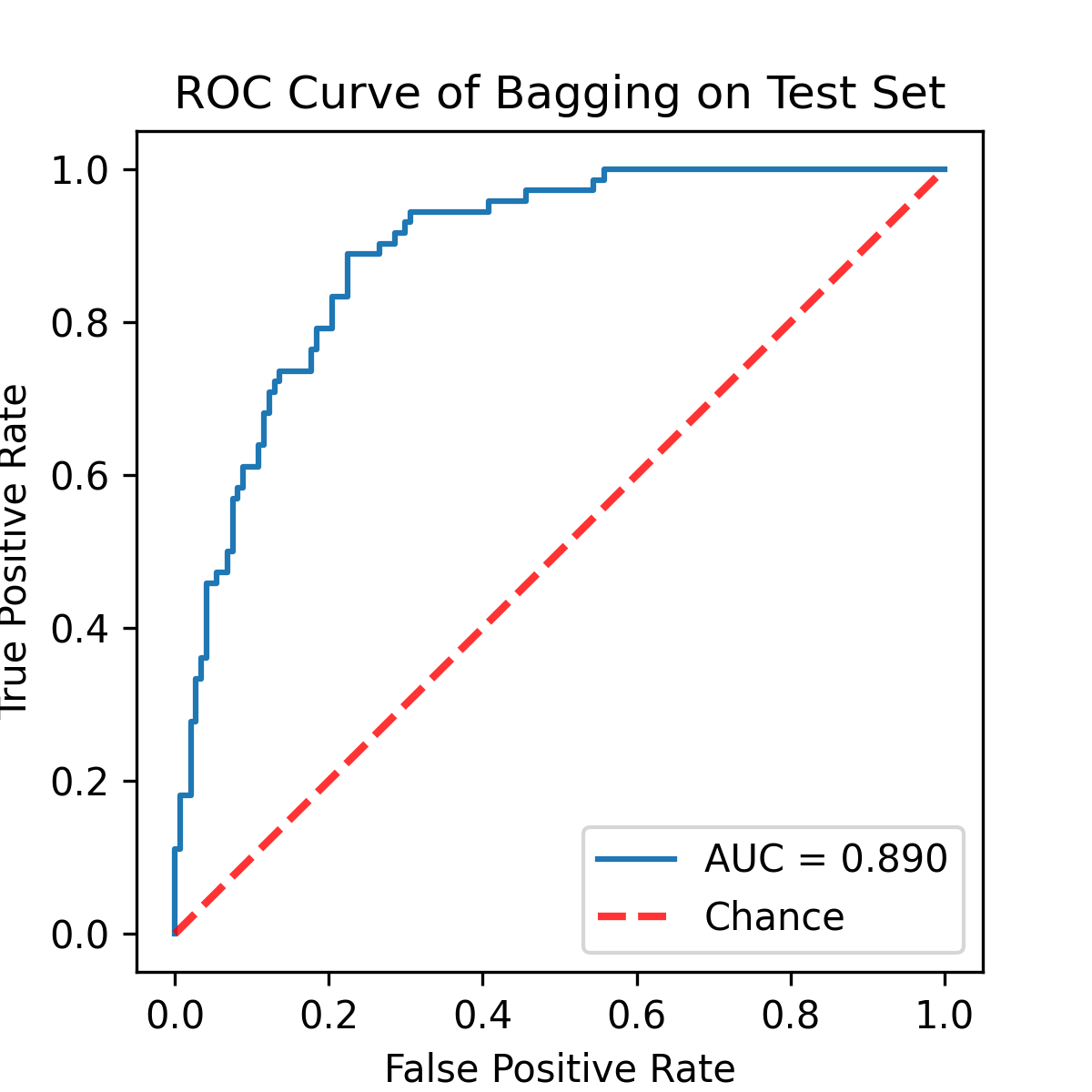

Supplement: Supplementary file 1 [file diagnostics-14-00053-s001.zip › Results of all classifiers/EmbeddingLR/Bagging/Test Set/ROC Curve of Bagging on Test Set.png]

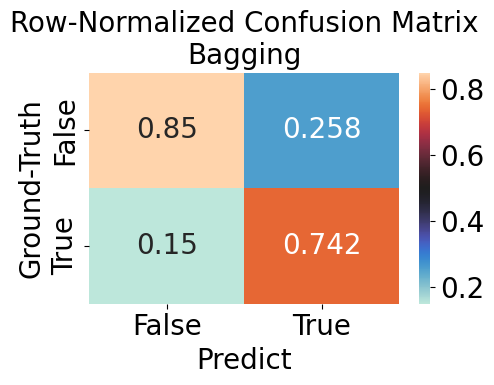

Supplement: Supplementary file 1 [file diagnostics-14-00053-s001.zip › Results of all classifiers/EmbeddingLR/Bagging/Test Set/Row-Normalized Confusion Matrix Bagging.png]

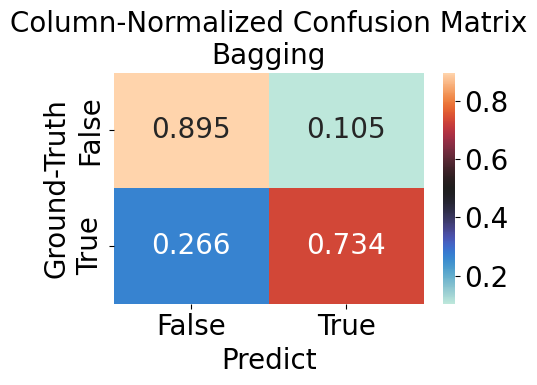

Supplement: Supplementary file 1 [file diagnostics-14-00053-s001.zip › Results of all classifiers/EmbeddingLR/Bagging/Train Set/Column-Normalized Confusion Matrix Bagging.png]

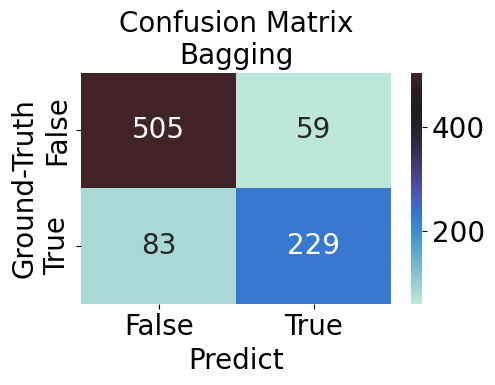

Supplement: Supplementary file 1 [file diagnostics-14-00053-s001.zip › Results of all classifiers/EmbeddingLR/Bagging/Train Set/Confusion Matrix Bagging.png]

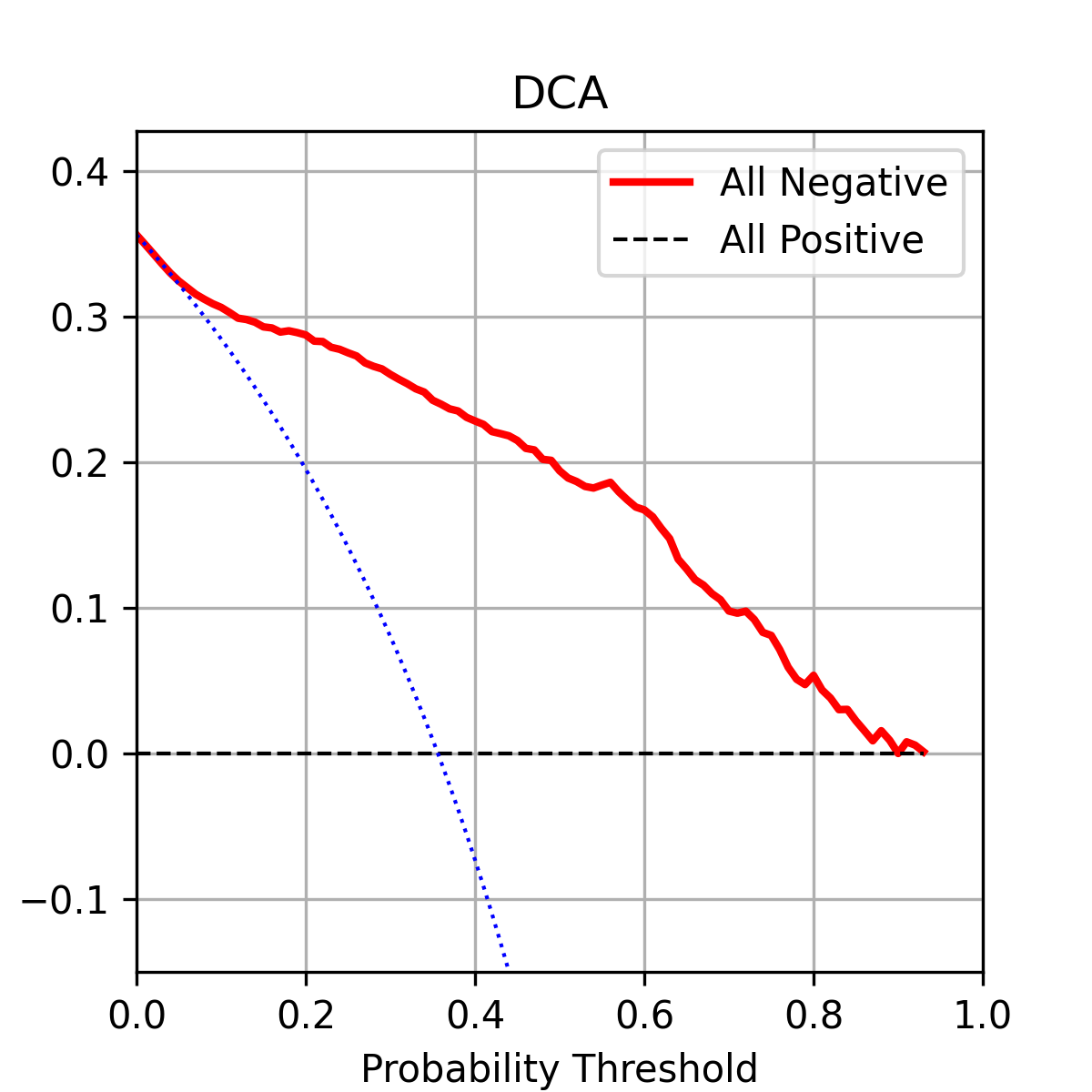

Supplement: Supplementary file 1 [file diagnostics-14-00053-s001.zip › Results of all classifiers/EmbeddingLR/Bagging/Train Set/DCA.png]

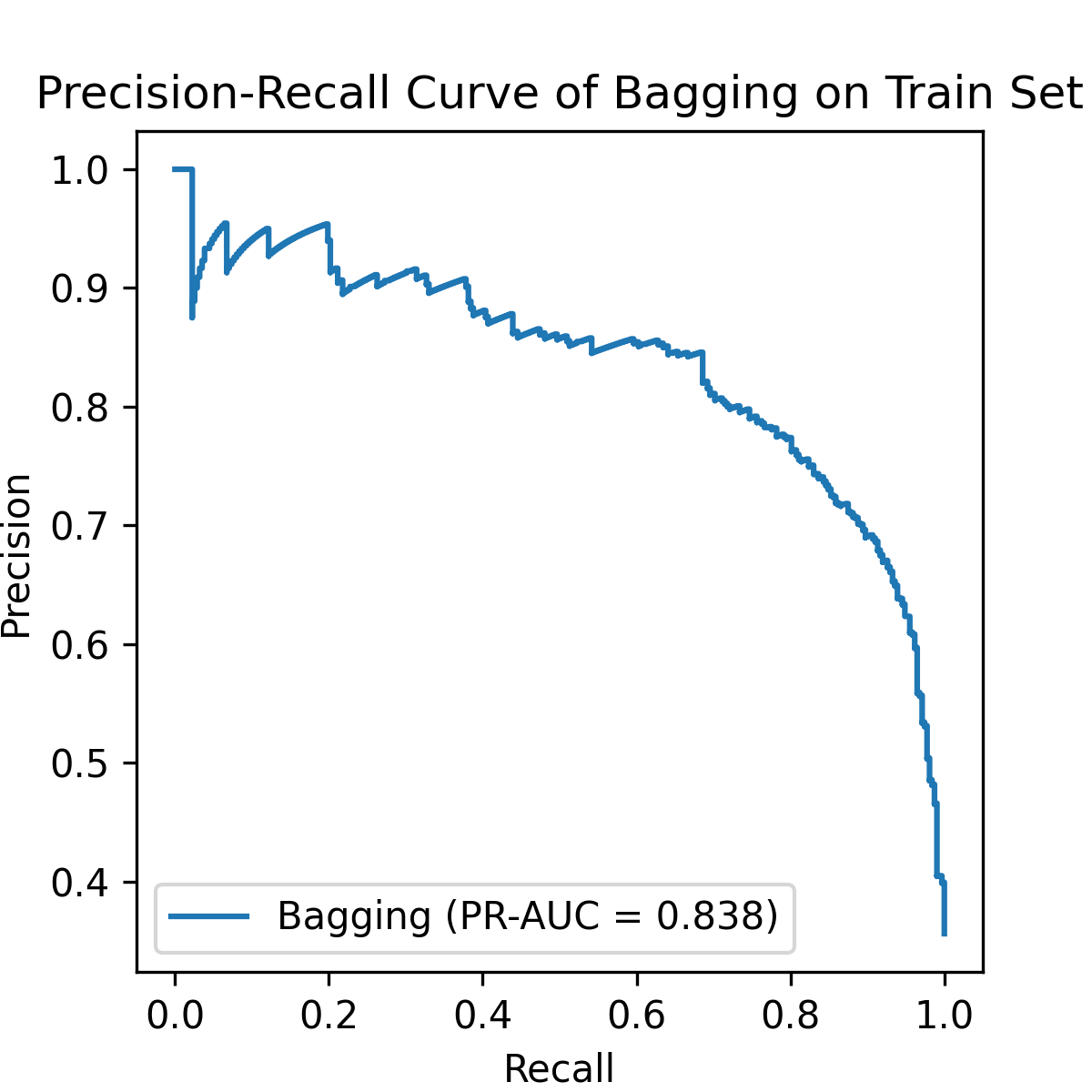

Supplement: Supplementary file 1 [file diagnostics-14-00053-s001.zip › Results of all classifiers/EmbeddingLR/Bagging/Train Set/Precision-Recall Curve of Bagging on Train Set.png]

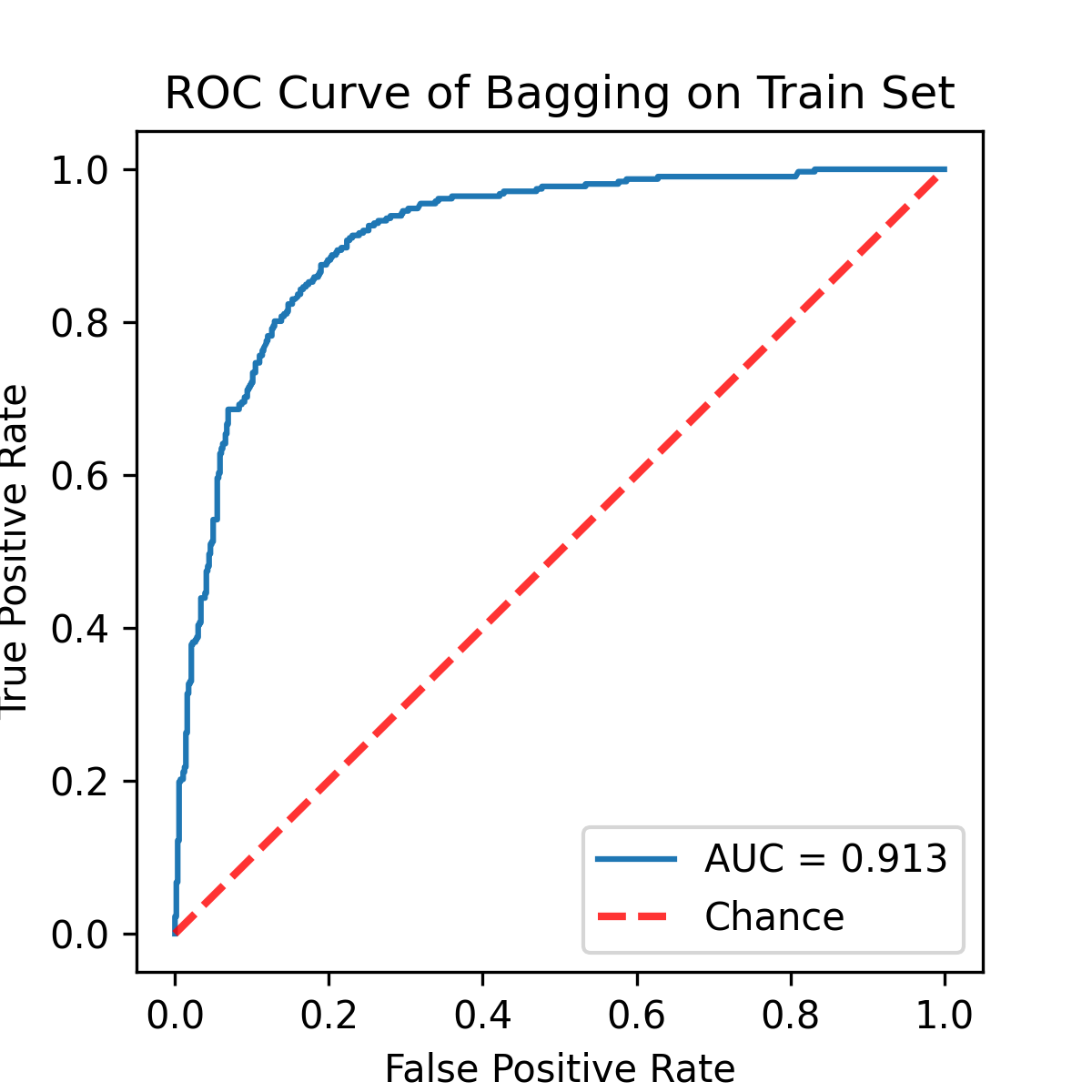

Supplement: Supplementary file 1 [file diagnostics-14-00053-s001.zip › Results of all classifiers/EmbeddingLR/Bagging/Train Set/ROC Curve of Bagging on Train Set.png]

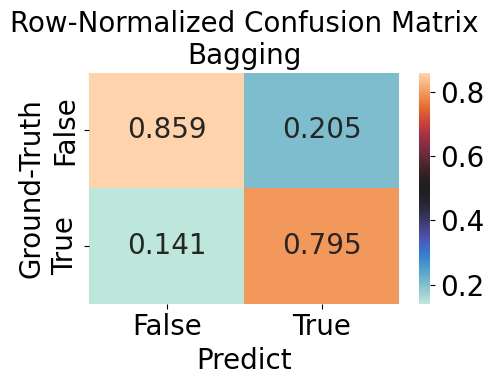

Supplement: Supplementary file 1 [file diagnostics-14-00053-s001.zip › Results of all classifiers/EmbeddingLR/Bagging/Train Set/Row-Normalized Confusion Matrix Bagging.png]

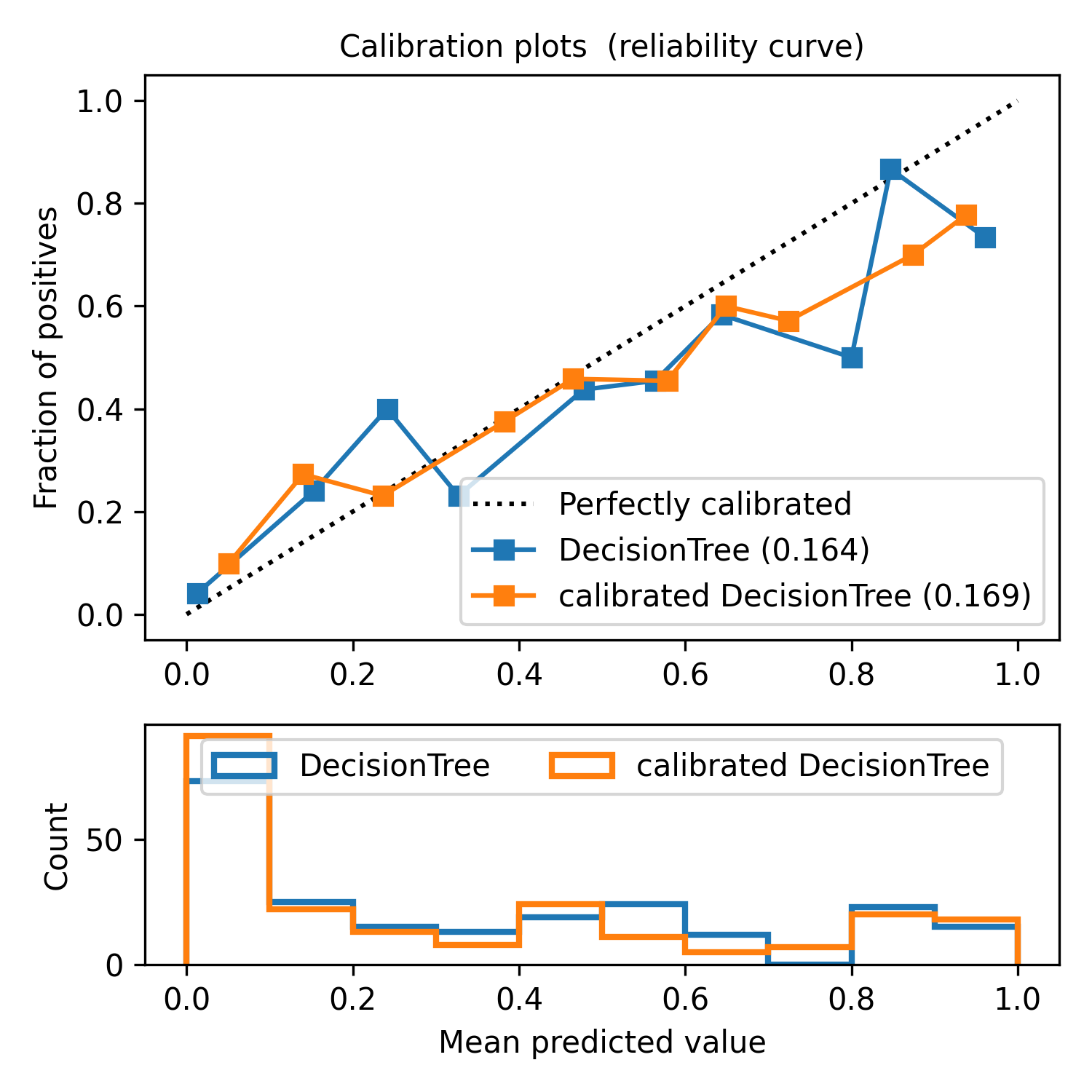

Supplement: Supplementary file 1 [file diagnostics-14-00053-s001.zip › Results of all classifiers/EmbeddingLR/DecisionTree/Test Set/Calibration plots.png]

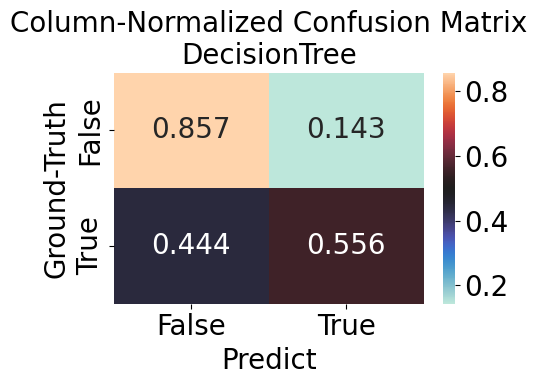

Supplement: Supplementary file 1 [file diagnostics-14-00053-s001.zip › Results of all classifiers/EmbeddingLR/DecisionTree/Test Set/Column-Normalized Confusion Matrix DecisionTree.png]

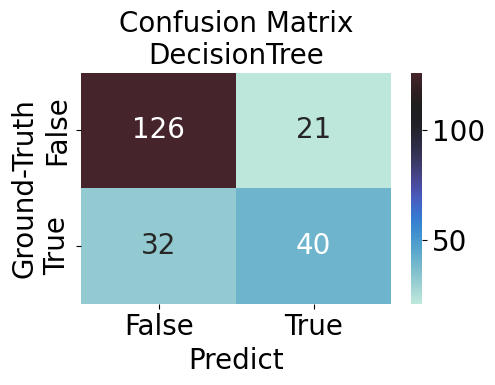

Supplement: Supplementary file 1 [file diagnostics-14-00053-s001.zip › Results of all classifiers/EmbeddingLR/DecisionTree/Test Set/Confusion Matrix DecisionTree.png]

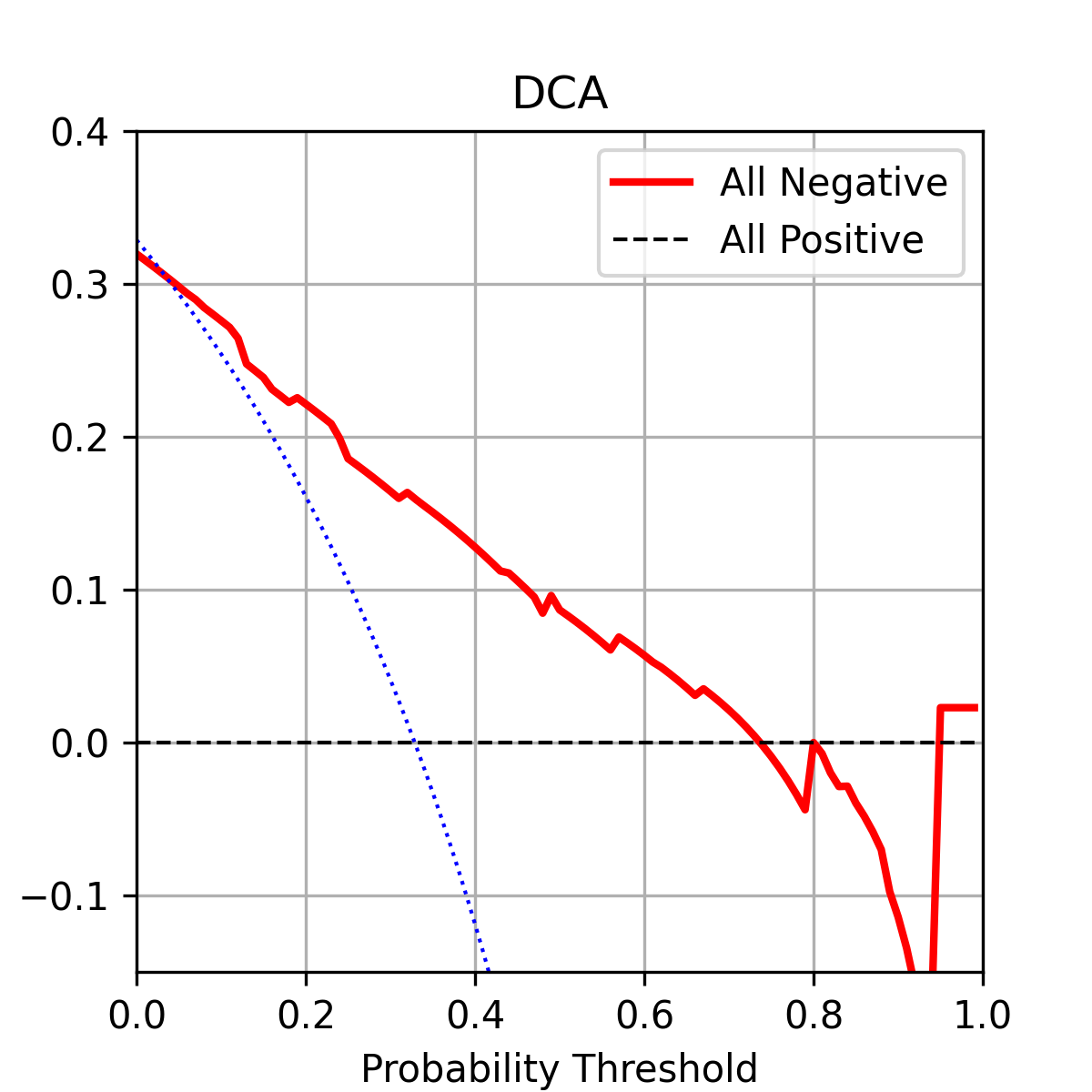

Supplement: Supplementary file 1 [file diagnostics-14-00053-s001.zip › Results of all classifiers/EmbeddingLR/DecisionTree/Test Set/DCA.png]

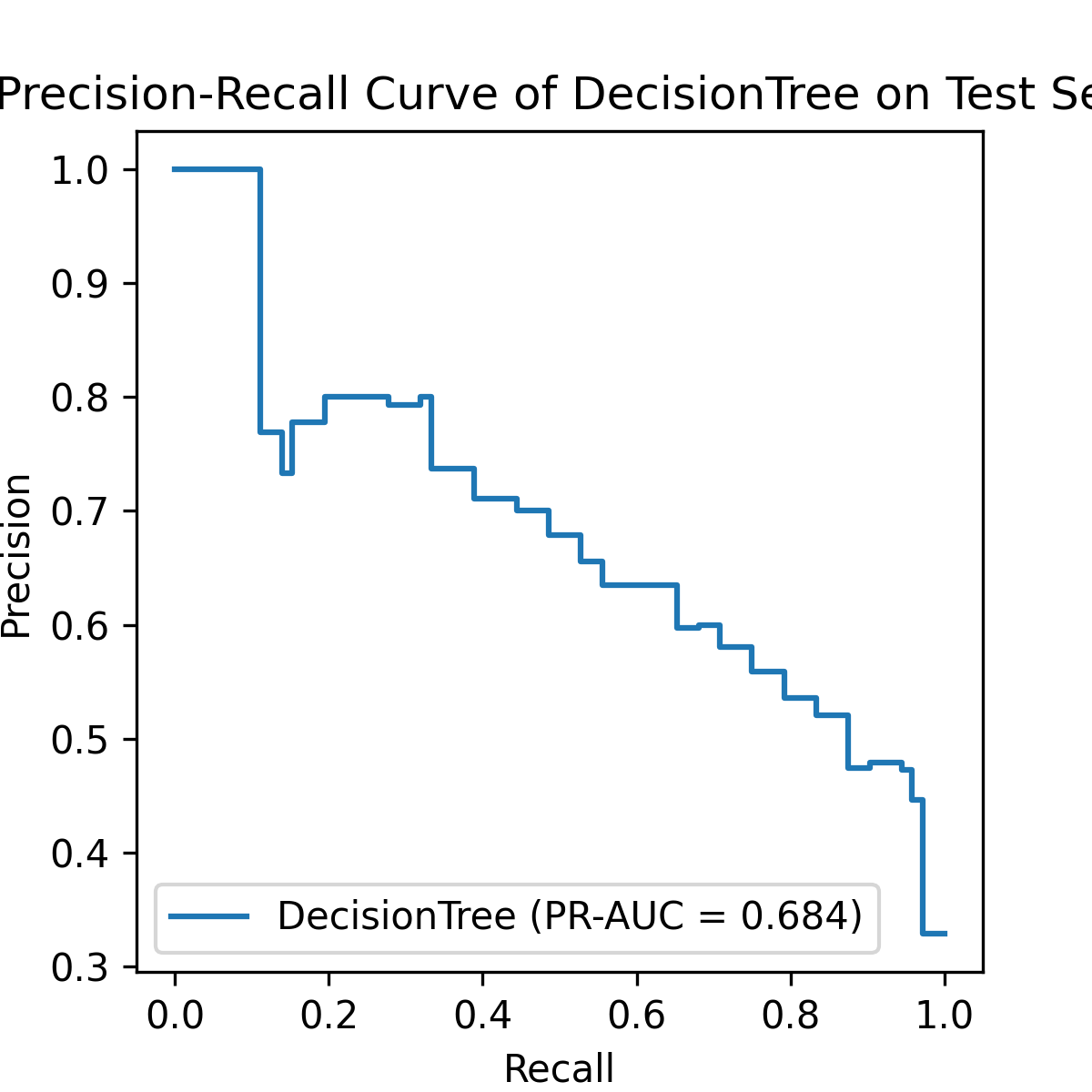

Supplement: Supplementary file 1 [file diagnostics-14-00053-s001.zip › Results of all classifiers/EmbeddingLR/DecisionTree/Test Set/Precision-Recall Curve of DecisionTree on Test Set.png]

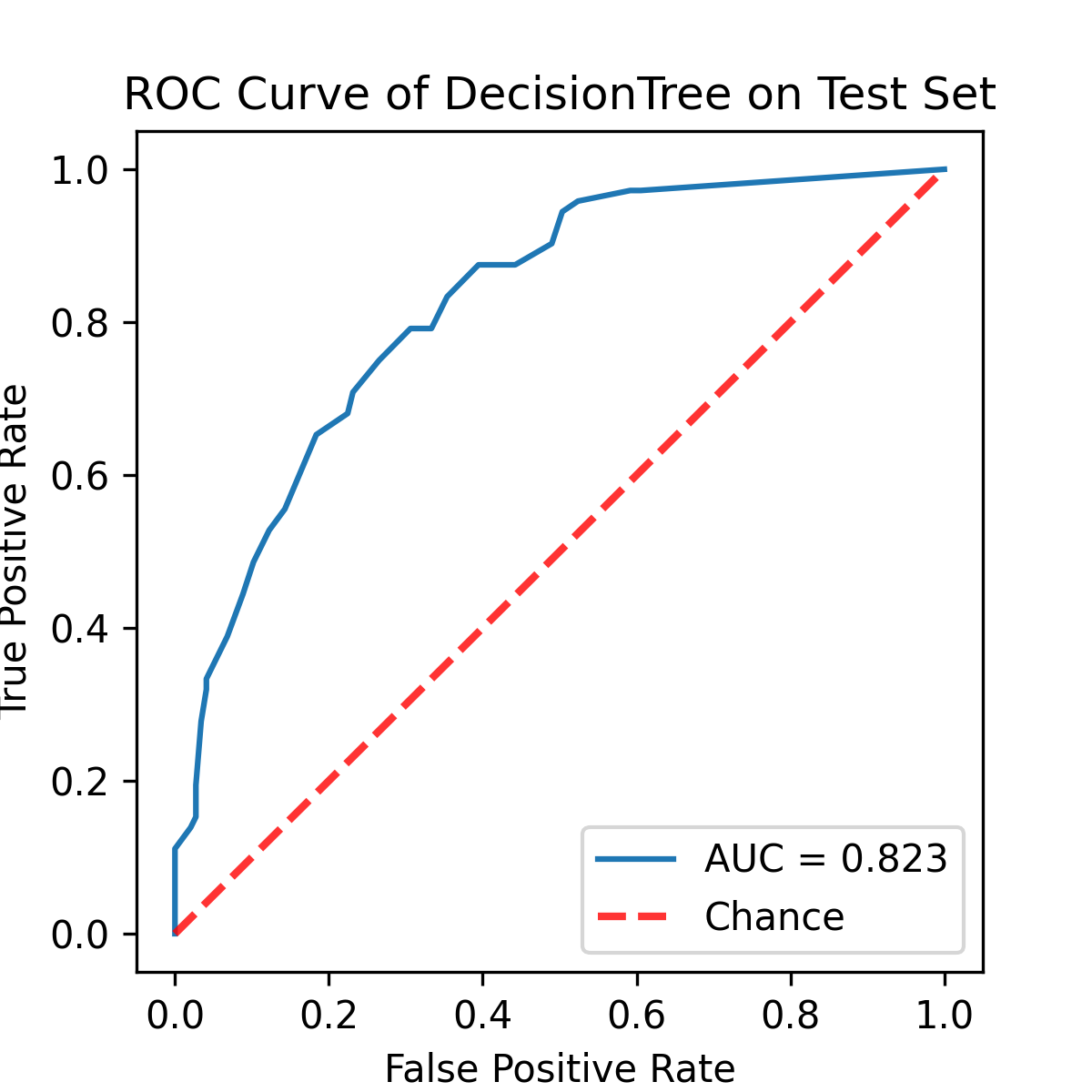

Supplement: Supplementary file 1 [file diagnostics-14-00053-s001.zip › Results of all classifiers/EmbeddingLR/DecisionTree/Test Set/ROC Curve of DecisionTree on Test Set.png]

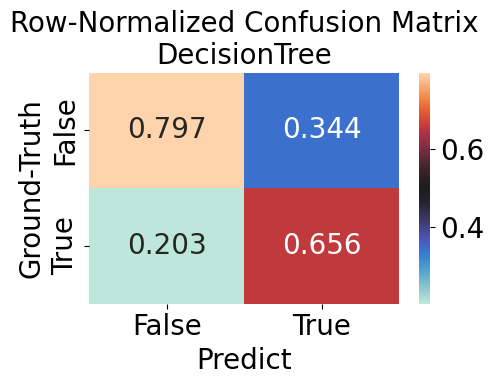

Supplement: Supplementary file 1 [file diagnostics-14-00053-s001.zip › Results of all classifiers/EmbeddingLR/DecisionTree/Test Set/Row-Normalized Confusion Matrix DecisionTree.png]

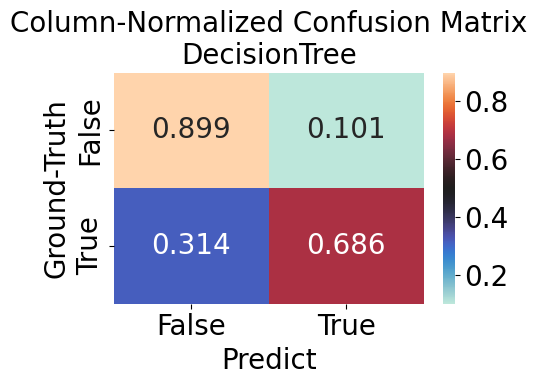

Supplement: Supplementary file 1 [file diagnostics-14-00053-s001.zip › Results of all classifiers/EmbeddingLR/DecisionTree/Train Set/Column-Normalized Confusion Matrix DecisionTree.png]

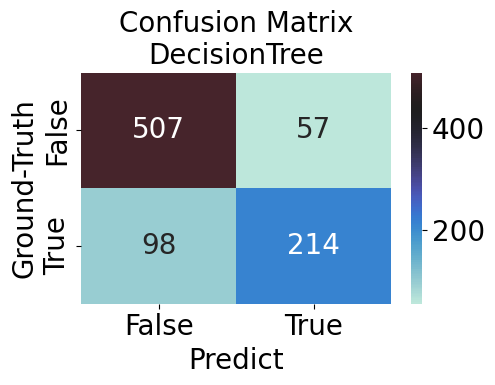

Supplement: Supplementary file 1 [file diagnostics-14-00053-s001.zip › Results of all classifiers/EmbeddingLR/DecisionTree/Train Set/Confusion Matrix DecisionTree.png]

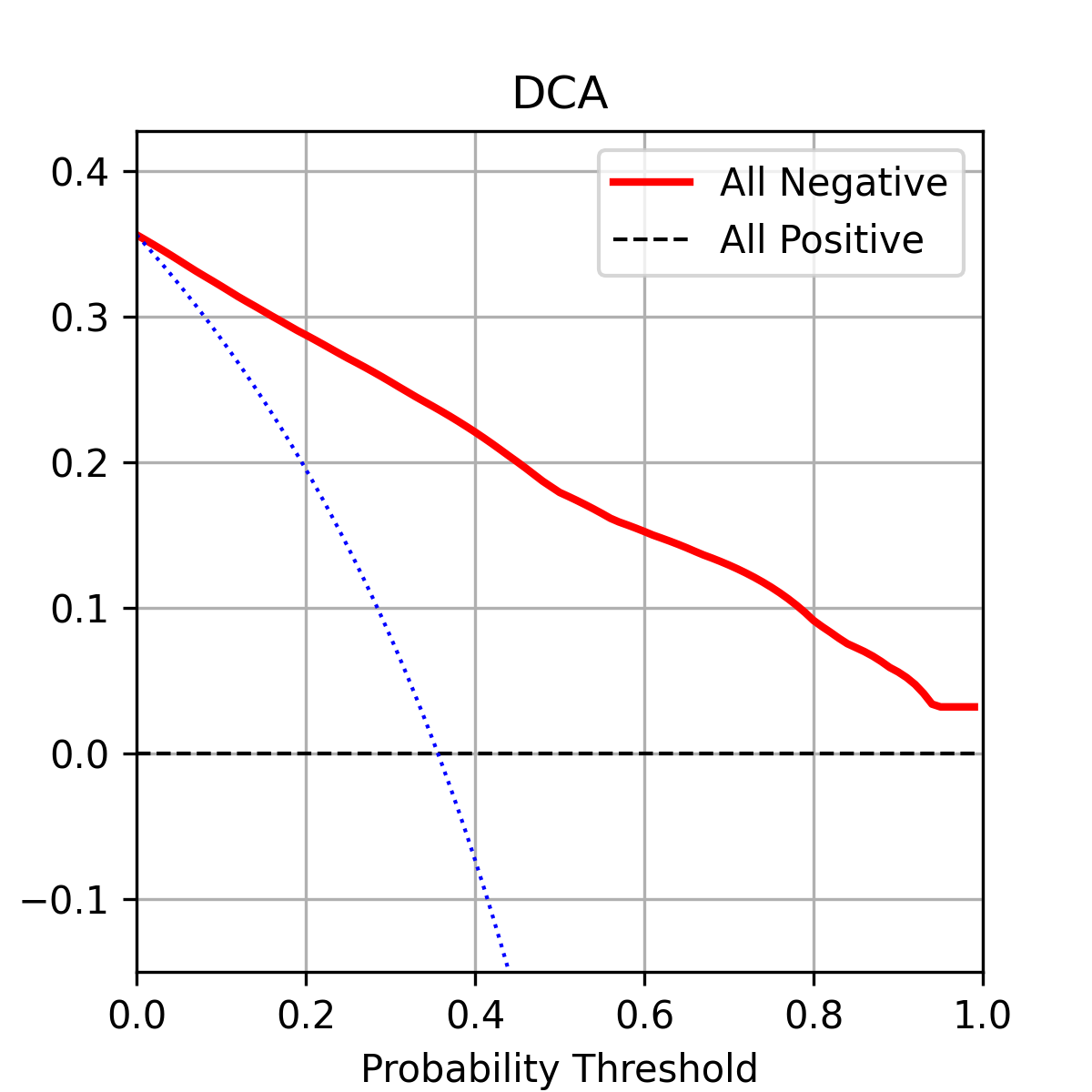

Supplement: Supplementary file 1 [file diagnostics-14-00053-s001.zip › Results of all classifiers/EmbeddingLR/DecisionTree/Train Set/DCA.png]

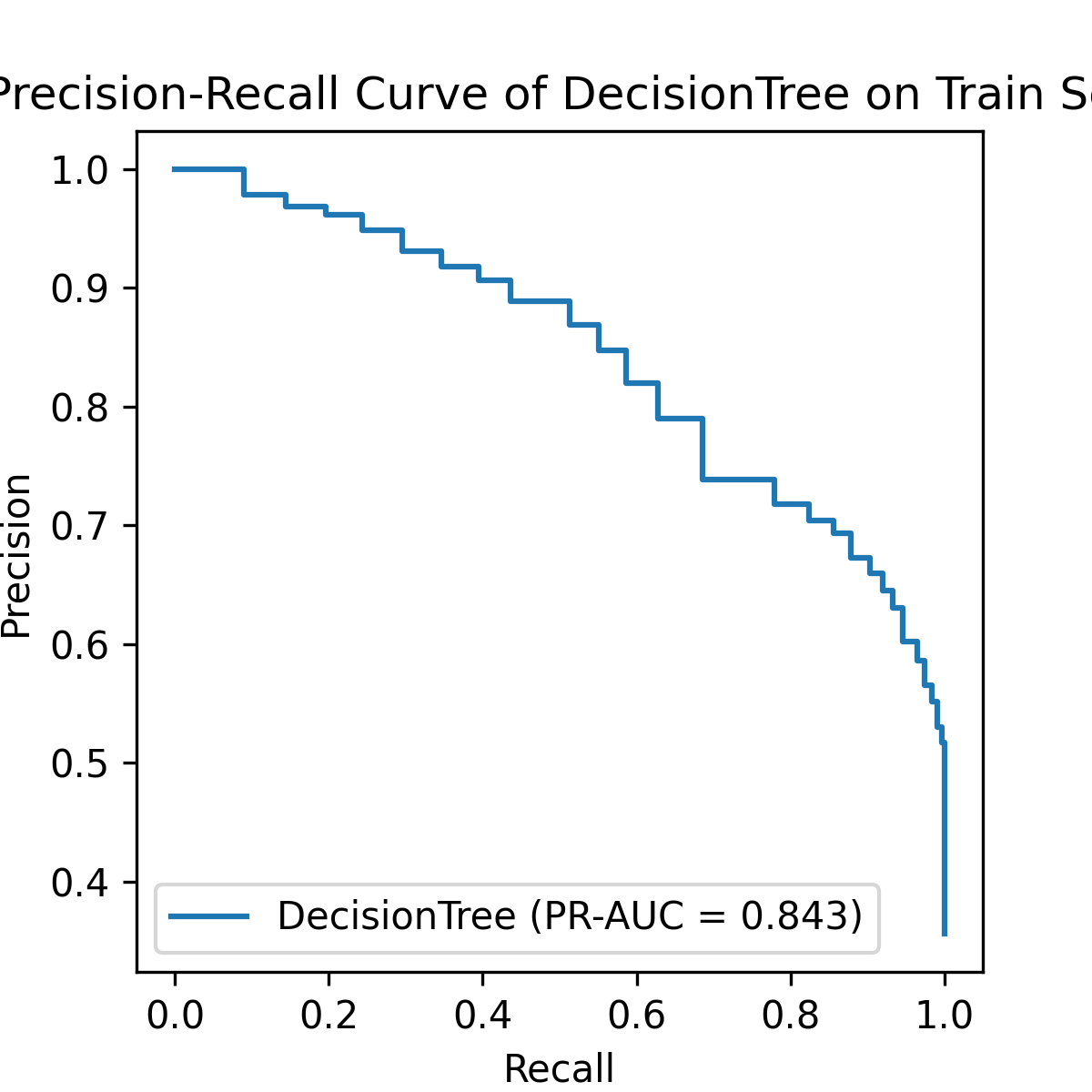

Supplement: Supplementary file 1 [file diagnostics-14-00053-s001.zip › Results of all classifiers/EmbeddingLR/DecisionTree/Train Set/Precision-Recall Curve of DecisionTree on Train Set.png]

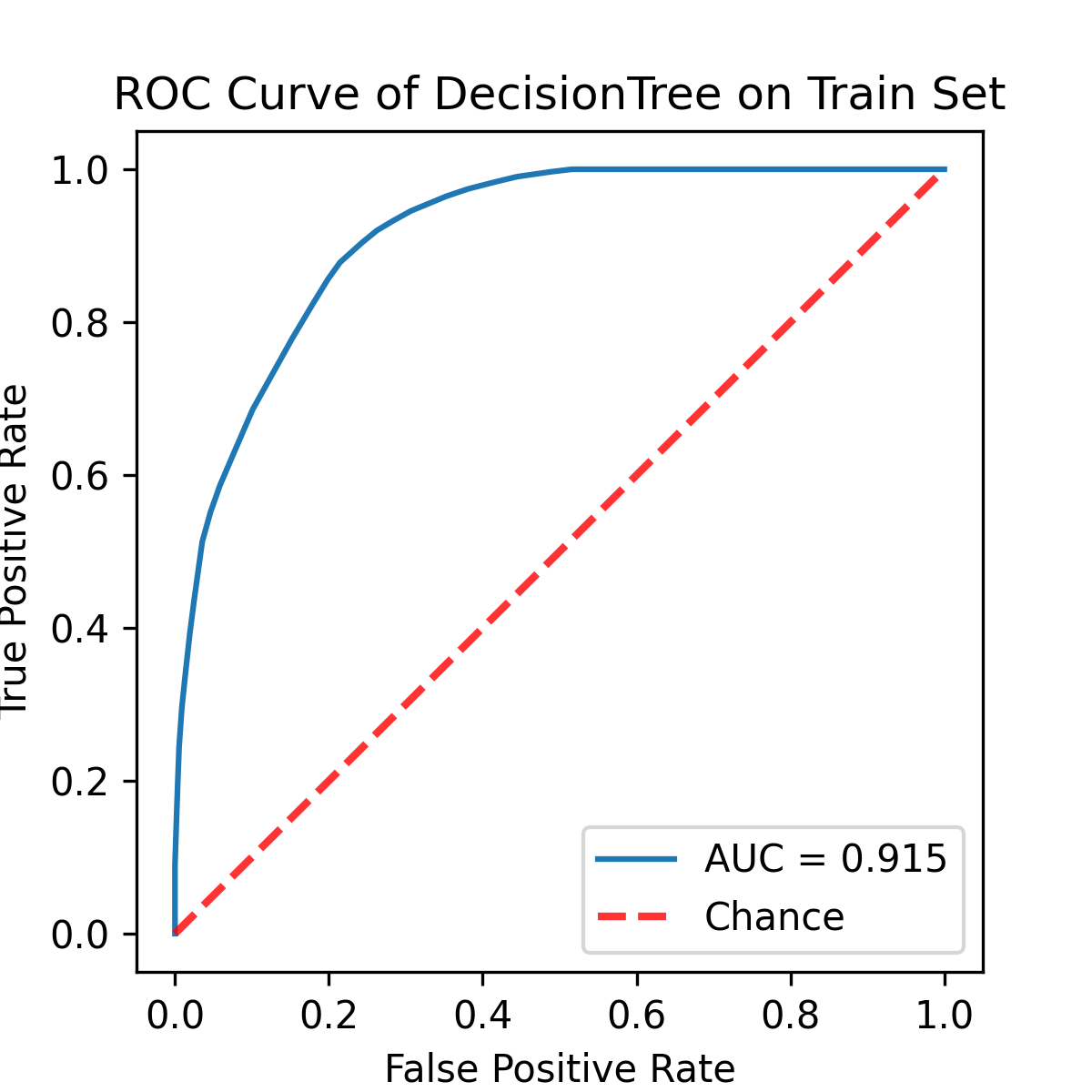

Supplement: Supplementary file 1 [file diagnostics-14-00053-s001.zip › Results of all classifiers/EmbeddingLR/DecisionTree/Train Set/ROC Curve of DecisionTree on Train Set.png]

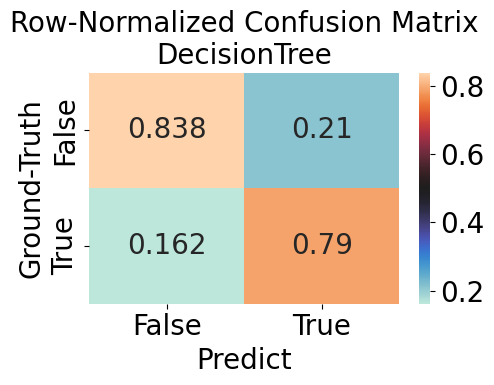

Supplement: Supplementary file 1 [file diagnostics-14-00053-s001.zip › Results of all classifiers/EmbeddingLR/DecisionTree/Train Set/Row-Normalized Confusion Matrix DecisionTree.png]

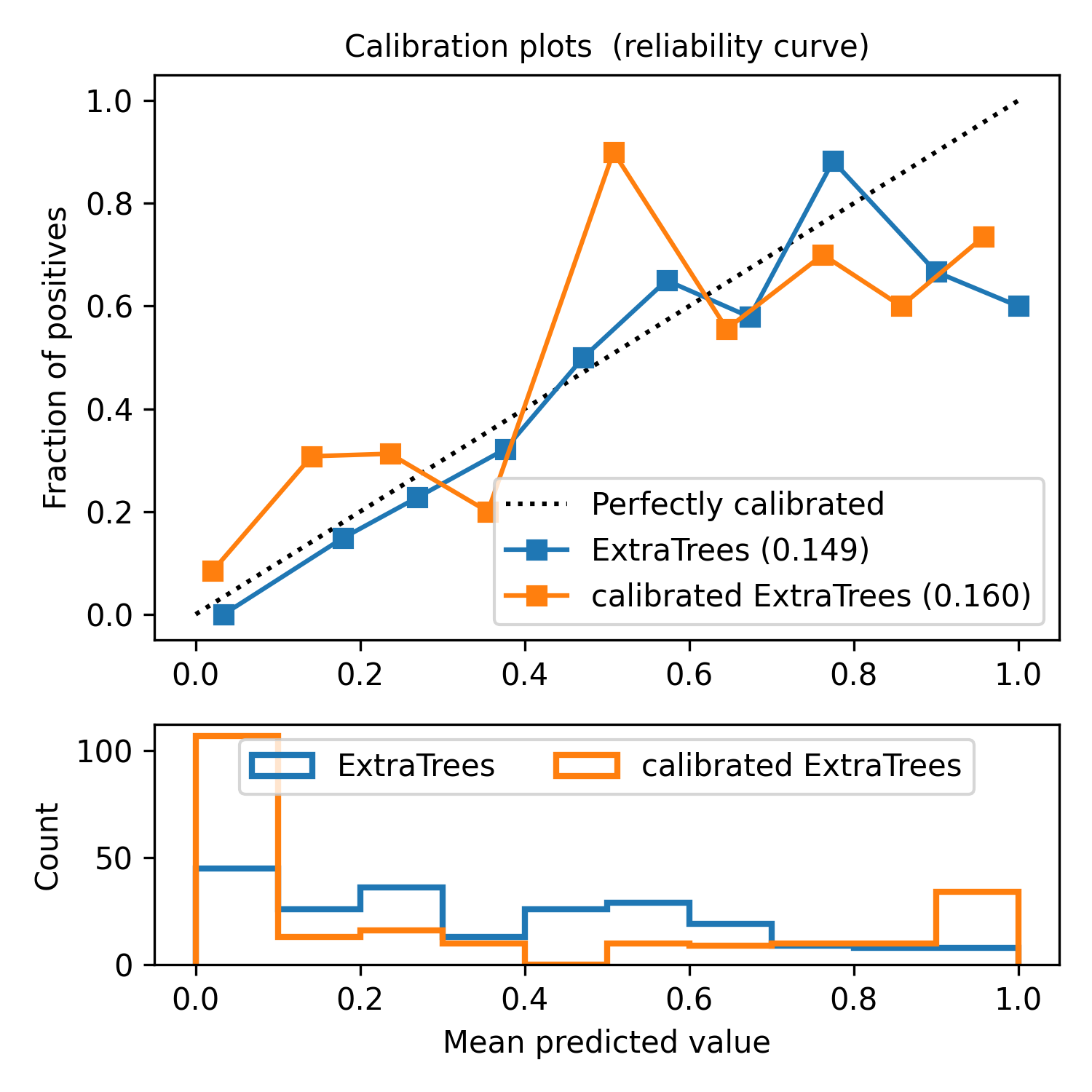

Supplement: Supplementary file 1 [file diagnostics-14-00053-s001.zip › Results of all classifiers/EmbeddingLR/ExtraTrees/Test Set/Calibration plots.png]

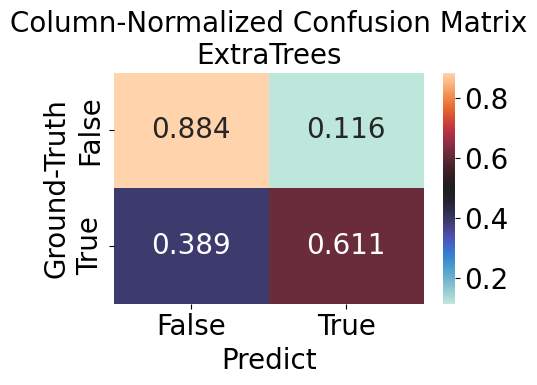

Supplement: Supplementary file 1 [file diagnostics-14-00053-s001.zip › Results of all classifiers/EmbeddingLR/ExtraTrees/Test Set/Column-Normalized Confusion Matrix ExtraTrees.png]

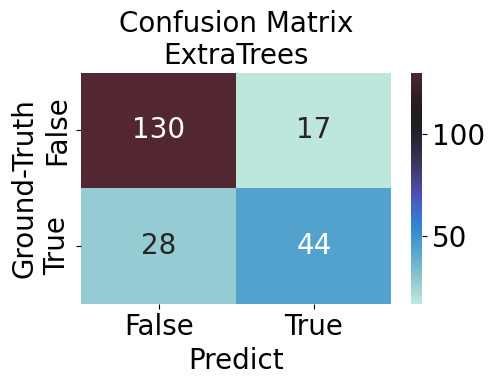

Supplement: Supplementary file 1 [file diagnostics-14-00053-s001.zip › Results of all classifiers/EmbeddingLR/ExtraTrees/Test Set/Confusion Matrix ExtraTrees.png]

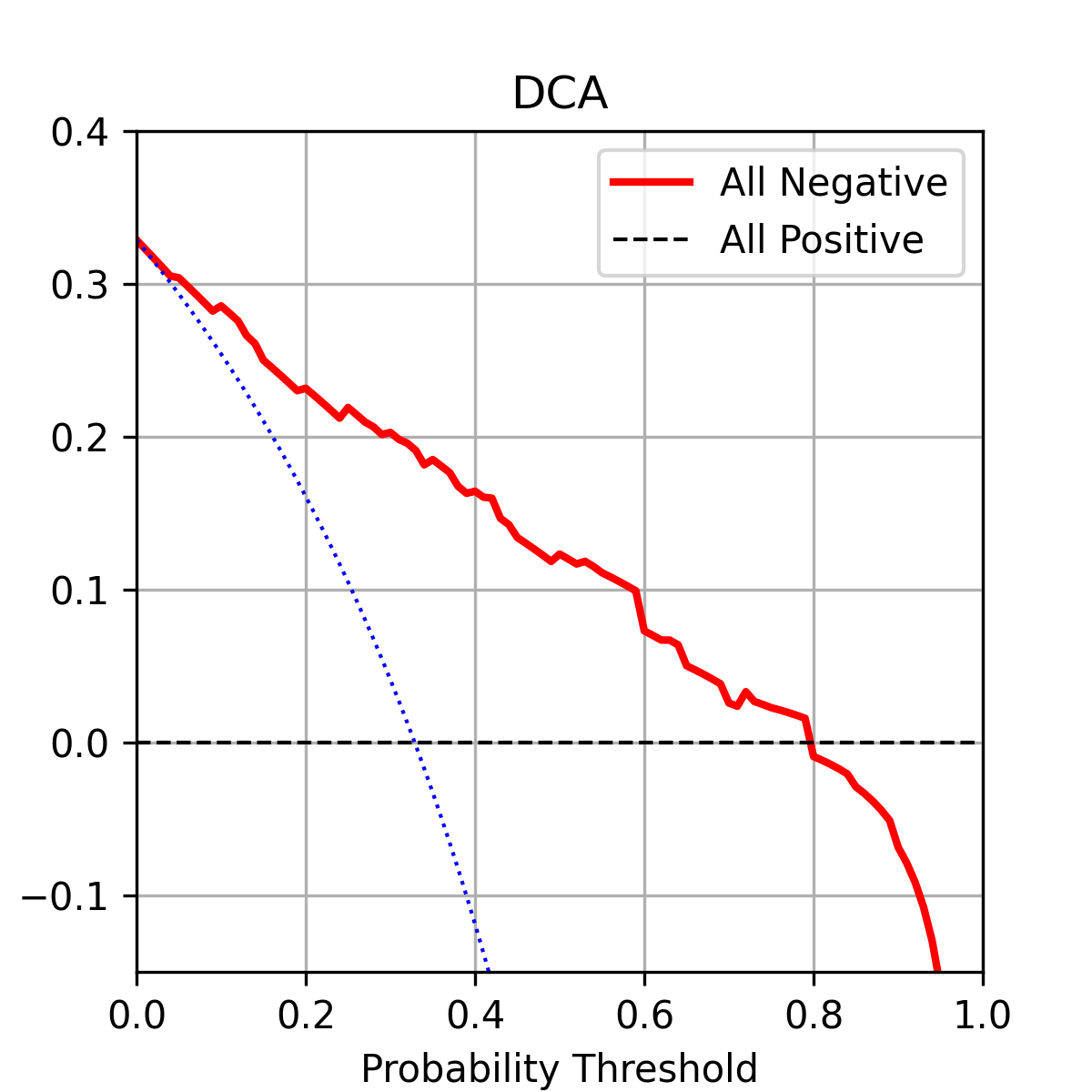

Supplement: Supplementary file 1 [file diagnostics-14-00053-s001.zip › Results of all classifiers/EmbeddingLR/ExtraTrees/Test Set/DCA.png]

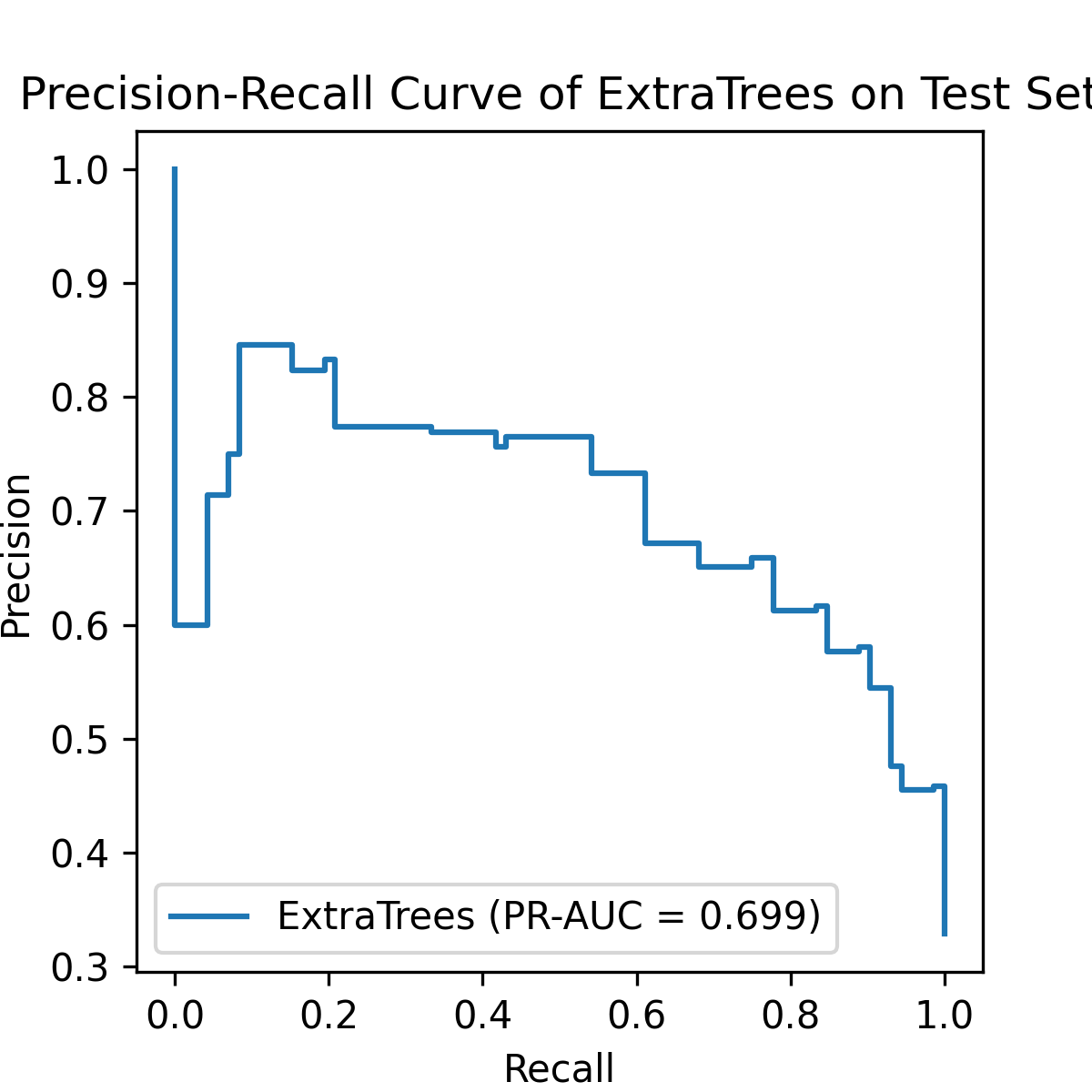

Supplement: Supplementary file 1 [file diagnostics-14-00053-s001.zip › Results of all classifiers/EmbeddingLR/ExtraTrees/Test Set/Precision-Recall Curve of ExtraTrees on Test Set.png]

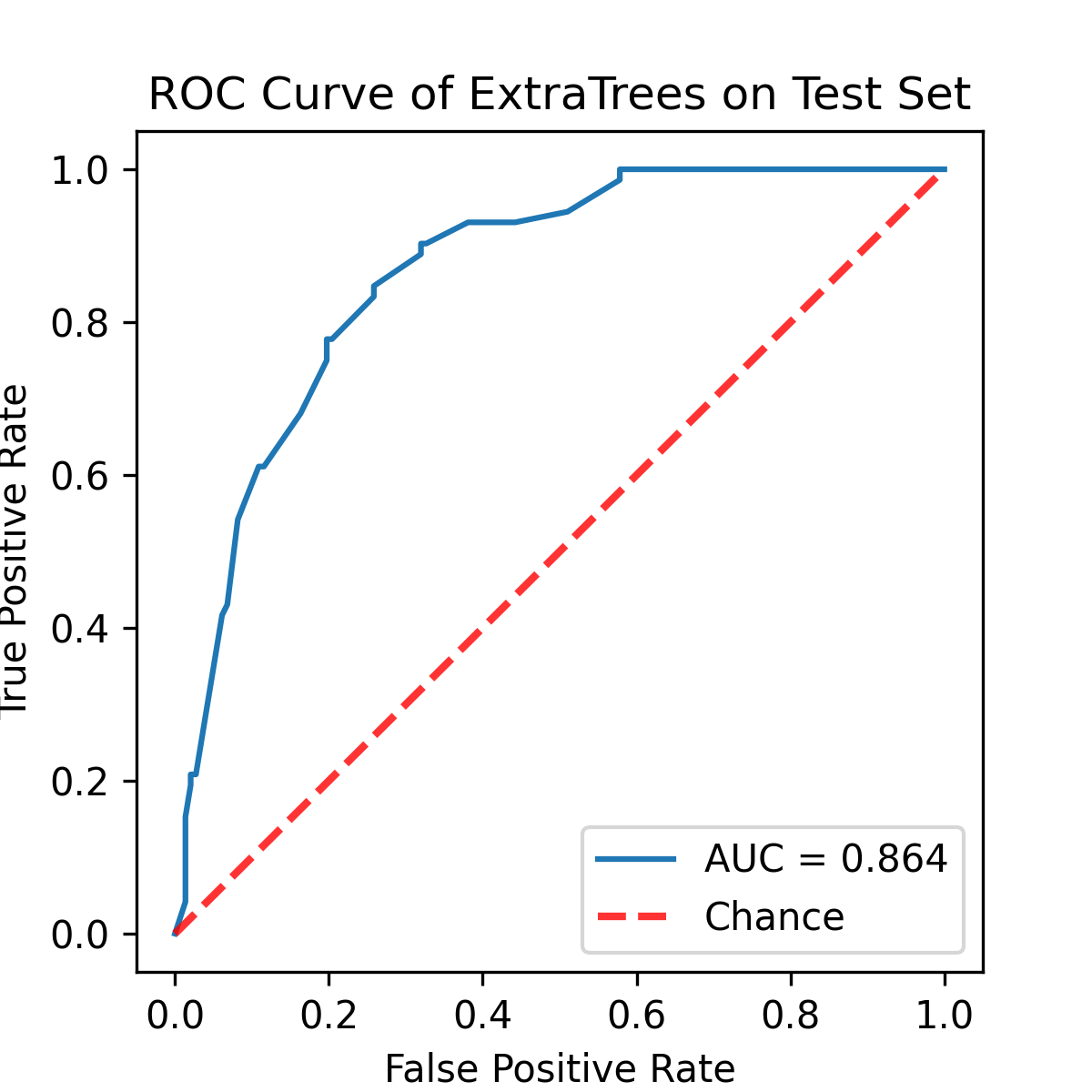

Supplement: Supplementary file 1 [file diagnostics-14-00053-s001.zip › Results of all classifiers/EmbeddingLR/ExtraTrees/Test Set/ROC Curve of ExtraTrees on Test Set.png]

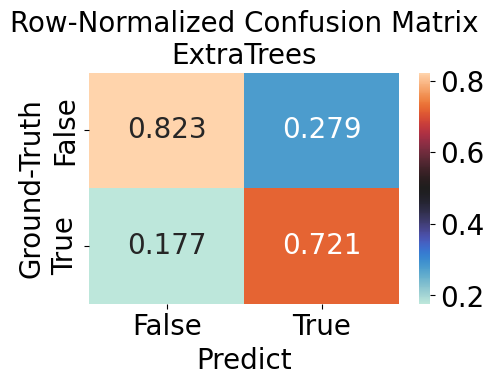

Supplement: Supplementary file 1 [file diagnostics-14-00053-s001.zip › Results of all classifiers/EmbeddingLR/ExtraTrees/Test Set/Row-Normalized Confusion Matrix ExtraTrees.png]

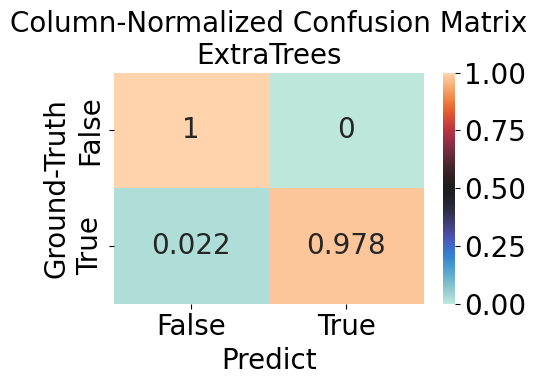

Supplement: Supplementary file 1 [file diagnostics-14-00053-s001.zip › Results of all classifiers/EmbeddingLR/ExtraTrees/Train Set/Column-Normalized Confusion Matrix ExtraTrees.png]

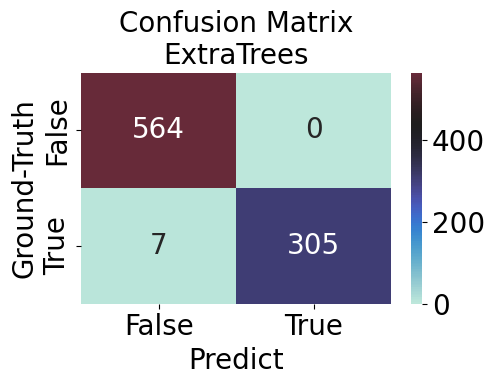

Supplement: Supplementary file 1 [file diagnostics-14-00053-s001.zip › Results of all classifiers/EmbeddingLR/ExtraTrees/Train Set/Confusion Matrix ExtraTrees.png]

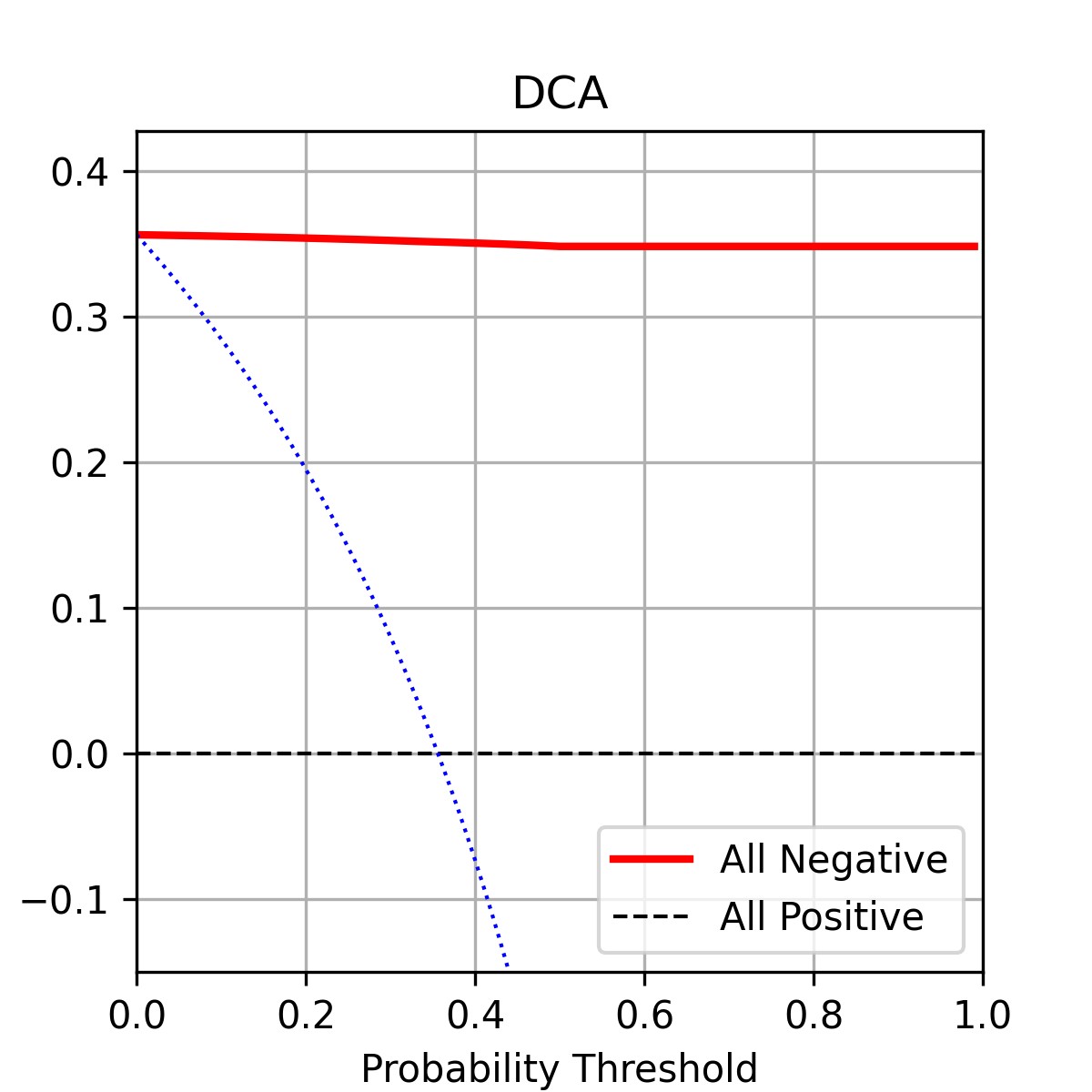

Supplement: Supplementary file 1 [file diagnostics-14-00053-s001.zip › Results of all classifiers/EmbeddingLR/ExtraTrees/Train Set/DCA.png]

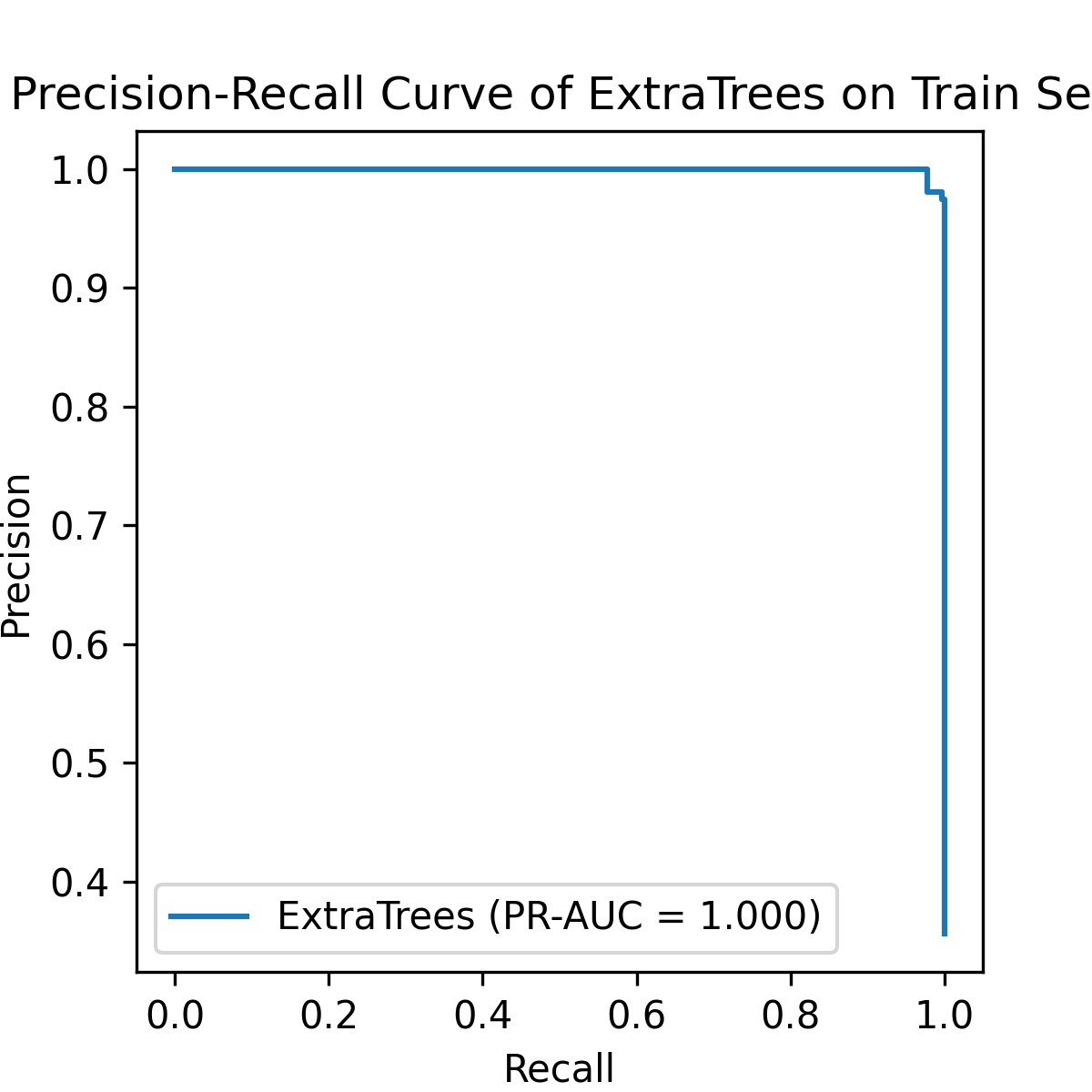

Supplement: Supplementary file 1 [file diagnostics-14-00053-s001.zip › Results of all classifiers/EmbeddingLR/ExtraTrees/Train Set/Precision-Recall Curve of ExtraTrees on Train Set.png]

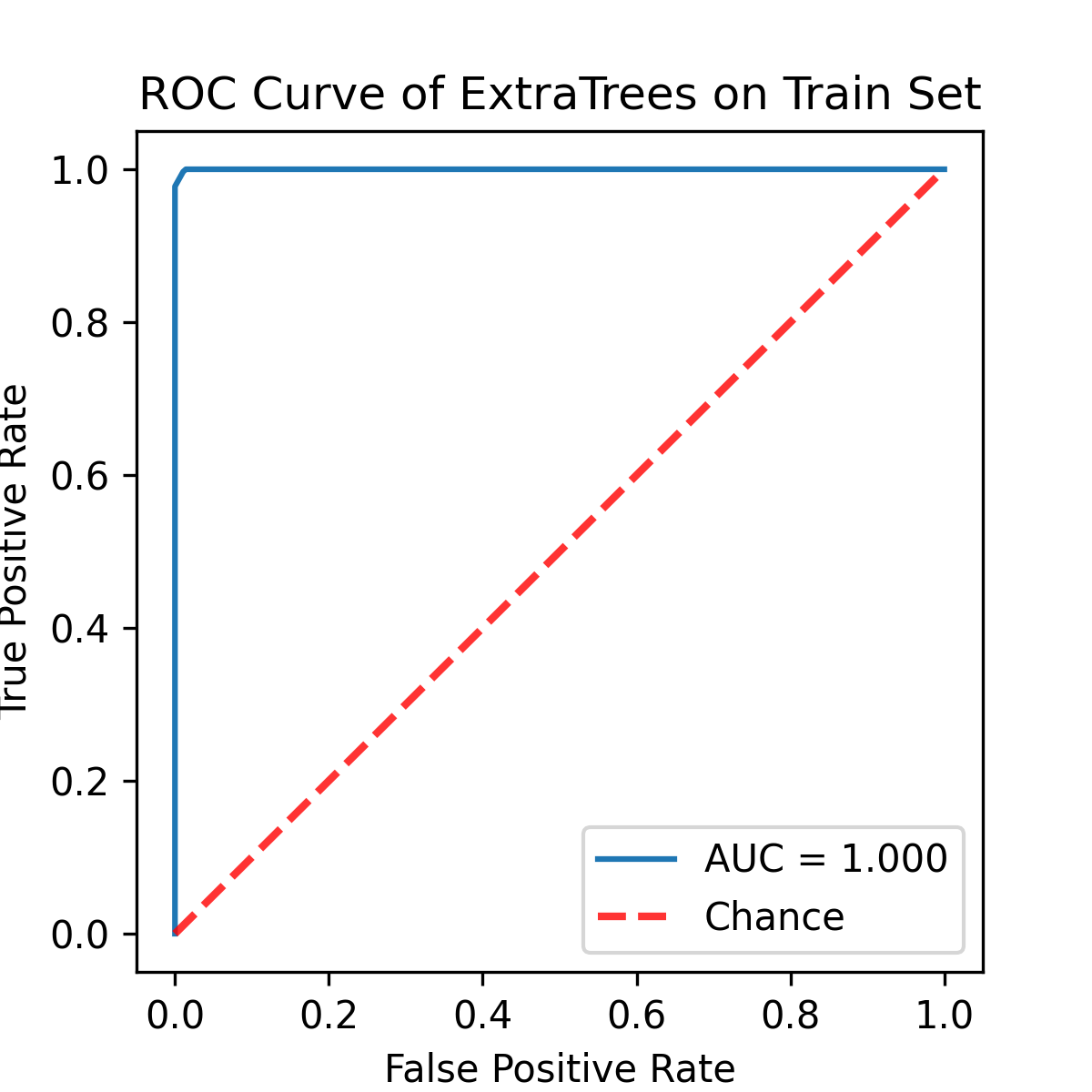

Supplement: Supplementary file 1 [file diagnostics-14-00053-s001.zip › Results of all classifiers/EmbeddingLR/ExtraTrees/Train Set/ROC Curve of ExtraTrees on Train Set.png]

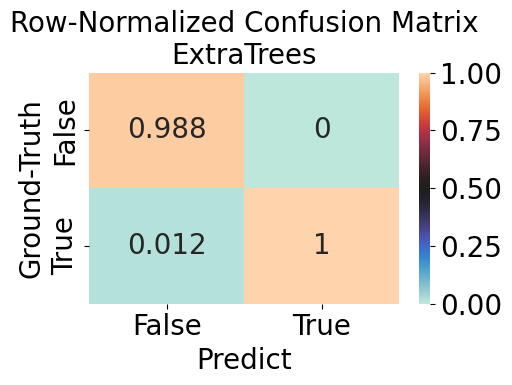

Supplement: Supplementary file 1 [file diagnostics-14-00053-s001.zip › Results of all classifiers/EmbeddingLR/ExtraTrees/Train Set/Row-Normalized Confusion Matrix ExtraTrees.png]

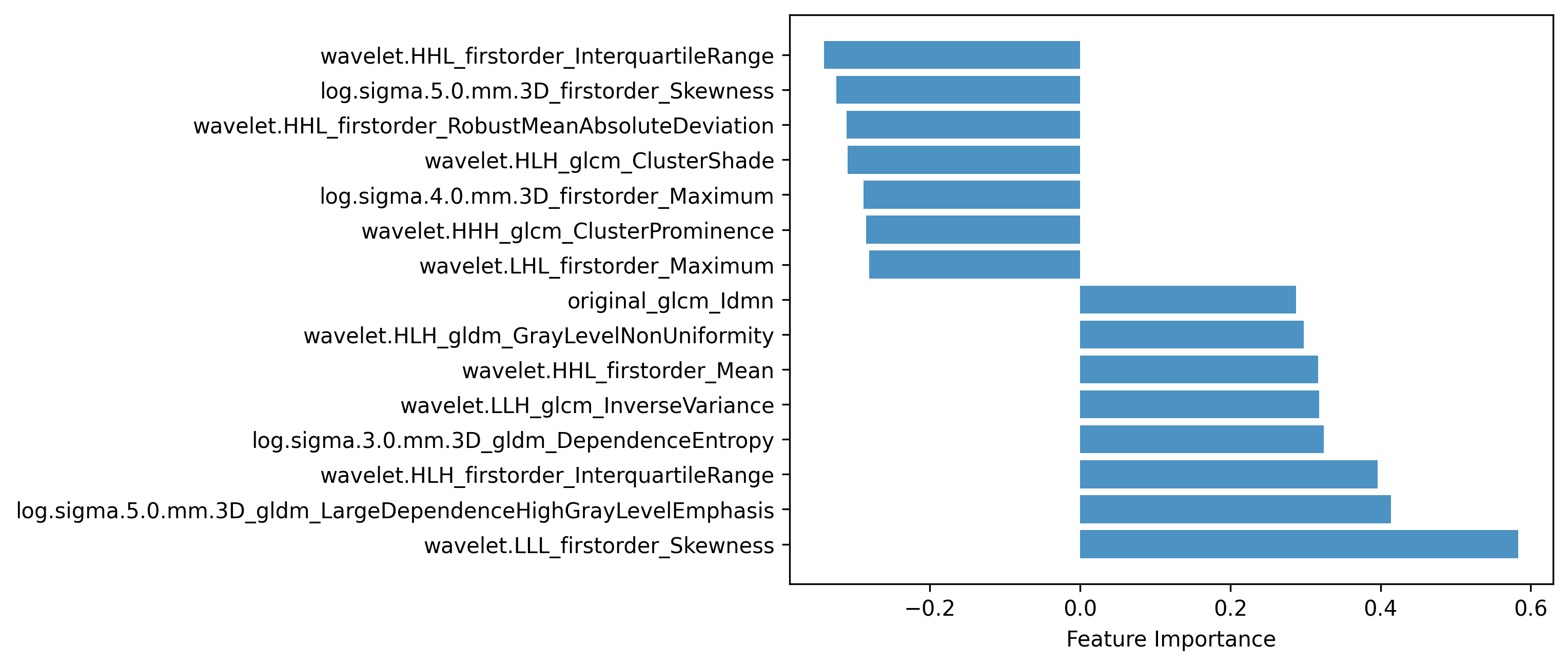

Supplement: Supplementary file 1 [file diagnostics-14-00053-s001.zip › Results of all classifiers/EmbeddingLR/Feature Importance.png]

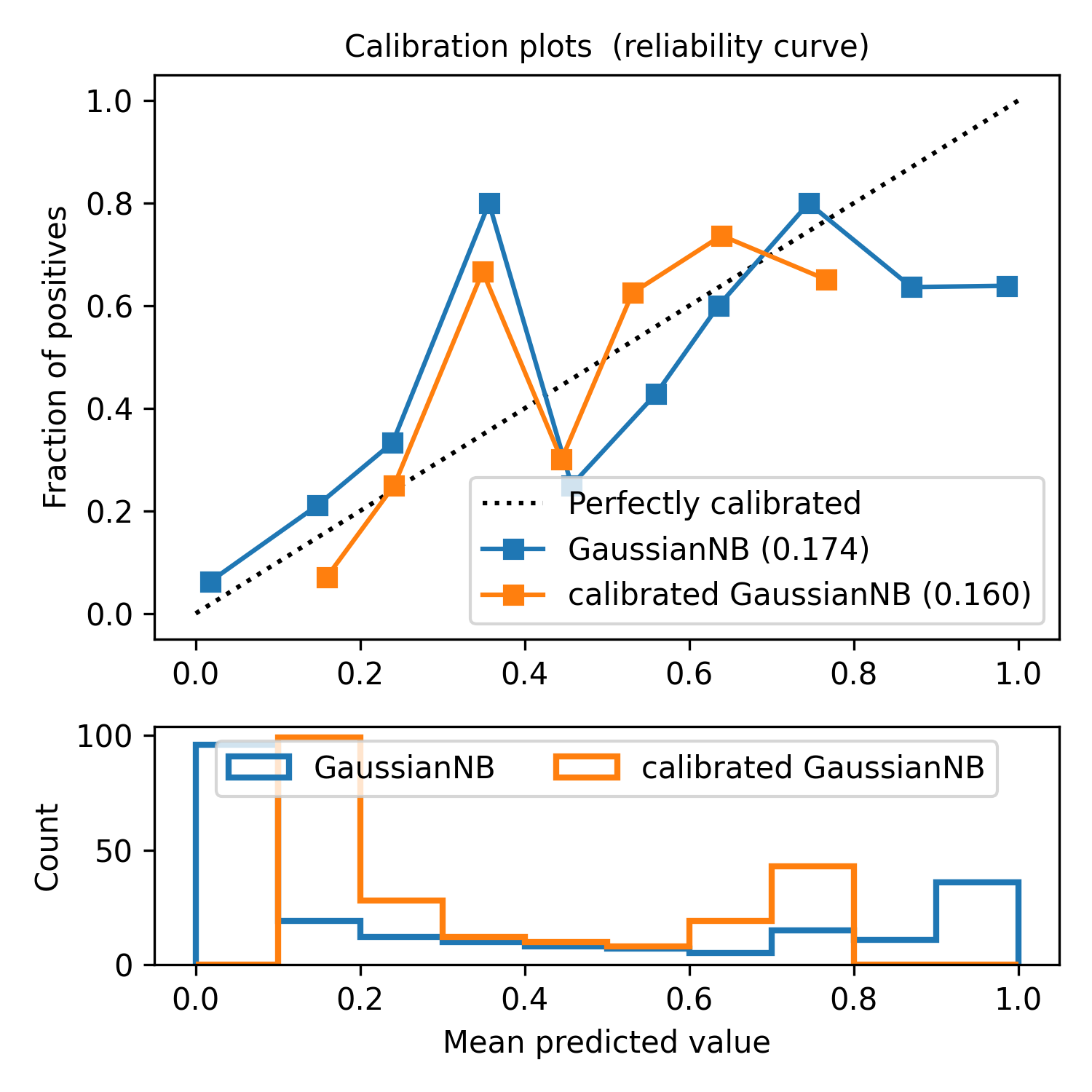

Supplement: Supplementary file 1 [file diagnostics-14-00053-s001.zip › Results of all classifiers/EmbeddingLR/GaussianNB/Test Set/Calibration plots.png]

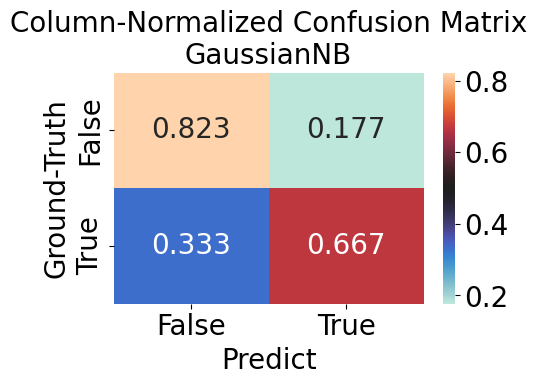

Supplement: Supplementary file 1 [file diagnostics-14-00053-s001.zip › Results of all classifiers/EmbeddingLR/GaussianNB/Test Set/Column-Normalized Confusion Matrix GaussianNB.png]

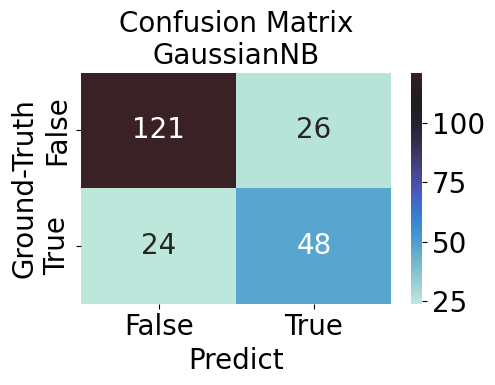

Supplement: Supplementary file 1 [file diagnostics-14-00053-s001.zip › Results of all classifiers/EmbeddingLR/GaussianNB/Test Set/Confusion Matrix GaussianNB.png]

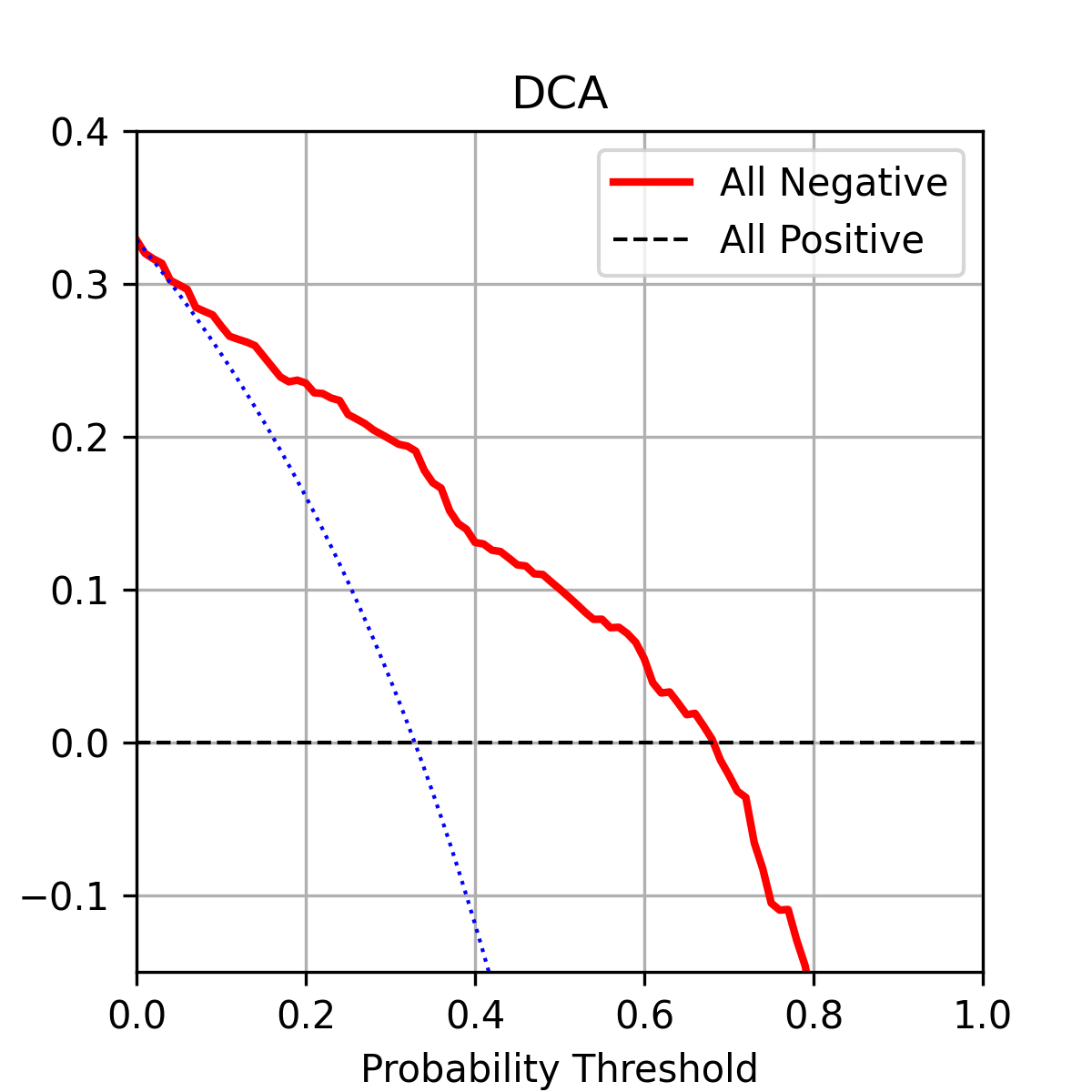

Supplement: Supplementary file 1 [file diagnostics-14-00053-s001.zip › Results of all classifiers/EmbeddingLR/GaussianNB/Test Set/DCA.png]

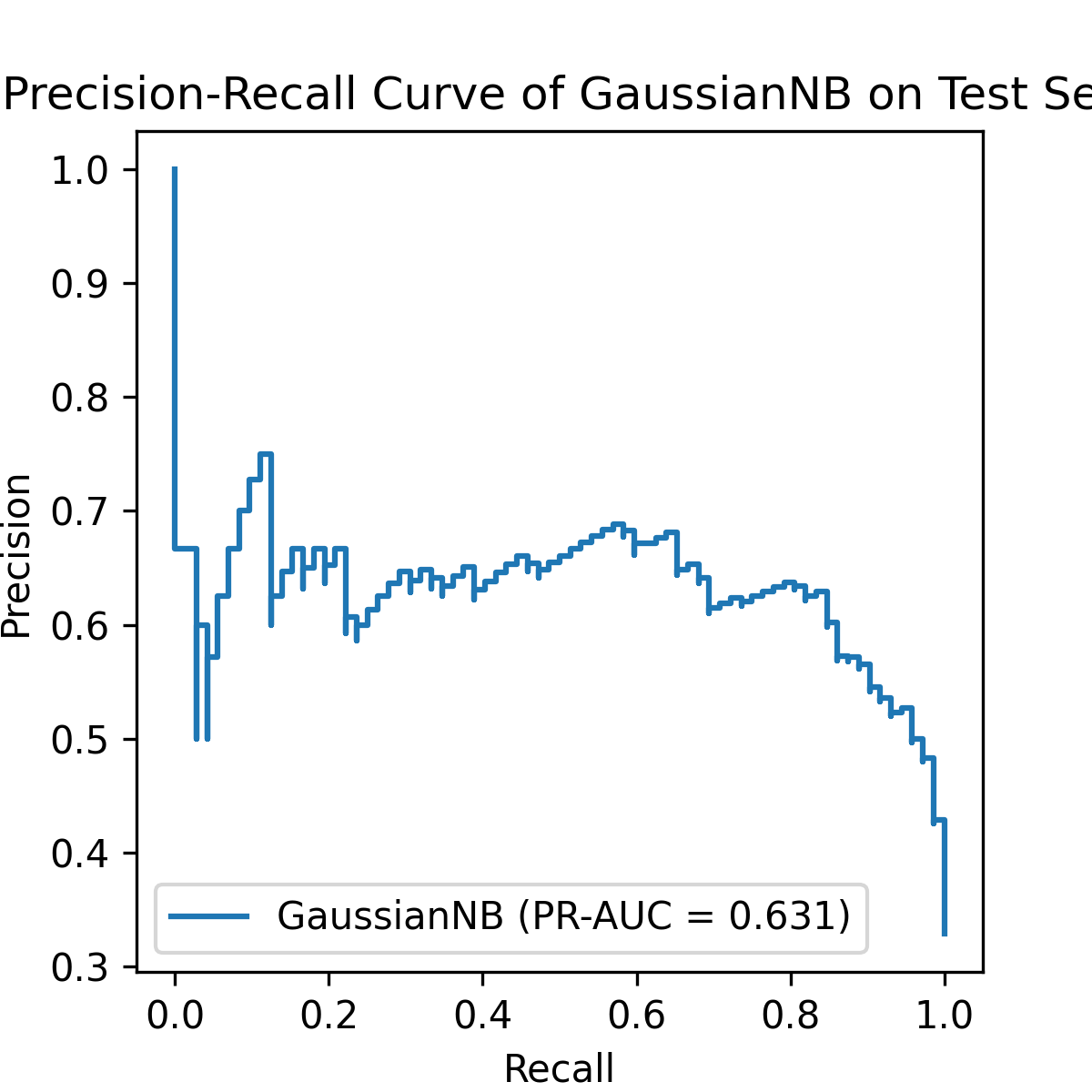

Supplement: Supplementary file 1 [file diagnostics-14-00053-s001.zip › Results of all classifiers/EmbeddingLR/GaussianNB/Test Set/Precision-Recall Curve of GaussianNB on Test Set.png]

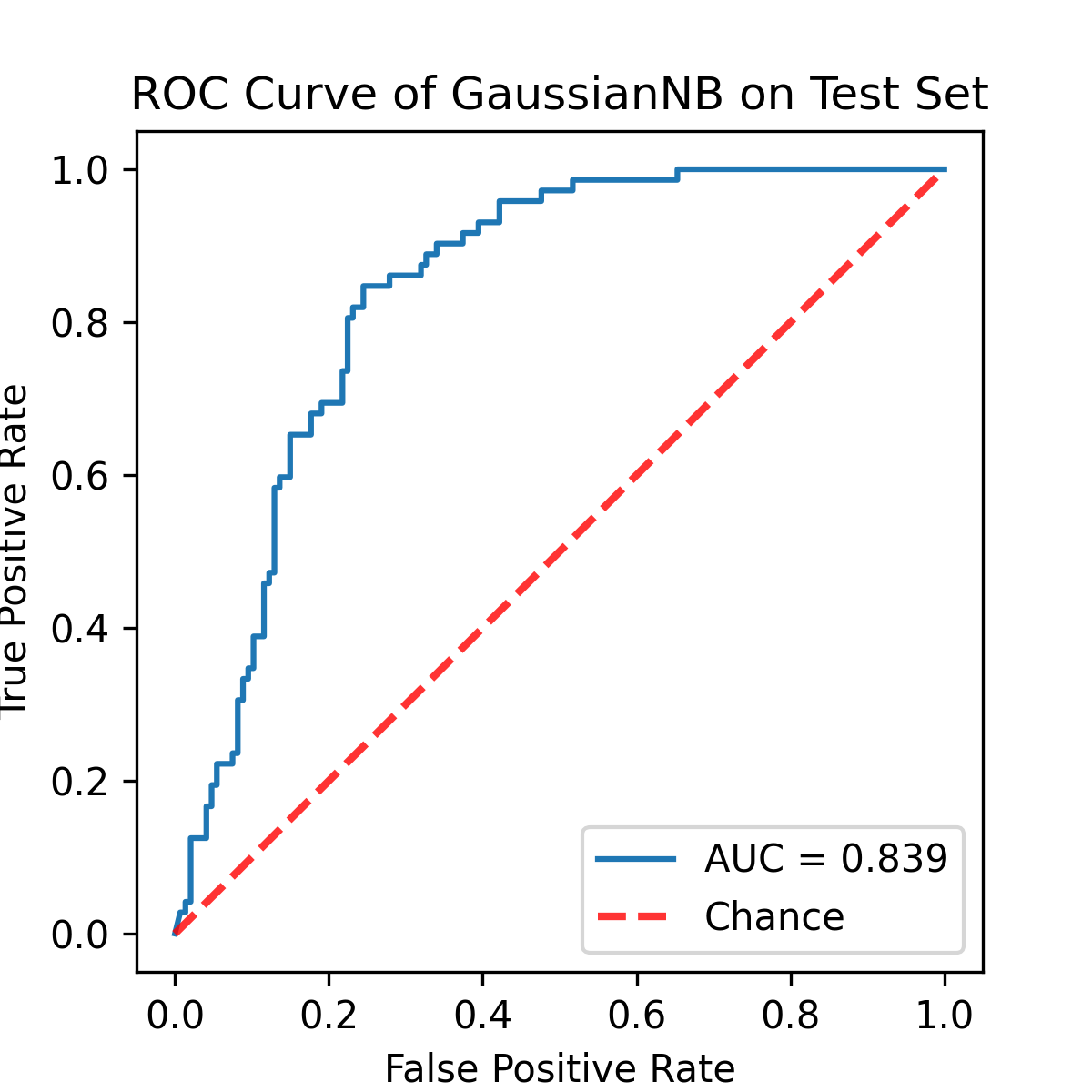

Supplement: Supplementary file 1 [file diagnostics-14-00053-s001.zip › Results of all classifiers/EmbeddingLR/GaussianNB/Test Set/ROC Curve of GaussianNB on Test Set.png]

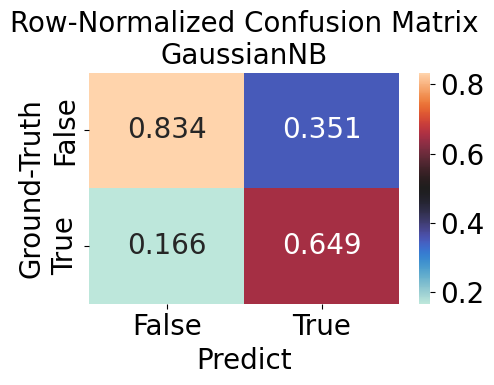

Supplement: Supplementary file 1 [file diagnostics-14-00053-s001.zip › Results of all classifiers/EmbeddingLR/GaussianNB/Test Set/Row-Normalized Confusion Matrix GaussianNB.png]

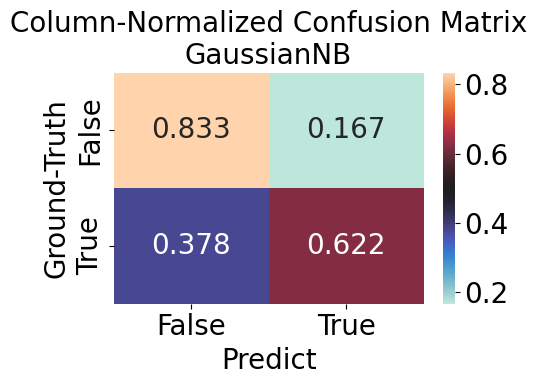

Supplement: Supplementary file 1 [file diagnostics-14-00053-s001.zip › Results of all classifiers/EmbeddingLR/GaussianNB/Train Set/Column-Normalized Confusion Matrix GaussianNB.png]

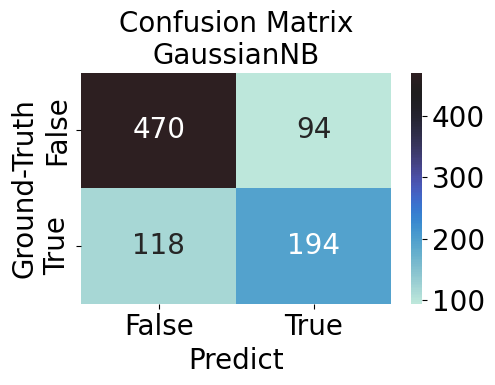

Supplement: Supplementary file 1 [file diagnostics-14-00053-s001.zip › Results of all classifiers/EmbeddingLR/GaussianNB/Train Set/Confusion Matrix GaussianNB.png]

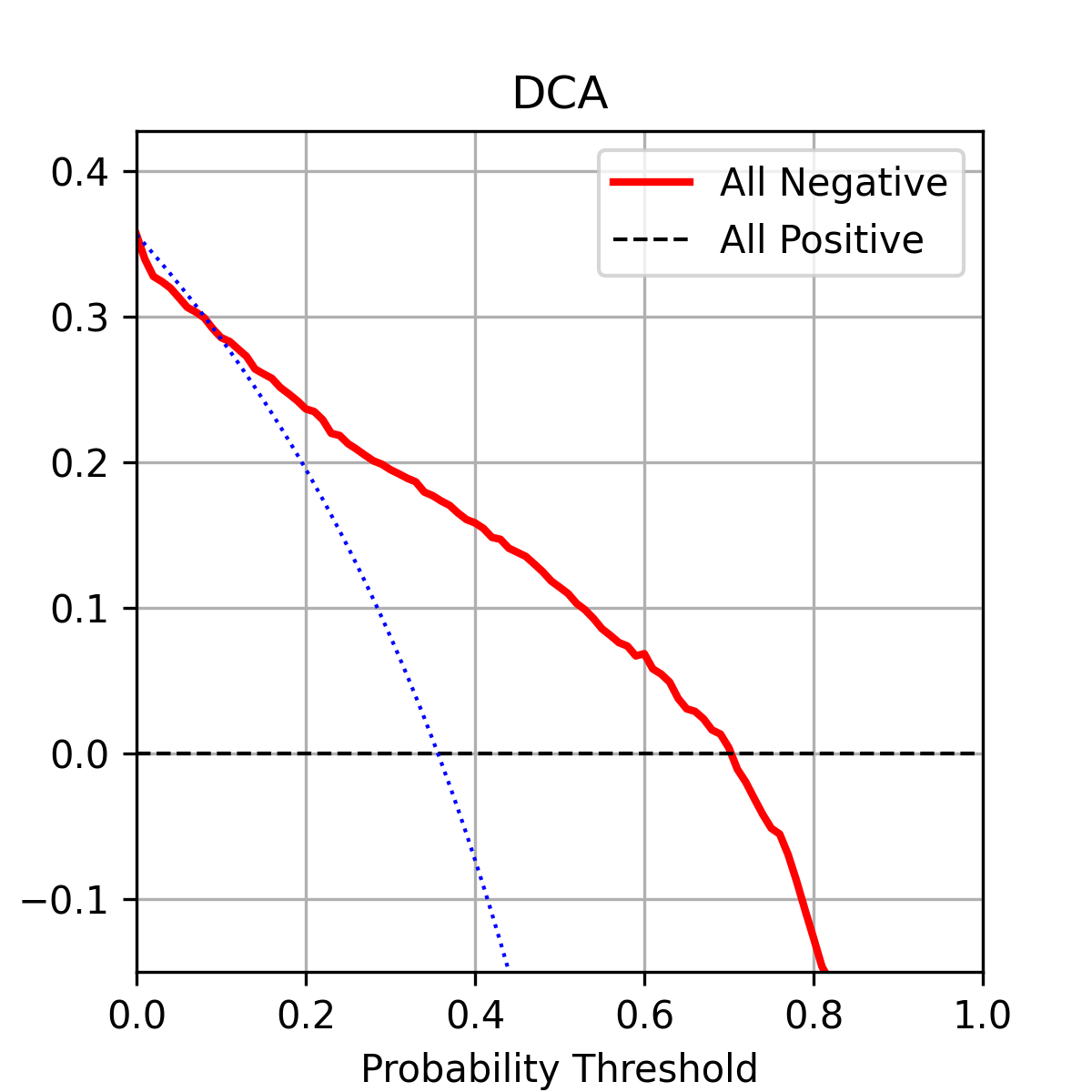

Supplement: Supplementary file 1 [file diagnostics-14-00053-s001.zip › Results of all classifiers/EmbeddingLR/GaussianNB/Train Set/DCA.png]

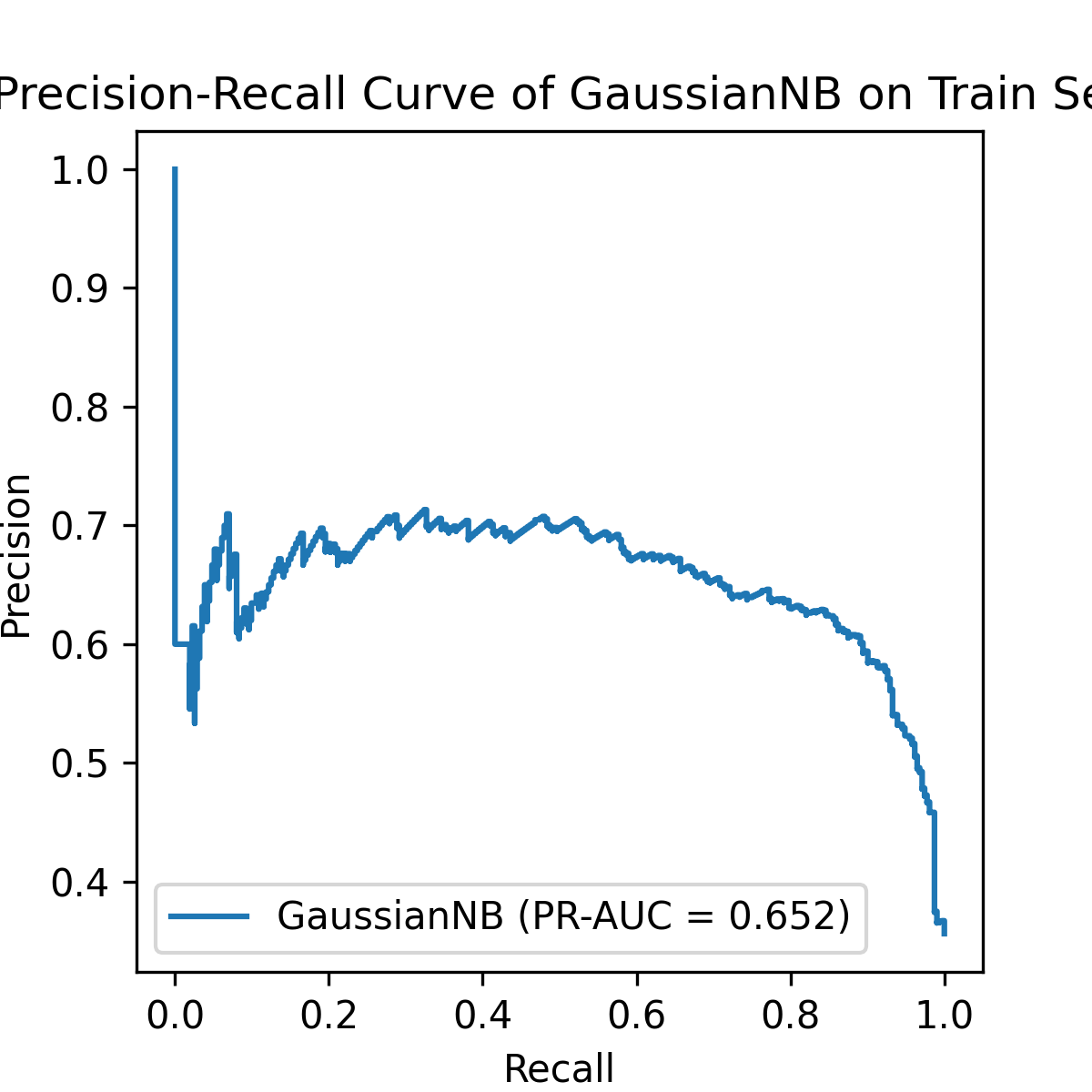

Supplement: Supplementary file 1 [file diagnostics-14-00053-s001.zip › Results of all classifiers/EmbeddingLR/GaussianNB/Train Set/Precision-Recall Curve of GaussianNB on Train Set.png]

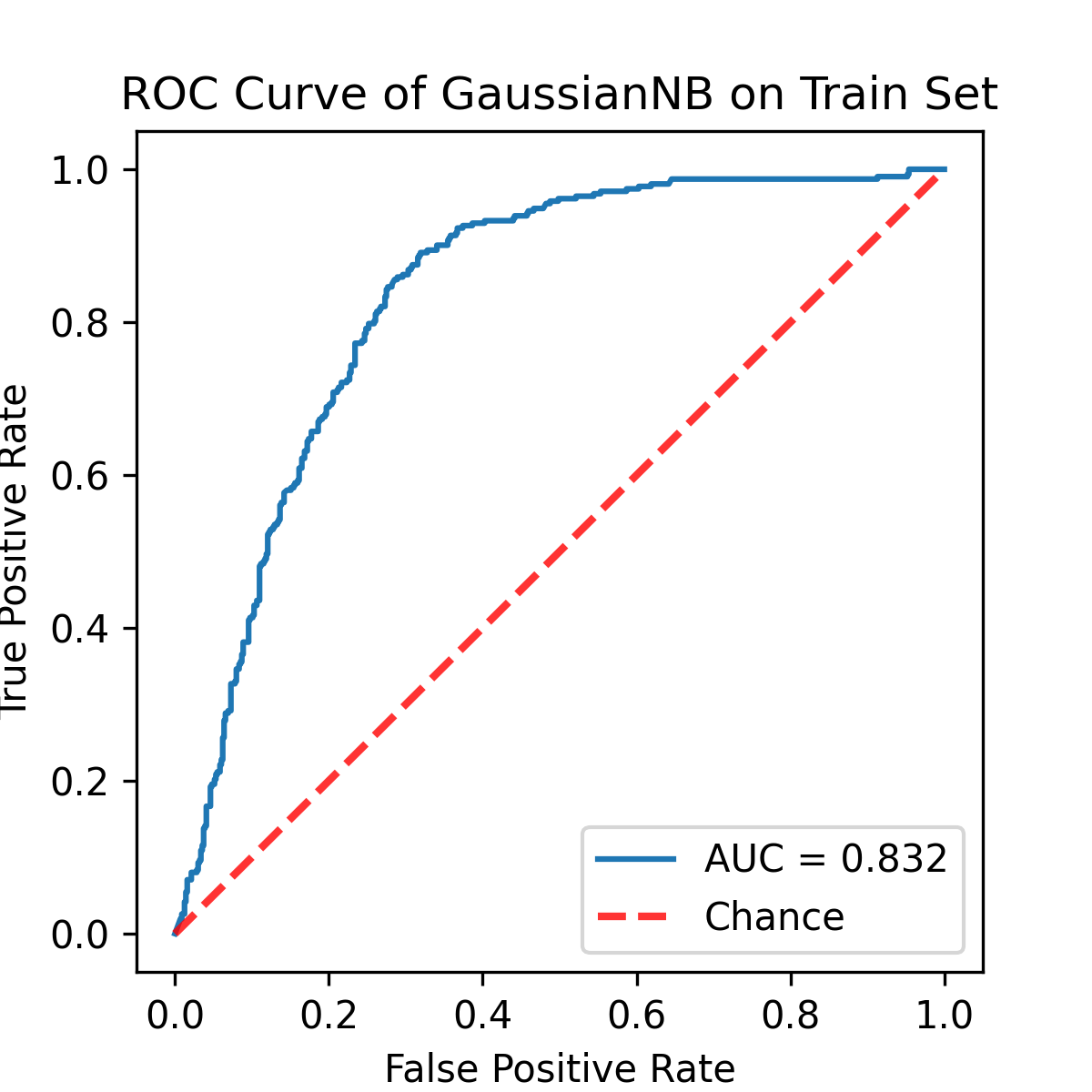

Supplement: Supplementary file 1 [file diagnostics-14-00053-s001.zip › Results of all classifiers/EmbeddingLR/GaussianNB/Train Set/ROC Curve of GaussianNB on Train Set.png]

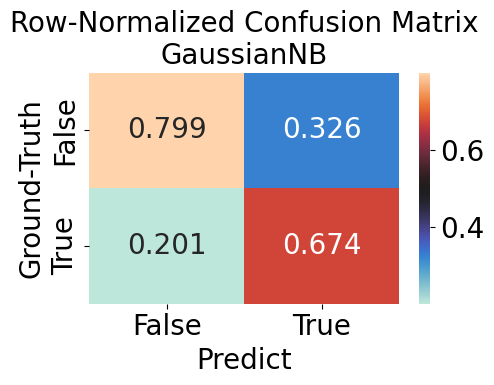

Supplement: Supplementary file 1 [file diagnostics-14-00053-s001.zip › Results of all classifiers/EmbeddingLR/GaussianNB/Train Set/Row-Normalized Confusion Matrix GaussianNB.png]

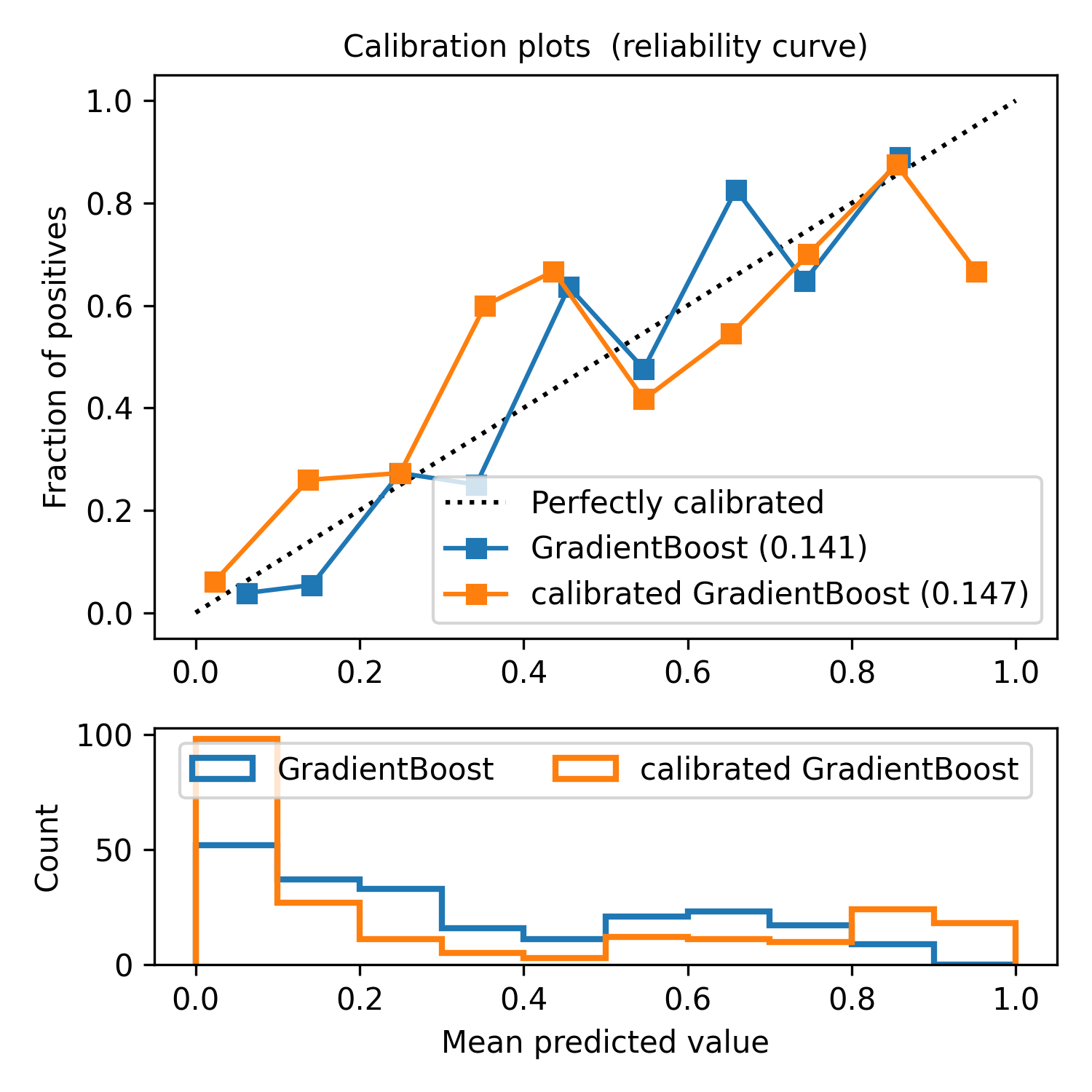

Supplement: Supplementary file 1 [file diagnostics-14-00053-s001.zip › Results of all classifiers/EmbeddingLR/GradientBoost/Test Set/Calibration plots.png]

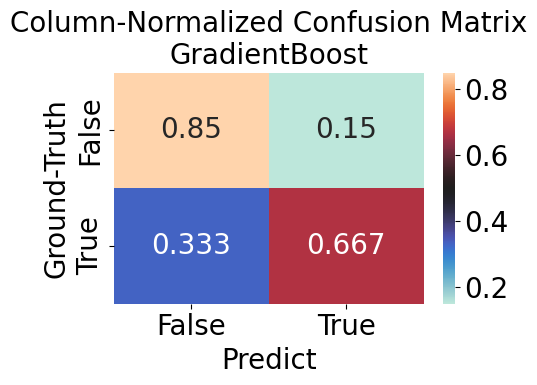

Supplement: Supplementary file 1 [file diagnostics-14-00053-s001.zip › Results of all classifiers/EmbeddingLR/GradientBoost/Test Set/Column-Normalized Confusion Matrix GradientBoost.png]

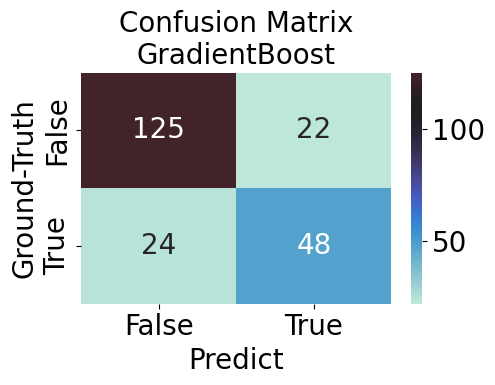

Supplement: Supplementary file 1 [file diagnostics-14-00053-s001.zip › Results of all classifiers/EmbeddingLR/GradientBoost/Test Set/Confusion Matrix GradientBoost.png]

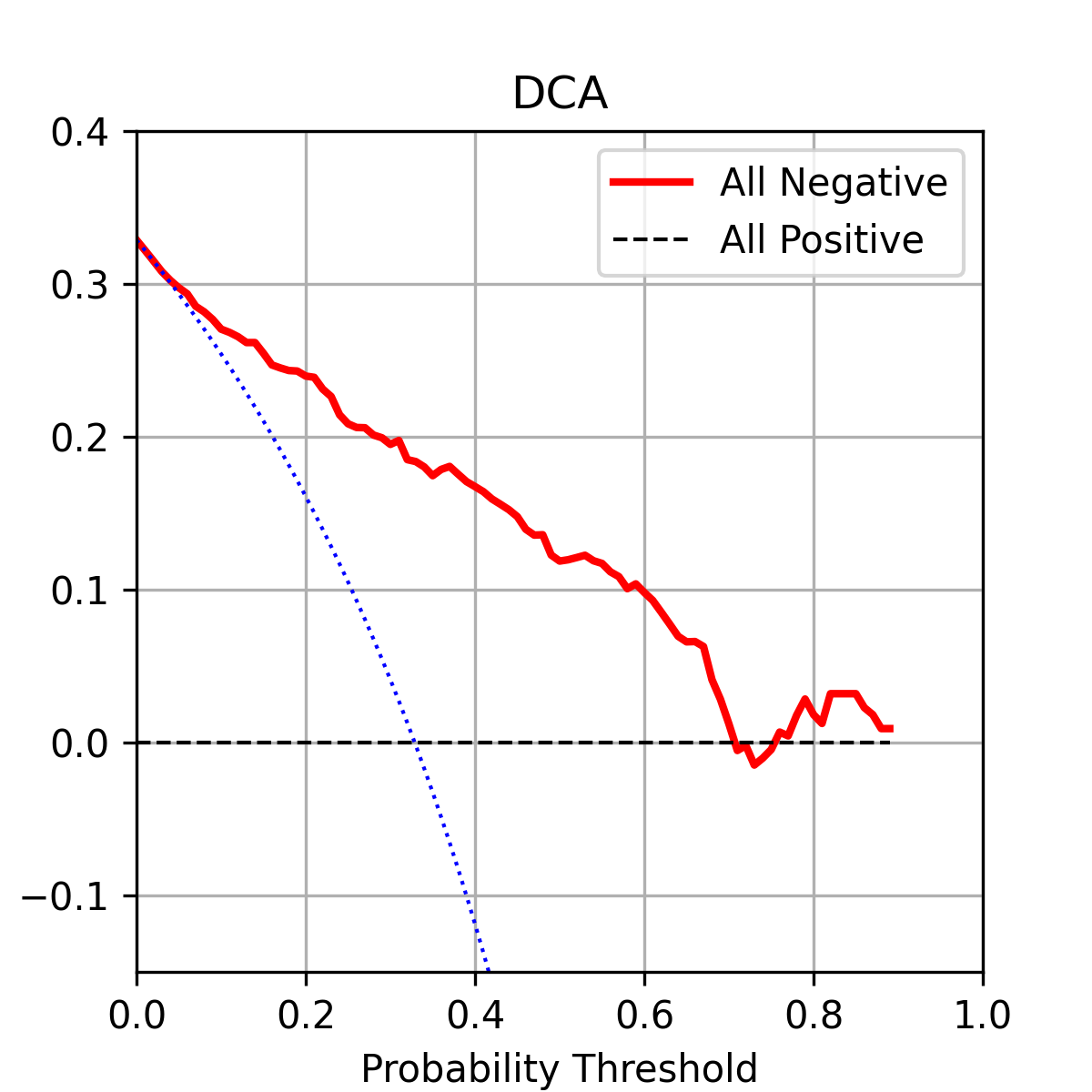

Supplement: Supplementary file 1 [file diagnostics-14-00053-s001.zip › Results of all classifiers/EmbeddingLR/GradientBoost/Test Set/DCA.png]

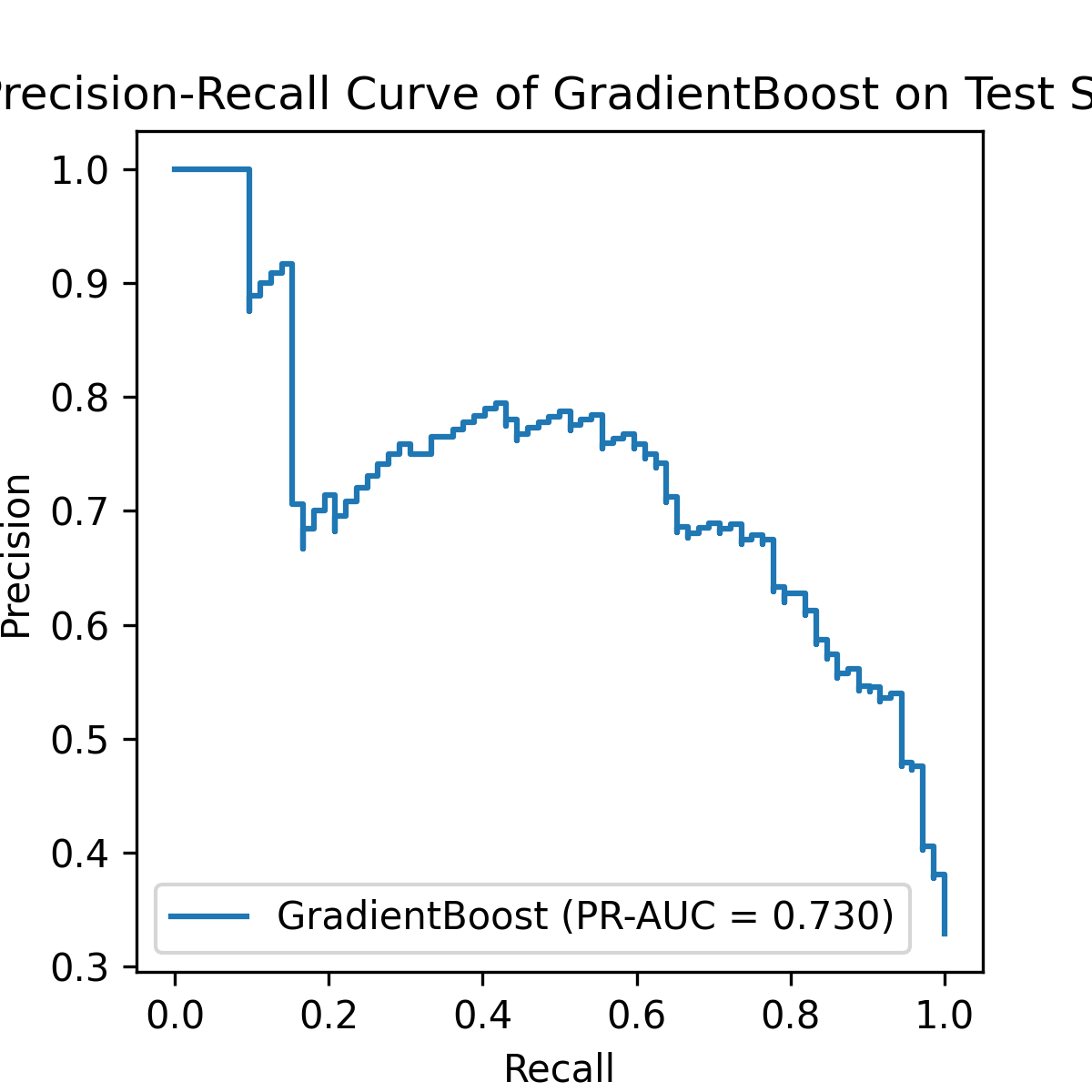

Supplement: Supplementary file 1 [file diagnostics-14-00053-s001.zip › Results of all classifiers/EmbeddingLR/GradientBoost/Test Set/Precision-Recall Curve of GradientBoost on Test Set.png]

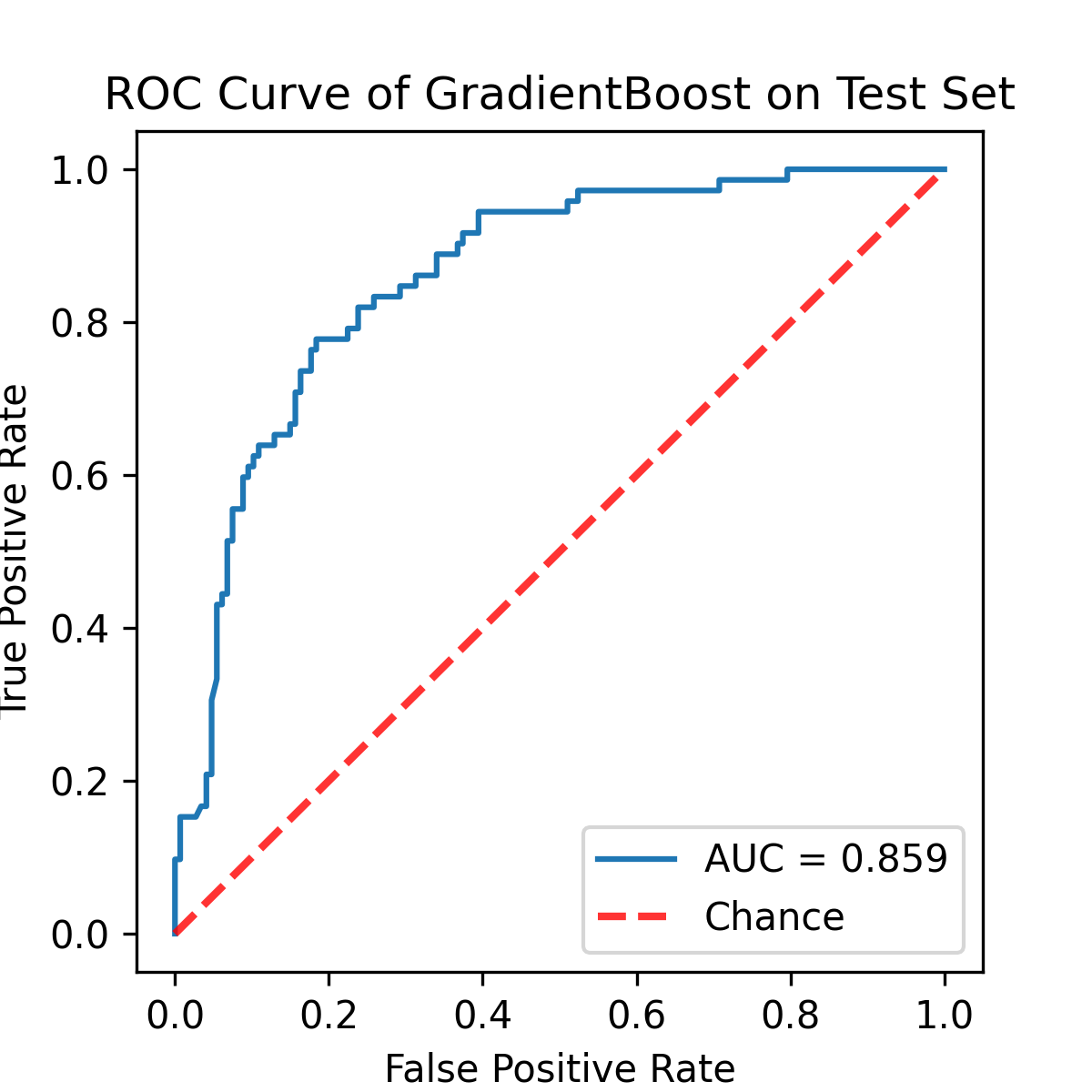

Supplement: Supplementary file 1 [file diagnostics-14-00053-s001.zip › Results of all classifiers/EmbeddingLR/GradientBoost/Test Set/ROC Curve of GradientBoost on Test Set.png]

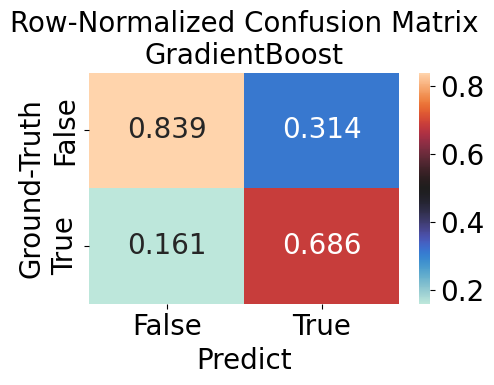

Supplement: Supplementary file 1 [file diagnostics-14-00053-s001.zip › Results of all classifiers/EmbeddingLR/GradientBoost/Test Set/Row-Normalized Confusion Matrix GradientBoost.png]

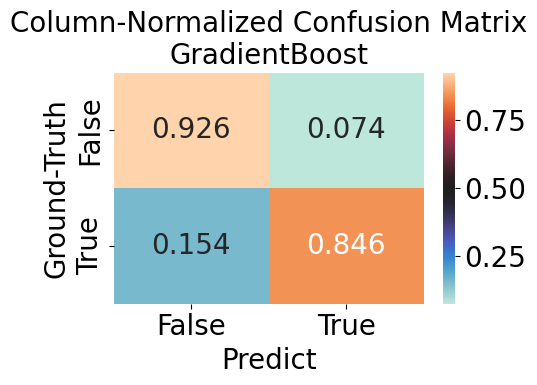

Supplement: Supplementary file 1 [file diagnostics-14-00053-s001.zip › Results of all classifiers/EmbeddingLR/GradientBoost/Train Set/Column-Normalized Confusion Matrix GradientBoost.png]

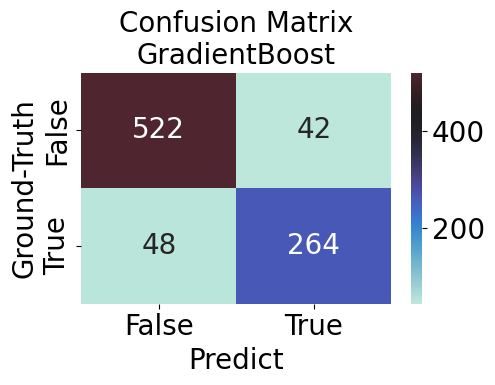

Supplement: Supplementary file 1 [file diagnostics-14-00053-s001.zip › Results of all classifiers/EmbeddingLR/GradientBoost/Train Set/Confusion Matrix GradientBoost.png]

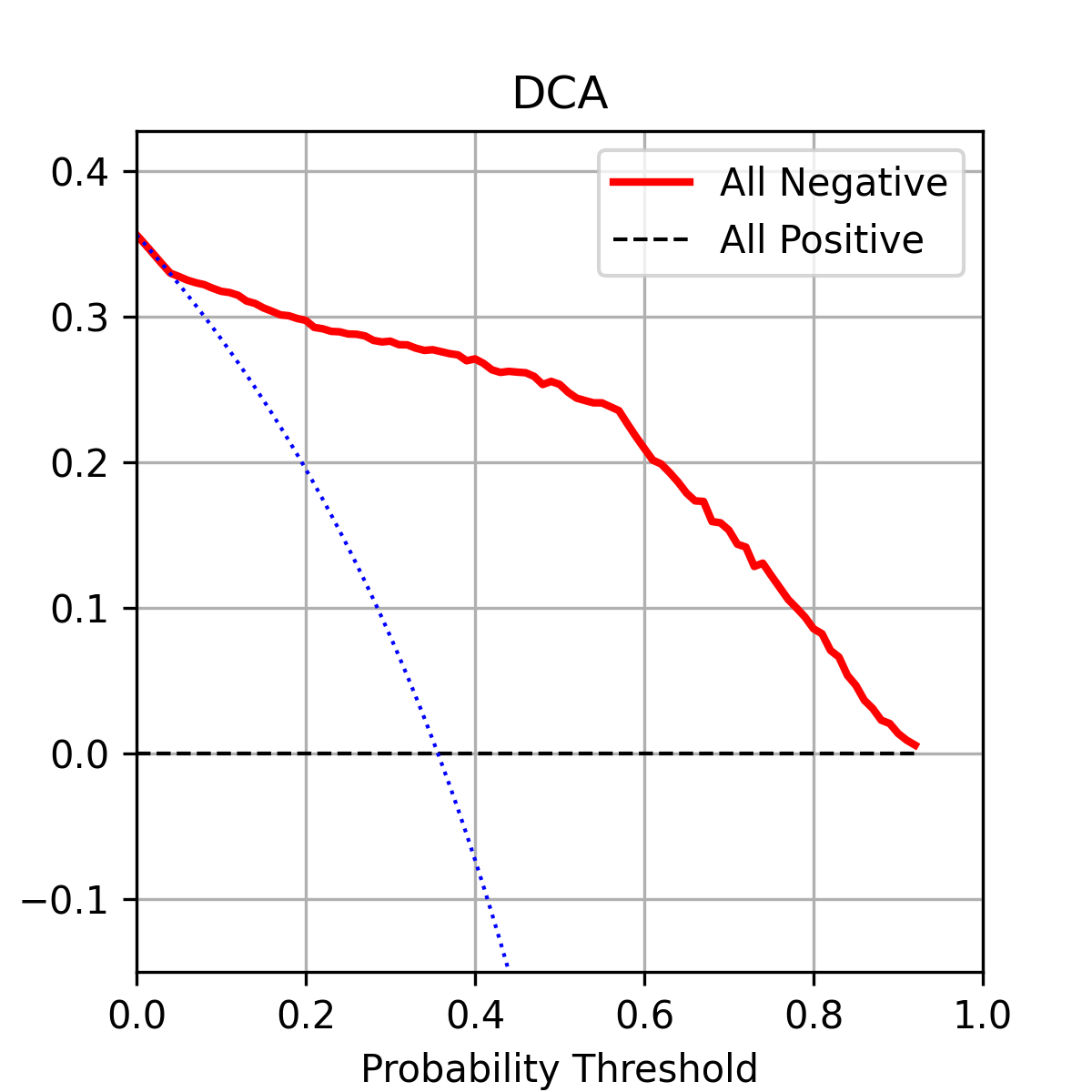

Supplement: Supplementary file 1 [file diagnostics-14-00053-s001.zip › Results of all classifiers/EmbeddingLR/GradientBoost/Train Set/DCA.png]

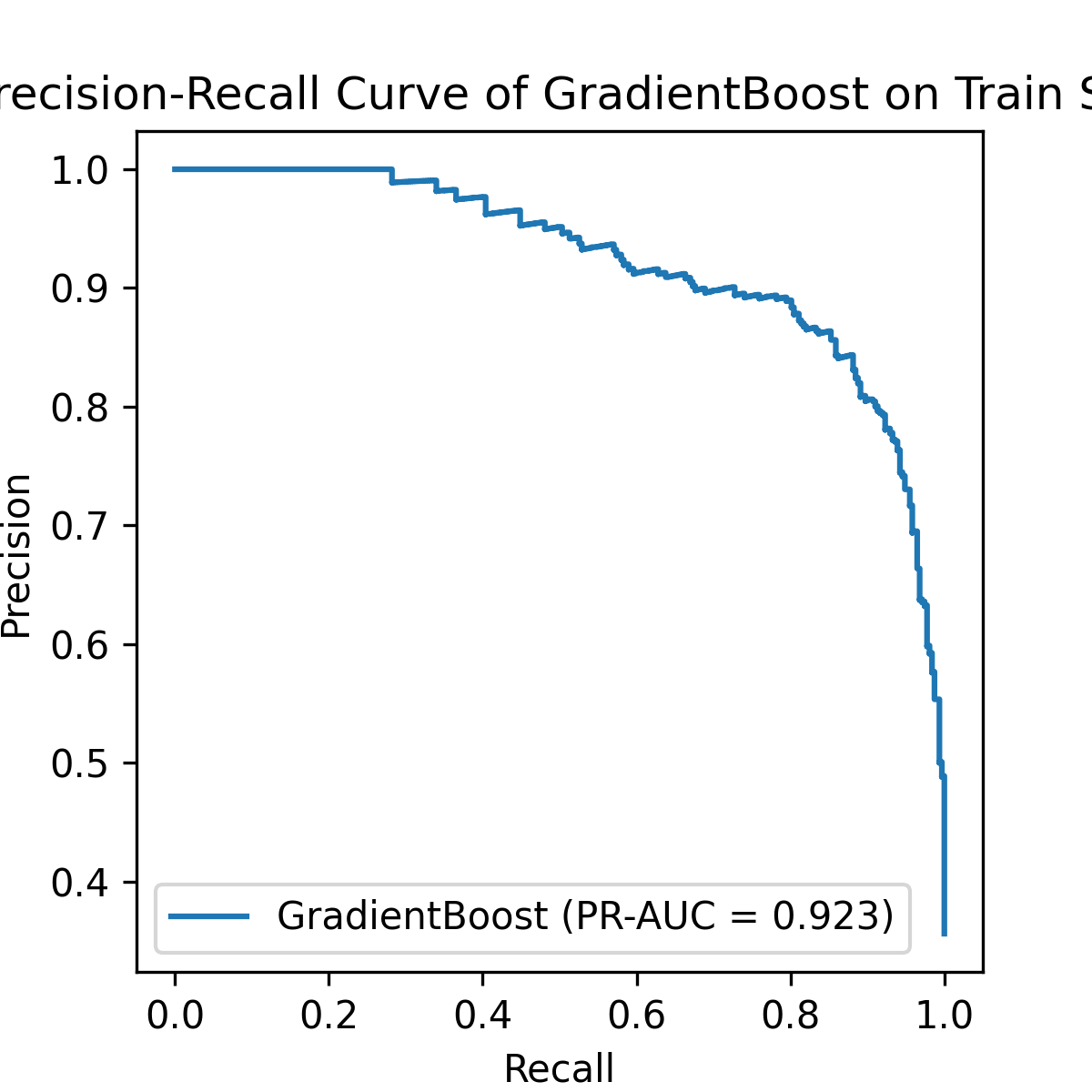

Supplement: Supplementary file 1 [file diagnostics-14-00053-s001.zip › Results of all classifiers/EmbeddingLR/GradientBoost/Train Set/Precision-Recall Curve of GradientBoost on Train Set.png]

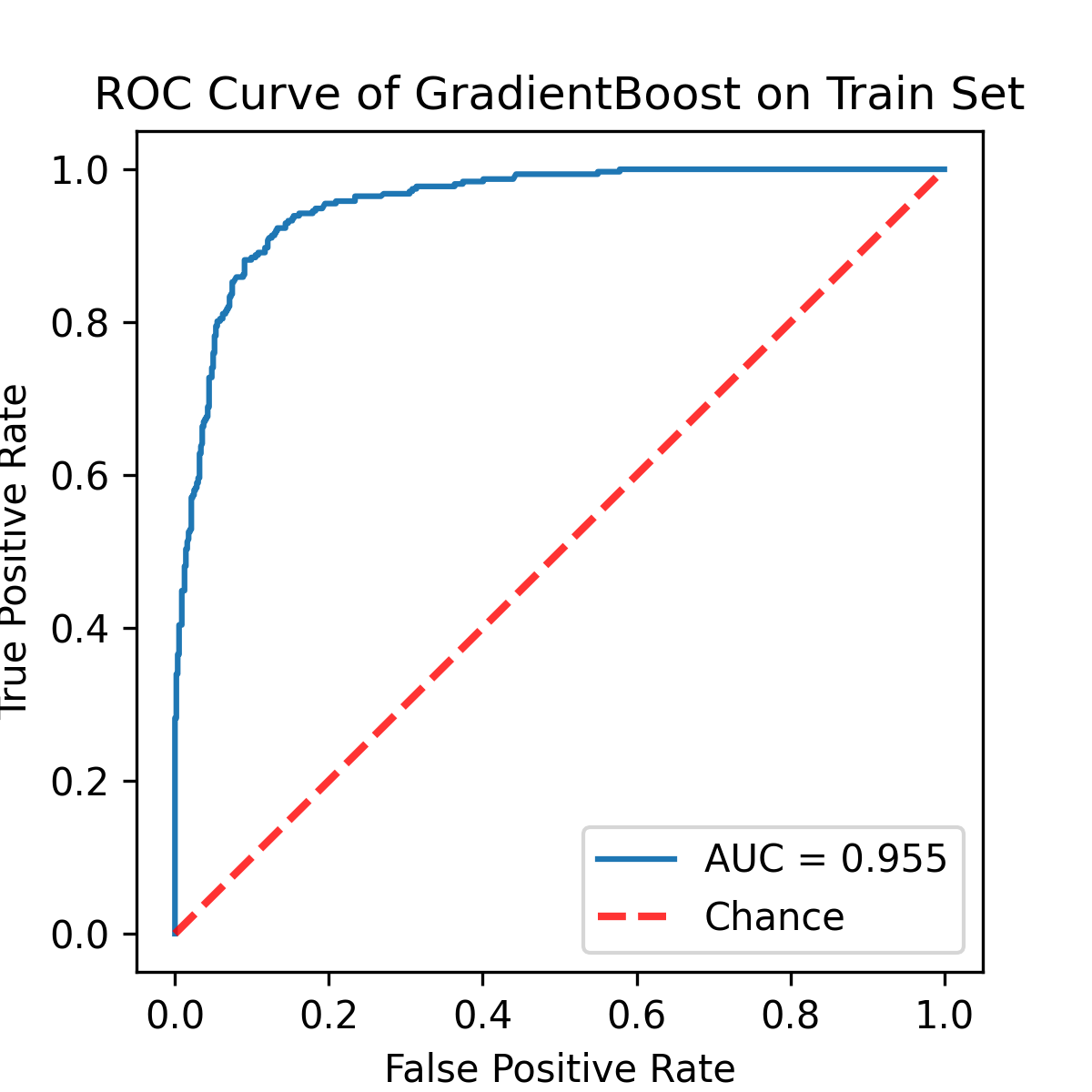

Supplement: Supplementary file 1 [file diagnostics-14-00053-s001.zip › Results of all classifiers/EmbeddingLR/GradientBoost/Train Set/ROC Curve of GradientBoost on Train Set.png]

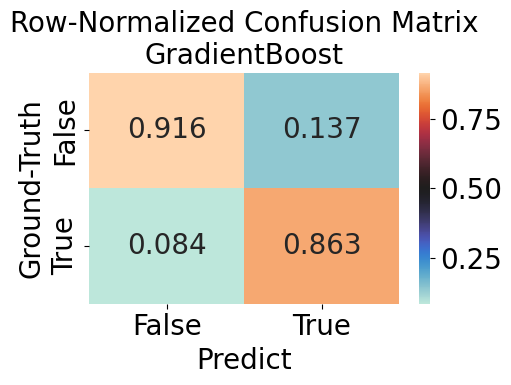

Supplement: Supplementary file 1 [file diagnostics-14-00053-s001.zip › Results of all classifiers/EmbeddingLR/GradientBoost/Train Set/Row-Normalized Confusion Matrix GradientBoost.png]

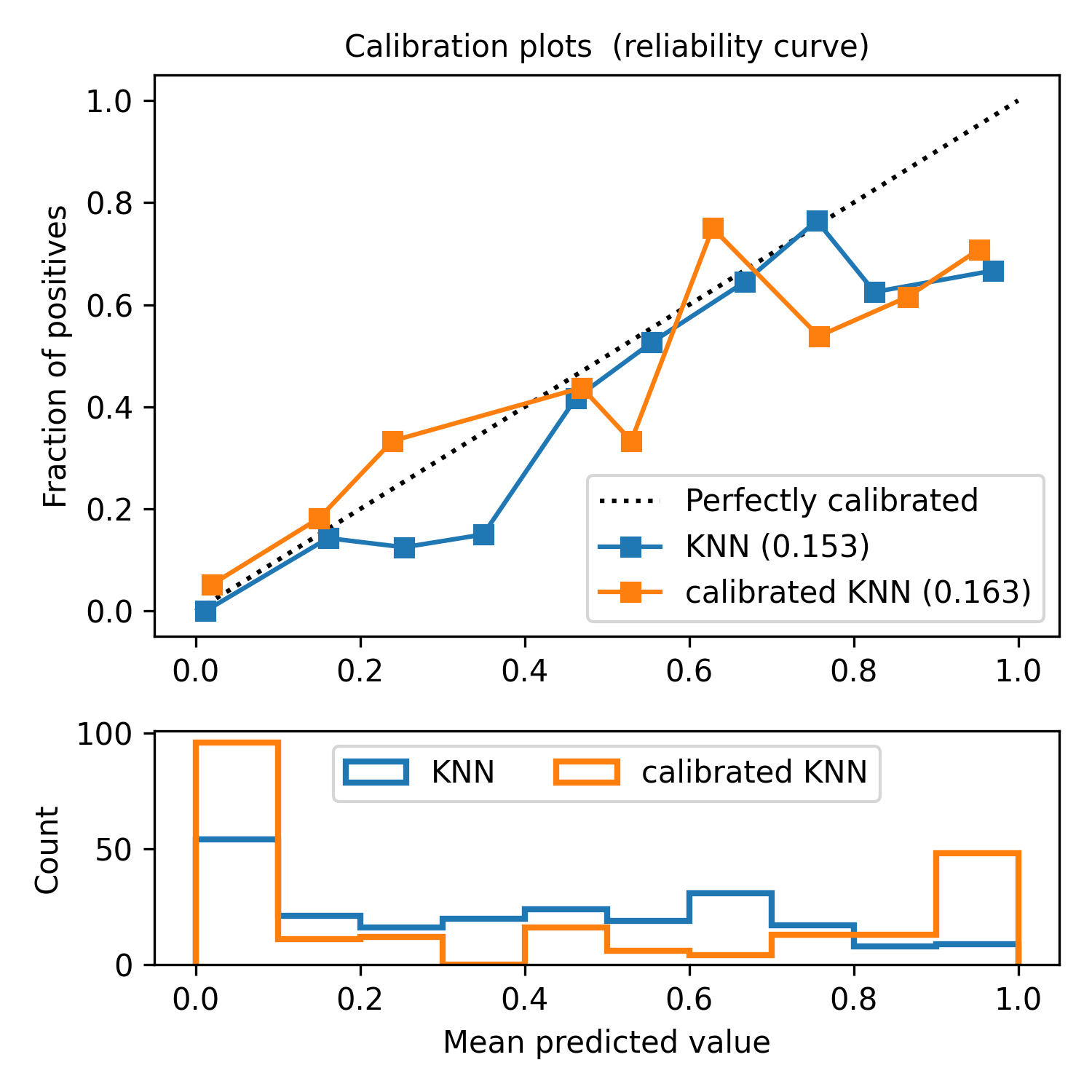

Supplement: Supplementary file 1 [file diagnostics-14-00053-s001.zip › Results of all classifiers/EmbeddingLR/KNN/Test Set/Calibration plots.png]

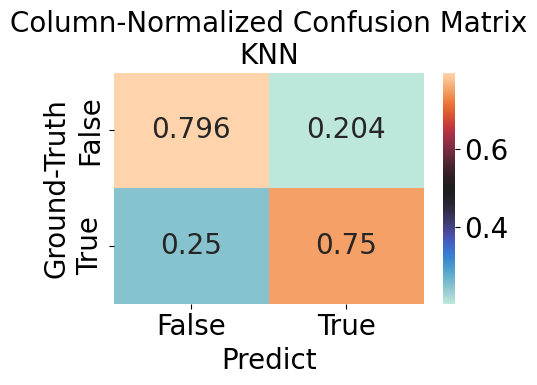

Supplement: Supplementary file 1 [file diagnostics-14-00053-s001.zip › Results of all classifiers/EmbeddingLR/KNN/Test Set/Column-Normalized Confusion Matrix KNN.png]

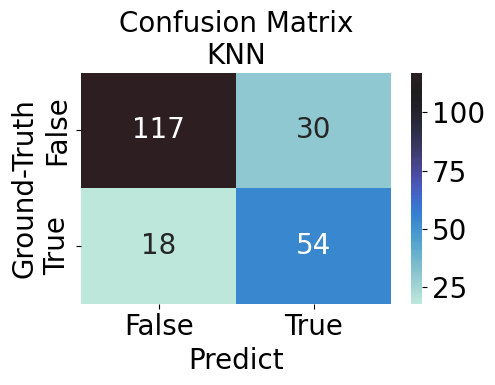

Supplement: Supplementary file 1 [file diagnostics-14-00053-s001.zip › Results of all classifiers/EmbeddingLR/KNN/Test Set/Confusion Matrix KNN.png]

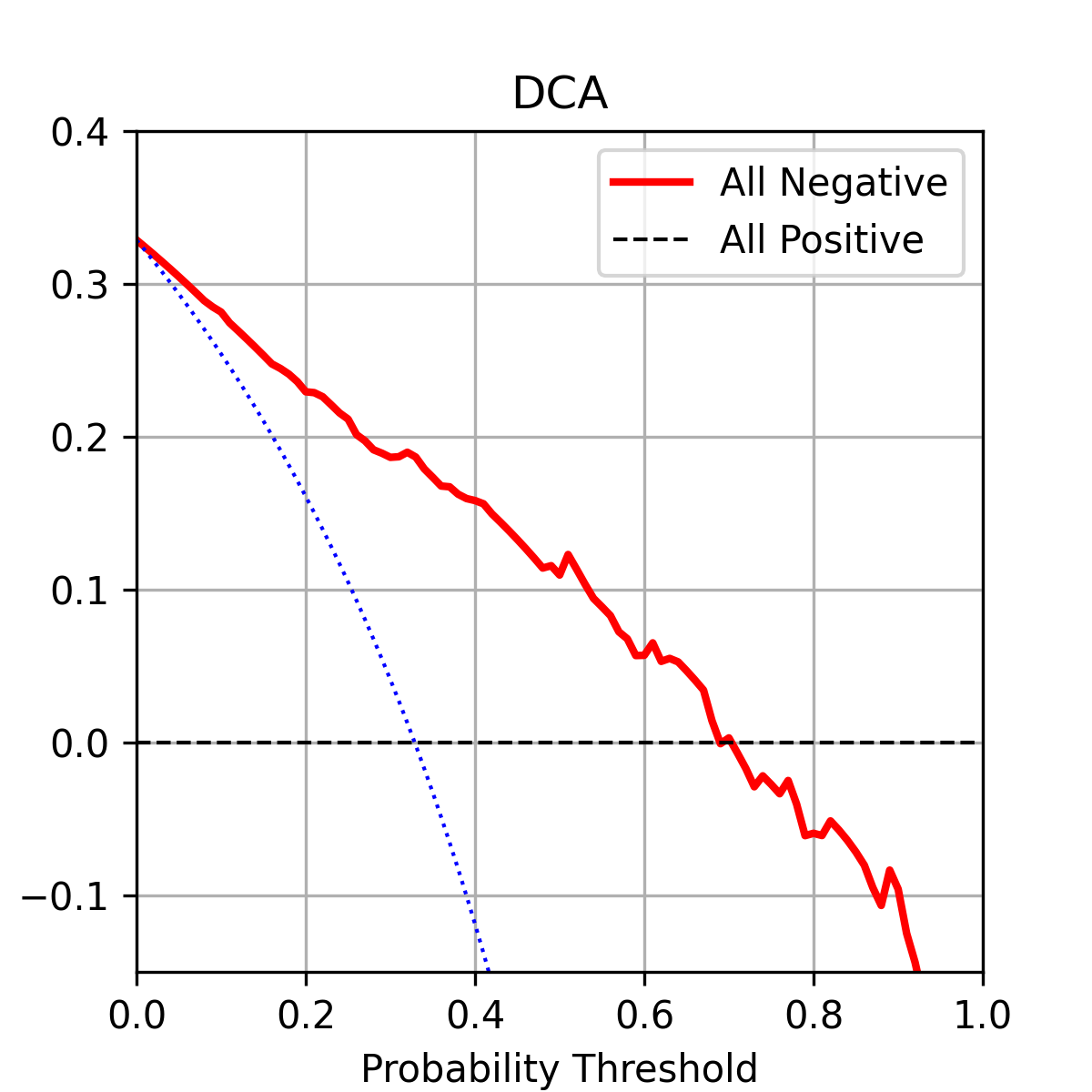

Supplement: Supplementary file 1 [file diagnostics-14-00053-s001.zip › Results of all classifiers/EmbeddingLR/KNN/Test Set/DCA.png]

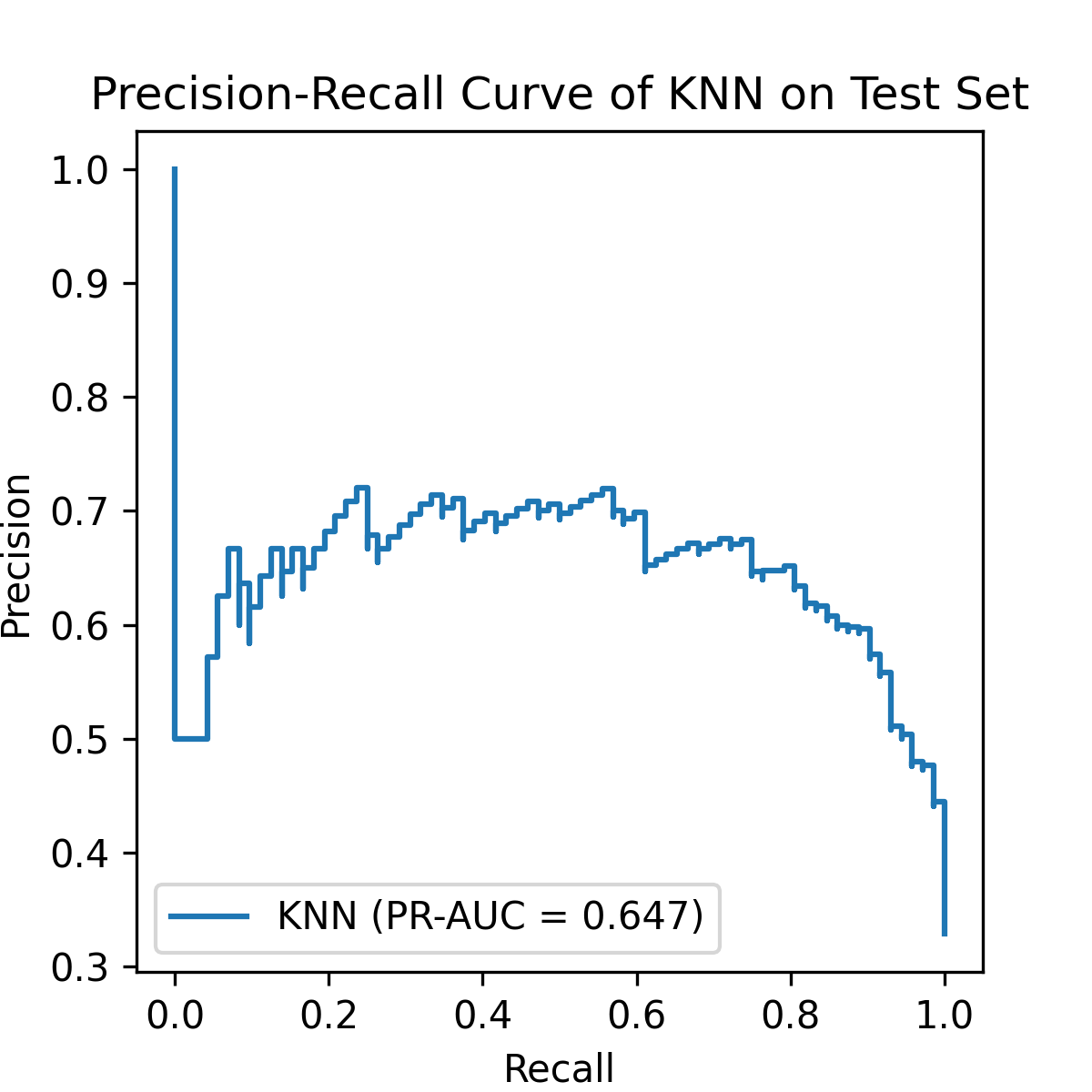

Supplement: Supplementary file 1 [file diagnostics-14-00053-s001.zip › Results of all classifiers/EmbeddingLR/KNN/Test Set/Precision-Recall Curve of KNN on Test Set.png]

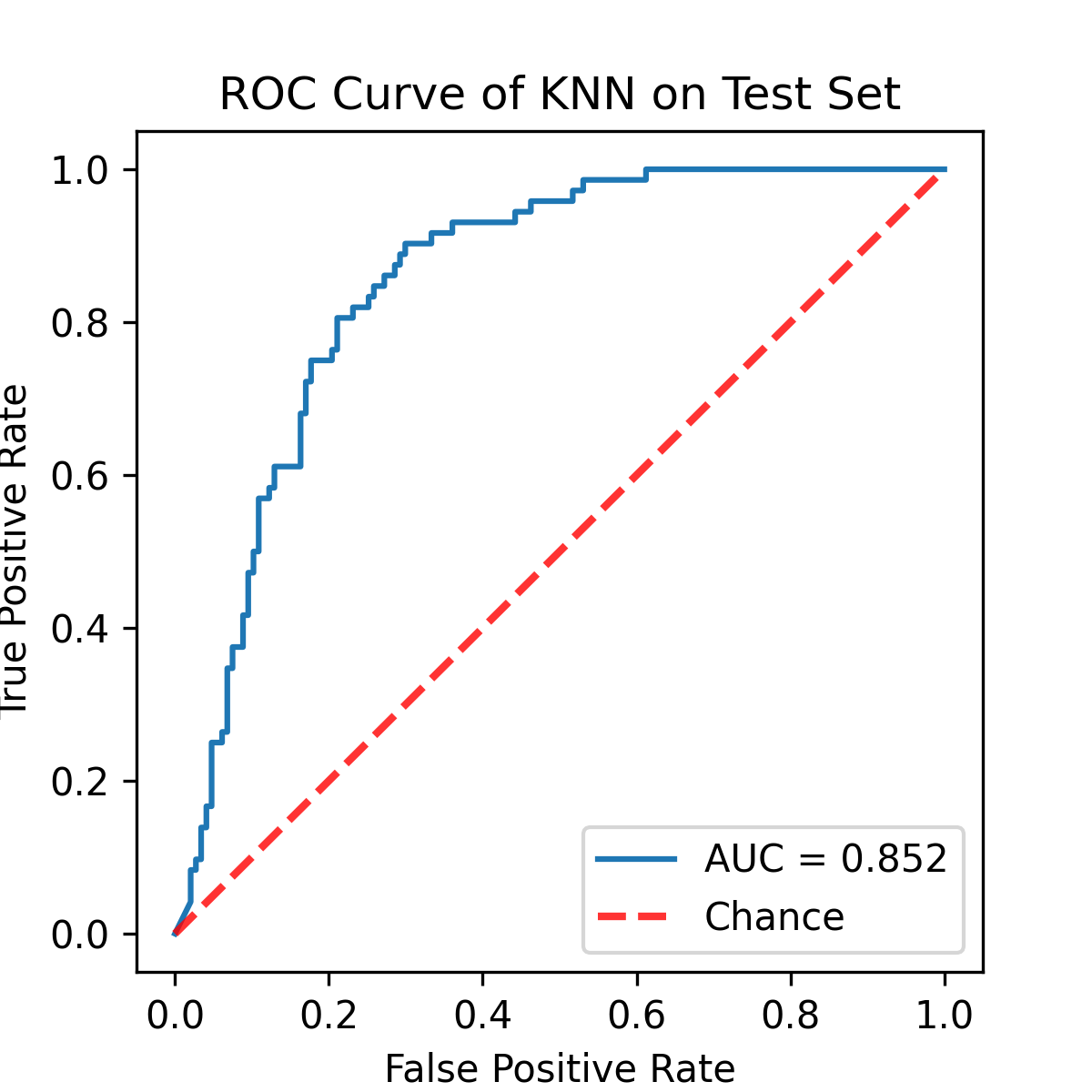

Supplement: Supplementary file 1 [file diagnostics-14-00053-s001.zip › Results of all classifiers/EmbeddingLR/KNN/Test Set/ROC Curve of KNN on Test Set.png]

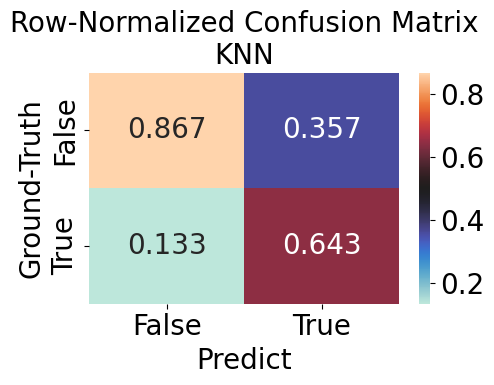

Supplement: Supplementary file 1 [file diagnostics-14-00053-s001.zip › Results of all classifiers/EmbeddingLR/KNN/Test Set/Row-Normalized Confusion Matrix KNN.png]

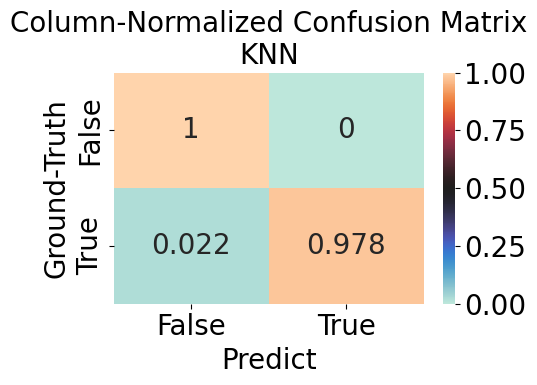

Supplement: Supplementary file 1 [file diagnostics-14-00053-s001.zip › Results of all classifiers/EmbeddingLR/KNN/Train Set/Column-Normalized Confusion Matrix KNN.png]

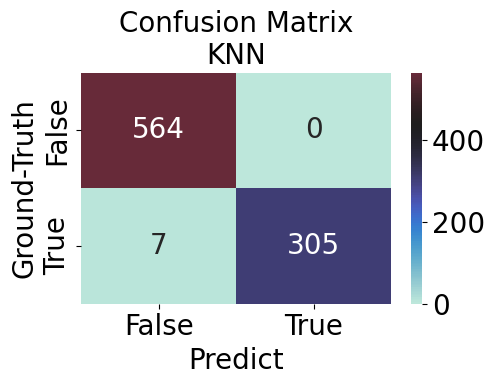

Supplement: Supplementary file 1 [file diagnostics-14-00053-s001.zip › Results of all classifiers/EmbeddingLR/KNN/Train Set/Confusion Matrix KNN.png]

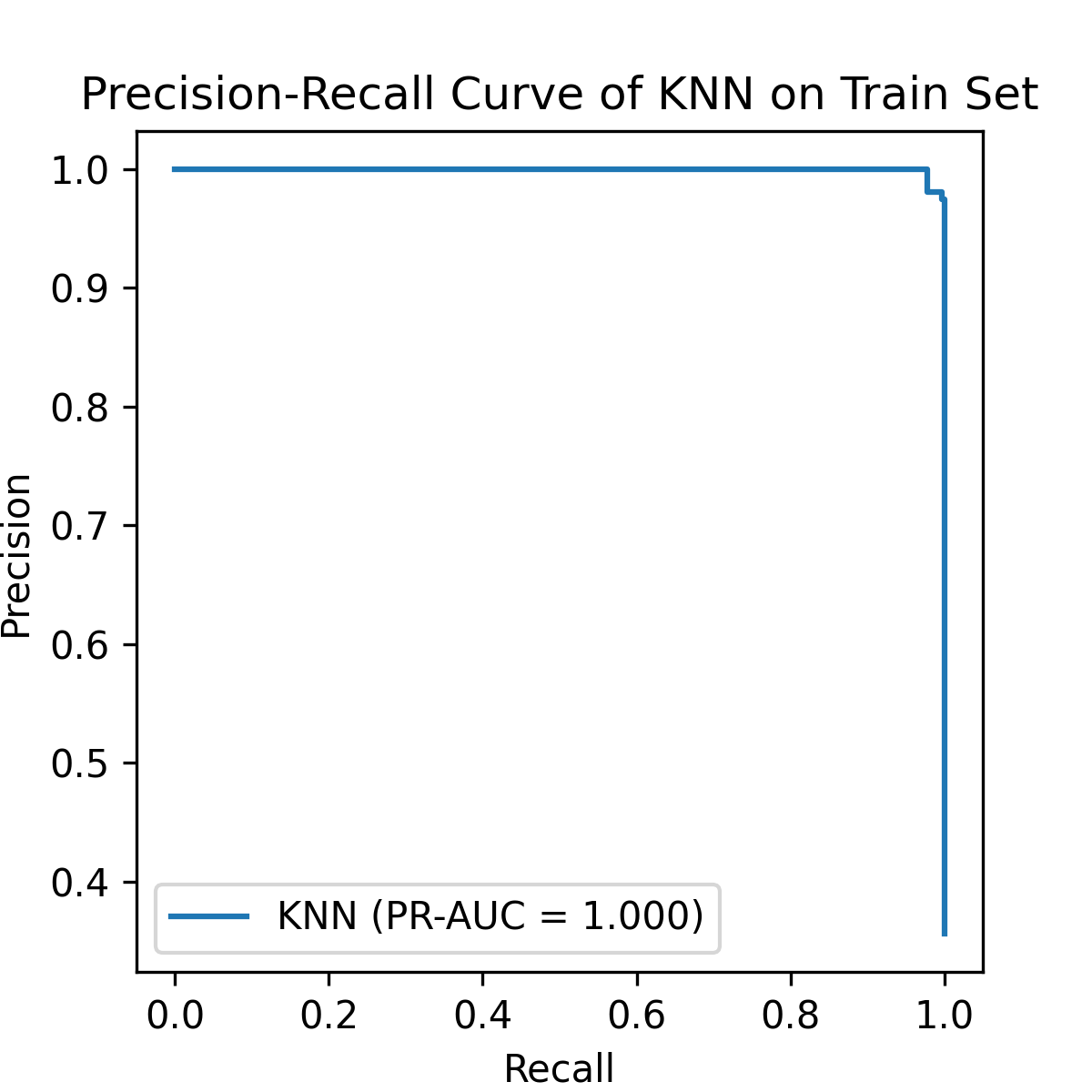

Supplement: Supplementary file 1 [file diagnostics-14-00053-s001.zip › Results of all classifiers/EmbeddingLR/KNN/Train Set/Precision-Recall Curve of KNN on Train Set.png]

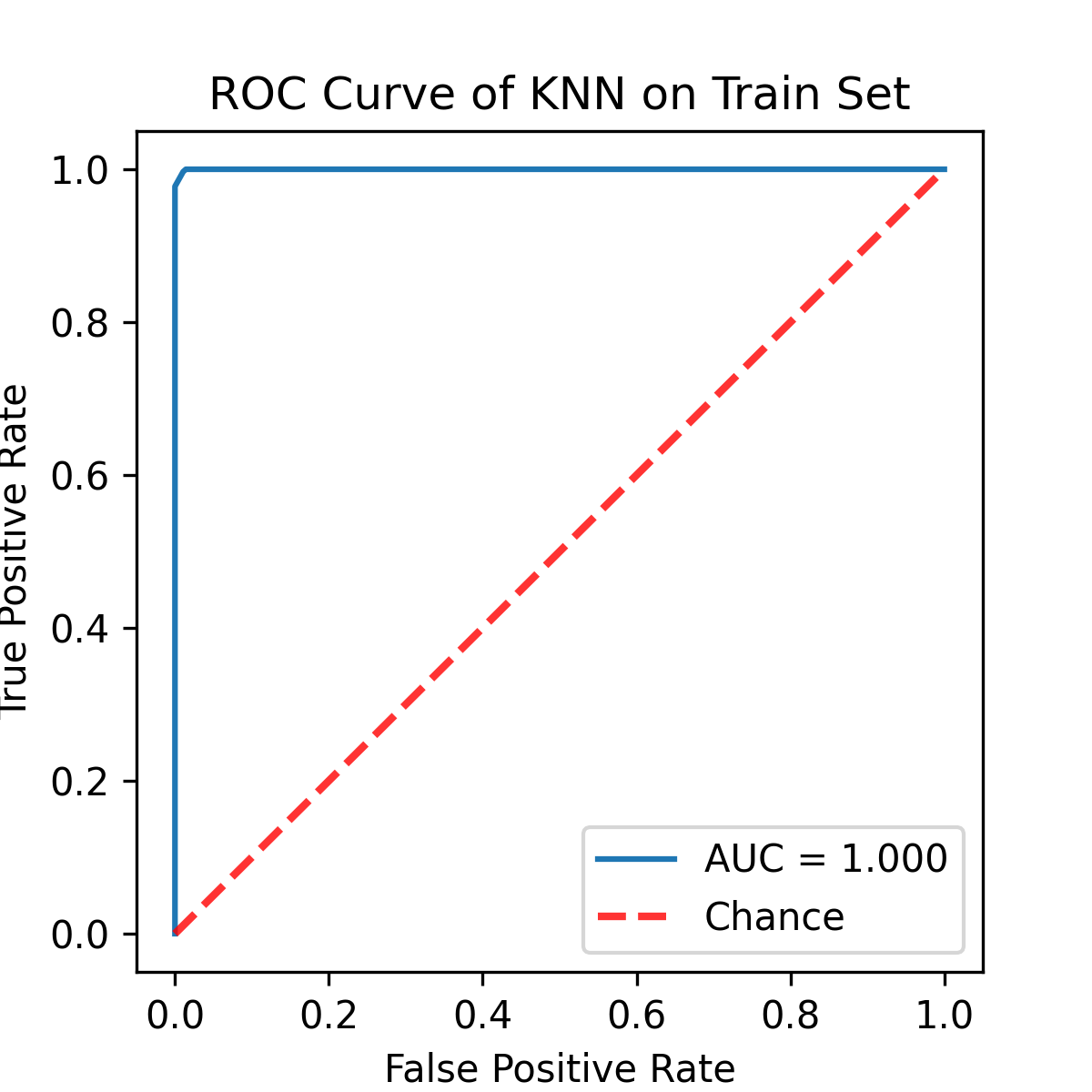

Supplement: Supplementary file 1 [file diagnostics-14-00053-s001.zip › Results of all classifiers/EmbeddingLR/KNN/Train Set/ROC Curve of KNN on Train Set.png]

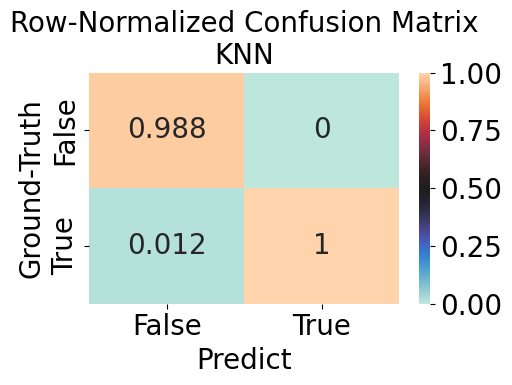

Supplement: Supplementary file 1 [file diagnostics-14-00053-s001.zip › Results of all classifiers/EmbeddingLR/KNN/Train Set/Row-Normalized Confusion Matrix KNN.png]

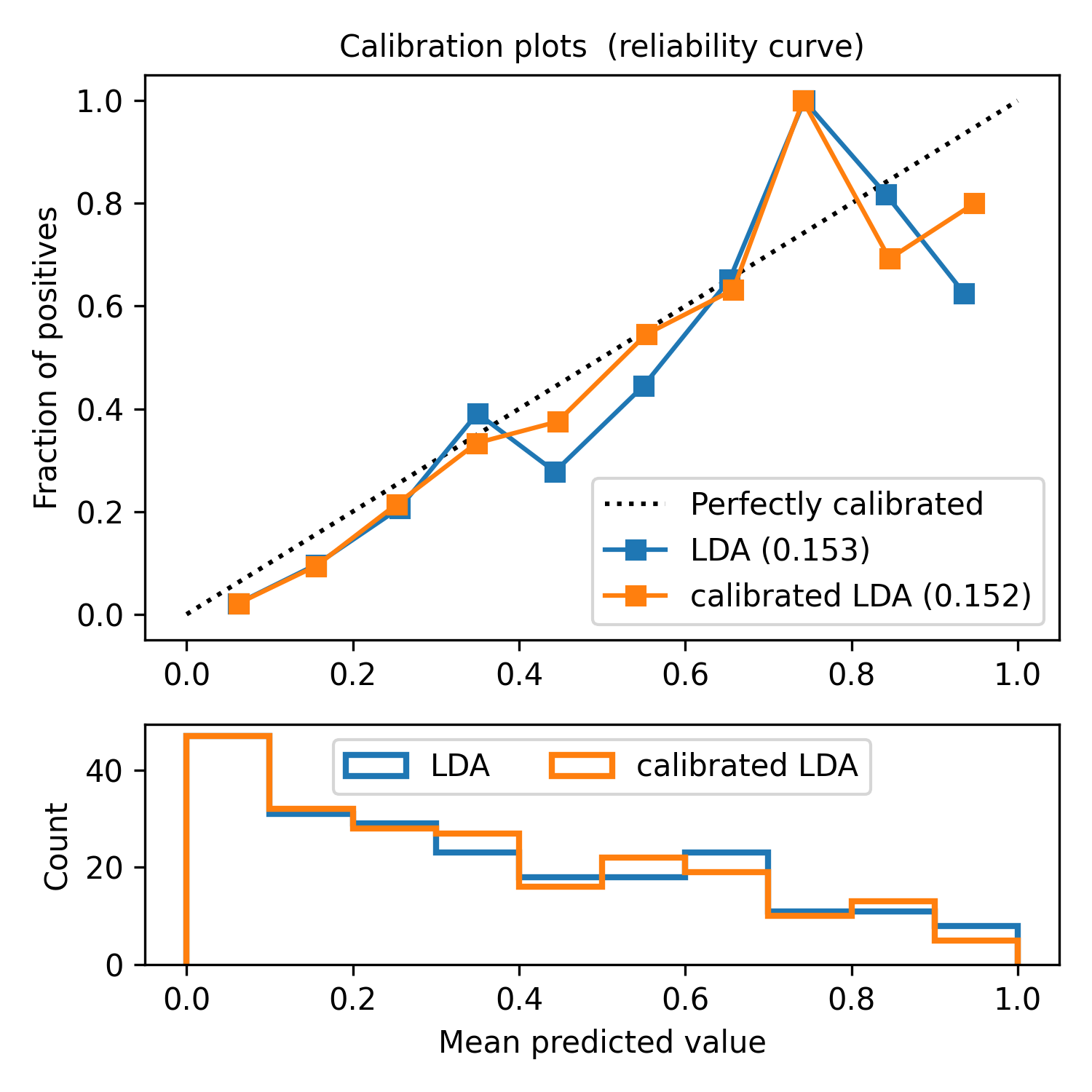

Supplement: Supplementary file 1 [file diagnostics-14-00053-s001.zip › Results of all classifiers/EmbeddingLR/LDA/Test Set/Calibration plots.png]

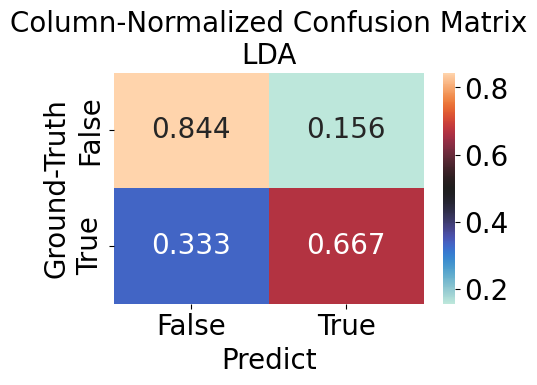

Supplement: Supplementary file 1 [file diagnostics-14-00053-s001.zip › Results of all classifiers/EmbeddingLR/LDA/Test Set/Column-Normalized Confusion Matrix LDA.png]

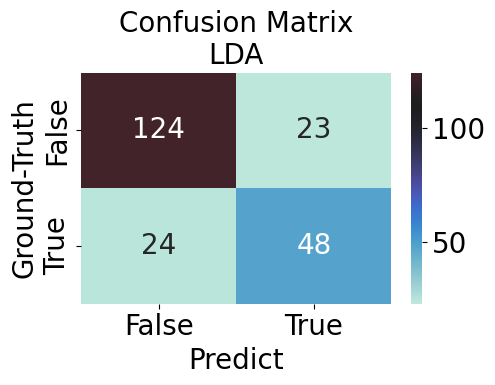

Supplement: Supplementary file 1 [file diagnostics-14-00053-s001.zip › Results of all classifiers/EmbeddingLR/LDA/Test Set/Confusion Matrix LDA.png]

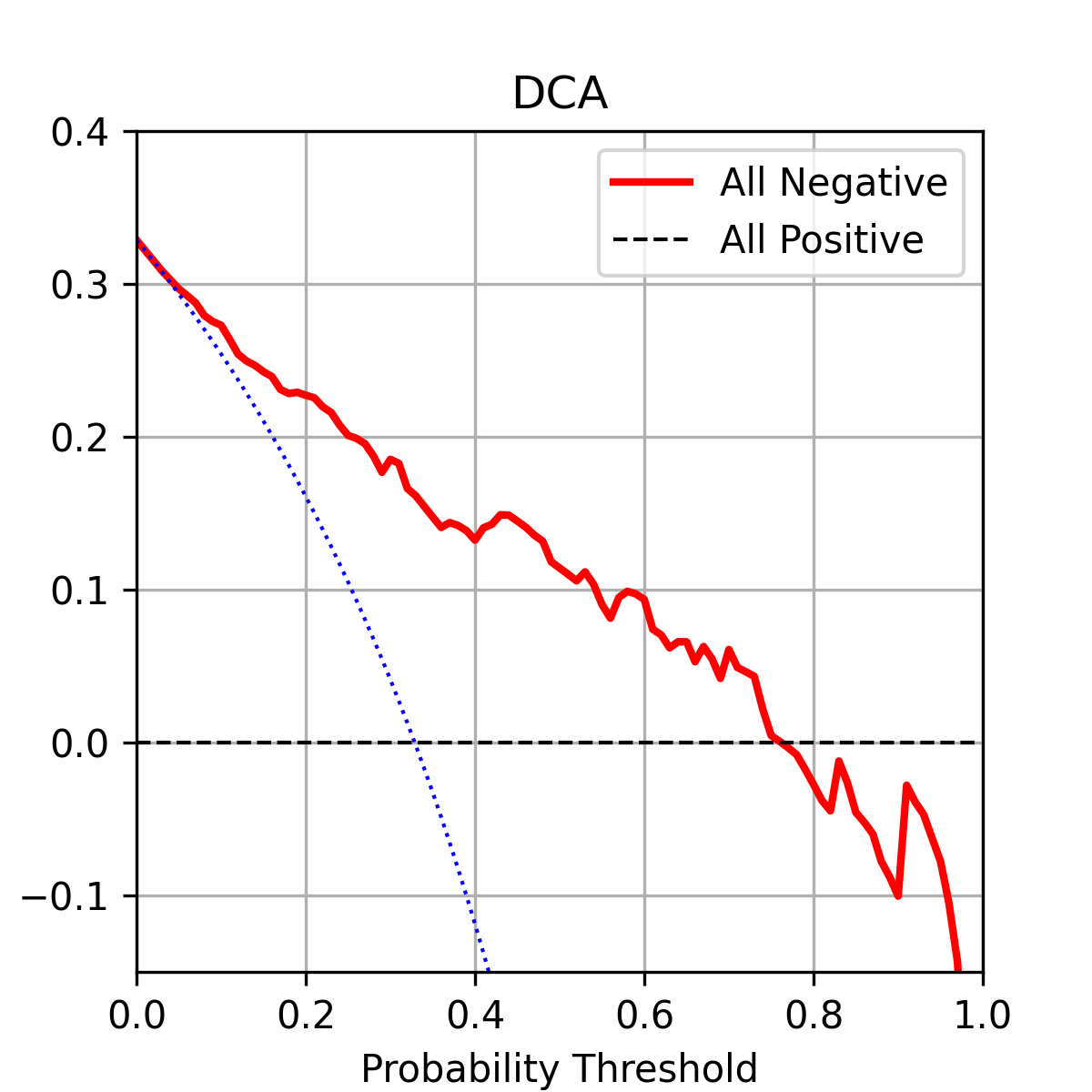

Supplement: Supplementary file 1 [file diagnostics-14-00053-s001.zip › Results of all classifiers/EmbeddingLR/LDA/Test Set/DCA.png]

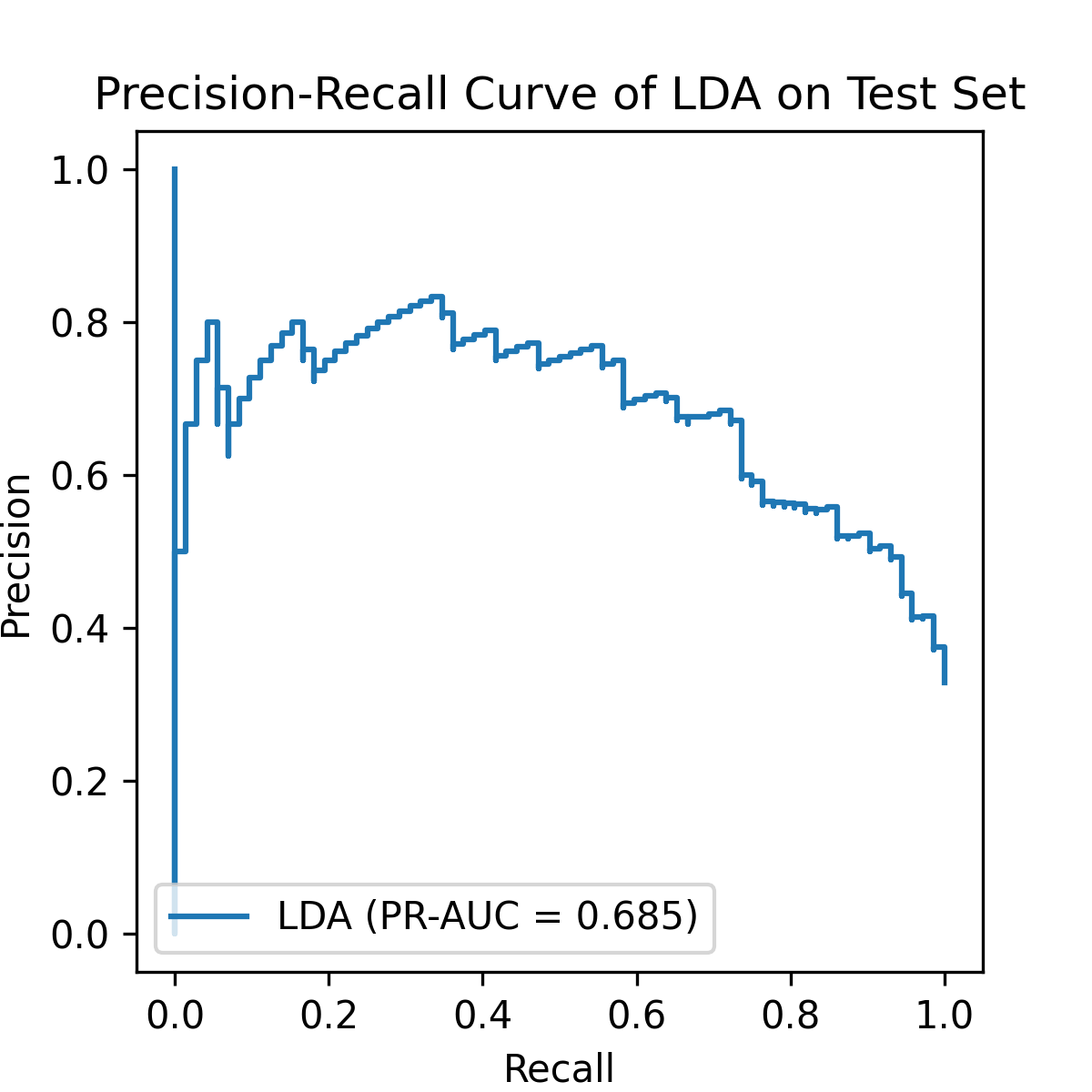

Supplement: Supplementary file 1 [file diagnostics-14-00053-s001.zip › Results of all classifiers/EmbeddingLR/LDA/Test Set/Precision-Recall Curve of LDA on Test Set.png]

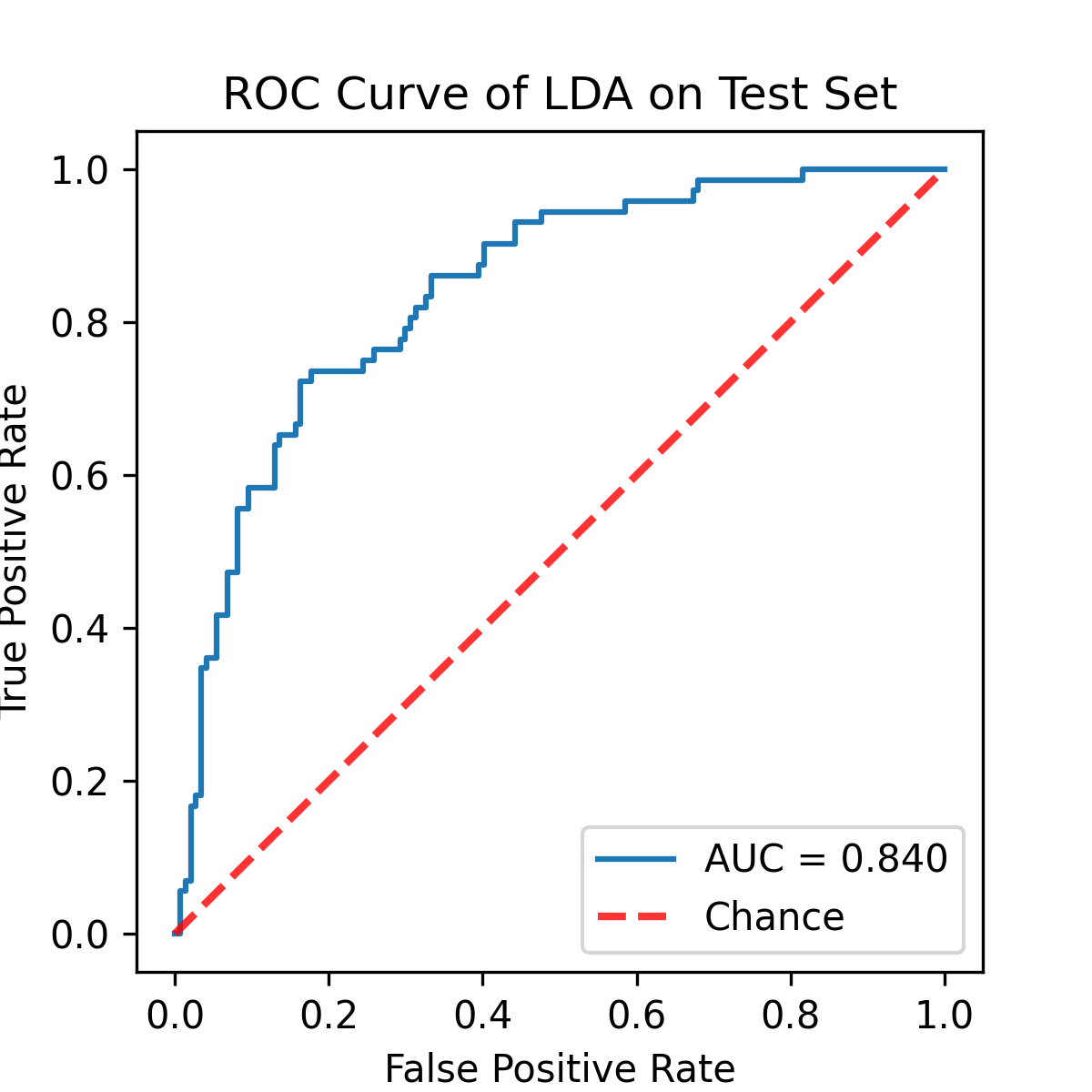

Supplement: Supplementary file 1 [file diagnostics-14-00053-s001.zip › Results of all classifiers/EmbeddingLR/LDA/Test Set/ROC Curve of LDA on Test Set.png]

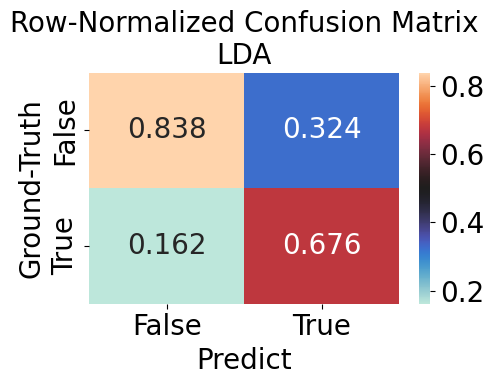

Supplement: Supplementary file 1 [file diagnostics-14-00053-s001.zip › Results of all classifiers/EmbeddingLR/LDA/Test Set/Row-Normalized Confusion Matrix LDA.png]
