# Supplementary material for: Machine Learning Predicts Decompression Levels for Lumbar Spinal Stenosis Using Canal Radiomic Features from Computed Tomography Myelography
Source: Diagnostics (Basel). 2023 Dec 26;14(1):53. doi: 10.3390/diagnostics14010053 (PMC10795799; doi:10.3390/diagnostics14010053)
Supplement: Supplementary file 1 [file diagnostics-14-00053-s001.zip › Table S1.The hyper-parameters used for model training.pdf]

Table S1. The hyper-parameters used for model training

Algorithms	Hyper-parameter settings
MLP	learning_rate: ['constant', 'invscaling', 'adaptive']<br><br>hidden_layer_sizes: [(32,),(64,),(128,),(32,32),(64,64),(128,128)]
gradient boosting	max_depth= [2,3,...,12]<br><br>n_estimators: [5, 10, 20, 40]
AdaBoost	estimator: [logistic regression, LDA, SVM, KNN,GaussianNB,decision tree]<br><br>n_estimators: [5, 10, 20, 40]
logistic regression	class_weight: ['balanced', None]
bagging	estimator: [logistic regression, LDA, SVM, KNN,GaussianNB,decision tree]<br><br>n_estimators: [5, 10, 20, 40]
LDA	/
RandomForest	max_depth: [5,6,...,15]<br><br>min_samples_leaf: uniform(loc=0, scale=0.1)<br><br>min_samples_split: uniform(loc=0, scale=0.1)<br><br>n_estimators: [5, 10, 20, 40]
extra trees	n_estimators: [5, 10, 20, 40]
SVM	kernel : ['rbf', 'sigmoid']<br><br>class_weight': ['balanced', None]
decision tree	max_depth: [5, 10, 20]

	min_samples_leaf: [2,4,8,16]<br><br>min_samples_split: [2,4,8,16]<br><br>class_weight: ["balanced",None]
KNN	n_neighbors:[2,5,10]<br><br>weights: ['uniform', 'distance']<br><br>p: [1,2]
GaussianNB	/
